# Monograph of *Coccinia* (Cucurbitaceae)

**DOI:** 10.3897/phytokeys.54.3285

**Published:** 2015-08-03

**Authors:** Norbert Holstein

**Affiliations:** 1 Nees-Institute for Biodiversity of Plants, Meckenheimer Allee 170, 53115 Bonn, Germany

**Keywords:** Cucurbitaceae, *Coccinia*, molecular phylogeny, biogeography, taxonomy, morphology, sex expression, useful plants

## Abstract

This monograph deals with all 95 names described in the Cucurbitaceae genus *Coccinia* and recognizes 25 species. Taxonomic novelties are Coccinia adoensis var. aurantiaca (C.Jeffrey) Holstein, **stat. nov.**, Coccinia sessilifolia var. variifolia (A.Meeuse) Holstein, **stat. nov.**, and Coccinia adoensis var. jeffreyana Holstein, **var. nov.** For the 25 species 3157 collections were examined, of which 2024 were georeferenced to produce distribution maps. All species are distributed in sub-Saharan Africa with one species, *Coccinia grandis*, extending from Senegal in West Africa east to Indonesia and being naturalized on Pacific Islands, in Australia, the Caribbean, and South America. *Coccinia* species are dioecious creepers or climbers with simple or bifid tendrils that occupy a range of habitats from arid scrubland, woodlands to lowland rainforest and mist forest. The corolla of *Coccinia* species is sympetalous, usually pale yellow to orange, and 1 to 4.5 cm long. Pollination is by bees foraging for pollen or nectar. After pollination, the developing ovary often exhibits longitudinal mottling, which usually disappears during maturation. All species produce berries with a pericarp in reddish colors (orange-red through to scarlet red), hence the generic name. The globose to cylindrical fruits contain numerous grayish-beige flat to lenticular seeds. Chromosome numbers are 2*n* = 20, 24, and 22 + XX/XY. Many *Coccinia* species are used for food, either as roasted tubers, greens as spinach, or the fruits as vegetables. Medicinal value is established in *Coccinia grandis*, of which leaves and sap are used against diabetes.

## Introduction

*Coccinia* Wight & Arn. comprises 25 species and is the 11^th^ largest of the 97 genera of the Cucurbitaceae (Schaefer and Renner 2011a). Especially in the 19^th^ century, it drew gardeners’ attention probably because of its striking fruits (André 1900; Edwards 1815; Huber 1864; 1865; Koch 1865; Sims 1816). All species are dioecious, and one species, *Coccinia grandis* (L.) Voigt, has heteromorphic sex chromosomes and therefore has been studied cytologically (Agarwal and Roy 1975; 1983; 1984; Bhaduri and Bose 1947; Chakravorti 1948; Datta 1988; Kumar and Deodikar 1940; Kumar and Vishveshwaraiah 1952; Roy 1974; Roy and Roy 1971b). The last complete taxonomic treatment of *Coccinia* is by Cogniaux (1881), more than 130 years ago. Since then, 16 new species have been described, and the genus has only been revised regionally (Hutchinson et al. 1954; Jeffrey 1967; 1978; 1995; Jeffrey and Fernandes 1986; Kéraudren-Aymonin 1975a; Kéraudren 1967; Meeuse 1962). The position of *Coccinia* in the Benincaseae has been confirmed by molecular data (Kocyan et al. 2007; Schaefer and Renner 2011b), and the monophyly has been tested with almost complete species sampling by Holstein and Renner (2011b).

The delimitation of *Coccinia* from other genera is difficult. The scarlet-red fruits to which the genus name – *Coccinia* from Latin *coccineus* – refers are also found in other African genera, such as *Eureiandra* Hook.f. Therefore, it is not surprising that early botanists described several species now considered to belong to *Coccinia* in other genera (*Cephalandra* Schrad. ex Eckl. & Zeyh., *Physedra* Hook.f., and *Staphylosyce* Hook.f.), or species described in *Coccinia* now belong to different genera. In all, 113 names at various ranks have been proposed for what are here considered 25 species. The species concepts in the present revision are based on 3157 herbarium collections and fieldwork in Tanzania, geo-referencing of 2024 collections and cultivation of 10 species in the greenhouse. In combination, plastid and nuclear data obtained for multiple accessions representing most species and ecological information coming from the mapping effort provide a modern understanding of the evolution and species relationships in *Coccinia*.

## Materials and methods

### General morphology

During this study, the present author examined 3157 herbarium collections from 39 herbaria from (physical or digital) loans or *in situ* (B, BM, BR, BRI, C, CANB, CBG, COI, DSM, EA, FR, FT, G, GAT, GOET, H, HBG, HEID, JE, K, L, LISC, LISU, M, MO, MSB, NHT, P, PERTH, PR, PRC, S, U, UBT, UPS, W, WAG, Z, ZT). Additional collections were obtained via personal communication (BO, DNA, TUB, US), from web pages such as like JStor Plant Science (JPS, http://plants.jstor.org/), Chinese Virtual Herbarium (CVH, http://www.cvh.org.cn/), and homepages of the following herbaria: A, AAU, BAR, CAY, FLAS, FSU, FTG, GH, HAST, NTUF, NY, PDA, TAIF, and USF. New collections were added if the photograph allowed identification or if misidentification appeared to be unlikely (esp. *Coccinia grandis* collections from the Pacific area), while duplicates were added without visual inspection of the specimen photo. Online availability of specimen images is mentioned in the list of exsiccatae (Suppl. material 1). Ten species were cultivated in greenhouses of Munich Botanical Garden: *Coccinia abyssinica* (Lam.) Cogn. (1 origin, 4 individuals), Coccinia adoensis var. jeffreyana (1 origin, 4 individuals), *Coccinia grandiflora* Cogn. (1 origin, 1 individual), *Coccinia grandis* (3 origins with 2, 61, 3 individuals, respectively), *Coccinia hirtella* Cogn. (1 origin, 12 individuals), *Coccinia megarrhiza* C.Jeffrey (1 origin, 8 individuals), *Coccinia microphylla* Gilg (2 origins, 2 and 1 individual, respectively), *Coccinia rehmannii* Cogn. with two varieties (2 origins, each 3 individuals), Coccinia sessilifolia (Sond.) Cogn. var. sessilifolia (1 origin, 8 individuals), and *Coccinia trilobata* (Cogn.) C.Jeffrey (2 origins, each 3 individuals). The present author performed crossing experiments among eight of these species. Morphological features were documented photographically and in the form of vouchers, with 47 collections deposited in M. Field data were obtained on a trip to NE Tanzania in 2009 resulting in 28 *Coccinia* collections.

### Phylogenies

For this monograph the phylogenetic data of Holstein and Renner (2011a; 2011b) were augmented with 20 new sequences from 8 accessions (GenBank accession numbers are given in Suppl. material 2), and new phylogenies were calculated using RAxML v. 7.2.6 (Stamatakis 2006) and MrBayes v. 3.2 (Ronquist et al. 2012). The substitution model was GTR+Γ as used before, and 1000 ML replicates were used to infer statistical support for the nodes via bootstrapping. For Bayesian analysis, four chains were run with 2,000,000 generations, with a sampling frequency of 1000. The first 25% of the trees were discarded as burn-in, and the rest were plotted as a 50% majority rule consensus tree using FigTree 1.3.1 (http://tree.bio.ed.ac.uk/software/figtree/). Gaps in the plastid matrix occurring in more than one accession were coded as “0”, “1”, or “?”, with “?” when data were missing or when shorter gaps were coded in the same place, but in different accessions.

### Distribution maps

Of the examined collections, 2024 were geo-referenced and mapped in Google Earth (Google Inc., Mountain View, CA, USA). Cultivated plants were geo-referenced according to the original collection site. If collecting sites were given as distances from locations, a path along major roads was used, beginning from the center of the starting location. Collecting sites were geo-referenced according to the description even if coordinates were given on the label, except for cases in which the coordinates were clearly derived from GPS or if the description did not allow further improvement. Location names were cross-validated from printed maps and then imported into DIVA-GIS 7.1.6.2 (http://www.diva-gis.org). If collecting sites of specimens appeared to be too useful to ignore in the distribution maps despite the large uncertainty of the position (radius > 5 km) or if the collecting site was only given as “nearby” a distinct locality, then geo-referenced coordinates are given in brackets. Political administrative borders were taken from GADM v.1 (Jan 2009) or v.2 (Jan 2012) (http://www.gadm.org/) and elevation data (1 km resolution) from CGIAR Consortium for Spatial Information (Jarvis et al. 2008). The geodetic datum for the maps is WGS84; the projection in each case is equirectangular. Coordinates are given in decimal degrees in Suppl. material 1 with WGS84 as geodetic datum.

## Morphology and anatomy

### Habit

*Coccinia* species are perennial climbers or creepers. The lignification of the mature shoots differs among the species from unlignified to completely lignified. Climbing is enabled by tendrils, which are either simple or bifid. Tendril development in young plants is delayed and emerges in *Coccinia abyssinica* after the 6^th^ node (Getahun 1974a). The tendril arms are only rarely equally sized, as one is usually much smaller; true dichotomy of tendrils is unknown from *Coccinia*. Whether a species has simple or bifid tendrils is often not fixed, but there is a strong predominance of one kind. Bifid tendrils regularly occur or are predominant in *Coccinia grandiflora* Cogn., *Coccinia heterophylla* (Hook.f.) Holstein, *Coccinia hirtella* Cogn., *Coccinia intermedia* Holstein, *Coccinia mackenii* Naudin ex C.Huber, *Coccinia mildbraedii* Gilg, *Coccinia racemiflora* Keraudren, *Coccinia schliebenii* Harms, and in some forms of *Coccinia barteri* (Holstein and Renner 2011b). Strikingly, *Coccinia* species with bifid tendrils occur in rather humid habitats. This suggests an adaptive advantage, because more tendril arms increase stability, as the leaves of rainforest species are larger, coriaceous, and thus heavier than leaves of species from drier habitats. Some species are regularly described as having simple tendrils in floristic treatments, but they may bare bifid tendrils such as *Coccinia sessilifolia* (*N. Holstein 13*) and *Coccinia senensis* (*H.J.E. Schlieben 5745* in B, K, and MO). *Coccinia adoensis* has bifid tendrils even in some type specimens (e.g., *G.H.W. Schimper 166* in BR8886781 and on the sheet with a drawing in K) and is still listed as simple-tendrilled. All three species with this polymorphism, however, have predominantly simple tendrils. Interestingly, these species are also closely related to species with predominantly bifid tendrils: *Coccinia sessilifolia* with *Coccinia hirtella* and *Coccinia mackenii*, and *Coccinia adoensis* with *Coccinia grandiflora* and *Coccinia schliebenii*.

### Roots

**Figure 1.**
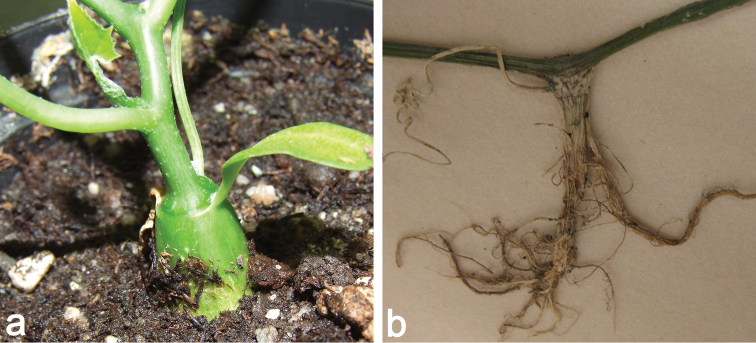
**a** Young plant of *Coccinia grandis*. The hypocotyl is already thickened and lignifies later-on to a tuber. The cotyledons are glabrous, have an entire margin and an obtuse apex **b** Adventitious root on *Coccinia grandis*; the beige structure to the left is a dry tendril.

*Coccinia* species have perennial roots. Most (if not all) species are woody at the base, and most of them produce hypocotyl tubers (Fig. 1a). Some species, such as *Coccinia adoensis* and *Coccinia grandiflora* (and most likely also *Coccinia senensis* (Klotzsch) Cogn. and *Coccinia schliebenii*), however, produce globular subterranean root tubers, much like potatoes, but smaller in size (Holstein and Renner 2011b; Zimmermann 1922b; pers. observ.). Root tubers in *Coccinia adoensis* are likely to be an adaptation to fire, as this species predominantly occurs in woodlands. In contrast to rather mild fires in semi-arid savannas with less inflammable biomass, woodland fires produce temperature rises of 60 °C in 0–3 cm depth (Bradstock and Auld 1995; Gignoux et al. 1997), so vegetative buds near the ground (hemicryptophytes) might be damaged, whereas root tubers (geophytes) have a higher chance of survival.

*Coccinia grandis* and *Coccinia barteri* produce adventitious roots if stems touch the soil (Fig. 1b). *Coccinia hirtella*, *Coccinia sessilifolia* and their F1 hybrids with *Coccinia grandis* appear to lack this ability (pers. observ.). Adventitious roots also occur along the hypocotyl of *Coccinia abyssinica* seedlings (Getahun 1974a). There is barely any research on root anatomy, solely Getahun (1974a) reports tetrarch vascular bundles in the primary roots of *Coccinia abyssinica* seedlings and di- to triarch bundles in secondary roots.

### Hypocotyl and shoots

Many species, such as *Coccinia abyssinica*, *Coccinia grandis*, *Coccinia hirtella*, *Coccinia megarrhiza*, *Coccinia microphylla*, *Coccinia rehmannii*, *Coccinia sessilifolia*, and *Coccinia trilobata*, produce a lignified tuber that is derived from the hypocotyl (Zimmermann 1922b; pers. observ., Fig. 1a). The tuber, at least of some species, contains starch as a storage nutrient (Getahun 1974b). It develops during the first season, and lignification may begin as soon as the appearance of the first tendrils, such as in *Coccinia abyssinica* (*N. Holstein 132*). Some species, such as *Coccinia adoensis* and *Coccinia grandiflora* (and most likely also *Coccinia senensis* and *Coccinia schliebenii*) do not produce hypocotyl tubers but root tubers. In Coccinia adoensis var. jeffreyana the hypocotyl is minute (*N. Holstein 130*), which prevents the development of a tuber. Whether West and Central African forest species produce tubers is unknown.

**Figure 2.**
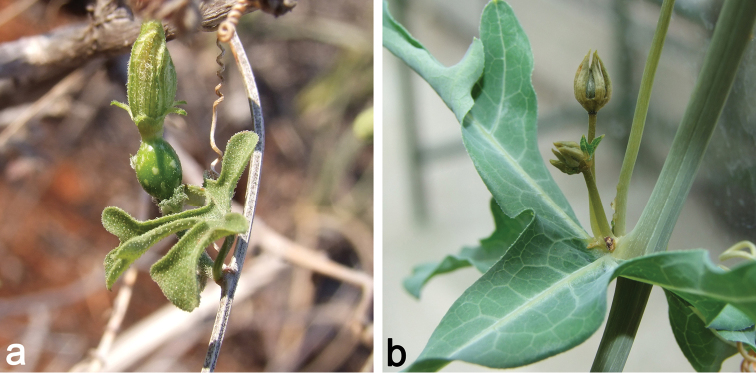
**a** Female flower bud of *Coccinia microphylla* (*N. Holstein et al. 90*); picture taken during the dry season. The stem is completely lignified, and only green short shoots are produced **b** Male plant of *Coccinia sessilifolia*. The stem is glaucous and does not lignify. Unusually, the bract is 3-lobate leaf-like.

**Figure 3.**
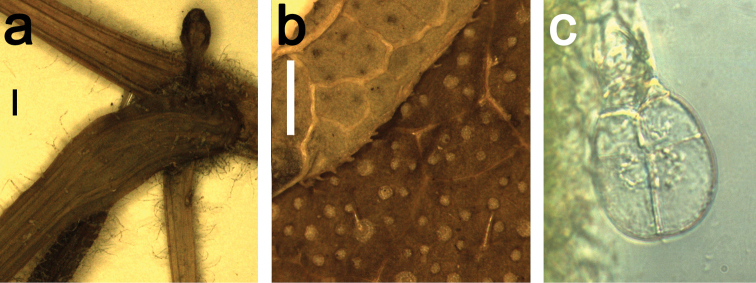
Trichomes on *Coccinia* species. **a** Node of Coccinia adoensis var. jeffreyana (*J.C. Lovett 1197*). The black bar equals 1 mm. **b** Part of a leaf Coccinia adoensis var. jeffreyana (*A.F. Stolz 504*). The white bar equals 1 mm. **c** Glandular trichome from *Coccinia grandis*.

Each plant produces one to several shoots, which can persist or die back completely during the dry season or due to fire or grazing. *Coccinia microphylla* shoots can lignify completely and produce short green branches with flowers and small leaves during the dry season (Fig. 2a), whereas shoots of *Coccinia sessilifolia* do not lignify at all (pers. observ. from greenhouse cultivation over 4 years). The shoots of *Coccinia grandis* can become slightly succulent. The length of the shoots varies from 70 cm in *Coccinia microphylla* to 20 m in *Coccinia grandiflora* and *Coccinia mildbraedii*. Zimmermann (1922a) reports a stem of *Coccinia grandiflora* being 6 cm in diameter. Usually, the bark of the hypocotyl tuber and the shoots is grayish in color. Fresh shoots and twigs are usually deep green to brownish green, sometimes speckled with pale to whitish pustules. In *Coccinia abyssinica* and *Coccinia megarrhiza* the shoots and tendrils can turn purple during maturity. *Coccinia sessilifolia* produces glaucous shoots that bear a waxy bloom (Fig. 2b). The indumentum of *Coccinia* species, if present, is composed of simple, oligo- to multicellular eglandular trichomes up to 2 mm in length. The long trichomes consist of oblong cells that may appear articulate when dried (Fig. 3a). Shorter trichomes can be lineal to conical (Fig. 3b). Sometimes, trichomes have a thickened base that appear warty when the trichomes break off. The density of the trichomes is often increased on the nodes. Trichome type and length on shoots are like those of the abaxial surface of the petioles, but usually less dense. Young shoots often exhibit short (< 0.5 mm), weak trichomes, even in species that are later glabrous, e.g., in *Coccinia grandis* or *Coccinia sessilifolia*. Glandular trichomes are rare, few-celled, not visible with the naked eye and have been found, e.g., in *Coccinia grandiflora* and *Coccinia grandis* (pers. observ.; Thanki 1989; Fig. 3c). Glandular trichomes are also observed in young stems of *Coccinia abyssinica* (Getahun 1974a), which are usually covered with long multicellular eglandular trichomes.

### Cotyledons

**Figure 4.**
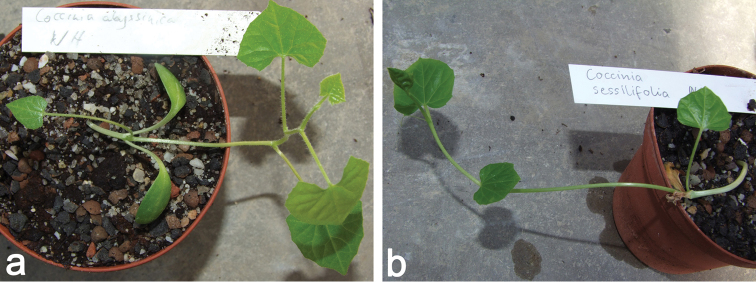
**a** Young plant of *Coccinia abyssinica* (*N. Holstein 132*). The first node is in the axilla where the glabrous cotyledons split off. The first nodes lack probracts and tendrils **b** Young plant of *Coccinia sessilifolia* (*N. Holstein 131*). The first leaves in this species are petiolate, sessile leaves are produced later-on. The glabrous cotyledons are already dried (plant had the same age as the one in Fig. 4a).

Zimmermann (1922b) reports epigeous cotyledons for *Coccinia grandiflora* and *Coccinia grandis*, of which the latter is confirmed by personal observations (Fig. 1a). Epigeous cotyledons also occur in *Coccinia abyssinica*, Coccinia adoensis var. jeffreyana, *Coccinia microphylla*, Coccinia rehmannii aff. var. littoralis, and *Coccinia sessilifolia*. The hypocotyl and cotyledons of all observed taxa are glabrous. The cotyledons are elliptical to obovate and have an entire margin. The cotyledons are slightly fleshy and green, which is also observed in those of *Coccinia abyssinica* (Getahun 1974a), and the cotyledonous apex has a pale marking and is obtuse to retuse. Getahun reports that the prominent veins and the margins on the lower cotyledon surface in *Coccinia abyssinica* are covered with multicellular trichomes. However, prominent veins in *Coccinia abyssinica* cotyledons cannot be confirmed, and if multicellular trichomes occur, then they are not visible to the naked eye. The first normal leaf in this species, however, emerges in the axilla of the cotyledons (*N. Holstein 132*, Fig. 4a), and thus might have been confused.

### Leaves

**Figure 5.**
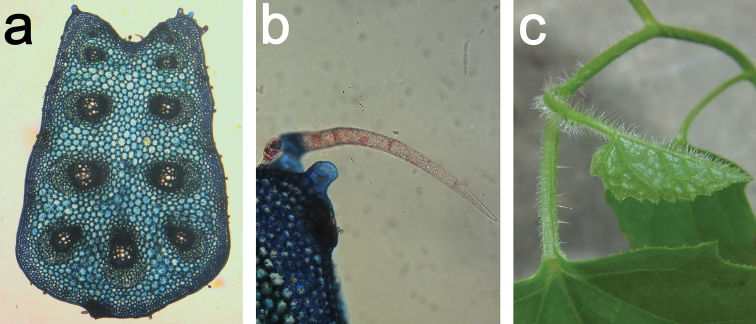
**a** Cross-section of a petiole of *Coccinia grandiflora*, stained with astra blue and safranin (3:2). The bicollateral vascular bundles are arranged in a U-shape **b** Trichome on the adaxial ridge of a cross-section of a petiole of *Coccinia grandiflora*. Although not visible by naked-eye, the petiole is also covered with few-celled glandular trichomes **c** Young plant of Coccinia adoensis var. jeffreyana. The trichomes are mainly occurring on the prominent veins. The adaxial side of the petiole bears smaller trichomes on the ridges, which fade into the leaf margin.

The leaves of *Coccinia* species are simple, alternate, and paired with a tendril on each node, except for the first nodes (Figs 1a, 4a, b). Leaves of all species are petiolate, except for Coccinia sessilifolia var. sessilifolia, which only develops petioles when young (*N. Holstein 131*, Fig. 4b) or rarely subsessile leaves when older; full petioles in this species are only realized in Coccinia sessilifolia var. variifolia (A.Meeuse) Holstein. Subsessile leaves are common in *Coccinia quinqueloba* and *Coccinia senensis*, while the other species’ leaves are usually distinctly petiolate. The petioles’ surface can be glabrous, at maturity speckled with hyaline to white cell clusters (*Coccinia grandis*, *Coccinia heterophylla*, *Coccinia intermedia*, *Coccinia quinqueloba*, *Coccinia rehmannii*, *Coccinia samburuensis*, *Coccinia senensis*, *Coccinia subsessiliflora*), or have an indumentum. The petiole contains several vascular bundles arranged in a U-shape (Fig. 5a). However, Hussain et al. (2011) report a ring of vascular bundles in *Coccinia grandis*. The adaxial side of the petiole often bears two ridges above the “lateral” vascular bundles (Fig. 5a). These ridges merge into the leaf margin and usually bear trichomes (Figs 3b, 5b, c). The abaxial side of the petiole shares its indumentum with the lower leaf lamina, at least at the base of the veins (Fig. 5c).

The venation in *Coccinia grandis* is reticulate, and the mid rib is reported to contain three bicollateral vascular bundles with xylem and phloem arranged in a ring (Hussain et al. 2011). Reticulate venation can be confirmed for all *Coccinia* species except *Coccinia ogadensis*, in which only the central vein in each lobe is visible.

**Figure 6.**
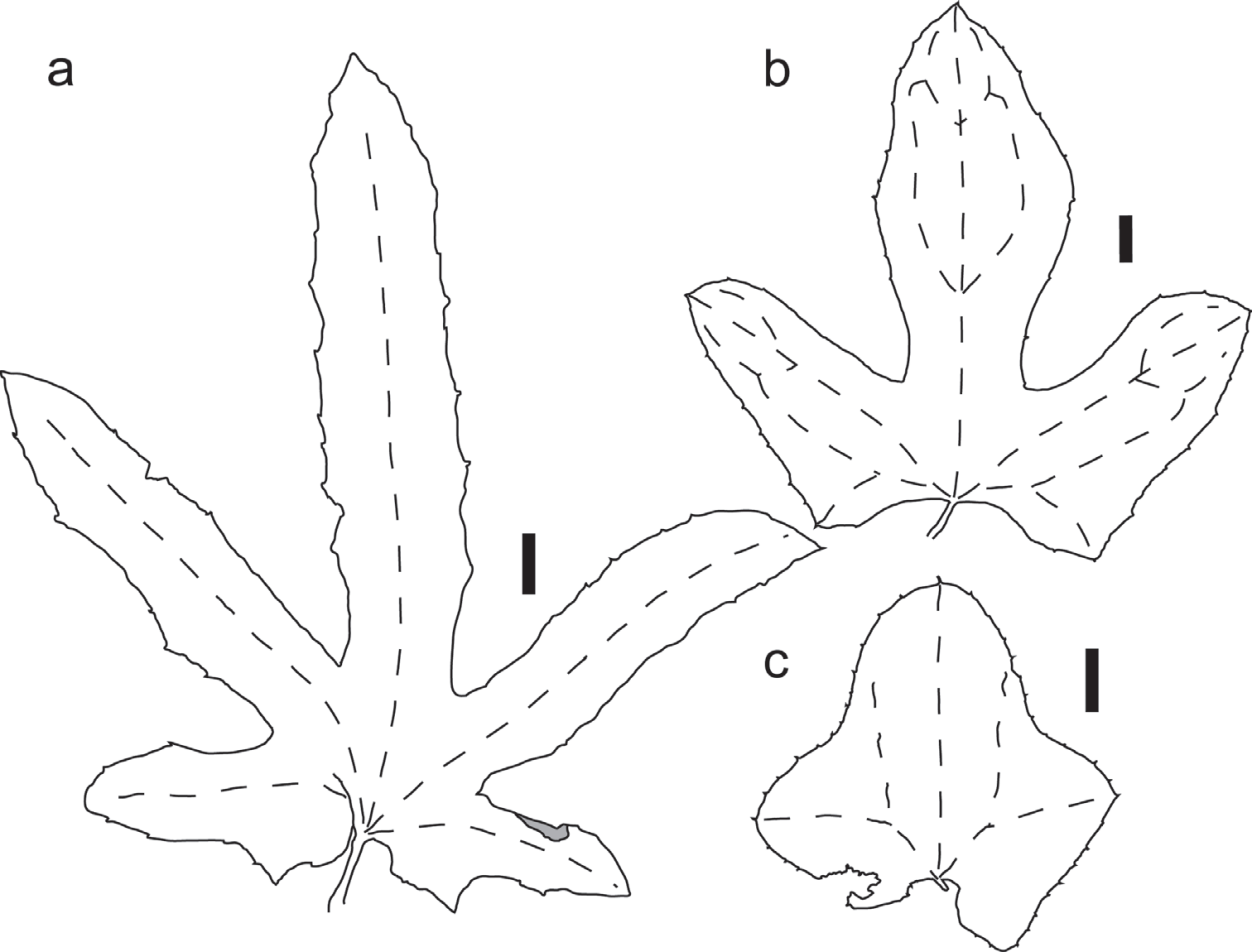
Leaf shape variability in *Coccinia senensis*. **a** *H.J. Schlieben 5259* (HBG) **b** *E.M.C. Groenendijk et al. 1031* (WAG) **c** *A.R. Torre et al. 18788* (MO). Black bars equal 1 cm.

**Figure 7.**
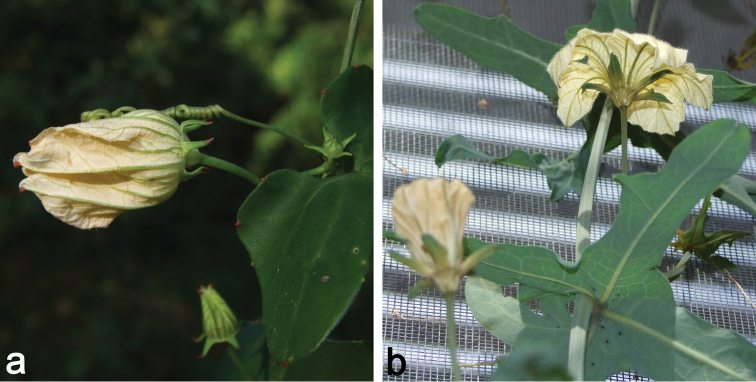
**a** Male flower of *Coccinia grandis* (*N. Holstein 37*). Apices of the petals and calyx lobes, as well as major teeth on the leaf margin are colored in red. Minor margin teeth are inconspicuously colored. The calyx lobes in *Coccinia grandis* are spreading in flower buds and reflexed in mature flowers **b** Male plant of *Coccinia sessilifolia*. Darkish glands (extranuptial nectaries) are commonly found at the base of a lower leaf lamina. The calyx lobes are unusually large in this specimen (cp. Fig. 2b).

Young leaf buds often bear a dense indumentum, even in species that are glabrous at maturity, e.g., in *Coccinia grandis*. The leaf lobes are linear, elliptic, (ob-)ovate to triangular. The incision depth of the lobes can be consistent (*Coccinia ogadensis*, *Coccinia subsessiliflora*) or highly variable (*Coccinia adoensis*, *Coccinia grandis*, *Coccinia senensis* (Fig. 6)), even within a single individual. In taxa with a variable degree of lobation, young leaves tend to be not or shallowly lobed (e.g., *Coccinia grandis*, *Coccinia megarrhiza*, Coccinia rehmannii aff. var. littoralis, *Coccinia sessilifolia*), a differentiation according to light exposure might also be possible. The leaf margin usually is beset conspicuously with small teeth and often bears trichomes (Figs 3b, 5b, c), even in otherwise glabrous species (e.g., *Coccinia grandis*, *Coccinia sessilifolia*). The teeth are at the apex of lobes, lobules and smaller orders of serration or situated along the entire margin. The term “dentate” (toothed) is therefore ambiguous in literature describing *Coccinia*, as it might also refer to the margin morphology (Stearn 2004). The teeth are often pale, but can also be colored, esp. when dry, such as in *Coccinia abyssinica*, *Coccinia grandis*, *Coccinia intermedia*, *Coccinia longicarpa*, *Coccinia megarrhiza*, and *Coccinia samburuensis* (Fig. 7a). The coloration of teeth is inconspicuous in young plants and develops during maturation (as observed in *Coccinia abyssinica*, *Coccinia grandis*, and *Coccinia megarrhiza*). The teeth are interpreted as hydathodes by Zimmermann (1922a), because he observed water drops in *Coccinia grandis* and *Coccinia trilobata* on the teeth of the 2^nd^ order (except those of the tip of the lobes) in the morning. A white deposit at the teeth on the upper side of the leaf of a *Coccinia adoensis* plant (*P. Quarré 75*; PR) seems to support Zimmermann’s interpretation.

The upper leaf lamina is often covered with transparent to white pustules, that contain cystoliths (Avetta 1894; Solereder 1899; Zimmermann 1922a; pers. observ.). The pustules consist of up to 25 cells in *Coccinia mackenii* (Avetta 1894) but are larger and denser in glabrous species from dry habitats (esp. *Coccinia ogadensis*). As they develop over time (they are smaller and less well visible in forest species), it can be assumed that the pustules are an adaptation towards protection against high solar radiation. When acetic acid is applied to microscopic sections of the leaves, heavy gas development suggests that the cystoliths consist of CaCO_3_ (pers. observ.). This can be observed in *Coccinia grandis*, *Coccinia hirtella*, and *Coccinia sessilifolia*, hence also when the pustules are not conspicuous as in the latter two species. The pustules may form the base of small trichomes, such as in Coccinia adoensis var. jeffreyana (Fig. 3b) or *Coccinia microphylla*. In some species, the upper surface is usually covered with an indumentum (*Coccinia hirtella*, *Coccinia schliebenii*, *Coccinia senensis*, and *Coccinia trilobata*), but it may also be reduced, and other species rarely exhibit a trichome-bearing upper surface, e.g., *Coccinia adoensis*. In each case, the trichomes are simple, < 1 mm, and whitish. The veins on the upper surface are either glabrous to the naked eye or are covered with small < 0.5 mm long simple trichomes. Zimmermann (1922a) observed in *Coccinia grandis* that the glabrous surface of the lamina is only slightly wettable, whereas a drop of eosine disperses along the veins rapidly. Zimmermann argues that these “capillary drainage lines” might serve to transport water to the hydathodes during the dry season.

The lower leaf lamina is paler than the upper side (Fig. 3b) and can be glabrous or bear an indumentum. The highest density of the indumentum can be found on the prominent veins (Fig. 5c). The indumentum on the lower leaf surface and the abaxial surface of the petiole can consist of eglandular oligo- to multicellular trichomes. The trichomes are appressed or upright (Fig. 5c), usually filiform, sometimes also narrowly conical (e.g., *Coccinia abyssinica*). Filiform trichomes are straight, curved, or sinuate. Long filiform trichomes often appear articulate when dry due to sunken lateral cell walls (Fig. 3a). Dry trichomes are hyaline, whitish, beige, or yellowish. The lower lamina often displays deeply colored to dark green to blackish extranuptial glands (Fig. 7b). The glands usually occur at the base of the leaf between the veins, sometimes also between secondary ramifications (*Coccinia grandis*) or along the main veins (*Coccinia grandiflora*). The epidermis of the lower leaf lamina in *Coccinia grandis* consists of cells with undulating anticlinal cell walls and anomocytic stomata (pers. observ.).

### Probracts and bracts

**Figure 8.**
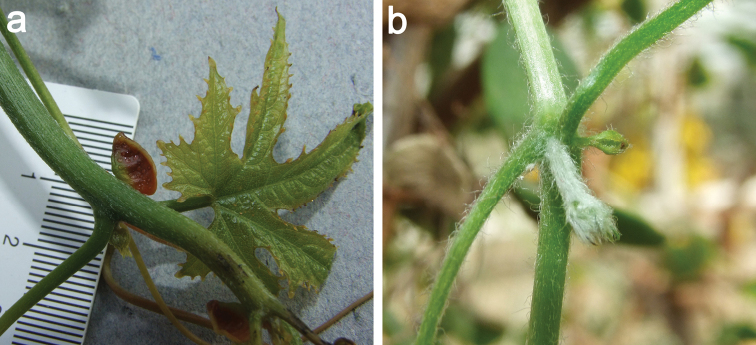
**a** The probract of *Coccinia grandiflora* is fleshy and has a keel. The abaxial side bears many extranuptial nectaries. The structure pointing to the lower left of the picture is the tendril **b** The probract of *Coccinia trilobata* (sampled as *N. Holstein & P. Sebastian 9*) is spoon-shaped, papery, and without a keel.

In addition to the foliose leaves, most *Coccinia* species have bracteose prophylls on sterile nodes, which are called “probracts” (Zimmermann 1922b). The probracts can be up to 5 mm long, but also rather small (< 1 mm) or caducous. The first nodes of the seedling lack probracts, and they are developed on later nodes. The shape of the probracts is ovate and entire with a round to acute apex. They are often spoon-like presenting the lower surface (e.g., *Coccinia adoensis*, *Coccinia barteri*, *Coccinia grandis*, *Coccinia megarrhiza*, *Coccinia sessilifolia*; Figs 3a, 8b), or they are folded in the middle with a prominent keel (*Coccinia grandiflora*; Fig. 8a). Probracts can be glabrous or bear short (< 1 mm) trichomes, and bear extranuptial glands on the lower surface (Fig. 8a; Okoli and Onofeghara 1984).

Bracts (leaves subtending inflorescences or flowers), if present, look like the probracts. Bracts below inflorescences are as large as probracts, while bracts below flowers tend to be smaller. Bracts can be present or absent, the latter being an indicative character for some species.

In rare cases, probracts and bracts can be leaf-like (e.g., *N. Holstein 126*, Fig. 2b; *P.C.M. Jansen 2065*; *H. Wanntorp & H.E. Wanntorp 1159*) indicating that the (pro-)bracts are likely derived from usual leaves.

### Extranuptial glands

The conspicuous glands on the lower leaf surface, probracts, and bracts (Figs 7b, 8a) are of the *Benincasa*-type (sensu Zimmermann 1932), meaning that they are flat and consist of several layers of secretory cells, which are surrounded by a single-layered sheath (Muhammad Ilyas 1992; Okoli and Onofeghara 1984; pers. observ.). This sheath is lignified in *Coccinia microphylla* and *Coccinia trilobata* (Zimmermann 1922b). However, Zimmermann (1932) cites Nieuwenhuis von Üxküll-Güldenbandt as saying that the sheath in *Coccinia grandis* is suberinized, but the present author did not find such a statement in the citations given in that paper (eventually, this was a personal communication). Schrödter (1926), however, finds that young sheaths in *Luffa aegyptiaca* are lignified but become suberinized with age, so the difference might be explained by different stages. Chakravarty (1948) interprets the sheath as filter tissue that is surrounded by an “external osmotic tissue”. Also Muhammad Ilyas (1992) interprets these radially elongated cells as secretory and notes that they have a connection to the vascular strand. However, Okoli and Onofeghara (1984) find that the glands in *Coccinia barteri* are too distant to be interpreted as vascularized. Zimmermann (1922b) observes intermediate forms between few-celled, stalked glandular hairs and the *Benincasa*-type glands in *Coccinia microphylla* and *Coccinia trilobata*, including the sheath that forms the base of the protruding glandular tissue. The glands secrete a clear, rarely slightly colored, sweet-tasting exudate (pers. observ. in *Coccinia grandiflora* and *Coccinia grandis*). In *Coccinia grandis* the exudates contain sucrose, glucose, fructose, alanine, tryptophane, threonine, and an unidentified amino acid (Muhammad Ilyas 1992).

### Peduncles and pedicels

Male flowers mostly occur in racemes that are usually accompanied by 1–2 solitary flowers on the same node (Fig. 2b). The first flowers in male plants of *Coccinia hirtella*, *Coccinia rehmannii*, and *Coccinia sessilifolia* are solitary. Racemes appear later in the course of the flowering season, although racemes are generally rare in the first species (pers. observ.). If solitary flowers and racemes are produced on the same node, then the solitary flower(s) precede(s) those of the racemes in time of maturity (Fig. 2b). The trigger to produce racemes instead of or additionally to solitary flowers is not known. The racemes bear up to 35 flowers (e.g., in *Coccinia pwaniensis*, *Coccinia racemiflora*). Within the racemes, flowering starts at the basalmost branches. If the peduncle is reduced, flowers appear clustered on the node. In *Coccinia grandis*, which usually produces single flowers only, flower clusters (short-peduncled racemes) rarely occur. This can be seen in plants from Ethiopia, Saudi Arabia but also from India and Sri Lanka. The pedicels of solitary male flowers of *Coccinia hirtella*, *Coccinia megarrhiza*, and *Coccinia rehmannii* exhibit a negative gravitropism. In creeping plants, pedicels that grow downwards in the beginning make a sharp bent upwards to present the flower upright.

Female flowers are mostly solitary. Only in some species, female flowers are usually in racemes, such as in *Coccinia heterophylla*, *Coccinia keayana*, and *Coccinia racemiflora*. Few-flowered female racemes or clustered flowers might also occur in *Coccinia grandiflora*, *Coccinia intermedia*, and *Coccinia subsessiliflora*. In *Coccinia barteri*, female flowers can be solitary or in few- or many-flowered racemes. Two female flowers per node have also been observed in *Coccinia microphylla*. The pedicels of solitary female flowers are negatively gravitropic during flower development. After pollination, the pedicels of solitary female flowers of *Coccinia grandis*, *Coccinia hirtella*, *Coccinia megarrhiza*, *Coccinia microphylla*, *Coccinia rehmannii*, and *Coccinia sessilifolia* exhibit positive gravitropism. The downturn is not due to slackness caused by the weight of the developing fruit but an active process, as the pedicels thicken and remain firm. However, only fertilized flowers turn downwards completely, as aborted flowers from mispollination never reach this state (pers. observ. in cultivated plants).

### Perianth

The perianth of all *Coccinia* species is synsepalous and sympetalous. At the base, calyx tube and corolla tube are connected with each other and form a perianth tube or funnel. Depending on the exsertion point of the staminodes in female flowers, parts of the tube form a hypanthium (e.g., *Coccinia grandiflora*).

**Figure 9.**
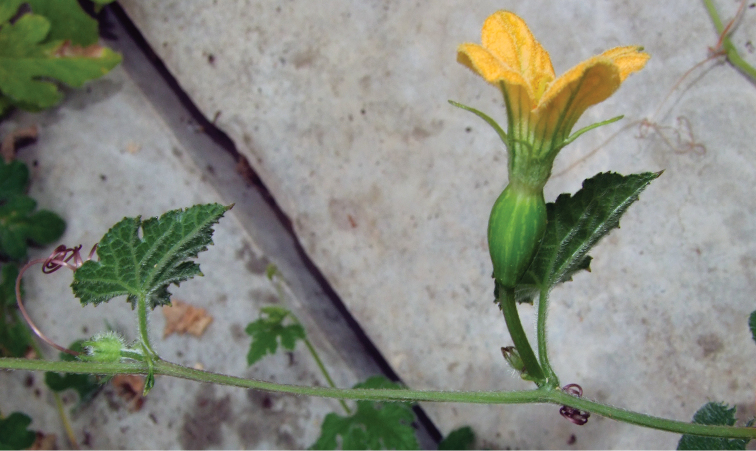
Leaky dioecy in a plant of *Coccinia megarrhiza*. The plant was flowering male (the bud on the left) through the season, but a single female flower developed (the second flower on this node was male). The flower was receptive and produced a normal-sized fruit and normal-shaped seeds. The probracts (left node) are spoon-shaped, the tendrils are purplish.

**Figure 10.**
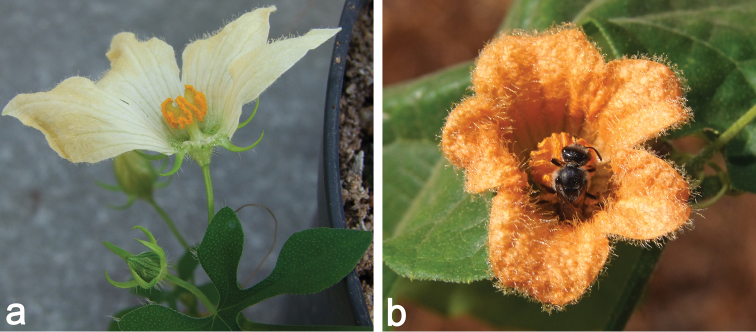
**a** Male plant of *Coccinia rehmannii* [aff. var. littoralis] (*N. Holstein 126*). The plant was raised from seeds of a female plant with ovoid fruits (B. Jarret – pers. comm.) **b** Male flower of Coccinia adoensis var. aurantiaca (*N. Holstein et al. 85*). The halictid bee (H. Schaefer – pers. comm.) circled around the globose anther head harvesting pollen. The scent of the flower was strong and honey melon-like.

The calyx differentiates as a bulge (Fig. 7a, b) with usually five lobes, or only the lobes emerge from the perianth tube (Fig. 9). If the calyx emerges as a bulge, then it and the perianth tube are rather conspicuously differentiated from the corolla in terms of color. If only the calyx lobes emerge, then the color of perianth tube fades to green color towards the receptacle, with the veins of the corolla remaining more intensely colored. Whether calyx and corolla are non-differentiated (congenital fusion) or postgenitally fused, is not known for *Coccinia*, but in the distantly related *Echinopepon wrightii* (A.Gray) S.Watson the perianth tube is non-differentiated (Leins and Galle 1971). The outside of the perianth tube can bear long trichomes of the type on as the lower leaf surface or the petioles (Fig. 10a). The calyx lobes are acute triangular to subulate or linear, rarely slightly lanceolate. The orientation of the calyx lobes is erect, spreading, or reflexed, although they can be curved inwards (e.g., Coccinia rehmannii aff. var. littoralis (Fig. 10a) or outwards (e.g., *Coccinia intermedia*). The color of the calyx lobes can be more intense (green) than the perianth tube or the pedicel (Fig. 7b). In *Coccinia grandis*, the tip of the calyx lobes is brownish to reddish just as the teeth on the leaves and the corolla (Fig. 7a).

**Figure 11.**
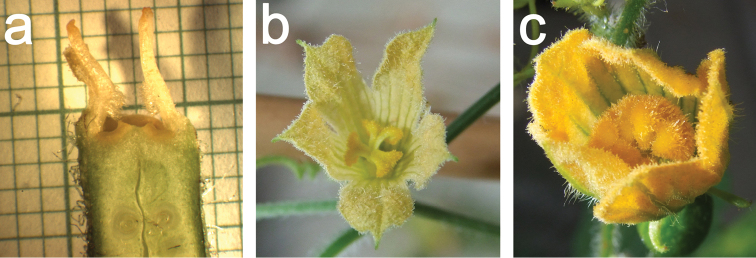
**a** Longitudinal section of a female *Coccinia hirtella* flower (size of the small squares is 1 mm²). Perianth and style were detached. The pollen sacs on the staminodes are highly reduced. The introrse side of the staminodes bears long trichomes that in intact flowers touched the style **b** Female flower of Coccinia rehmannii var. rehmannii with bilobate stigma arms **c** Female flower of *Coccinia megarrhiza* with bulging stigma arms.

The petals of *Coccinia* species are fused at the base, usually for at least one third of the total length. Rarely, the petals are free down to the height of the calyx lobes (pers. observ. in *Coccinia megarrhiza*, Coccinia rehmannii var. rehmannii, and *Coccinia sessilifolia*). Perianth tube and corolla tube are often campanulate, rarely funnel-shaped or tubular. The perianth tube can be urceolate in *Coccinia longicarpa*, *Coccinia racemiflora* and sometimes in *Coccinia barteri*. The tips of the (4–)5(–7) corolla lobes are rounded to acute with an apical tooth. The apical tooth can be inconspicuous or colored claret red or brown, such as in Coccinia adoensis var. aurantica or *Coccinia grandis*. Outside, the perianth tube and the corolla is glabrous or covered with short (< 10 globose cells in *Coccinia grandis*) trichomes. Inside, the corolla is covered with long trichomes (up to 20 cells in *Coccinia grandis*), sometimes with a glandular apical cell (Fig. 10b). The trichomes become shorter towards the receptacle. The inner side of the hypanthium of female flowers is glabrous and smooth, which suggests nectary tissue, in *Coccinia grandiflora*, *Coccinia grandis*, and *Coccinia hirtella* (Fig. 11a). The size of the corolla does not differ conspicuously between staminate and pistillate flowers; pistillate flowers might be a bit smaller.

### Androecium

In staminate flowers, the three stamens originate from the base of the perianth tube, and the filaments are fused to a central column (Fig. 10a). The fusion point sometimes leaves a small gap to the hollow receptacle center. This gap, however, can be filled with long multi-cellular trichomes (e.g., in *Coccinia abyssinica* and *Coccinia megarrhiza*). Rarely, the filaments can also be separate. The filaments are glabrous and white, greenish, yellowish, or orange. The number of vascular bundles in the stamens is disputed. Chakravarty (1954) reports five vascular bundles in *Coccinia grandis*: two stamens have two bundles each, and the third stamen has a single bundle. Later research shows three vascular bundles for the same species with one per stamen (Bhuskute et al. 1986; Deshpande et al. 1986). In *Coccinia hirtella* each stamen contains a single vascular bundle (pers. observ.).

The anthers together form a globose head (Fig. 10b). Each anther is bithecate; sometimes one can be monothecate (Bhuskute et al. 1986; Chakravarty 1954). Each theca is sinuate. Deshpande et al. (1986) report a bi-layered fibrous endothecium and a secretory tapetum, which they found differs from distantly related *Momordica charantia* L.

In pistillate flowers, the three, now free, stamens are reduced to staminodes that originate from the interior perianth wall, forming a hypanthium. Introrsely, the staminodes of *Coccinia grandiflora*, *Coccinia grandis* and *Coccinia hirtella* bear long, multicellular trichomes, except for the apex, extrorsely the staminodes are glabrous (Fig. 11a). The anthers of the staminodes are strongly reduced to a slightly yellowish spot at the apex in *Coccinia grandis* and *Coccinia hirtella*. The staminodes of *Coccinia megarrhiza* bear long multicellular trichomes introrsely and laterally but are glabrous extrorsely.

### Pollen

Pollen in *Coccinia* species shows little diversity. The pollen is oblate-spheroidal to prolate with a reticulate exine (Table 1). Additionally, the pollen of *Coccinia pwaniensis* (Holstein and Renner 2010), *Coccinia hirtella*, and *Coccinia trilobata* is prolate, the exine texture is unknown. The sampling of the examined species covers all clades and suggests uniformity in shape and exine texture, which negates systematic value of pollen in *Coccinia*. The color is yellow in *Coccinia abyssinica*, *Coccinia grandiflora* (Zimmermann 1922b; and pers. observ.), *Coccinia grandis*, *Coccinia hirtella*, *Coccinia megarrhiza*, *Coccinia microphylla*, *Coccinia rehmannii*, *Coccinia sessilifolia*, and *Coccinia trilobata*, and orange in Coccinia adoensis var. aurantiaca. Zimmermann (1922b) reports that pollenkitt of *Coccinia grandiflora* contains a yellow colorant that is soluble in peanut oil, but not in water and only slightly in heated chloral hydrate solution. It changes its color in concentrated sulfuric acid to blue, in Lugol’s iodine (I_2_KI) to green, and in osmic acid to brown. As in several other cucurbit species, *in vitro* germination of *Coccinia grandis* pollen increases from pH = 7 towards alkalinity and is maximal at pH = 8.5 (Zaman 2009).

**Table 1.** Pollen characters in *Coccinia* species.

	shape	Size (P × E) [µm]	Exine texture	source
*Coccinia abyssinica*	Prolate-spheroidal	60–70 × 56–65	Reticulate	Marticorena 1963
	Oblate-spheroidal to spheroidal	76 × 81	Reticulate	Khunwasi 1998
*Coccinia adoensis*	Prolate	66–73 × 45–50	Reticulate	Marticorena 1963
	Prolate-spheroidal	72 × 61	Reticulate	Khunwasi 1998
*Coccinia barteri*	Prolate	70–80 × 50–60	Reticulate	Marticorena 1963
	Prolate-spheroidal	71 × 58	Reticulate	Khunwasi 1998
*Coccinia grandiflora*	Prolate			Zimmermann 1922b
*Coccinia grandis*	Prolate	60–63 × 34–40	Reticulate	Marticorena 1963
	Prolate-spheroidal	58 × 52	Reticulate	Khunwasi 1998
	Prolate	47.61–64.62 × 35.91–44.80	Coarsely reticulate	Perveen and Qaiser 2008
	Subprolate to prolate	34–52 × 28–35	Reticulate	Awasthi 1962
	Prolate	41.20 ± 0.61 × 34.00 ± 0.45	Reticulate	Datta 1988
*Coccinia megarrhiza*	Oblate-spheroidal to spheroidal	92 × 92	Reticulate	Khunwasi 1998
*Coccinia mildbraedii*	Prolate	55–60 × 35–41	Reticulate	Marticorena 1963
Coccinia sessilifolia var. sessilifolia	Prolate-spheroidal to prolate	70 × 58	Reticulate	Khunwasi 1998

### Gynoecium

Pistillate flowers are epigynous and have three (rarely two or four) carpels. The ovary is narrowly spindle-shaped, oblong to globose. The surface is smooth or warty; it is glabrous or has the indumentum of the pedicel. The style is often greenish-white or pale-yellowish; the stigmas are frequently in yellowish colors and covered with long trichomes (Fig. 11c). Each stigma in *Coccinia grandiflora*, *Coccinia grandis*, *Coccinia hirtella*, *Coccinia megarrhiza*, *Coccinia rehmannii*, and *Coccinia sessilifolia* is U-shaped with the ends of the lower sides of the arms touching each other. The stigmatic branches can be long and free, such as in *Coccinia grandiflora*, *Coccinia grandis* and Coccinia rehmannii var. rehmannii (Fig. 11b), or short and bulbous, such as in *Coccinia hirtella*, *Coccinia megarrhiza* (Fig. 11c), *Coccinia microphylla*, and *Coccinia sessilifolia*.

The placentation of the ovules in *Coccinia* is involute, which is also discussed for other Cucurbitaceae by Leins and Galle (1971). The funicle appears to be attached to the outer wall, but actually attaches to a septum coming from the axis (Fig. 11a, b, c), which itself is connected to the outer wall. The axis-wall septum, however, might be reduced during ripening, but this needs further study.

**Figure 12.**
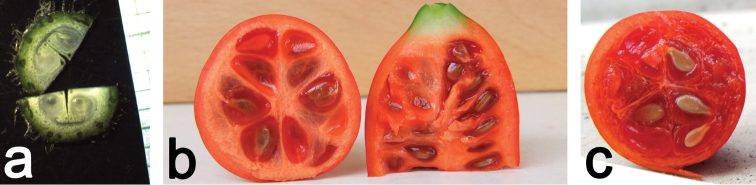
**a** Cross-section through an ovary of *Coccinia hirtella*. The ovules are anatropous with the micropyle facing outwards **b** Cross- and longitudinal section of a *Coccinia megarrhiza* fruit. The seeds are enclosed in a hyaline hull (aril) and seemingly attached to the periphery **c** Cross-section through a fruit of *Coccinia sessilifolia*. Note that the vascular bundles in the lower left of the picture bend in the periphery, so the placentation is not parietal but involute.

The anatomy of the ovules is barely surveyed in *Coccinia*. In *Coccinia abyssinica* and *Coccinia grandis*, the ovules are reported to be anatropous, bitegmic, and crassinucellate (Getahun 1973; Zahur 1962). Anatropus ovules also occur in *Coccinia hirtella* (Fig. 12a). The position of the ovules is horizontal in *Coccinia grandis* (Kirkwood 1904), *Coccinia hirtella* (Fig. 12a), and *Coccinia megarrhiza* (Fig. 12b) with the micropyle facing outwards. Horizontal ovules are regularly reported in the Benincaseae and the Cucurbiteae (Schaefer and Renner 2011a).

In staminate flowers, a pistil is not developed because the stamens fuse to a central column. The pedicel is narrow and reaches the perianth, and there is no indication of even a thin (sterile) inferior ovary in the flower.

### Female gametophyte development and embryology

The development of the embryo has only been investigated in *Coccinia grandis*. Chakravorti (1947) and Zahur (1962) report that the female gametophyte development is according to the *Polygonum*-type. Both observe that the synergides possess hooks instead of the filiform apparatus. Chakravorti (1947) describes the developing endosperm as a nuclear type, which is confirmed by Chopra (1955). By formation of a large central vacuole, the nuclei become displaced to the periphery. After the endosperm becomes cellular, the often lateral chalazal haustorium remains coenocytic. Then, the haustorium becomes cellular with multinuclear cells except for the apex (Chopra 1955).

### Fruits

The fruits are many-seeded berries, which vary in size and shape between species (and within *Coccinia grandis*, *Coccinia rehmannii*, and *Coccinia subsessiliflora*). The smallest fruits occur in Coccinia rehmannii var. rehmannii and *Coccinia microphylla* with globose berries as small as 1 cm in diameter at maturity. However, in both species larger globose fruits (up to 2.5 cm in diam.) and in *Coccinia rehmannii* ovoid fruits may occur additionally, in the latter case especially in more humid habitats. The largest fruits occur in *Coccinia samburuensis* and the rainforest species *Coccinia grandiflora*, *Coccinia longicarpa*, *Coccinia mildbraedii*, and *Coccinia schliebenii*, which have long elliptical to cylindrical (sausage-shaped) fruits up to 20 cm long and up to 5 cm in diameter.

**Figure 13.**
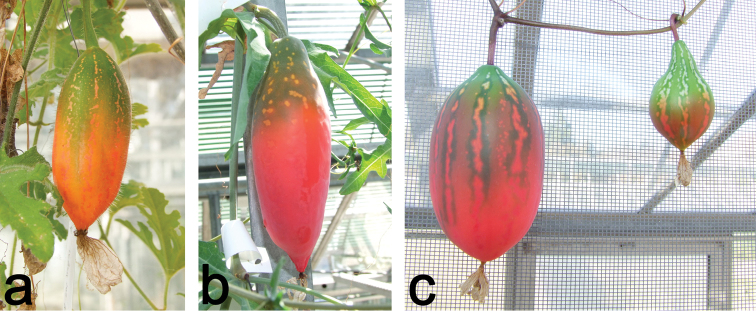
**a** Ripening fruit of *Coccinia hirtella*. Note the typical lobulate leaves of this species in the lower right **b** Ripening fruit of *Coccinia sessilifolia*. The fruit, like the plant, bears a waxy bloom **c** Ripening fruits of *Coccinia megarrhiza* have a dark green halo around the white longitudinal mottling. The left fruit is derived from pollination with *Coccinia megarrhiza* pollen, whereas the smaller fruit on the right is derived from cross-pollination with *Coccinia trilobata* (both pollinations were conducted on the same day).

Immature fruits often have a white or pale-green (*Coccinia hirtella*, *Coccinia sessilifolia*) or dark green (*Coccinia adoensis*) longitudinal mottling or lines, even when the ovary and the ripe fruit is single-colored (Figs 2a, 13a, b, c). In the *Coccinia rehmannii* clade, the white spots or lines become surrounded by a dark green halo during ripening (Fig. 13c). Rarely, if no white mottling develops, e.g., in some *Coccinia microphylla*, dark green spots develop nevertheless. In any case, the mature fruit in species of the *Coccinia rehmannii* clade is usually uniformly colored red (Zimmermann 1922b; and pers. observ.). Ripening usually occurs from green with or without mottling via yellow to orange to the final coloration. The color changes from the apex of the fruit downwards (Fig. 13a, b, c), independent of the position (hanging vs. horizontal) in *Coccinia sessilifolia*. In *Coccinia megarrhiza*, pendulous fruits ripen from the apex to the base, which sometimes remains green even when the apex already turns soft. In lying fruits from creeping *Coccinia megarrhiza* plants, ripening does not proceed from the apex, but starts from point that is closest to the source of either warmth or light (pers. observ. from greenhouse cultivation). The degree of the yellow to orange ripening zone varies. In *Coccinia sessilifolia*, fruits directly turn red, whereas in *Coccinia grandis* the color change includes a well visible yellow zone. Unripe fruits collected of *Coccinia grandis* tend to turn yellow outside and pink to red inside (Imbumi 2004). Mature fruits are in deep red colors (hence the genus name) or orange-red. Rarely, a white longitudinal mottling is described in ripe fruits (e.g., *Coccinia mackenii*).

Immature fruits are glabrous or have the same indumentum as the ovary. By ripening, the indumentum is usually reduced. The exocarp of *Coccinia* fruits is papery thin and has a waxy bloom when ripe. The endo- and mesocarp are red, fleshy and soft (Fig. 12b, c). The pulp is nerved with a dense network of tubular tissue. Shah et al. (1983) report that such a network consists of sieve tubes in *Coccinia grandis*. The sieve tubes are not connected to the main vascular strands and are filled with a proteinaceous material. The authors suggest that the sieve tube network aids nutrient transport during the rapid growth of the fruit.

### Seeds

**Figure 14.**
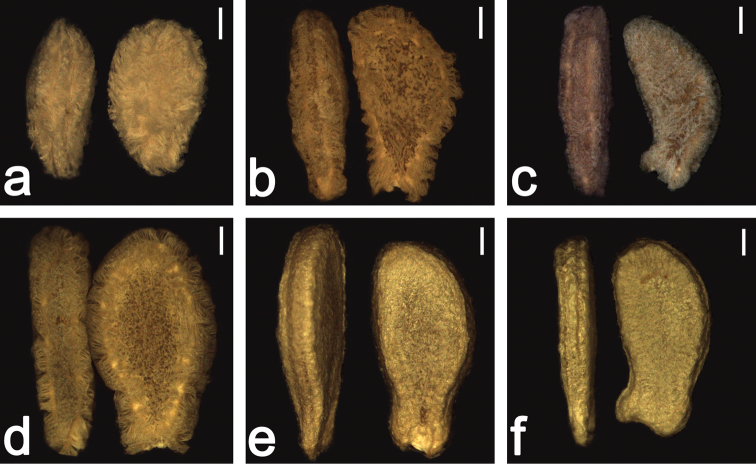
Seeds of *Coccinia*. The lack of fibers in e and f are preparation artifacts due to mechanical removal of the hyaline aril. Maceration (coarse crushing of the fruit and soaking of the mass in water for 2–3 weeks; R. Brüggemann – pers. comm.) retains the surface fibers. Length of white bars equals 1 mm. **a** Seeds of Coccinia adoensis var. jeffreyana (plant derived from seed of the same fruit: *N. Holstein 130*). Note the lenticular face and symmetrical shape of the seed **b** seeds of *Coccinia abyssinica* (plant derived from seed of the same fruit: *N. Holstein 120* and *132*) **c** Seeds of *Coccinia trilobata* **d** Seeds of *Coccinia sessilifolia* (harvested by maceration) **e** Seeds of *Coccinia sessilifolia* (harvested by mechanical extraction; taken from *N. Holstein 119*) **f** Seeds of *Coccinia grandis* (harvested by mechanical extraction).

The seeds (Fig. 14) in *Coccinia* species are beige to grayish with a small margin, which often has a darker coloration. The shape is more (esp. in the *Coccinia rehmannii* clade) or less asymmetrically ovate (especially *Coccinia adoensis*, *Coccinia pwaniensis*, and *Coccinia senensis*). The surface is flat, esp. in the *Coccinia rehmannii* clade, to lenticular (esp. *Coccinia adoensis*, *Coccinia pwaniensis*, and *Coccinia senensis*). The size varies from 4.5–7 × 3–5 × 1–2 mm (L/W/H). Seed numbers per fruit vary drastically from about 10 (*Coccinia microphylla*) to c. 100 (+ c. 20 infertile) in *Coccinia sessilifolia* (*N. Holstein 119*). Species with larger fruits might contain more seeds.

Detailed observations of the seed anatomy have been made by Getahun (1973) for *Coccinia abyssinica* and by Chakravorti (1947) for *Coccinia grandis*. Getahun describes the mature seed as consisting of the embryo, a membrane-like structure (pellicle) closely adhering the embryo but separated from the hard testa. However, Chakravorti does not recognize a pellicle in *Coccinia grandis*. Both authors agree that the inner integument disappears and the testa develops solely from the outer integument. The testa of *Coccinia abyssinica* is described as comprising four layers (from center outwards): (1) a thin-walled parenchyma, (2) a sclerenchyma of macrosclereids, (3) a thick-walled parenchyma, and (4) an epidermal layer. The outermost layer, the epidermis, is disintegrated, leaving the cell walls as slender rods of 500 µm length. This has also been noticed in other species, and the surface has been described as a fibrillose testa (Jeffrey 1967; Kéraudren 1967). De Wilde et al. (2011) also interpret the seed surface of *Coccinia grandis* as having a disintegrated, pulpy, radiately striate exotesta.

Getahun contrasts his observations with those of Chakravorti (1947) in *Coccinia grandis*, but the seeds are in fact similar, just incompletely described by Chakravorti. Chakravorti draws a four-layered testa, but does not name the innermost layer that has the same hatching as the third layer, which he describes as “cells with thickened walls”. This is what Getahun calls parenchyma. Chakravorti’s outermost layer, the epidermis, consists of radially elongated cells with thin walls. These cells have likely just not yet disintegrated as observed by Getahun. The only difference between both observations is the second layer, which consists of macrosclereids in *Coccinia abyssinica* and of radially slightly elongated cells with thin walls in *Coccinia grandis*. These different observations are explainable by two possibilities: 1) different developmental stages of the seeds, since Chakravorti surveys the seed development, so the layers are immature, while Getahun surveys mature seeds and germination, or 2) different staining. Chakravorti uses haematoxylin alone, which does not stain lignified cell walls, whereas Getahun uses haematoxylin with safranin as counter stain, which makes lignin, and thus sclerenchyma, well visible (von Aufseß 1973).

The seeds in *Coccinia*, at least in *Coccinia abyssinica* (Hora 1995), *Coccinia grandis*, *Coccinia hirtella*, *Coccinia megarrhiza*, *Coccinia microphylla*, *Coccinia mildbraedii*, *Coccinia sessilifolia*, and *Coccinia subsessiliflora* (pers. observ.) are surrounded by a hyaline red juicy envelope (Fig. 12b). As the ovule is bitegmic (see above), one might assume the hyaline envelope is the testa. Chakravorti (1947), however, observes that the juicy envelope is derived from carpellary tissue. However, Getahun (1973) does not recognize the hyaline hull in *Coccinia abyssinica*, which is surprising as it also occurs in the closely related species *Coccinia megarrhiza* and *Coccinia microphylla*. Similar structures to the hyaline hull are also found in other Cucurbitaceae, esp. in *Momordica*. Van der Pijl (1982) interprets these as “endocarp-pulpa” taking over the function of an aril for seed dispersal as the fruits of *Momordica* species dehisce at maturity. However, *Coccinia* fruits disintegrate and do not dehisce, e.g., into valves.

### Germination

The seeds of *Coccinia abyssinica* maintain a high germination rate (100%) after four years of storage at room temperature (Getahun 1973). However, time from watering until germinating increases from 4 days (after one year of storage) to 16 days (after four years of storage). Seeds of *Coccinia grandis* are also able to germinate after four years of storage, while seeds of *Coccinia ogadensis* Thulin (3 seeds tested) did not germinate after five years (pers. observ.). Getahun (1973) reports that *Coccinia abyssinica* seeds do not germinate below 10 °C and above 35 °C. In the latter case, he observes thermal damage to hypocotyls and primary roots. The optimum for germination in *Coccinia abyssinica* is between 20 and 30 °C and that of *Coccinia grandis* is 35 °C, whereas temperatures < 23.5 °C and > 40 °C inhibit germination (Li et al. 2001). The germination rate of *Coccinia abyssinica* seeds in the light is decreased by 35% compared to germination in darkness (Getahun 1973). In *Coccinia sessilifolia*, seed viability declines after 9 months, and germination is at a maximum after 10–20 min smoke exposure or red:far red light treatment, followed by burying and a long-day cycle (Weiersbye and Witkowski 2003). Rotting of a crushed ripe fruit in water (for seed extraction) resulted in germination of two seeds in an artificial hybrid (*Coccinia megarrhiza* ♀× Coccinia rehmannii aff. var. littoralis ♂) after 3 weeks of soaking (pers. observ.). *Coccinia grandis* seeds do not exhibit dormancy (Motooka et al. 2003); for the other species there is no information available.

## Genome, chromosomes, and hybridization

### Chromosomes and sex determination

**Figure 15.**
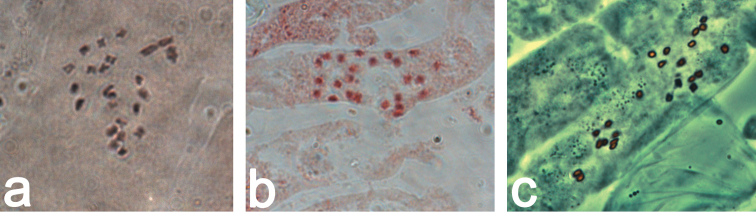
Chromosome preparations fixed in 3:1 EtOH – acetic acid and stained with orcein, objective lens: 100×. **a** Mitotic plate of a male *Coccinia grandis* (2*n* = 22 + XY) **b** Mitotic plate of a male plant of *Coccinia hirtella* (2*n* = 24) **c** Phase contrast image of a mitotic plate of a male plant of *Coccinia sessilifolia* (2*n* = 24).

*Coccinia* is one of the few examples in the plant kingdom, in which at least one species has heteromorphic sex chromosomes (Ming et al. 2011). *Coccinia grandis* contains 22 autosomes plus 2 gonosomes. Female individuals have homomorphic XX, whereas male individuals have heteromorphic XY chromosomes (Fig. 15a). Although Kumar and Deodikar (1940) report males to have two large “X” and females a large X and a smaller Y chromosome, later studies (Chakravorti 1948; Kumar and Vishveshwaraiah 1952) reveal that males are heteromorphic and the Y is 2.5 (Bhaduri and Bose 1947) to 3–4 times longer (Guha et al. 2004) than the other chromosomes. Some years before Kumar and Deodikar, Sutaria (1936) reported *n* = 12 from pollen mother cells of *Coccinia grandis*, without finding the large Y chromosome. Although scientists from India conducted some research for *Coccinia grandis*, chromosome work in other *Coccinia* species is almost none-existent. McKay (1930) reports *n* = 12 for *Coccinia hirtella*, without mentioning whether he studied a male or a female individual. The author’s own chromosome counts (Table 2; Fig. 15b) support McKay’s report and reject the existence of heteromorphic sex chromosome in *Coccinia hirtella* males. This is also the case for *Coccinia sessilifolia* (Fig. 15c). Two counts in the *Coccinia rehmannii* clade reveal a reduction of chromosome number and the non-existence of a heteromorphic Y chromosome there.

**Table 2.** Chromosome numbers of Coccinia species and sexes of surveyed individuals.

Species	Sex of individual	Chromosomes (2*n*)	Voucher
*Coccinia grandiflora*	female	24	*N. Holstein 114* (EA, M)
*Coccinia grandis*	male	22 + XY (Fig. 15a)	*N. Holstein 32* (M)
*Coccinia grandis*	female	22 + XX	*N. Holstein 25* (M)
*Coccinia hirtella*	male	24 (Fig. 15b)	*N. Holstein 29* (M)
Coccinia rehmannii aff. var. littoralis	male	20	*N. Holstein 126* (M)
*Coccinia sessilifolia*	male	24 (Fig. 15c)	*N. Holstein 13* (M), *N. Holstein 109* (M (3))
*Coccinia sessilifolia*	female	24	*N. Holstein 119* (B, M)
*Coccinia trilobata*	male	20 (A. Sousa, pers. comm.)	*N. Holstein & P. Sebastian 9* (M)

Due to the sex chromosomes, sex expression in *Coccinia grandis* plants is pre-determined and sex ratios in the offspring basically follow Mendelian inheritance of a single allele. However, Agarwal and Roy (1983) report that of 500 planted seeds only 181 (36.2%) were male, and their interpretation is that there might be a genetic mechanism to reduce the number of male plants. As they do not report XY females, their finding might be explained rather by an increased lethality of XY embryos due to deleterious mutations on the single X or on the Y in Y-containing pollen. The Y chromosome in *Coccinia grandis* is dominant, as the presence of a single Y results in male phenotypes, regardless of the number of X chromosomes (Agarwal and Roy 1975; Kumar and Vishveshwaraiah 1952; Roy and Roy 1971b). Triploids of *Coccinia grandis* with a 3*n* = XYY constitution are also male but bear flowers in clusters instead of the usually solitary ones and exhibit leaf deformations (Roy and Roy 1971b).

Evidence for Mendelian inheritance of sex in *Coccinia hirtella* is not so clear, as the same plant can produce flowers of the opposite sex in succeeding seasons. Two plants marked as female and one as male from observation of flowers produced flowers of the opposite sex in the following year (pers. observ.), making *Coccinia hirtella* functionally dioecious, but genetically hermaphroditic. On the other hand, there are several observations of flowers of the opposite sex in otherwise unisexual plants. Kumar and Vishveshwaraiah (1952) report a gynodioecious form of *Coccinia grandis* that has homomorphic chromosomes (XX). Although bisexual flowers are reported to develop, pollen grain development is arrested, and the male function remains suppressed. Roy and Saran (1990), however, report fully fertile hermaphroditic flowers in an otherwise female individual. Holstein and Renner (2011a) report a collection of *Coccinia intermedia* that bears male floral buds and female flowers and young fruits on the same nodes. This observation can be interpreted as ‘leaky dioecy’ (Baker and Cox 1984). Among the author’s own cultivated plants, a male individual of *Coccinia megarrhiza* produced a single female flower towards the end of the season (Fig. 9). Although two male flowers on the same individual were open at the same time when the female flower was mature, the pollen sacs did not open. It is not known whether this was a coincidence or functionally significant, e.g., to prevent selfing. Selfing is often discussed as being advantageous in small population sizes, e.g., when new islands are colonized. Prevention of simultaneous flowering of both sexes on the same plant implies that leaky dioecy would not immediately aid the establishment of new distant populations per se. It might require the establishment of several plants or clonal separation. In any case, the single female flower was receptive and was fertilized by another male *Coccinia megarrhiza* plant derived from the same fruit as the “female” plant. The resulting fruit and seeds developed normally.

The production of hermaphroditic flowers in X-radiation studies (Agarwal and Roy 1983; Roy 1974) shows that dicliny in *Coccinia* is kept up actively. Agarwal and Roy found hermaphroditic flowers on two plants with otherwise female flowers and an XX configuration. They also report the development of a normal fruit without mentioning the fertility of pollen from hermaphroditic flowers, but interpret their finding as cleistogamy. However, fruit development without previous pollination (parthenogenesis) or from pseudogamy with pollen from different genera as fructification stimulus is described by Lal (1973). True selfing from own pollen (in irradiated XX individuals) would mean that the Y chromosome is not important for fertile pollen development. Furthermore, this means that it only carries at least one gene for suppression of the development of the female organs, and the occurrence of a second X suppresses pollen development in “normal” plants. Agarwal and Roy (1983) also report that X-ray dosages of 5 to 50 R [1.29 × 10^-3^ to 12.9 × 10^-3^ C/kg] result in a drastic reduction of the sex ratio (11 males, 2 hermaphrodites, and 127 females out of 500 irradiated seeds). This might indicate that the single X bears many functionally important genes in contrast to the Y, as mutations in the single X lead to an increased mortality compared to females with a balancing second X chromosome.

### Genome of *Coccinia grandis*

Aside from research on the sex chromosomes, a few studies on the genome of *Coccinia grandis* have been undertaken. Guha et al. (2004) report the 4C nuclear DNA content of female *Coccinia grandis* as 8.37 ± 0.14 pg, whereas that of male *Coccinia grandis* is 10.17 ± 0.24 pg. This means that the difference between X and Y chromosome adds about 20% to the complete DNA content. Patankar et al. (1985) report a DNA content of 1C = 2.75 pg for *Coccinia grandis*, however, they do not report the sex of the analyzed individual. Interphase nuclear structure in *Coccinia grandis* is chromocentric with 14 ± 0.25 chromocenters (Patankar et al. 1985).

Surveys on the reassociation kinetics in Cucurbitaceae suggest that *Coccinia grandis* has the lowest amount of repetitive DNA among the six species studied in the Cucurbitaceae (Bhave et al. 1984). Fragments of 550 bp length have 25% of repetitive elements (Bhave et al. 1984), whereas 7400 bp long fragments consist of 49% repetitive DNA (Bhave et al. 1986). However, Pasha and Sen (1995) report different results as they find 400–600 bp long fragments to comprise 38% highly repetitive DNA (52% total repetitive DNA), which appears to be average in the Cucurbitaceae. Pasha and Sen do not discuss this difference, which cannot be explained by difference in sex, as the large amounts of plant material used (1 kg seeds or 100 seeds respectively) suggest that it must have comprised elements of both sexes. A sex discriminating study of *Coccinia grandis* reassociation kinetics has not been undertaken yet.

### Hybridization and crossing experiments

Charles Naudin’s famous work on the effects of hybridization included crosses between *Coccinia* plants. He reports successful crosses between Asian *Coccinia indica* (nom. illeg. for *Coccinia grandis*) and the NE African *Coccinia schimperi* (P06809214, P06809215, P06809216; Naudin 1862), which is now seen as a synonym of *Coccinia grandis*. Naudin’s *Coccinia schimperi*, however, has buff petals, whereas the Asian *Coccinia grandis* has snow-white petals. Both supposed species hybridized without problems. During the following two years, Naudin could not intercross within the F1 generation because plants of different sexes did not flower at the same time, so he crossed F1 individuals with a female *Coccinia grandis* from Asia, which again produced offspring. As Naudin erroneously supposed that he dealt with two species, he deduced that hybrids between species could be fully fertile, have a reduced fertility or be sterile, and that there was no clear boundary between species and varieties. However, he proved rather that the buff-petaled, African *Coccinia schimperi* and the white-petaled Asian *Coccinia grandis* are a single species obeying the biological species concept.

Naudin also crossed other *Coccinia* species that he had in cultivation. *Coccinia quinqueloba* and *Coccinia mackenii*, although sometimes not easily distinguishable, were not amenable to crossing (Naudin 1866). Only 1 out of 20 crossing trials resulted in a fruit that developed poorly. Naudin did not publish whether the hybrid seeds were fertile or even viable, but his observations are valuable as each one accession of *Coccinia quinqueloba* and *Coccinia mackenii* were not distinguishable using more than 3500 bp of plastid sequences, and hence might share the same plastid haplotype (Holstein and Renner 2011b). There are collections that share characters of *Coccinia mackenii* and *Coccinia quinqueloba*, but these are not intermediates. In these collections, long petioles (typical for *Coccinia mackenii*) are coupled with simple tendrils (typical for *Coccinia quinqueloba*), and thus cannot be unambiguously allocated to either species. However, if both typical forms are indeed reproductively isolated, then they are species sensu Mayr (1942), and the crossing behavior of these species needs to be tested reciprocally to define the morphological scope of the two species.

Naudin also crossed male *Coccinia diversifolia* (*Coccinia abyssinica*) with a female *Coccinia mackenii*, which are rather distantly related and do not co-occur in nature. However, the cross resulted in onset of mediocre fruits with only few, but well-developed and viable seeds (Naudin 1866). Naudin did not report further results for this cross either.

As reproductive isolation between species is often assumed but rarely tested, crossing experiments among species that are cultivated in Munich Botanical Garden have been performed. Positive results are given in Table 3.

**Table 3.** Description of the F1 from crosses and a natural hybrid between *Coccinia* species. Species used for the artificial crosses are not sympatric.

Parent species	Offspring	Occurrence
*Coccinia grandis* ♀ × *Coccinia hirtella* ♂	F1 vegetatively morphologically intermediate; flowers are either aborted or sterile (pollen sacs remain closed); pollen globose; corolla is smaller than in each parent species (Fig. 16a)	Artificially in Munich Botanical Garden; voucher: *N. Holstein 108* (M)
*Coccinia hirtella* ♀ × *Coccinia grandis* ♂	F1 vegetatively morphologically intermediate; flowers smaller (Fig. 16b), sterile	Artificially in Munich Botanical Garden; voucher: *N. Holstein 116* (M)
*Coccinia grandis* ♀ × *Coccinia pwaniensis* ♂	F1 morphologically intermediate; flowers sterile (pollen sacs remain closed) (Fig. 16c)	Naturally in Pugu Hills, Dar es Salaam, Tanzania; vouchers: *N. Holstein et al. 102* (DSM, M), *103* (M), *104* (M), *105* (DSM, M)
*Coccinia grandis* ♀ × *Coccinia sessilifolia* ♂	F1 vegetatively morphologically intermediate; flowers are either aborted or sterile (pollen sacs remain closed); corolla is smaller than in each parent species (Fig. 16d)	Artificially in Munich Botanical Garden; voucher: *N. Holstein 113* (B, M); *N. Holstein 115* (M)
*Coccinia hirtella* ♀ × *Coccinia trilobata* ♂	F1 morphologically intermediate; males flowering vigorously with intermediate flowers, pollen sacs open, but pollen is sterile	Artificially in Munich Botanical Garden; *N. Holstein 121* (M)

**Figure 16.**
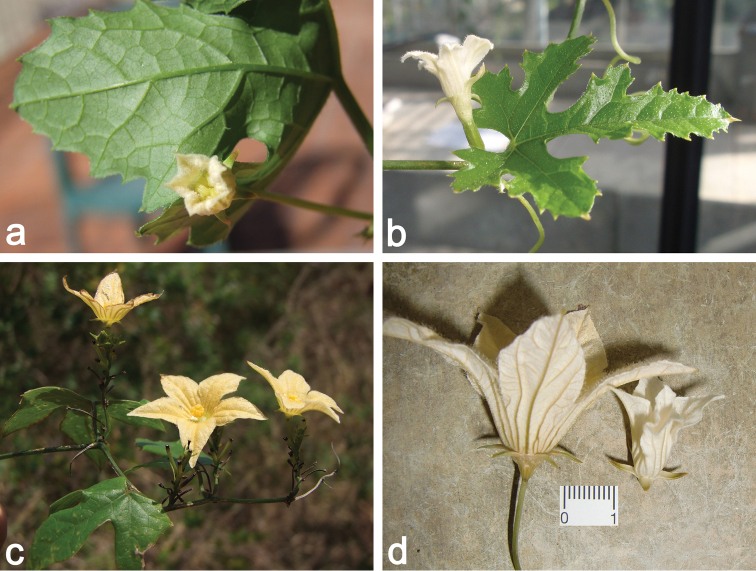
Hybrids of *Coccinia* species. **a** *Coccinia grandis* ♀ × *Coccinia hirtella* ♂ **b** *Coccinia hirtella* ♀ × *Coccinia grandis* ♂ **c** *Coccinia grandis* ♀ × *Coccinia pwaniensis* ♂; note that the pollen sacs are not open although the flower is in full bloom indicating sterility **d** Left flower: male *Coccinia sessilifolia*, right flower: male flower of *Coccinia grandis* ♀ × *Coccinia sessilifolia* ♂ (*Coccinia grandis* flowers are about equally sized as *Coccinia sessilifolia*).

Interspecific fertilization succeeded or failed without correlation of relatedness or co-occurrence (Table 4). Female flowers of *Coccinia sessilifolia* could not be fertilized with pollen of *Coccinia megarrhiza* (4 trials), *Coccinia trilobata* (4 trials), *Coccinia rehmannii* (2 trials), *Coccinia hirtella* (3 trials) or *Diplocyclos palmatus* (2 trials). Pollinated female flowers were discarded like non-fertilized flowers. Hence, hybridization seems to be prevented prezygotically in female *Coccinia sessilifolia* with members of the *Coccinia rehmannii* clade and *Coccinia hirtella* as pollen donor. As *Coccinia sessilifolia* and *Coccinia rehmannii* co-occur widely in their range and share flowering time and floral syndrome, the production of hybrids would reduce fitness drastically. *Coccinia sessilifolia* and *Coccinia hirtella* do not co-occur, but belong to the same clade (see chapter Evolution and phylogeny). Although a female *Coccinia sessilifolia* could not be fertilized by pollen from *Coccinia hirtella*, pollination of a female *Coccinia hirtella* with pollen from *Coccinia sessilifolia* resulted in fruit onset.

**Table 4.** Observations on fruit development of crosses between *Coccinia* species (except for crosses mentioned in Table 3). Viability and morphology of the F1 is not known so far. ^a^ = sympatrically distributed species, ^b^ = close relatives/sister species, ^c^ = species occur in the same area but in different habitats.

Parent species	crossability
*Coccinia grandis* ♀ × *Coccinia abyssinica* ♂	Onset of fruit (1 trial) **^c^**
*Coccinia grandis* ♀ × *Coccinia megarrhiza* ♂	Onset of fruit (1 trial) ^a^
*Coccinia grandis* ♀ × Coccinia rehmannii aff. var. littoralis ♂	Abortion of flower (1 trial)
*Coccinia grandis* ♀ × *Coccinia trilobata* ♂	Abortion of flower (2 trials) **^c^**
*Coccinia hirtella* ♀ × Coccinia rehmannii aff. var. littoralis ♂	Onset of fruit (1 trial) **^c^**
*Coccinia hirtella* ♀ × *Coccinia sessilifolia* ♂	Onset of fruit (1 trial)
*Coccinia megarrhiza* ♀ × *Coccinia abyssinica* ♂	Onset of fruit (1 trial) **^b^**^ & ^**^c^**
*Coccinia megarrhiza* ♀ × *Coccinia hirtella* ♂	Abortion of flower (1 trial)
*Coccinia megarrhiza* ♀ × Coccinia rehmannii aff. var. littoralis ♂	Onset of fruit (1 trial)
*Coccinia megarrhiza* ♀ × *Coccinia sessilifolia* ♂	Abortion of flower (2 trials)
*Coccinia megarrhiza* ♀ × *Coccinia trilobata* ♂	Onset of fruit (Fig. 13c; 1 trial)
*Coccinia microphylla* ♀ × *Coccinia megarrhiza* ♂	Abortion of flower (1 trial) ^a^
*Coccinia microphylla* ♀ × *Coccinia trilobata* ♂	Onset of fruit (1 trial) **^b^**^ & ^**^c^**
Coccinia rehmannii var. rehmannii ♀ × Coccinia rehmannii aff. var. littoralis ♂	Onset of fruit (1 trial) **^b^**
Coccinia rehmannii var. rehmannii ♀ × *Coccinia trilobata* ♂	Abortion of flower (2 trials) **^b^**

In contrast to *Coccinia sessilifolia*, *Coccinia grandis* is fertilized easily by *Coccinia hirtella* and *Coccinia sessilifolia*, although the species neither co-occur, nor are closely related. The cross resulted in offspring, which was growing vigorously but sterile, as pollen sacs did not open (Table 3). Hybrid pollen that was extracted from the pollen sacs of fully open flowers was also not able to fertilize *Coccinia sessilifolia* (1 trial). The occurrence of sex chromosomes in *Coccinia grandis* might result in gene dosage imbalance, which interferes with the floral development, leading to sterile offspring. The inability of female *Coccinia grandis* to be fertilized by Coccinia rehmannii aff. var. littoralis and *Coccinia trilobata* (Table 4) might be explained by the fact that the chromosome numbers differ (see Table 2) and translocations lead to gene loss in hybrid genomes and thus inviability of the offspring. On the other hand, the cross between female *Coccinia hirtella* and male *Coccinia trilobata* (not sympatric) produced a purely intermediate F1 generation, which flowers vigorously despite the difference in chromosome numbers (see Table 2 and 3). Although the anthers open like in fertile flowers, unlike in *Coccinia grandis* hybrids, the pollen of this hybrid was not able to fertilize female flowers of *Coccinia hirtella* (1 trial), *Coccinia grandis* (2 trials), or *Coccinia sessilifolia* (2 trials).

Roy and Roy (1971a) report an intergeneric cross between a female *Coccinia grandis* and a male individual of the monoecious *Diplocyclos palmatus*, resulting in a morphologically intermediate F1 offspring in 5% of the trials. All F1 individuals are female, indicating that the X chromosome bears at least one gene for maleness suppression, which is dominant over the maleness genes of *Diplocyclos palmatus*. Whether the F1 is fertile, is not clear, as the authors report successful back-crossing only with a female [*sic*, male?] *Coccinia grandis* but not with *Diplocyclos palmatus*. To the author’s knowledge, there are no reports of the F2 generation. However, if the parental sexes were the other way around (male *Coccinia grandis* × female *Diplocyclos palmatus*), fertilization was not possible.

## Plant – biotic environment interactions

### Pollination

Although bee pollination is observed for only a few species, most *Coccinia* species exhibit characters that support a general attraction to bees. The petal color is commonly pale yellow but can also range to white, pale pink or bright orange, with green, yellow, orange to purple venation. Anthesis is during the day in *Coccinia abyssinica*, Coccinia adoensis var. aurantiaca, *Coccinia grandiflora*, *Coccinia grandis*, *Coccinia hirtella*, *Coccinia megarrhiza*, *Coccinia microphylla*, *Coccinia rehmannii*, *Coccinia sessilifolia*, and *Coccinia trilobata* (pers. observ.) but often only for a few hours (in, e.g., *Coccinia megarrhiza*, *Coccinia rehmannii*). Zimmermann (1922b) reports pollen release in *Coccinia grandiflora* at 6.30 a.m. before blooming of the flower, opening of the flower between 7 and 8 a.m. and wilting after noon. Ash (*J.W. Ash 898*) reports flower opening a.m. in *Coccinia schliebenii*. Anthesis time in the other *Coccinia* species is not reported, but also likely to happen during the day. The scent is rather weak and dull sweetish, resembling that of honey melon, in *Coccinia abyssinica*, *Coccinia grandiflora*, *Coccinia hirtella*, *Coccinia rehmannii*, *Coccinia sessilifolia*, and *Coccinia trilobata*, weak but fresh in *Coccinia grandis*, and intense, sweet, and fruit-like (like honey melon) in Coccinia adoensis var. aurantiaca and *Coccinia megarrhiza*. The only evidence of an alternative to bee pollination is *Coccinia ogadensis*, which is reported to smell of rotten meat (*P. Ellis 163* and *383*). However, it is unclear whether the flowers emit a fetid scent or whether the smell comes from crushed vegetative parts, as it is known from *Momordica foetida* or *Kedrostis foetidissima* (Jacq.) Cogn. (Jeffrey 1967).

Bee pollination is confirmed for *Coccinia rehmannii* (*C.J. Ward 12250*), Coccinia adoensis var. aurantiaca (Fig. 10b), *Coccinia grandiflora*, and *Coccinia grandis*. Observed pollinators of *Coccinia grandis* are *Trigona apicalis* Smith, 1857 and *Trigona collina* Smith, 1857 in Thailand (Jongjitvimol and Wattanachaiyingcharoen 2006), and *Megachile* sp. in Cambodia (H. Schaefer, pers. comm.). The present author observed a halictid bee (Fig. 10b; identification by H. Schaefer, pers. comm.) in a male Coccinia adoensis var. aurantiaca walking on the globose anther head and collecting pollen in the corbicula. Stigmas in *Coccinia* are lobed (Fig. 11b) or bulging (Fig. 11c), and nectaries are located presumably in the hypanthium, so one can assume stripping of the pollen from the venter when crawling into the flower. Zimmermann (1922b) also observed the circling around the anther head in the large-flowered *Coccinia grandiflora*. He identified the visiting small bee as *Trigona* sp. He also noted that a bee just having visited a *Momordica* flower walked on the inner side of the corolla loading dorsally located pollen on the anthers of a male *Coccinia grandiflora* flower.

### Seed dispersal

There are no observations of actual seed dispersal but mammals and birds appear to be attracted by the fruits and likely act as seed dispersers. Fruit bats such as *Cynopterus sphinx* (Vahl, 1797) feed on *Coccinia grandis* fruits in Thailand (Elangovan et al. 2001; Ruby et al. 2000). Fruits of *Coccinia grandis* are also taken up by birds (Bhatt and Kumar 2001) and eaten by humans (Voigt 1845). Elephants also feed on *Coccinia grandis* (Mubalama 2000) and are possibly also seed dispersers. From its introduction to Pacific Islands, dispersal of *Coccinia grandis* by humans is well-known (Muniappan et al. 2009). Human dispersal, in many cases likely to be intentional (Starr et al. 2010), also explains the occurrence of this species in the Neotropics and even in Missouri, USA. Some occurrences in Australia can also be explained by escape from gardens. Zimmermann reports feeding on *Coccinia grandiflora* fruits by birds, small mammals but also snails and beetles (Zimmermann 1922a), the latter two unlikely being seed dispersers. The forest weaver *Ploceus bicolor* Vieillot, 1819 was observed to feed on fruits of *Coccinia mackenii* (Bleher et al. 2003). Stanford and Nkurunungi (2003) report differing preference of *Coccinia* plant parts by gorillas. Whereas the gorillas feed on the leaves and fruit pulp of *Coccinia mildbraedii* but not the seeds, they take only the leaves of *Coccinia barteri*.

Successful seed germination in Munich Botanical Garden indicates that passage through a digestive tract is not necessary at least for *Coccinia abyssinica*, Coccinia adoensis var. jeffreyana, *Coccinia grandiflora*, *Coccinia grandis*, *Coccinia hirtella*, *Coccinia megarrhiza*, *Coccinia microphylla*, *Coccinia rehmannii*, *Coccinia sessilifolia*, and *Coccinia trilobata*. However, whether seeds would survive intestine passage and the role of endozoochoric dispersal is also not known.

### Interaction with ants

Many species of *Coccinia* bear extranuptial glands (nectar producing glands outside of the flower) on the lower lamina of the leaves and/or on the bracts and probracts (Figs 7b, 8a). The glands are sunken into the surface and are surrounded by cells with a thicker cell wall (Muhammad Ilyas 1992; pers. observ.). Ants take up the sweet-tasting sap in *Coccinia grandiflora* (Zimmermann 1922b) and in *Coccinia grandis* (pers. observ.). Whereas Ilyas (1992) reports aggressive behavior of the ants on herbivores for Indian *Coccinia grandis*, the present author could not observe this in Tanzanian *Coccinia grandis*. Nieuwenhuis von Üxküll-Güldenbandt (1907) found a weak attraction of ants and heavy damage by herbivores in *Coccinia grandis* in Bogor Botanical Garden (Java, Indonesia). In addition, Zimmermann (1922b) does not find aggressive behavior in *Coccinia grandiflora* either but reports that the ants attacked a caterpillar he had placed onto the plant. Agarwal and Rastogi (2008), on the other hand, report a significant reduction in residence time of herbivores on the cucurbit *Luffa aegyptiaca* when ants are patrolling on the plant. Most likely, there is no close relationship to certain ant species as guardians, and plant-defense is carried out only by a few ant species. How *Coccinia* species without or few probracts, bracts or sublaminal extrafloral nectaries (e.g., *Coccinia microphylla*) react when damage by herbivores occurs, is unknown. Agarwal and Rastogi (2008) found an increase of total numbers of extrafloral nectaries over time but did not discuss changes of nectary density as reaction to grazing.

### Diseases and parasites

Some research has been undertaken on parasites and diseases for *Coccinia grandis* for its status as crop but also as weed. As *Coccinia grandis* is naturalized on several Pacific islands, in Australia, and the Neotropics, the plants can either overgrow other plants or represent a non-specific host for diseases of cucurbitaceous crops (Bamba et al. 2009; Muniappan et al. 2009). Its rapid growth can become problematic, as Pangelinan (2002) reports that *Coccinia grandis* covered 35% of the vegetation of the island of Saipan only eleven years after its introduction.

Many different organisms are reported to live in, on, or to feed from *Coccinia* species. Beetle and fly larvae are either a disease for *Coccinia*, or in some cases, they are used to eradicate *Coccinia grandis*. Fruits of *Coccinia grandis* are a host for the larvae of the melon fly *Bactrocera* (= *Dacus*) *cucurbitae* (Coquillett, 1899), a tephritid fruit fly (Uchida et al. 1990). *Bactrocera cucurbitae* larvae usually populate the fruits but are also reported to hatch from galls (Murthy 1959). The galls are not produced by these flies, however, but by the gall midge *Lasioptera* (= *Bimba*) *toombii* (Grover, 1962) (Bhatia and Mahto 1968). The gall infestation is interpreted as non-specific, as the female fly might not be able to differentiate between the gall and an unripe fruit, which would be the usual target. In addition, also the tephritid fruit fly *Dacus ciliatus* Loew, 1862 infests the galls, sometimes even together with *Bactrocera cucurbitae* (Bhatia and Mahto 1968). The galls in *Coccinia grandis* do not only result from *Lasioptera toombii* but can also be produced by the Itonidid gall midge *Neolasioptera cephalandrae* Mani, 1934 (Dharmamaraju 1968), which is reported to be the major disease in *Coccinia grandis* in India (Unni et al. 1976). The galls induced by *Neolasioptera cephalandrae* also appear to be gateway for a fungal infection with a mold, which is identified tentatively as *Cladosporium* sp. (Krishnamurthy 1984).

Other major cucurbit pests can also use *Coccinia grandis* as a host such as *Diaphania* (= *Palpita*) *indica* (Saunders, 1851) (Lepidoptera: Pyralidae), *Aulacophora foveicollis* (Lucas, 1849) (Coleoptera: Chrysomelidae), *Leptoglossus australis* (Fabricius, 1775) (Hemiptera: Coreidae), *Aphis gossypii* Glover, 1877 (Hemiptera: Aphididae), *Liriomyza* spp. leafminers (Diptera: Agromyzidae), *Bemesia* spp. white flies (Hemiptera: Aleyrodidae) (Bamba et al. 2009), and *Epilachna vigintioctopunctata* (Fabricius, 1775) (Coleoptera: Coccinellidae) (Maurice and Ramteke 2012).

As a result of the damage that can be done to cucurbitaceous crops and of its weedy behavior on Pacific islands, larvae of the clearwing moth (Sesiidae) *Melittia oedipus* Oberthür, 1878 and the weevil (Curculionidae) species *Acythopeus burkhartorum* O’Brian, 1998 and *Acythopeus cocciniae* O’Brian, 1998 were introduced to Hawaii for biological pest control against *Coccinia grandis* (Muniappan et al. 2002). Immediately after hatching from the eggs, *Melittia oedipus* larvae bore into the stems, where they live and pupate after two to four months (Chun 2002). This moth, originating from Zanzibar (Oberthür 1878), appears to be quite specific as larvae only rarely develop on *Cucumis sativus* L. (Chun 2001). Also *Zehneria guamensis* (Merrill) Fosberg, a Guam endemic, is not attacked by *Melittia oedipus* (Bamba et al. 2009; Reddy et al. 2009). As *Coccinia grandis* is a noxious weed in Hawaii (Hawaiian Department of Agriculture 1992) active search for pests for biological control was undertaken, which led to the discovery of two new beetle species from Kenya: *Acythopeus burkhartorum* whose larvae produce galls in young shoots, and *Acythopeus cocciniae* whose larvae mine the leaves (Chun 2002; O’Brian and Pakaluk 1998). O’Brian and Pakaluk report a close morphological similarity of both *Acythopeus* species to *Acythopeus cucurbitae* (Marshall), which is a major pest on various cucurbitaceous crops in Africa, the Middle East, and South India.

Many crop plants are attacked by root parasites or diseases, but there is little known from *Coccinia*. Only root lesion nematodes *Pratylenchus dasi* Fortuner, 1985 (nom. nov. for *Pratylenchus capitatus* Das & Sultana, 1979) and *Pratylenchus crassi* Das & Sultana, 1979 were described from the soil around the roots of *Coccinia grandis* (Das and Sultana 1979; Siddiqi 2000), but it is not known if they harm the plants.

The only known plant parasite growing on *Coccinia* is the hemiparasitic vine *Cuscuta chinensis* Lam., which is reported to grow on *Coccinia grandis* in Gujarat, India (Patel and Patel 2010).

Several fungi have been reported from *Coccinia* (Table 5). The rust fungus *Puccinia windhoekensis* Mennicken, Maier & Oberw. was described on *Coccinia rehmannii* (Mennicken et al. 2005), although Berndt (2007) noticed a great similarity of this rust to *Pratylenchus ctenolepidis* Ramachar & Bagyanar. Berndt could not confirm the identity of the host specimen, so it seems to be likely that it was misidentified, since *Ctenolepis cerasiformis* looks quite similar to *Coccinia rehmannii*.

**Table 5.** List of fungi reported from *Coccinia* species (sorted by phylum of the fungus).

Fungus	Host	Symptom	Citation
*Plasmopara cubensis* (Berk. & M.A.Curtis) C.J.Humphrey (Peronosporales, Oomycota)	*Coccinia grandis*	Downy mildew	Selby (1899)
*Alternaria pluriseptata* (P.Karst. & Har. ex Peck) Jørst. (Saccharomycetales, Ascomycota)	*Coccinia grandis*	Fruit rot	Chagale and Bhale (2010)
*Cercospora cocciniae* Munjal, Lall & Chona (Dothideales, Ascomycota)	*Coccinia grandis*	Leaf spot disease	Rangaswami and Chandrasekaran (1961)
*Cercospora elaterii* Pass.	*Coccinia grandis*	Leaf spot disease	Rangaswami and Mahadevan (2004)
*Colletotrichum gloeosporioides* (Penz.) Sacc. (Glomerellales, Ascomycota)	*Coccinia grandis*	Fruit rot	Bhagavan Reddy and Reddy (1987)
*Colletotrichum orbiculare* (Berk. & Mont.) Arx	*Coccinia grandis*	Anthracnose fruit rot	Imbumi (2004)
*Corynespora cassiicola* (Berk. & M.A.Curtis) C.T.Wei (Pleosporales, Ascomycota)	*Coccinia grandis*	Leaf blight	Philip et al. (1972)
*Curvularia pallescens* Boedijn (Pleosporales)	*Coccinia grandis*	Black rot	Imbumi (2004)
*Erysiphe cichoracearum* DC. ex Merat (Erysiphales, Ascomycota)	*Coccinia grandis*	Powdery mildew	Imbumi (2004)
*Fusarium moniliforme* J.Sheld. (Hypocreales, Ascomycota)	*Coccinia grandis*	Fruit rot	Kapoor et al. (1981)
*Geotrichum candidum* Link (Pleosporales)	*Coccinia grandis*	Fruit rot	Chagale and Bhale (2010)
*Sphaerotheca fuliginea* (Schltdl.) Pollacci (Erysiphales)	*Coccinia grandis*	Powdery mildew	Imbumi (2004)
*Puccinia cephalandrae* Thümen (Uredinales, Basidiomycota)	*Coccinia quinqueloba*	Rust	Berndt (2007)
*Puccinia cephalandrae-indicae* Syd. & P.Syd.	*Coccinia grandis*	Rust	Berndt (2007)
*Puccinia physedrae* Syd.	*Coccinia barteri*	Rust	Berndt (2007)
*Puccinia windhoekensis* Mennicken, Maier & Oberw.	*Coccinia rehmannii*?	Rust	Mennicken et al. (2005)
*Rhizoctonia solani* Khun (Cantharellales, Basidiomycota)	*Coccinia grandis*	Fruit rot	Bhagavan Reddy and Reddy (1988)

There are several reports of plant viruses from *Coccinia* species. Purcifull and colleagues (1988) tested the infectability of several Cucurbitaceae to different plant viruses. They found that *Coccinia grandis* can be infected by the papaya ringspot virus type W (PRSV-W) and the Trichosanthes virus but not by the cucumber mosaic virus, squash mosaic virus, watermelon mosaic virus-2, and the zucchini yellow mosaic virus. PRCV-W infections of *Coccinia grandis* are also reported from several Pacific islands (Davis and Ruabete 2010).

Verma et al. (1983) suggest a yet undescribed mosaic virus, which is expressed in the occurrence of deformed leaves and a mosaic pattern in *Coccinia grandis* leaves. A strain of the Moroccan watermelon mosaic virus, a Potyvirus, can infest *Coccinia barteri* (Owolabi et al. 2012), whereas the infection of *Coccinia sessilifolia* with this virus, maybe a different strain, failed (van der Meer and Garnett 1987).

### Use, economic potential, and phytochemistry

Several *Coccinia* species are used by tribal communities, mainly as a food source but also for cultural applications (for details see species descriptions). *Coccinia grandis* is notable for its economic value (although often cited erroneously as *Coccinia cordifolia* or *Coccinia indica*), whereas the importance of *Coccinia abyssinica* is only regional. Other species are used by local tribes only.

*Coccinia grandis* is used in a wide variety of applications. The plant is well-known in India, where its fruits had an impact even in classical Sanskrit literature. The red fruits are regularly used to describe lips, such as those of a beloved wife, who is described by her husband in Kālidāsa’s poem Meghadūta (Wilson 1867) or those of the goddess Sita and the god Rama in the epic Ramayana (Dutt 1891–1894). However, the fruits are also edible (raw when ripe and cooked when unripe) and are valued for their high content of carotenoids, esp. lycopene (Barua and Goswami 1979). Also young shoots and leaves are eaten as spinach and contain high amounts of lutein and other carotenoids (Addis et al. 2009; Wasantwisut and Viriyapanich 2003). The high carotenoid value is of special importance in developing countries, as vitamin A deficiency is widespread among young children and pregnant women (WHO 2009). Social marketing has proven to be valuable in promoting the use of *Coccinia grandis* to prevent vitamin A deficiency (Chittchang et al. 1999). Domestication of *Coccinia grandis* is in an early stage but promising cultivars are developed in South and SE Asia (Bharathi 2007; Engle et al. 1998; Ramachandran and Subramaniam 1983). Additionally, the leaves seem to be a good source of selenium and potassium, as well as vegetable protein (Xu et al. 2003; Xu et al. 2004). In Africa, *Coccinia grandis* is mostly used from wild collections (Addis et al. 2009; Imbumi 2004). Contraindications to the use of *Coccinia grandis* are also reported (Adanson 1757; Orech et al. 2005), but these might also be the result from either misidentification or regional chemo-varieties with differing amounts of secondary metabolites.

*Coccinia grandis* has been used in Indian traditional medicine for several hundred years (Nadkarni and Nadkarni 1976; Ramachandran and Subramaniam 1983). There are some studies that suggest a high potential for the use of *Coccinia grandis* leaf extracts in diabetes treatment (Azad Khan et al. 1980; Kuriyan et al. 2008; Munasinghe et al. 2011). Parts of the observed effects are explained by inhibition of gluconeogenesis in the liver due to repression of glucose-6-phosphatase (Hossain et al. 1992) and fructose-1,6-bisphosphatase (Shibib et al. 1993). Also an activating effect on the promotor of the glucose transporter gene GLUT1 from rats is reported (Graidist and Purintrabipan 2009). Eshrat (2003) observes a positive effect of *Coccinia grandis* in rats with hyperlipidemia, which is often connected to diabetes. However, its effectivity in diabetes treatment and the overall experimental design is in dispute (Ramachandran and Subramaniam 1983; Sadikot 2009), and more research to test the medical value is necessary. Since 2005, more than 15 studies researched chemical compounds in *Coccinia grandis* and tested their validity in folk medicine. Some applications by tribal people could be reproduced *ex situ* but research is still in its infancy. Suggested effects are, e.g., anti-anthelmintic (Dewanjee et al. 2007b), anti-tussive (Pattanayak and Sunita 2009), hepatoprotective (Moideen et al. 2011; Vadivu et al. 2008), antioxidative (Umamaheswari and Chatterjee 2008), antipyretic, analgesic, and anti-inflammatory (Niazi et al. 2009), anti-ulcerogenic (Mazumder et al. 2008), and antimicrobial (Bulbul et al. 2011; Dewanjee et al. 2007a; Farrukh et al. 2008; Shaheen et al. 2009). Antimicrobial activity is explained by the occurrence of a protease inhibitor (Satheesh and Murugan 2011). Observed xanthine oxidase inhibition and antiuricaemical activity (Umamaheswari et al. 2007) suggests a use for gout treatment. Female rats with hyperprolactinemia-caused infertility regain fertility when treated with an aqueous extract of *Coccinia grandis* stems and leaves (Jha et al. 2010).

*Coccinia abyssinica* is mainly an Ethiopian tuber crop. Under the name *anchote*, its starch containing (c. 20%) tubers are an important staple food in the SW semi-humid highland regions (Aga and Badada 1997; Asfaw 1997; Hora 1995). Additionally, the tubers contain a relatively high amount of calcium, which might explain the local belief that the plant helps with repairing bone fractures and displaced joints (Hora 1995). Locally (around Dembi Dolo, Oromia), young shoots and leaves are also eaten (Hora 1995). Although the fruits of the cultivated landraces are not eaten (Getahun 1973), the use might be beneficial due to the carotenoid content of the fruits, which are likely to be comparable to those of *Coccinia grandis*. However, fruits of wild races of *Coccinia abyssinica* are already used (Asfaw and Tadesse 2001). In Wollega (W Ethiopia), *Coccinia abyssinica* is also used to treat gonorrhea, tuberculosis, and cancer, as well as in traditional ceremonies and celebrations and for animal fattening (Gelmesa 2010). Currently, much effort is put into the development of *anchote* to increase the yield by selection of cultivars with larger tubers and by improving crop growing with better suited fertilizers (Abera and Guteta 2007; Bekele et al. 2014; Mengesha et al. 2012).

Also other species of *Coccinia* are used as food sources but if so, then only locally. In these species, such as *Coccinia sessilifolia*, some wild landraces lack bitter substances (Bosch 2004; Dinter 1912). Bitterness in Cucurbitaceae is mainly caused by triterpenoids called cucurbitacins, although not all cucurbitacins are bitter. Cucurbitacins are often cytotoxic and often exist as β-glucosides (Miró 1995). All *Coccinia* species screened so far contain cucurbitacins, although the cucurbitacin type, organ, and time of expression differ greatly. Whereas fruits of *Coccinia hirtella* and *Coccinia quinqueloba* contain glycosidic cucurbitacin B, *Coccinia adoensis* from South Africa contains aglycosidic cucurbitacin B only in unripe fruits and traces of cucurbitacin D but not in ripe fruits (Rehm et al. 1957). Unripe fruits of *Coccinia rehmannii* and *Coccinia sessilifolia* are not bitter, and therefore lack bitter cucurbitacins (Enslin et al. 1956). Njoroge and Newton (1994) tested the type and distribution of cucurbitacins within the plant in different Cucurbitaceae and found in Kenyan *Coccinia adoensis* plants cucurbitacins H, I, and R in the stem but no cucurbitacins in the roots, leaves, fruits, or seeds. *Coccinia trilobata* was found to contain the cucurbitacins B, D, and G in the stems, cucurbitacin D, H, I, and R in the leaves, and cucurbitacin G in the fruits, with no cucurbitacins in the roots and seeds. However, there seems to be much variability, as there are reports of edible (non-bitter) *Coccinia trilobata* leaves (*Coilly*? *24*, *F. Msajiri 19*). *Coccinia grandis* is also reported to contain cucurbitacin B (Bhakuni et al. 1962), and bitter and sweet fruited varieties are known. Guha and Sen (1973) find that cucurbitacin B has an antigibberelic effect, and its occurrence in seeds of *Coccinia grandis* might enable or increase dormancy of the seeds.

Cucurbitaceae are also known for the occurrence of non-coded amino acids, such as citrulline in *Citrullus lanatus* (Thunb.) Matsum. & Nakai (Wada 1930). In a survey of such amino acids in Cucurbitaceae *Coccinia grandis* and *Coccinia hirtella* seeds were found to contain citrulline in low amounts, β-(pyrazol-1-yl)-L-alanine in very high amounts and the peptide Γ-L-glutamyl-β-(pyrazol-1-yl)-L-alanine in intermediate amounts (Dunnill and Fowden 1965). This pattern is similar to those that were found in *Diplocyclos tenuis* (Klotzsch) C.Jeffrey, *Acanthosicyos horridus* Welw. ex Hook.f., *Peponium hirtellum* Keraudren, *Ruthalicia eglandulosa* (Hook.f.) C.Jeffrey, *Dactyliandra welwitschii* Hook.f., and *Ctenolepis cerasiformis* (Stocks) C.B.Clarke (all in the same tribe as *Coccinia*).

## Evolution and phylogeny

Recent phylogenetic analyses (Kocyan et al. 2007; Schaefer and Renner 2011b) show that *Coccinia* belongs to the tribe Benincaseae with a moderately supported sister group relationship to the genus *Diplocyclos*. However, the backbone of the tribe is not resolved and the relationship of the *Coccinia*-*Diplocyclos* clade to the other genera is unknown. *Citrullus*, *Cucumis*, or *Scopellaria* cluster with this clade but each without support, and morphological characters also do not seem to suggest any closer relatives.

**Figure 17.**
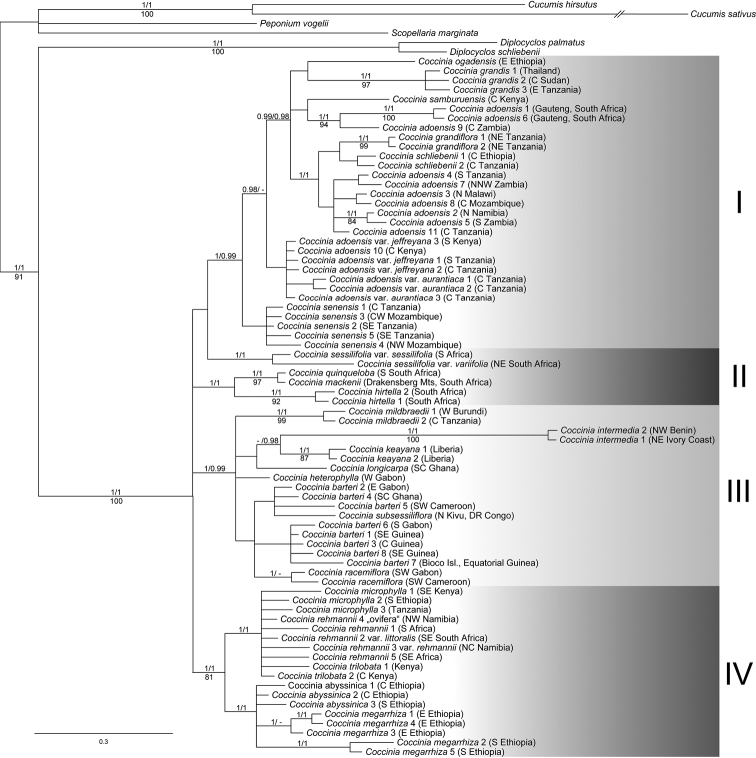
Phylogenetic relationships in *Coccinia* based on five plastid DNA loci (*mat*K, *ndh*F–*rpl*32 intergenic spacer (IS), *rpl*20–*rps*12 IS, *trn*L intron, *trn*L–*trn*F IS, *trn*S–*trn*G IS) obtained for 75 accessions from 24 species. Shown is the topology of the 50% majority rule consensus tree obtained from Bayesian analysis including simple gap coding for ingroup InDels. Numbers above the branches are posterior probability values ≥ 0.98 with values “with InDel coding” first, followed by “without InDel coding.” Numbers below the branches are bootstrap support values from ML analysis. Topologies from the different analyses were not contradictive, although some clades were not resolved without gap coding. Roman numbers indicate clades as discussed in the text: I = *Coccinia adoensis* clade, II = *Coccinia quinqueloba* group, III = *Coccinia barteri* clade, and IV = *Coccinia rehmannii* clade.

**Figure 18.**
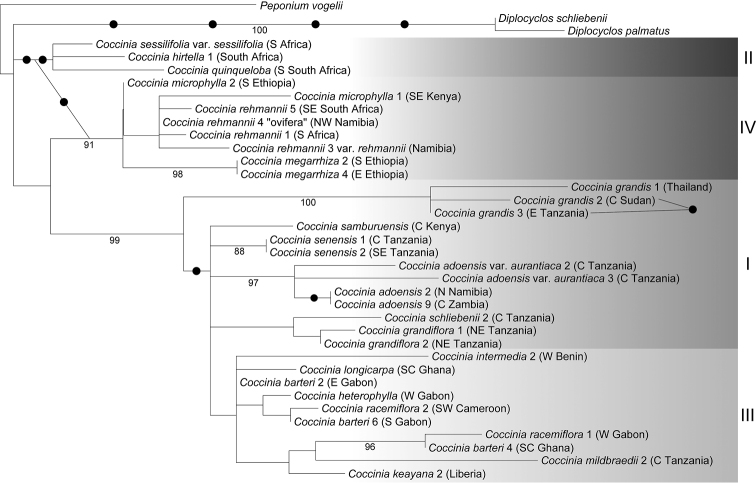
Phylogenetic relationships in *Coccinia* based on 505 nucleotides of the nuclear *LEAFY*-like 2^nd^ intron, obtained for 37 accessions from 23 species analyzed under maximum likelihood (ML) and the GTR + Γ model. Numbers below branches refer to ML bootstrap support > 80% from 1000 replicates. The dots at nodes and behind the two accessions refer to uniquely shared indels. Roman numbers indicate clades as discussed in the text: I = *Coccinia adoensis* clade, II = *Coccinia quinqueloba* group, III = *Coccinia barteri* clade, and IV = *Coccinia rehmannii* clade.

Both phylogenies, plastid (Fig. 17) and the nuclear *LEAFY*-like 2^nd^ intron (Fig. 18), suggest four major clades, although the backbone lacks bootstrap or posterior probability support (Holstein and Renner 2011b). The *Coccinia rehmannii* clade (IV) is well-supported in all phylogenies. The *Coccinia quinqueloba* group (II) is well-supported in the plastid DNA analysis, and consists of *Coccinia hirtella*, *Coccinia mackenii*, and *Coccinia quinqueloba*. Additionally, *Coccinia sessilifolia* belongs to this group, but it is only supported here by the nrDNA data. According to the nuclear data, the *Coccinia barteri* clade (III) is nested within the *Coccinia adoensis* clade (I). The plastid analysis tree separates these two clades but without support.

The *Coccinia rehmannii* clade (IV) consists of five species. *Coccinia abyssinica* and *Coccinia megarrhiza* are sister species from Ethiopia and semi-arid parts of N Kenya and Somalia (Fig. 20). They differ ecologically with the former species occurring in the semi-humid highlands and the latter one in the semi-arid lowlands. Both species differ weakly in morphology, and hybridization cannot be ruled out. The plastid haplotypes of both species do not form clades in the tree, which might be explained best by incomplete lineage sorting. The other three species of clade IV contain several plastid haplotypes and nrDNA sequences that each also do not form clades. The geographical distribution of the haplotypes is not assessed. The three species, however, are distinct. *Coccinia rehmannii* occurs in southern Africa while the other two species occur in NE Africa. In *Coccinia rehmannii* four forms can be recognized, which are included in the plastid phylogeny: (1) an inland form from dry habitats with small globose fruits (type form / var. rehmannii), (2) a form similar to var. rehmannii, but with larger globose fruits (described by Dinter and Gilg as *Coccinia ovifera*), (3) a long-petiolate and long-peduncled coastal form from the (semi-)humid Southeast (described by Meeuse as var. littoralis), and (4) plants with oblong fruits occurring in all semi-humid areas from the Southeast to the northern parts in the periphery of the *Coccinia rehmannii* distribution (*Coccinia rehmannii* 5; here referred to Coccinia rehmannii aff. var. littoralis). None of these forms cluster together. The other two species differ morphologically and ecologically from each other: *Coccinia trilobata* has, e.g., oblong fruits and occurs in the semi-humid uplands, and *Coccinia microphylla* has globose fruits and occurs in the semi-arid lowlands (Fig. 33). Interestingly, *Coccinia microphylla* does not differ morphologically from the *Coccinia rehmannii* form from the dry inland. This scenario suggests incomplete lineage sorting and a speciation event with ecological differentiation in the northeastern Africa but not in southern Africa as intermediate collections between the four forms are found regularly. The distribution of these three species and the estimated age of this clade of 3.2 Ma suggest either a long distance dispersal or vicariance. As each of the three species contains several plastid haplotypes, vicariance is more likely, which indicates that semi-arid conditions might have prevailed between today’s Tanzania and Zimbabwe. This has been suggested several times for different clades under the term “arid track” (Balinsky 1962; de Winter 1971).

The *Coccinia quinqueloba* clade (II) is only supported in the nrDNA phylogeny, as plastid sequences of *Coccinia sessilifolia* and its distinctly petiolate variety *variifolia* lack synapomorphies that support a closer relationship to any clade in *Coccinia*. The two varieties of *Coccinia sessilifolia* occur in the semi-arid and sub-semi-humid inland (Fig. 40; see species description), whereas the other three species prefer more humid habitats in the Southeast (Fig. 30; see species description; Holstein and Renner 2011b). One species, *Coccinia hirtella*, occurs in the rather open habitats, especially in the Drakensberg Mountains, which receive high amounts of rainfall. *Coccinia mackenii* occurs in remnant forest sites in the humid Southeast of southern Africa, whereas *Coccinia quinqueloba* occurs only in coastal bushlands of the Eastern Cape, where it receives less precipitation than the other two species but has a more evenly distributed water availability all over the year (Holstein and Renner 2011b). As *Coccinia mackenii* and *Coccinia quinqueloba* do not co-occur but have similar ecologies, and as they only slightly differ morphologically but hardly produce hybrids (see chapter Hybridization and crossing experiments), a recent allopatric speciation event is probable. The lack of differentiation in the plastid sequences over 3500 bp between two accessions might support this hypothesis. In contrast to the *Coccinia rehmannii* clade, all species of this clade occur exclusively in southern Africa, although the clade is older (c. 5.0 Ma vs. 3.2 Ma).

**Figure 19.**
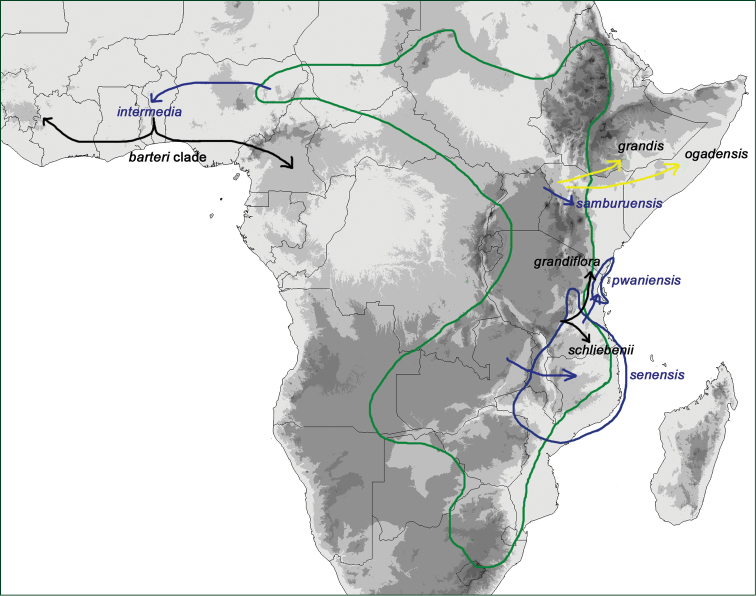
Scenario of evolution in the *Coccinia adoensis* clade. The green line surrounds today’s distribution of *Coccinia adoensis*. Blue lines surround today’s distributions of *Coccinia senensis* and *Coccinia pwaniensis*. Blue arrows indicate peripatric speciation without shift in precipitation preference. Yellow arrows indicate speciation with shifts towards more arid habitats. Black arrows indicate speciation with shift towards more humid habitats.

The *Coccinia adoensis* clade (I) contains several morphologically and ecologically well differentiated species (Holstein and Renner 2011b). There are three subclades in the plastid tree with accessions having the name *Coccinia adoensis*. The type form (Fig. 21; see species description) from Ethiopia (no DNA sequences available) is morphologically inseparable from South African forms (*Coccinia adoensis* 1 and 6). Geographically between those two populations, however, there are many populations that mostly differ gradually in length and density of trichomes. Two forms (with especially dense and long trichomes, respectively) could be assessed geographically and are accepted by the present author as varieties. The Coccinia adoensis var. aurantiaca accessions are neither in the plastid nor in the nuclear tree monophyletic but share a dense indumentum. These forms cluster in the nuclear tree with collections that have a less dense indumentum and thus are rather referred to as Coccinia adoensis var. adoensis (Fig. 18). In the plastid tree, these collections cluster together with a Kenyan specimen of Coccinia adoensis var. adoensis and var. jeffreyana (Fig. 17). Coccinia adoensis var. jeffreyana, however, shares the longer trichomes (Figs 3a, 5c) of some *Coccinia senensis*, but it differs from these by lacking subulate calyx lobes and a 569 bp deletion in the *trn*S^GCU^–*trn*G^UUC^ intergenic spacer region. However, one collection that does not differ morphologically from the variety *jeffreyana* (*R.E. Gereau and C.J. Kayombo 3582*) clusters within the East African forms of *Coccinia adoensis*, which indicates either homoplasy of the trichome length or gene flow. Additionally, gene flow among the *Coccinia adoensis* clades might also occur. Holstein and Renner (2011b) found a collection from Namibia (*Coccinia adoensis* 5) that contained ITS sequences that are otherwise found exclusively in the South African and East African plastid haplotypes. Thus it can be suggested that all these forms belong to one widespread species, *Coccinia adoensis*, which contains different plastid haplotypes. From this widespread species, several populations might have undergone ecological and morphological divergence. *Coccinia grandiflora* and *Coccinia schliebenii* are nested within one *Coccinia adoensis* subclade, and they occupy rather humid habitats while *Coccinia grandis* and *Coccinia ogadensis* occupy more arid habitats. Some populations probably evolved parapatrically in former times with morphological shifts (*Coccinia senensis*, *Coccinia pwaniensis*, *Coccinia samburuensis*) or evolved in allopatry (*Coccinia intermedia*) (Holstein and Renner 2011b; Fig. 19). Some populations, however, did not diverge sufficiently to be taxonomically well-separated as a species, such as Coccinia adoensis var. aurantiaca.

The *Coccinia barteri* clade (III) mostly consists of rainforest species from West and Central Africa, except for the recently described *Coccinia intermedia* (Holstein and Renner 2011a). *Coccinia mildbraedii* (including *Coccinia ulugurensis*) also differs ecologically, as it occurs in mountain forest communities not in typical lowland rainforests as does the rest of the species. The phylogenetic position of *Coccinia intermedia* is unclear as the resolution within this clade is generally poor. *Coccinia intermedia* shares morphological characters with *Coccinia adoensis*, especially the open campanulate flowers. Both species occur in the same habitat type with the former occurring in West Africa and the latter north and east of the Central African rainforests. If the *Coccinia barteri* clade is indeed nested in the *Coccinia adoensis* clade, as suggested by the nuclear phylogeny, then it is possible that *Coccinia intermedia* might have split allopatrically from a proto-*Coccinia adoensis* species and is sister to the rest of the *Coccinia barteri* clade (Fig. 19). Then, the common ancestor of the other species of the *barteri* clade might have shifted the habitat preference towards perhumidity once and evolved allopatrically in refugia during arid periods of the Pliocene and Pleistocene. Alternatively, the habitat of *Coccinia intermedia* would be explained as a reversal from a rainforest distributed common ancestor of the *Coccinia barteri* clade. As the frequency of the Pleistocene climatic oscillations increased, reproductive isolation did not always occur, leading to weak morphological differentiation of interbreeding populations, such as in the polymorphic *Coccinia barteri* (Holstein and Renner 2011b).

**Figure 20.**
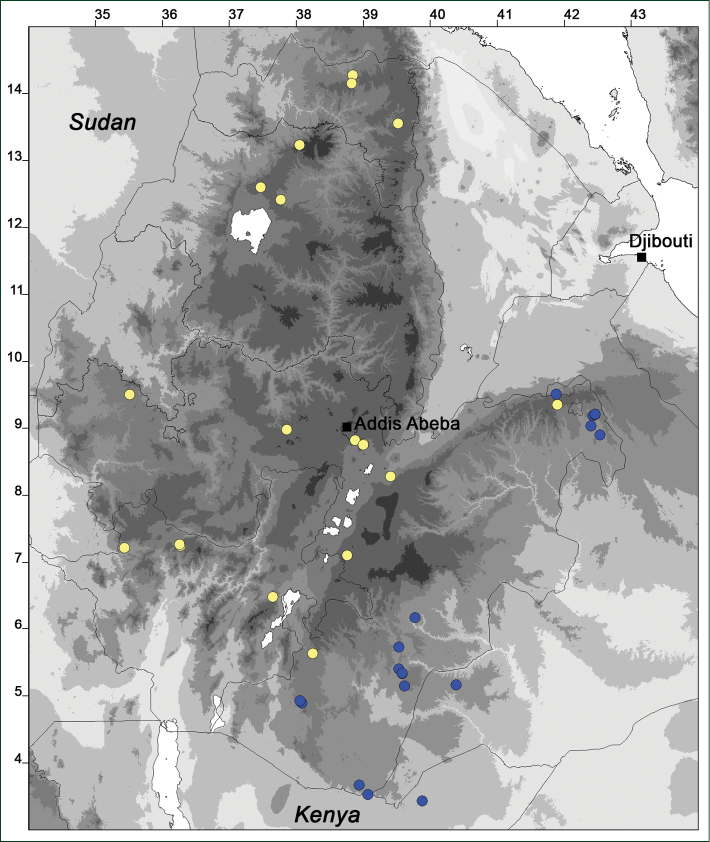
Distribution map of *Coccinia abyssinica* (pale yellow dots; based on 23 collections) and *Coccinia megarrhiza* (blue dots; based on 28 collections). For Ethiopia the borders of the regions are given.

**Figure 21.**
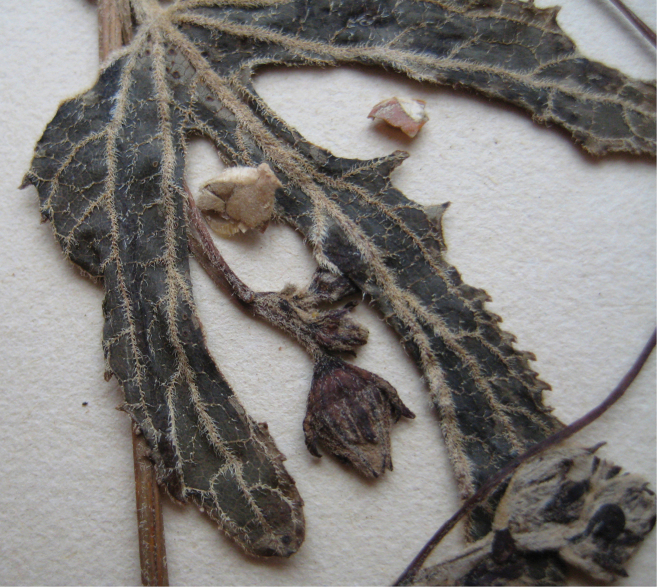
Male inflorescence and leaf of *Coccinia adoensis*; picture taken from lectotype (*G.H.W. Schimper 166* (P00346261)). Note the short bent trichomes, which are a good indicator for this species (but glabrous collections or other kinds of trichomes may occur in this species, too).

## Identification of *Coccinia* species

### Possible confusion with other genera

Some *Coccinia* species are easily confused with collections of other Cucurbitaceae genera (Table 6). The similarity is sometimes striking and without generative structures, one might need some experience to differentiate between the genera.

**Table 6.** African taxa that *Coccinia* species might be misidentified with.

*Coccinia* species	Similar taxon	Differences
*Coccinia barteri*, *Coccinia heterophylla*, *Coccinia racemiflora*	*Bambekea racemosa* Cogn.	*Bambekea racemosa*: petals free, has veins running along the leaf margin *Coccinia*: petals connate, veins not running directly along the margin
*Coccinia barteri*, *Coccinia heterophylla*, *Coccinia racemiflora*	*Cogniauxia* spp.	*Cogniauxia*: petals free and up to 8 cm long, has veins running along the leaf margin, prophylls lanceolate, fruits up to 15 × 8 cm (L × D) large, seeds up to 2 cm long (Schaefer and Renner 2011a) *Coccinia*: petals connate and < 3.5(–4.5 cm), leaf veins not directly running along the leaf margin, probracts ovate or missing, fruit diameter < 4(–5) cm, if length > 6 cm, then not ovate, seeds < 0.8 cm long
W African rainforest species	*Ruthalicia* spp.	*Ruthalicia*: bracts lanceolate, petals free, seeds black or dark brown; *Ruthalicia eglandulosa* with trichomes with a claret-red color (coloration is often at the ends of the long, centrically sunken-in cells) *Coccinia*: bracts ovate or missing, petals connate, seeds gray to beige; trichomes whitish, beige, yellowish or rarely light brownish
rainforest species	*Peponium* spp.	*Peponium*: petals free, male flowers with long-stretched hypanthium and three free stamens, which connect only with the long-stretched anthers, seeds dark colored; *Peponium vogelii*: sessile probracts and bracts are round and up to 3 cm long *Coccinia*: petals connate, male flowers with perianth tube of which the length does not exceed two times the diameter, three already connected filaments and a globose anther head, seeds gray to beige; short petiolate probracts and bracts ≤ 0.5 cm in C & W African species
*Coccinia schliebenii*	*Luffa aegyptiaca* Mill.	*Luffa aegyptiaca*: mostly (2–)3–5-fid tendrils (check as many as possible), petals free, petals bright yellow (in *Luffa acutangula* (L.) Roxb. also dull yellowish), stamens 5 *Coccinia schliebenii*: (1–)2-fid tendrils, petals connate, petals dull yellowish or yellow-orange, stamens 3
*Coccinia schliebenii*	*Lagenaria* spp.	*Lagenaria*: often tooth-like glands at the base of the lamina or along the petiole, trichomes > 1 mm, petals free and white, anthers serpentine *Coccinia schliebenii*: never glands as above, trichomes < 1 mm, petals connate and dull yellowish or yellow-orange, anthers S-shaped
*Coccinia adoensis*	*Eureiandra* spp.	*Eureiandra*: petals free, calyx lobes triangulate to lanceolate, stamens 5, seeds almost globose and whitish (Jeffrey 1967) *Coccinia*: petals connate, calyx lobes lineal, subulate to narrowly triangulate, stamens 3 in a central column, seeds grayish to beige, flattened
*Coccinia microphylla*, *Coccinia rehmannii*	*Ctenolepis cerasiformis* C.B.Clarke	*Coccinia cerasiformis*: large roundish, sinuate-ciliate probract, petals < 5 mm (Jeffrey 1967; Meeuse 1962) *Coccinia*: probract < 3 mm, ovate or missing, petals > 1 cm
*Coccinia microphylla*, *Coccinia rehmannii*	*Dactyliandra* spp.	*Dactyliandra*: large roundish, sinuate-ciliate probract, petals < 5 mm; *Dactyliandra stefaninii* (Chiov.) C.Jeffrey from N Africa lacks the probracts but the seed shape is conspicuously rounded (Jeffrey 1967; Meeuse 1962) *Coccinia*: probract < 3 mm, ovate or missing, petals > 1 cm; seeds asymmetrical (almost falcate)

### Characters for species discrimination

There is no character that is useful for all species. For example, whereas the direction of the calyx lobes can be a useful character for some species (e.g., *Coccinia grandis*, *Coccinia intermedia*, *Coccinia keayana*), it is less useful in others (e.g., in the *Coccinia quinqueloba* clade). Collections without flowers are harder to identify. In some cases it is almost impossible to discriminate between species if flowers are lacking. Identification of only vegetative material is often possible but needs experience. The indumentum can be a useful character; especially the trichomes (length, somewhat also the shape) on the abaxial side of the petiole and the lower leaf lamina can be helpful. However, the trichomes on the adaxial side of the petiole and the leaf margin do not seem to have any purpose for species identification.

### Key to *Coccinia* species

The key is made from observations of herbarium material but also includes some characters from personal observations of living material and observations as given on herbarium labels. Fresh material is not needed, however, to use the key. The term ‘articulate’ refers to dried trichomes that appear wrinkled due to equatorially sunken cell walls (see Fig. 3a) but not to trichomes with ramifications, which have never been observed in *Coccinia*. In the living state, these trichomes are rather long and stiff. The term “dentate” refers to the sometimes colored structures (hydathodes?) at the leaf margin and leaf tip (Figs 6, 7a, 8a, 16a, 16b, 21, 39).

Habitats in this key (not the species descriptions) are given rather crudely and reflect the vegetation that would be found naturally. Savannas and woodlands (tree stands with not largely overlapping canopies) can also include mopane, but also dry forests (larger amounts of deciduous trees and overlapping canopies), deciduous thickets, tall grasslands, and secondary vegetation derived from these. “Rainforests” include gallery forests, semi-deciduous forests derived from rainforests, e.g., in relict areas, perhumid savanna types, and open areas, in which rainforest would be predominant if it was not for human impact, or swamps.

A local key for *Coccinia* from West Africa is provided separately by Holstein and Renner (2011a). If the plant is collected from outside of Africa, then it is *Coccinia grandis*.

**Table:** 

1	Mature leaves sessile (first leaves may be petiolate), rarely subsessile; alive usually bluish-green; glabrous; male flowers solitary or in few-flowered racemes, female flowers solitary; fruit long ovoid, elliptical to spindle-shaped; preferring dry habitats; from S Africa (Figs 2b, 7b, 13b)	**Coccinia sessilifolia var. sessilifolia**
1*	All leaves petiolate; plant not like in 1	**2**
2	Tendrils mostly bifid; usually forest species or from Drakensberg Mts or humid coastal bushland in SE Africa (in E and W Africa also in woodlands or savannas)	**3**
3	Plant with flowers	**4**
4	Corolla ≥ 4 cm long, calyx lobes > 3 mm long; ovaries and fruits long ovoid to cylindrical; E Africa or Ethiopia	**5**
5	Leaf surface usually glabrous, rarely with sparse weak thin trichomes on the abaxial side; leaves profoundly lobed (Fig. 8a); E African (rain) forests of C Zimbabwe and Mozambique to S Kenya	***Coccinia grandiflora***
5*	Leaf surface, at least below (secondary and tertiary veins) densely covered with small trichomes; leaf shallowly or rarely profoundly lobed; margin of humid forests and in forests; from N Mozambique to C–S Tanzania or Ethiopia to South Sudan	***Coccinia schliebenii***
4*	Corolla < 4 cm long	**6**
6	Calyx lobes (> 2.5 mm) subulate (Fig. 28), western C Africa	***Coccinia heterophylla***
6*	Calyx lobes < 2.5 mm, if longer then from S Africa	**7**
7	Calyx lobes > 3 mm, plant from S Africa	**8**
8	Leaf lamina and stem usually densely covered with long (> 0.5 mm) trichomes; lamina profoundly lobate and lobulate; lobe tips usually rounded; pedicels covered with long (> 0.5 mm) trichomes (Fig. 13a)	***Coccinia hirtella***
8*	Leaf lamina and stem glabrous or rarely sparsely covered with long trichomes, with lobes often tapering into an acute tip, only side lobes with a slight lobule on outer side; pedicels glabrous	***Coccinia mackenii***
7*	Calyx lobes < 3 mm, plant not from S Africa	**9**
9	Flowers in lax many-(> 6-)flowered racemes, western C Africa	***Coccinia racemiflora***
9*	Flowers in dense racemes, few-flowered or on a long common peduncle that surpasses the length of the branched part; female flowers may also be solitary	**10**
10	Male flowers on a long common peduncle that surpasses the length of the branched part; female flowers solitary with cylindrical ovary; mountain forests of Kivu Mts, Livingstone Mts, and Eastern Arc Mts, introduced into Kenyan high mts	***Coccinia mildbraedii***
10*	Male flowers in a raceme, in which the common peduncle is shorter than the branched part; female flowers in racemes, clustered or if solitary, then with subglobose to elliptical ovary	**11**
11	Corolla campanulate, calyx lobes erect with recurved tips. Lower leaf surface at maturity often with white speckles and leaf margin with colored teeth. W African semi-humid savannas and woodlands	***Coccinia intermedia***
11*	Corolla urn-, cup-, funnel-shaped or narrow campanulate. Calyx lobes variable but not as above. Lower leaf surface rarely with white speckles, teeth on leaf margin not conspicuously colored. Rainforests of W Africa, C Africa, and in relict forests to Angola, Zambia?, W Tanzania, Uganda, and the Chimanimani Mts (Mozambique, Zimbabwe)	***Coccinia barteri***
3*	Plant with fruits or vegetative parts only	**12**
12	Plant with fruits	**13**
13	Fruit oblong to cylindrical (mature > 5 cm long), plant from E or NE Africa	**14**
14	Lower leaf surface, often also upper surface densely covered with short trichomes; N Mozambique, C and S Tanzania or W Ethiopian to SE South Sudanian mts	***Coccinia schliebenii***
14*	Upper leaf surface glabrous, lower leaf surface glabrous or rarely with some trichomes; plant from E Africa, incl. Kivu Mts and Chimanimani Mts (Mozambique, Zimbabwe); hard to differentiate in shared mountain ranges	**15**
15	Probracts > 3.5 mm (Fig. 8a); corolla > 3 cm, calyx lobes > 3 mm; forests and forest relicts in S Kenya, Mt Meru to Usambara Mts, Eastern Arc Mts, SE Tanzania, N Mozambique, Chimanimani Mts (Mozambique, Zimbabwe)	***Coccinia grandiflora***
15*	Probracts < 3.5 mm; corolla < 3 cm, calyx lobes < 3 mm; forests of Kivu Mts, Livingstone Mts, and Eastern Arc Mts, introduced into Kenyan high mts	***Coccinia mildbraedii***
13*	Fruits ovoid, if long elliptical, then from S Africa	**16**
16	Fruits in lax racemes, plant from western C Africa	***Coccinia racemiflora***
16*	Fruits in dense racemes or solitary	**17**
17	Plant from S Africa; fruits solitary	**18**
18	Leaf surface and stem usually densely covered with long (> 0.5 mm) trichomes; lamina profoundly lobate and lobulate; lobe tips usually rounded (Fig. 13a)	***Coccinia hirtella***
18*	Leaf surface and stem glabrous or sparsely covered with long (> 0.5 mm) trichomes, with lobes often tapering into an acute tip, only side lobes with a slight lobule on outer side	***Coccinia mackenii***
17*	Plant from W to C Africa to Chimanimani Mts (Mozambique, Zimbabwe)	**19**
19	Plant from western C Africa, not distinguishable with confidence without flowers	***Coccinia heterophylla* or *Coccinia barteri***
19*	Plant not from western C Africa	**20**
20	Lower leaf surface at maturity often with white speckles and leaf margin with colored teeth when dry. W African semi-humid savannas and woodlands	***Coccinia intermedia***
20*	Lower leaf surface rarely covered with white speckles, teeth on leaf margin not conspicuously colored. Rainforests of W to C Africa to Chimanimani Mts (Mozambique, Zimbabwe)	***Coccinia barteri***
11*	Plant vegetative only	**21**
21	Lower leaf surface, often also upper surface densely conspicuously covered with short trichomes; N Mozambique, C and S Tanzania or W Ethiopian to SE South Sudanian mts	***Coccinia schliebenii***
21*	Leaves glabrous, or if covered with trichomes, then they are long (> 0.7 mm) or inconspicuous	**22**
22	Plant from S Africa	**23**
23	Leaf surface and stem usually densely covered with long (> 0.5 mm) trichomes; leaves profoundly lobate and lobulate; lobe tips usually rounded (Fig. 13a)	***Coccinia hirtella***
23*	Leaf surface and stem glabrous or sparsely covered with long (> 0.5 mm) trichomes, with lobes often tapering into an acute tip, only side lobes with a slight lobule on outer side	***Coccinia mackenii***
22*	Plant from W to E Africa	**24**
24	Plant from E African (incl. S Kenyan) rainforests or forest relicts, Mt Meru to Usambara Mts, Eastern Arc Mts to Chimanimani Mts (Mozambique/Zimbabwe); probracts > 3 mm (Fig. 8a)	***Coccinia grandiflora***
24*	Plant from W, C, or E Africa, if from E Africa, then probracts < 3 mm	**25**
25	From mountain forests of E Africa, incl. Kivu Mts	***Coccinia mildbraedii***
25*	Rather from lowland rainforests from W Africa, C Africa, or from rainforests surrounding the Western Rift	**26**
26	Plant from western C Africa not confidently distinguishable without flowers	***Coccinia heterophylla*, *Coccinia racemiflora*, or *Coccinia barteri***
26*	Plant not from western C Africa	**27**
27	Lower leaf surface at maturity often with white speckles and leaf margin with colored teeth. W African semi-humid savannas and woodlands	***Coccinia intermedia***
27*	Lower leaf surface rarely with white speckles, teeth on leaf margin not conspicuously colored. Rainforests of W to C Africa to Chimanimani Mts (Mozambique, Zimbabwe)	***Coccinia barteri***
2*	Tendrils usually simple, if not, then from semi-arid habitats or E and NE-African woodlands	**28**
28	Leaves deeply palmately lobed with lineal lobes. If lobes lobulate, then leaf lamina at lobe base as broad as vein. E Ethiopia and C Somalia	***Coccinia ogadensis***
28*	Leaves profoundly, but not deeply lobed, or if deeply lobed, then lobes lanceolate or lobe base broader than vein	**29**
29	Leaves deeply lobed with lanceolate lobes. Male flowers in racemes with short peduncle and pedicels. Rainforests of C Africa and around the Western Rift	***Coccinia subsessiliflora***
29*	Plant not as above	**30**
30	Leaves 7-lobate, rarely 5-lobate. Outer side of lobes serrate to lobulate with conspicuously colored tips. Calyx lobes > 4 mm, corolla > 2.5 cm, fruit ripe > 10 cm long, from Samburu area (C Kenya, E Africa)	***Coccinia samburuensis***
30*	Leaves cordate or 3-lobate. If 5-lobate, then plant not as above. Calyx lobes shorter than above or if longer then corolla shorter. Fruit shorter than above or if longer then from S Africa	**31**
31	Plant glabrous; leaves usually subsessile, 5-lobate. Plant from coastal bushlands of Eastern Cape (South Africa)	***Coccinia quinqueloba***
31*	Plant with trichomes or if glabrous, then from different region	**32**
32	Plant glabrous, glaucous. Fruit long (> 6 cm) elliptical to spindle-shaped. Limpopo Province (South Africa)	**Coccinia sessilifolia var. variifolia**
32*	Plant with trichomes, or if glabrous then not glaucous and from different region	**33**
33	Lower leaf surface, often also upper surface densely conspicuously covered with short trichomes; bracts > 3 mm; calyx > 1 cm, corolla > 4 cm; fruit oblong to short cylindrical, > 5 cm long; N Mozambique, C and S Tanzania or W Ethiopian to SE South Sudanian mts	***Coccinia schliebenii***
33*	Lower leaf surface glabrous or sparsely covered with trichomes. If densely covered with trichomes, then bracts < 3 mm and flowers smaller	**34**
34	Plant with male flowers	**35**
35	Plant glabrous, rarely some trichomes on adaxial petiole and leaf margin. Lower leaf lamina with pale (rarely also black when oxidized) glands towards the base, sometimes also between secondary veins. Margin of mature leaves with claret-red or brownish (black when dry) teeth, when young pale. Flowers solitary, rarely clustered, calyx lobes spreading to reflexed, corolla white or buff (Fig. 7a). NW (Senegal) to NE and E Africa, southern Arabia, S and SE Asia, naturalized or likely to become so in (sub)tropical regions worldwide	***Coccinia grandis***
35*	Plant with trichomes, or if glabrous then different from above	**36**
36	Plant glabrous or rarely with soft multicellular trichomes. Flowers in lax ebracteate racemes, calyx lobes lineal, > 2 mm long, in buds spreading, when mature reflexed (Fig. 32). Rainforests of W Africa (W of the Dahomey Gap)	***Coccinia keayana***
36*	Plant not as above	**37**
37	Plant glabrous. Leaves cordate to subhastate, rarely 3-lobate. Flowers in ebracteate racemes. Calyx lobes erect, at base broader than 0.75 mm, corolla urceolate. Rainforests of W Africa	***Coccinia longicarpa***
37*	Plant different and not from W Africa, or if from W Africa then calyx lobes narrower or spreading to reflexed	**38**
38	Plant glabrous, at maturity often with white speckles on stem, petiole, and lower leaf lamina. Flowers in racemes or 1 solitary. Calyx lobes erect with recurved tips. Corolla campanulate. Semi-humid savannas and woodlands of W Africa	***Coccinia intermedia***
38*	Plant not from W Africa or if so, then rainforest species (sometimes hard to distinguish from *Coccinia intermedia*), or lower leaf surface conspicuously covered with trichomes	**39**
39	Plant glabrous (or puberulous), leaves usually coriaceous. W or C (or western E) African rainforests	**40**
40	Male flowers in racemes with common peduncle shorter than racemose part. Lowland rainforests or rainforest relicts in higher altitudes or along rivers	***Coccinia barteri***
40*	Male flowers in racemes with common peduncle longer than racemose part. Mountain forests from Kivu Mts, Eastern Arc Mts, Livingstone Mts, also introduced in Kenyan high mts	***Coccinia mildbraedii***
39*	Plant conspicuously covered with trichomes or if glabrous, then leaves papery or from NE, E, or S Africa	**41**
41	Plant from S Africa (except C and N Mozambique)	**42**
42	Plant (esp. stem oder petioles) with or without white speckles, perianth tube/hypanthium with long (> 0.7 mm) trichomes or if glabrous then calyx lobes > 2 mm (Fig. 10a)	***Coccinia rehmannii***
42*	Plant without white speckles, perianth tube/hypanthium with short (< 0.7 mm) trichomes or if glabrous then calyx lobes < 2 mm (Fig. 21)	**Coccinia adoensis var. adoensis**
41*	Plant from E (incl. C and N Mozambique), NE, or NC Africa	**43**
43	Flowers clustered, common peduncle < 1 cm, if flower solitary then pedicel usually < 1 cm. NE Africa (incl. N Tanzania)	**44**
44	Upper and lower leaf surface rather densely covered with multicellular trichomes. Plant usually from higher elevations of N Tanzania and Kenya	***Coccinia trilobata***
44*	Upper leaf surface pustulate but without trichomes, or with minute trichomes from pustules. Plant rather from dry habitats and lower elevations. May be hard to distinguish	**45**
45	Leaf margin in mature leaves with conspicuously colored teeth. Plant densely covered with long trichomes that appear articulate when dry (Fig. 9)	***Coccinia megarrhiza***
45*	Leaf margin in mature leaves without conspicuously colored teeth. Plant less densely covered with trichomes or if densely, then trichomes minute (< 0.2 mm) or if longer then not appearing articulate when dry (Fig. 2a)	***Coccinia microphylla***
43*	Flowers in racemes with peduncle > 1 cm (if smaller then from C Tanzania), or if solitary then either pedicel > 1 cm or plant from C Tanzania	**46**
46	Calyx lobes subulate to narrowly triangulate with pointed tip, > 2.5 mm. Petiole and lower leaf surface not puberulous. Plant from E Africa (Tanzania, Mozambique, Malawi)	**47**
47	Leaves 3-lobate, distinctly petiolate, often with few short trichomes on the main nerves of the lower leaf surface. Racemes with > 8 flowers. Coastal forests of Kenya or NE Tanzania (Fig. 23)	***Coccinia pwaniensis***
47*	Leaves subcordate to 3- or 5-lobate, subsessile or distinctly petiolate (Fig. 6). Lower leaf surface glabrous or nerves with short (wart-like) to long trichomes. Racemes < 10 flowers (Fig. 39). SE Tanzania, C and N Mozambique, or Malawi (Fig. 23)	***Coccinia senensis***
46*	Calyx lobes < 2.5 mm or if longer, then not pointed (may be lineal though) or petiole and lower leaf lamina puberulous, or plant from NE Africa (Kenya, Ethiopia, Somalia)	**48**
48	Plant with long (> 0.5 mm) trichomes or with short, narrowly conical trichomes, calyx lobes > 2 mm, lineal. NE Africa	**49**
49	Apex of the cordate leaf or central lobe tapering into a long, acute tip. Male flowers solitary or in racemes with a long common peduncle. Plant from high elevations (Fig. 20)	***Coccinia abyssinica***
49*	Apex of leaf or central lobe retuse, obtuse, or rather abruptly tapering into a short acute tip (Fig. 9). Male flowers solitary, clustered or if in racemes, then peduncle short. Plant rather from lower elevations (Fig. 20)	***Coccinia megarrhiza***
48*	Plant glabrous or with short trichomes (< 0.8 mm), if with longer trichomes then not from NE Africa. Calyx lobes < 2.5 (–3.5) mm long. Taxa in E Africa not easily distinguishable (complex around *Coccinia adoensis*)	**50**
50	Plant with long (> 0.8 mm) trichomes (Figs 3a, 5c), calyx lobes 1.5–3.5 mm long but not with pointed tip. Malawi, C, and S Tanzania, maybe also N Mozambique (Fig. 23)	**Coccinia adoensis var. jeffreyana**
50*	Plant glabrous or with short (< 0.8 mm) trichomes	**51**
51	Lower leaf surface and usually also herbaceous stems, petioles, and upper leaf surface densely covered with short (< 0.5 mm) trichomes. Peduncle often shorter than pedicelled part. Calyx lobes < 2 mm. Corolla orange, rarely yellow? E Africa (C Tanzania; Fig. 23)	**Coccinia adoensis var. aurantiaca**
51*	Lower leaf surface glabrous to densely covered with trichomes, but if so then peduncle longer than pedicelled part. Calyx lobes usually < 2 mm. E, NE, or NC Africa (Fig. 21)	**Coccinia adoensis var. adoensis**
34*	Plant with female flowers, fruits or vegetative	**52**
52	Plant with female flowers	**53**
53	Flowers solitary or in ebracteate racemes. Calyx lobes lineal, spreading in buds, reflexed in mature flowers, > 2 mm long. W African rainforests (W of Dahomey Gap) (Fig. 32)	***Coccinia keayana***
53*	Plant not as above. If with spreading to reflexed calyx lobes, then not from rainforest regions or < 2 mm long	**54**
54	Ovary cylindrical. Calyx lobes broader than 0.75 mm at base, corolla urceolate. W African rainforests	***Coccinia longicarpa***
54*	Ovary shortly elliptical, (ob-)ovoid or globose, if cylindrical then not from W African rainforests. Calyx lobes narrower at base	**55**
55	Flowers in bracteate or ebracteate racemes or solitary. Ovary globose or (ob-)ovoid, if longer then from E Africa. Calyx lobes < 2 mm long. W and C Africa but also in rainforest relicts or mountain forests in E Africa	**56**
56	Female flowers solitary or in racemes. Corolla cup-, urn- or funnel-shaped, not open campanulate. Ovary globose to (ob-)ovoid. W or C (or western E) Africa. Lowland or in relict rainforests in highlands	***Coccinia barteri***
56*	Female flowers solitary. Corolla cup-shaped to campanulate. Ovary long spindle-shaped to oblong. Mountain forests of Kivu Mts, Eastern Arc Mts, Livingstone Mts, also introduced in Kenyan high mts	***Coccinia mildbraedii***
55*	Flowers solitary. Calyx lobes > 2 mm or if shorter then plant not from rainforests from regions as above	**57**
57	Calyx lobes spreading to reflexed, lower leaf surface glabrous with pale glands between main veins. Leaf margin with colored teeth (Fig. 7a). Calyx lobes lineal, in buds spreading, later reflexed. Corolla white or buff. Plant from NW (Senegal) to NE and E Africa, southern Arabia, S and SE Asia, naturalized or likely to become so in (sub)tropical regions worldwide but natively not from C or S Africa	***Coccinia grandis***
57*	Plant with trichomes or if glabrous, then with darkish glands or without glands on lower leaf surface. Calyx lobes not reflexed. Corolla in various colors but not snow-white	**58**
58	Calyx lobes erect with recurved tips, lower leaf lamina with dark glands between veins, sometimes with white pustules on veins and petiole. Margin of mature leaves with colored teeth. Plant from woodlands or savannas of W Africa	***Coccinia intermedia***
58*	Plant not as above and not from W Africa or if so, then not with white pustules and dark teeth	**59**
59	Calyx lobes subulate to narrowly triangulate with pointed tip, > 2.5 mm (Fig. 39); petiole and lower leaf surface not puberulous. E Africa (Tanzania, Mozambique, Malawi; Fig. 23)	**60**
60	Leaves 3-lobate, distinctly petiolate, often with few short trichomes on the main nerves of the lower lamina. Coastal forests of Kenya or NE Tanzania	***Coccinia pwaniensis***
60*	Leaves cordate to 3- or 5-lobate, subsessile or distinctly petiolate. Lower leaf lamina glabrous or nerves with short (wart-like) to long articulate trichomes. SE Tanzania, C and N Mozambique, or Malawi	***Coccinia senensis***
59*	Calyx lobes < 2.5 mm or if longer, then not with pointed tip or then petiole and lower leaf lamina puberulous, or plant from NE Africa (Kenya, Ethiopia, Somalia)	**61**
61	Plant from S Africa (S Angola, Zimbabwe, C Mozambique and further S)	**62**
62	Plant (esp. stem and petioles) with or without white speckles, perianth tube/hypanthium with long (> 0.7 mm) trichomes or if glabrous then calyx lobes > 2 mm (Fig. 10a)	***Coccinia rehmannii***
62*	Plant without white speckles, perianth tube/hypanthium with short (< 0.7 mm) trichomes or if glabrous then calyx lobes < 2 mm (Fig. 21)	**Coccinia adoensis var. adoensis**
61*	Plant from NC, NE or E Africa	**63**
63	Plant with long (> 0.5 mm) trichomes or straight, narrowly conical trichomes or if trichomes short (< 0.2 mm) then ovary globose. Plant from NE Africa (Ethiopia, Kenya, Somalia, N Tanzania)	**64**
64	Apex of leaf or central lobe tapering into a long, acute tip. Plant from high (> 800 m) elevations of Ethiopia (Fig. 20)	***Coccinia abyssinica***
64*	Apex of leaf or central lobe retuse, obtuse, or rather abruptly tapering into a short, acute tip. Plant rather of dry habitats in lower elevation	**65**
65	Plant rather densely covered with long (> 0.5 mm) trichomes that appear articulate when dry. Leaf apex retuse, obtuse, or rather abruptly tapering into a short acute tip (Fig. 9). Leaf margin of mature leaves with dark glands. Ovary ellipsoid, never globose. N Kenya, Ethiopia and likely also Somalia	***Coccinia megarrhiza***
65*	Plant rather laxly covered with trichomes, if denser then trichomes usually minute (< 0.2 mm), if longer then not appearing articulate when dry. Leaf apex rarely obtuse (e.g., around the Usambaras), often abruptly tapering into a short acute tip. Leaf margin never with dark glands. Ovary globose, rarely (ob-)ovoid. N Tanzania, Kenya, Ethiopia and likely also Somalia (Fig. 2a)	***Coccinia microphylla***
63*	Plant glabrous or with short (< 0.5 mm) trichomes, if with longer trichomes then not from NE Africa, ovary not globose	**66**
66	Leaves on upper lamina with short trichomes (Fig. 8b). Calyx lobes 2–5 mm long. N Tanzania or Kenya	***Coccinia trilobata***
66*	Leaves on upper lamina glabrous (but with white pustules) or if with short trichomes, then from C Tanzania. Calyx lobes < 2 mm or if longer, then lower leaf lamina with long (> 0.8 mm) trichomes or puberulous. Taxa in E Africa not easily distinguishable (complex around *Coccinia adoensis*)	**67**
67	Plant with long (> 0.8 mm) trichomes that appear articulate when dry (Figs 3a, 5c), calyx lobes 1.5–3 mm long but not pointed (as in Fig. 39). C and S Tanzania, Malawi, maybe also N Mozambique (Fig. 23)	**Coccinia adoensis var. jeffreyana**
67*	Plant glabrous or with short (< 0.8 mm) trichomes only	**68**
68	Lower sometimes also upper leaf lamina densely covered with short trichomes. Ovary densely covered with short (< 0.5 mm) trichomes. Calyx lobes < 2 mm. Corolla orange, rarely yellow? E Africa (C Tanzania; Fig. 23)	**Coccinia adoensis var. aurantiaca**
68*	Lower leaf lamina glabrous or covered with short trichomes (Fig. 21). Ovary glabrous or only laxly (inconspicuously) covered with short trichomes. Corolla yellowish, pinkish or orange. E, NE, or NC Africa (Fig. 22)	**Coccinia adoensis var. adoensis**
52*	Plant with fruits only or vegetative	**69**
69	Plant with fruits	**70**
70	Fruit long elliptical to cylindrical (> 8 cm). Forest species	**71**
71	Plant from West African rainforests	***Coccinia longicarpa***
71*	Plant from mountain forests of Kivu Mts, Eastern Arc Mts, Livingstone Mts, also introduced in Kenyan high mts	***Coccinia mildbraedii***
70*	Fruit globose to oblong (< 8 cm)	**72**
72	Fruit globose. Plant from dry habitats	**73**
73	Plant from N Tanzania, Kenya, S and SE Ethiopia or Somalia	***Coccinia microphylla***
73*	Plant from S Africa	***Coccinia rehmannii***
72*	Fruit obovoid to oblong, if (sub-)globose, then from humid habitats	**74**
74	Leaf margin at maturity with colored teeth (blackening when dry), lower leaf surface glabrous and with pale glands between main veins, petioles and veins at maturity often with white pustules. Fruit (ob-)ovoid to elliptical. Plant natively not from C or S Africa	***Coccinia grandis***
74*	Lower leaf surface without glands or with darkish glands or if with pale glands, then mature leaves without colored teeth on leaf margin	**75**
75	Fruit subglobose to obovoid-elliptical, in raceme or if solitary, then rainforest species	**76**
76	Fruit in ebracteate raceme or solitary. Plant from W Africa (W of Dahomey Gap) (hardly distinguishable in shared distribution range)	***Coccinia keayana* or *Coccinia barteri***
76*	Fruit in bracteate or ebracteate raceme. Plant from W and C Africa and in relict rainforest patches along the Western Rift	***Coccinia barteri***
75*	Fruit solitary or 1–3 clustered but not in raceme. Plant not from rainforests	**77**
77	Plant glabrous, at maturity often with white speckles on stem, petiole, and lower leaf lamina. Fruit subglobose to obovoid-elliptical, solitary or 1–3 clustered. Semi-humid savannas and woodlands of W Africa	***Coccinia intermedia***
77*	Plant different and not from W Africa. If from (eastern) W Africa then fruit often with sterile apex (“beak”)	**78**
78	Fruit elliptical to oblong, often with sterile apical tip (“beak”). Unripe with dark green/light green longitudinal stripes or mottling. Seeds rather lenticular and with symmetrical shape (Fig. 14a). Lower leaf lamina glabrous or with trichomes, if trichomes appearing articulate and > 0.5 mm, then from C and S Tanzania, maybe also Malawi or N Mozambique	**79**
79	Leaves 3-lobate. Leaf surface glabrous but veins and petiole often with few short trichomes. Plant from coastal forests of SE Kenya to E Tanzania (Fig. 23)	***Coccinia pwaniensis***
79*	Plant different or from different region (hardly distinguishable)	**80**
80	Plant with long (> 0.8 mm) trichomes (Figs 3a, 5c) or if reduced, then stiff but not bent (as in Fig. 21), warty or subglabrous. C and S Tanzania, Malawi or C to N Mozambique (Fig. 23)	***Coccinia senensis* or Coccinia adoensis var. jeffreyana**
80*	Plant glabrous or with short (< 0.8 mm), but not warty or articulate appearing trichomes	**81**
81	Stem, petiole, lower leaf lamina, and ovary/young fruit densely covered with short trichomes. C Tanzania (Fig. 23)	**Coccinia adoensis var. aurantiaca**
81*	Stem, petiole, lower leaf lamina glabrous or with short trichomes, but young fruit only with lax indumentum. S, E, NE, or NC Africa (Fig. 22)	**Coccinia adoensis var. adoensis**
78*	Fruit obovoid, shortly to long elliptical, but not oblong and not with conspicuous sterile apical tip (“beak”). Unripe fruits with whitish longitudinal mottling that often has a dark green halo. Seed face rather flat, shape often asymmetrical (Fig. 14b, c). S Africa or NE Africa, incl. Kenya and N Tanzania	**82**
82	Plant from S Africa	***Coccinia rehmannii***
82*	Plant from NE Africa, incl. Kenya and N Tanzania	**83**
83	Upper leaf lamina with fine, short trichomes. Lower leaf lamina with rather shortly (< 0.8 mm) articulate (Fig. 8b) or with narrowly conical trichomes. Plant from higher elevations of N Tanzania and S to C Kenya	***Coccinia trilobata***
83*	Upper leaf lamina glabrous (but with pustules), rarely with narrowly conical trichomes. Lower leaf lamina with often long (> 0.8 mm), articulate or with narrowly conical trichomes	**84**
84	Apex of leaf or central lobe tapering into a long acute tip. Plant from high elevations (> 900 m) (Fig. 20)	***Coccinia abyssinica***
84*	Apex of leaf or central lobe retuse, obtuse, or rather abruptly tapering into a shortly acute tip (Fig. 9). Plant rather from low elevations	**85**
85	Plant rather densely covered with trichomes that appear articulate when dry. Leaf apex retuse, obtuse, or rather abruptly tapering into a shortly acute tip (Fig. 9). Leaf margin of mature leaves with dark teeth. N Kenya, Ethiopia and likely also Somalia (Fig. 20)	***Coccinia megarrhiza***
85*	Plant rather laxly covered with trichomes or if densely, then trichomes minute (< 0.5 mm). Leaf apex, rarely obtuse (e.g., around the Usambaras), often abruptly tapering into a short acute tip. Leaf margin never with dark teeth. Fruit globose to elliptical. N Tanzania, Kenya, Ethiopia and likely also Somalia (Figs 2a, 33)	***Coccinia microphylla***
69*	Plants with vegetative characters only	**86**
86	Plant glabrous, lower leaf surface with pale glands (if strongly oxidized then also dark, but then leaf margin also with black markings/teeth) between veins, veins at maturity often with white speckles. Leaf margin at maturity with colored teeth (Fig. 7a). Plant from W to NE Africa, incl. Kenya and N Tanzania or from outside of Africa	***Coccinia grandis***
86*	Plant with trichomes or if glabrous, then with darkish glands between veins or without glands on lower leaf surface	**87**
87	Plant from W Africa	**88**
88	Leaves cordate to 5-lobate, rarely broader than 10 cm, rather papery, lobes triangulate to narrowly lanceolate or oblong. Lower leaf surface without white speckles, glabrous or with often short, bent trichomes (Fig. 21). Semi-humid savannas and woodlands of N Cameroon, N Nigeria, distribution in the area imperfectly known	**Coccinia adoensis var. adoensis**
88*	Leaves cordate to subhastate to 3- or 5-lobate, mature often > 10 cm wide. Leaf lobes triangulate to broad lanceolate, but not narrowly lanceolate or oblong. Rainforest species or if from semi-humid savannas or woodlands (rarely dry forests), then margin of mature leaves with conspicuously colored teeth and lower leaf lamina often with white speckles	**89**
89	Plant glabrous, at maturity often with white speckles on stem, petiole, and lower leaf surface. Margin of mature leaves with conspicuously colored teeth. Semi-humid savannas and woodlands of W Africa	***Coccinia intermedia***
89*	Plant not as above, from rainforests or gallery forests. Species not confidently distinguishable	***Coccinia barteri*, *Coccinia keayana* or *Coccinia longicarpa***
87*	Plant not from W Africa	**90**
90	Leaves coriaceous. Plant glabrous or puberulous on abaxial side of petiole. Rainforest or mountain forest plant from C Africa or western E Africa (along the Western Rift, Livingstone Mts, Eastern Arc Mts), plants vegetatively hardly distinguishable	**91**
91	Lowland rainforest (in relict sites of western E Africa also in mountains) plant from C Africa, incl. areas around Kivu Mts, Chimanimani Mts, and forests (mountain ranges) along the Western Rift, incl. Uganda	***Coccinia barteri***
91*	Plant from mountain forests of Kivu Mts, Eastern Arc Mts, Livingstone Mts, also introduced in Kenyan high mts	***Coccinia mildbraedii***
90*	Plant not from C African rainforests	**92**
92	Plant from S Africa	**93**
93	Lower leaf surface with usually bent trichomes (Fig. 21), never white speckled, rarely subglabrous	**Coccinia adoensis var. adoensis**
93*	Lower leaf surface with straight, in herbarium collections often articulate appearing trichomes, often with white speckles towards maturity or glabrous	***Coccinia rehmannii***
92*	Plant from E, NE, or NC Africa	**94**
94	Plant from NE or NC Africa	**95**
95	Teeth on leaf margin conspicuously colored. NE Africa	**96**
96	Apex of leaf or central lobe tapering into a long acute tip. Plant from high elevations (> 900 m; Fig. 20)	***Coccinia abyssinica***
96*	Apex of leaf or central lobe retuse, obtuse, or rather abruptly tapering into a shortly acute tip (Fig. 9). Plant rather from lower elevation (< 1200 m) (Fig. 20)	***Coccinia megarrhiza***
95*	Teeth on leaf margin not conspicuously colored. Plant from NC or NE Africa	**97**
97	Lower leaf surface glabrous or with short (often bent) trichomes, cordate to deeply lobate (Figs 2a, 21). Plant from NC and NE Africa (incl. N Tanzania)	**98**
97*	Lower leaf surface with long (> 0.5 mm) trichomes that appear articulate when dry (such as in Figs 8b, 9), or with narrowly conical trichomes. Plant from NE Africa	**99**
98	Stem sometimes pustulate. Leaf shape variable, if lobate then lobes extending and not pointing forward, lobes not oblong to linear. Leaves usually with trichomes, often also on upper lamina and then minute (< 0.2 mm). Trichomes on lower lamina often not only restricted to the veins. (Fig. 2a) Plant rather from low elevation drylands (rarely in higher elevations) of NE Africa (incl. N Tanzania)	***Coccinia microphylla***
98*	Stem glabrous or with short (< 0.5 mm) trichomes but not pustulate. Leaf shape variable (cordate to deeply lobate) but also with oblong to linear lobes that point forward. Leaves glabrous or with (often bent) trichomes, on upper leaf lamina rarely beset with trichomes in this distribution area. Trichomes on lower leaf lamina usually restricted to the veins (Fig. 21). Plant from woodlands and semi-humid habitats	**Coccinia adoensis var. adoensis**
99	Leaves cordate to lobate but not lobulate. If profoundly lobate, then central lobe lanceolate or ovate tapering into an acute tip. Lower leaf surface with long (> 0.5 mm) trichomes that appear articulate when dry, or with narrow conical trichomes. Plant from higher elevations (> 900 m) or cultivated, from Ethiopia (Fig. 20)	***Coccinia abyssinica***
99*	Leaf reniform to lobate, rarely lobulate. Apex of leaf or central lobe retuse, obtuse, rather abruptly tapering into a shortly acute tip (Fig. 9), if longer tapering into an acute tip then lobes lobulate. Lower leaf surface with long (> 0.5 mm) trichomes that appear articulate when dry, only rarely with conical trichomes. Plant rather from lower elevations (< 1200 m) (Fig. 33)	***Coccinia microphylla***
94*	Plant from E Africa	**100**
100	Leaves 3- or 5-lobate. Lobes extending, not pointing towards apex, broadly triangulate, elliptical, ovate or somewhat angulate but not narrow, oblong, or lineal. Upper and lower leaf surface with short, white trichomes that appear articulate when dry (Fig. 8b). Plant from higher elevations of N Tanzania and Kenyan highlands (Fig. 33)	***Coccinia trilobata***
100*	Upper leaf surface glabrous (but pustulate) or if with trichomes then leaf shape different or from different region	**101**
101	Stem sometimes pustulate. Leaf shape variable, if lobate then lobes extending and not pointing forward, lobes not oblong to linear. Leaves usually with trichomes, often also on upper lamina and then minute (< 0.2 mm). Trichomes on lower lamina often not only restricted to the veins. (Fig. 2a) Plant rather from low elevation drylands (rarely in higher elevations) of E Africa (Kenya, N Tanzania)	***Coccinia microphylla***
101*	Stem not pustulate, leaves with or without oblong to elliptical lobes. If upper lamina densely covered with minute trichomes then from C Tanzanian woodlands. If plant with long articulate appearing trichomes then from highlands of C to S Tanzania, Malawi or N Mozambique	**102**
102	Leaves 3-lobate (rather small auriculate), upper surface glabrous (but pustulate), lower lamina glabrous, but often with short trichomes on main veins. Coastal forests of SE Kenya and NE to E Tanzania (Fig. 23)	***Coccinia pwaniensis***
102*	Plant not as above or from different area (in some cases hard to distinguish)	**103**
103	Lower leaf surface with long (> 0.5 mm) trichomes that appear articulate when dry or reduced to warts, rarely almost glabrous; sometimes leaves subsessile (Fig. 23)	***Coccinia senensis* or Coccinia adoensis var. jeffreyana**
103*	Lower leaf surface glabrous or with short, thin, straight or bent trichomes	**104**
104	Lower leaf surface (also often upper lamina), petiole and stem rather densely covered with short trichomes. C Tanzania	**Coccinia adoensis var. aurantiaca**
104*	Lower leaf lamina glabrous or covered with trichomes, if densely then upper lamina glabrous (but pustulate) or with few straight trichomes but not tomentose. Widespread in E Africa	**Coccinia adoensis var. adoensis**

## Taxonomic treatment

Herbarium abbreviations follow Index Herbariorum (http://sciweb.nybg.org/science2/IndexHerbariorum.asp). Digital collections were accessed from the homepages of the corresponding herbaria, except for “JPS” (= JStor Plant Science; http://plants.jstor.org/) and “CVH” (= Chinese Virtual Herbarium; http://www.cvh.org.cn/).

Species concepts in this treatment mainly follow the morphospecies concept but also include ecological aspects (habitats) and biogeography. Apart from easily recognizable distinct forms, it was tried to include molecular data (plastid and nuclear; Figs 17, 18) from as many forms as possible to check whether they cluster together or not. Accessions in polytomies are treated as one species as long as they are not morphologically or ecologically (habitat) distinct or are distantly distributed, if not contra-indicated otherwise (e.g., full crossing compatibility in Asian and African *Coccinia grandis*). Names have been synonymized if no character was found to separate confidently the collections from the type material. Names have been changed in status (in this treatment to varieties), when characters to separate the collections change in degree, rather than absence/presence.

The minimum leaf size and petiole length were taken from leaves on the same node as open flowers or fruits. Leaf length is measured from the attachment point of the petiole on the lamina to the apex.

### Coccinia

Taxon classificationPlantaeCucurbitalesCucurbitaceae

Wight & Arn., Prodr. fl. Ind. orient.: 347. 1834.

Cephalandra Schrad. ex Eckl. & Zeyh., Enum. pl. afric. austral. 2: 280. 1836. Type species: *Bryonia quinqueloba* Thunb.
Physedra Benth. & Hook.f., Gen pl. 1(3): 827. 1867. Indirectly lectotypified by Jeffrey (1962: 361) Type species: *Physedra heterophylla* Hook.f.
Staphylosyce Benth. & Hook.f., Gen pl. 1(3): 828. 1867. Type species: *Staphylosyce barteri* Hook.f.

#### Type species.

see *Bryonia grandis* L.

#### Description.

Dioecious. Perennial climbers or creepers. Stems up to 20 m, glabrous or covered with simple smutty-white to yellowish trichomes. Leaves alternate, simple, paired with a tendril. Leaves sessile (Coccinia sessilifolia var. sessilifolia), subsessile to distinctly petiolate. Petioles up to 16.5 cm. Petioles glabrous or covered with simple trichomes. Leaves 0.7–20 × 1.1–23 cm, reniform, cordate to deeply palmately 3- to 7-lobate, sometimes lobulate. Lobes triangulate, ovate, elliptical to linear. Margin entire to more or less densely serrate, dentate. Teeth inconspicuous or colored. Leaf apex obtuse, acute to acuminate. Upper leaf surface with clear or whitish pustules, sometimes with trichomes emerging from the lamina or from pustules. Nerves glabrous or with simple trichomes. Lower leaf surface paler than upper surface, glabrous or with simple trichomes. Probracts caducous or persistent, ovate, up to 4.5 mm long. Lower surface keeled or bulging outwards, often with extranuptial glands. Tendrils simple or unequally bifid. Flowers and inflorescences emerging from leaf axils. Male flowers solitary, fascicled or in up to 20-flowered racemes. If solitary flowers and racemes are developed, then solitary flowers occurring before the racemes (within the plant and per node). Common peduncle of raceme 0.5–10 cm, pedicel of flowers in racemes 0.3–1.8 cm, glabrous or with indumentum as on stem but often less dense. Bracts ovate, up to 4 mm long or missing. Pedicel of solitary flowers 0.2–8.5 cm, glabrous or with simple trichomes. Perianth tube glabrous or more or less densely covered with trichomes. Calyx connate, campanulate, rarely cupulate or urceolate, glabrous, puberulous or with long, simple trichomes. Calyx lobes 0.5–15 mm, triangulate, lineal or subulate; reflexed, spreading to erect. Corolla connate, campanulate, urn-shaped or tubular, 0.7–6.2 cm long; white, dull yellow to orange, salmon; lobes 0.3–4.7 cm, inside densely covered with multicellular trichomes, of which some end with a glandular endcell. Filament column (greenish-)white or orange, anther head pale yellowish green to orange, pollen sacs S-shaped. Female flowers solitary, in pairs or in racemes. Common peduncle 0.3–2.1 cm, glabrous or puberulous. Pedicel of flowers in racemes 0.3–1 cm, glabrous or with simple trichomes, pedicels of solitary flowers 0.7–5 cm, glabrous or puberulous. Calyx and corolla as in males but with hypogynous ovary. Calyx in few cases urn-shaped. Style columnar, greenish yellow, yellow, or orange. Stigmas bulging or 2-lobed, greenish yellow, yellow, or orange. Staminodes 3, attached to the perianth, white (also yellowish or orange?), anthers reduced. Ovary glabrous or with simple, short to long trichomes that then appear articulate when dry. Fruits 1.8–30 × 1.4–5 cm, globose, ovoid, elliptical, or cylindrical; glabrous or with sparse trichomes. Unripe fruits glaucous green to green, sometimes with white, white-and-green or rarely green longitudinal mottling. Ripe fruits orange-red to scarlet red; unicolored or rarely with white to yellowish longitudinal mottling. Seeds enclosed in a hyaline hull, 4.5–7 × 2–3.5 × 1–1.5 mm (L/W/H), symmetrically or asymmetrically obovate, apex round, base narrowed, obtuse, round or square-edged. Face flat to lenticular. Seed surface, depending on the extraction mode, rugulose or filamentose.

### 1. Coccinia abyssinica

Taxon classificationPlantaeCucurbitalesCucurbitaceae

(Lam.) Cogn. in A.DC. & C.DC., Monogr. Phan. 3: 536. 1881.

Bryonia abyssinica Lam., Encycl. 1(2): 497. 1785.
Coccinia abyssinica Type: Cultivated. Unknown, from seeds sent by Bruce (Jeffrey 1962) from Ethiopia, cultivated in Paris Royal Botanical Garden, male, fl, *Anon. in herb. J.-B. Lamarck s.n.* (Holotype: P-LAM! [P00307815, digital image: P-LAM]).
Bryonia macrophylla Ser. in DC., Prodr. 3: 308. 1828.
Coccinia abyssinica Type: without location [probably Ethiopia]. Male and female, fl, 1815, *Anon. in coll. [E.]Thibaud s.n.* (Holotype: G-DC!).
Cucumis striatus A.Rich., Tent. Fl. Abyss. 1: 295. 1847.
Coccinia abyssinica Type: Ethiopia. [Tigray]: Mt Sholada near Adwa, fr, [Aug], *R. Quartin-Dillon s.n.* (Lectotype, designated here: P! [from “Sholada”]).
Coccinia abyssinica Type: Ethiopia. [Tigray]: [Mt] Selleuda [Mt Sholada near Adwa], fr, *R. Quartin-Dillon s.n.* (Syntype: P! [P05621224, digital image: P]).
Cucurbita exanthematica Fenzl ex A.Rich., Tent. Fl. Abyss. 1: 296. 1847.
Coccinia abyssinica Type: Ethiopia. Without detailed location, female, fl, *G.H.W. Schimper 1418* (Lectotype, designated here: W! [digital image: WU]; isolectotypes: BM!, G!, P! [P05621261, digital image: P], TUB! [TUB004727], non TUB-004726!).
Coccinia diversifolia Naudin ex C.Huber, Cat. Print. 1864: 6. 1864. *Cephalandra diversifolia* (Naudin ex C.Huber) Naudin, Ann. Sci. Nat. Bot. ser. 5, 5: 19. 1866.
Coccinia abyssinica Type: Cultivated. From seeds sent by Schimper from Ethiopia, cultivated in Paris Botanical Garden and Huber’s Garden in Olbia [Hyères, France], *C.V. Naudin s.n.*(Lectotype, designated by Jeffrey (1962: 347): P! [P06745719, digital image: P]; isolectotypes: P [P06745720, digital image: P], G-DC (2)!, K).
Coccinia diversifolia var. glabrescens Cogn. in A.DC. & C.DC., Monogr. Phan. 3: 537. 1881.
Coccinia abyssinica Type: Ethiopia. Chaqou-Choada, 2000 m, in thicket, male, fl, 21 Jul 1852, *G.H.W. Schimper 250* (Lectotype, designated here: P! [sheet with descriptive text]; isolectotypes: P (2)!).

#### Description.

Perennial climber. Stems up to 5 m, covered with more or less dense, articulate, dirty-white to yellowish trichomes, rarely glabrous. Petioles 1.5–14 cm, at least on nerves more or less densely covered with articulate trichomes, rarely glabrous. Leaves 7.5–12 × 6.5–12 cm, often cordate to profoundly 3- or 5-lobate. If lobate then central lobe dominating, over-all shape rather (long) cordate (Fig. 4a). Lobes triangulate, ovate to elliptical. Margin more or less densely serrate, dentate. Teeth rarely (if so then small) pale brownish colored in living state or blackish when dried. Leaf apex acute, or if leaf lobate then central lobe acute to long acuminate. Upper leaf surface with clear or whitish pustules, sometimes with some trichomes. Lower leaf surface with soft trichomes articulate appearing when dry or sparsely with stiff narrowly conical trichomes, which can appear warty when short or broken off. Probracts up to 3 mm long. Tendrils simple. Male flowers solitary or in long-pedicelled few-flowered racemes. Pedicel with indumentum as on stem. Common peduncle of raceme 2.5–10 cm, pedicel of flowers in racemes up to 1.5 cm, indumentum as on stem or less dense. Bracts up to 1.7 mm long or missing. Solitary flowers with up to 5 cm long pedicel with trichomes as on stem. Perianth tube more or less densely covered with articulate trichomes. Calyx lobes 2–4 mm, lineal-subulate, upright. Corolla c. 1.4 cm long, yellow to slightly orange, darker on the lobes, lobes up to 5 mm. Filament column white, anther head pale yellowish green, pollen sacs yellow. Female flowers solitary. Pedicel up to 3.5 cm long, indumentum as on stem to glabrous. Style not seen. Stigma shape not seen, yellow. Staminodes not seen. Ovary with long trichomes, often appearing articulate when dry. Hypanthium more or less densely covered with articulate trichomes, calyx lobes and corolla as in males. Fruits 5.5–6 × 3.5–4 cm, short elliptical, glabrous, orange-red sometimes with yellow longitudinal mottling. Seeds 5–6 × 3 × 1.5 mm (L/W/H), slightly asymmetrically obovate, face flat (Fig. 14b).

#### Phenology.

Flowering time: June–October.

#### Distribution.

Fig. 20. Ethiopia (Amhara, Oromia, Southern Nations, Nationalities and People’s Region, Tigray). Elevation 1300–2800 m. On limestone, sandstone, black soil, chromic nitisol (Mengesha et al. 2012), loam, on deep to shallow soil. Along lake shores among *Typha* sp., in *Podocarpus*-*Celtis* forest (clearings) and degraded forms of these, evergreen shrubs (e.g., *Euclea* sp.).

#### Use.

Edibility of fruits is disputed and may differ between wild and cultivated forms (*E. Westphal & J.M.C. Westphal-Stevels 1951* and *1953*). Tuberous roots boiled for food (*T. Ebba 250*), young shoots and leaves are eaten when cooked (Hora 1995). For details see chapter Use, economic potential, and phytochemistry.

#### Vernacular names.

Dawuro: shushe, ushushe (Hora 1995); Galinya [Oromo]: anchote (Getahun 1974b); Kefinya [Kaffa]: ajjo (Hora 1995); Tigrinya: wouchich (*G.H.W. Schimper 1048*); Wollamo [Wolleyta]: ušuše (*W. Kuls 681*). The Kefinya name is not exclusive for *Coccinia abyssinica* but also used for another crop, *Plectranthus edulis* (Vatke) Agnew.

#### Remarks.

The occurrence of monoecy has been reported by *W.J.J.O. de Wilde et al. 7805*, but the seen specimens contained male flowers only. If both sexes are found on the same individual, this is likely to be a case of leaky dioecy (see also section on Chromosomes and sex determination).

#### Taxonomic remarks.

The *Coccinia abyssinica* specimen in the Lamarck herbarium must be the holotype, since there is only one specimen of *Coccinia abyssinica* in the herbarium of Lamarck in Paris and none in the herbarium of Sonnerat, which he has seen, too. The specimen in the Linnaean herbarium was not annotated with a corresponding name.

*Cucurbita exanthematica* Fenzl ex A.Rich. is commonly recognized as a synonym of *Coccinia grandis* with a K.G.T. Kotschy collection as type. However, the label on the *Kotschy 308* specimens merely state the species name, the locality, and “frutices scandens” (= climbing on shrubs; W. Greuter – pers. comm.), which cannot be regarded as a diagnostic feature. The label is printed and therefore effectively published but not validly so. Valid publication of that name was effected by Achille Richard (1847), but he chose a different specimen (*G.H.W. Schimper 1418*), which belongs to *Coccinia abyssinica*. The *Schimper 1418* specimens bear printed labels on which Fenzl designated a variety of his invalid name with the phrase “var. foliis superioribus integris (non lobatis)”. The phrase, however, is also not a validation since the species to which this variety is supposed to belong, is not validly published either (Art. 41.3a and b ICN). Naudin (1859) suggested that Eduard Fenzl mixed-up some specimens. He accepted the *Kotschy 308* specimen as a synonym of his *Coccinia schimperi* and recognized the similarity of the Schimper specimen to Lamarck’s Bryonia (Coccinia) abyssinica and *Cucumis striatus*.

The identity of *Cucumis striatus* A.Rich. is not obvious. There are two original specimens with this name in P herbarium: one from Selleuda (P05621224) and the other one from Sholada, both names for the same mountain near the city of Adwa. The P05621224 specimen consists of a ripe fruit, a drawing of the fruit, and a tiny fragment of a leaf. Cogniaux identified this specimen as *Coccinia adoensis*. However, the fruit is ovoid, which would be unusual for that species in which fruits are long ovoid to short cylindrical and often have a sterile apex (“beak”). Since there are no seeds, which would help to clear this problem up easily, the fruit shape is the only usable character. The leaf fragment might be *Coccinia adoensis* but it is too small to be certain, and it is loose so it might also be debris from another specimen. The other original specimen (with a number “26” from “Sholada”) contains much leaf material and fruits. The fruits are darker than in the first type specimen. The indumentum of the lower leaf lamina matches certain *Coccinia abyssinica* collections, as does the leaf shape (cf. *G. Negri 703*, *G.H.W. Schimper 250*) although they are not very typical. This specimen is not close to *Coccinia adoensis*, therefore the present author chose it to be the lectotype and to synonymize the name *Cucumis striatus* with *Coccinia abyssinica*.

#### Specimens examined.

(Selection, in total: 57) Ethiopia. Amhara: Sanka-Berr [vicinity of Reb river] and Begemder [highland], *G.H.W. Schimper 1446* (E [E00303229], S [S08-12052], S [S08-12057], W, Z (3)). Oromia: 32 km from Addis Abeba on road to Debre Zeit [Debre Zeyit], *E. Westphal & J.M.C. Westphal-Stevels 1951* (BR [BR0000008914613], EA, MO, PRE, WAG [WAG0225550], WAG [WAG0225551], WAG [WAG0225552]) & *1953* (MO, WAG [WAG0225546], WAG [WAG0225547]). SNNPR: Bonga, near Roman Catholic Mission, *W.J.J.O. de Wilde & B.E.E. de Wilde-Duyfjes 7805* (MO, WAG [WAG0225537], WAG [WAG0225538], WAG [WAG0225539]). Tigray: 18 km along road from Adu Abun to Axum, 14°09'N, 38°49'E, *J.J.F.E. de Wilde 7059* (M, WAG [WAG0225544], WAG [WAG0225545]).

### 2a. Coccinia adoensis (Hochst. ex A.Rich.) Cogn. var. adoensis

Taxon classificationPlantaeCucurbitalesCucurbitaceae

Momordica adoensis Hochst. ex A.Rich., Tent. Fl. Abyss. 1: 293. 1847. *Coccinia adoensis* (Hochst. ex A.Rich.) Cogn. in A.DC. & C.DC., Monogr. Phan. 3: 538. 1881.
Coccinia adoensis (Hochst. ex A.Rich.) Cogn. var. adoensis Type: Ethiopia. [Tigray]: Adwa, near church “Eta Mariam”, mixed specimens, male and female, fl, fr, 5 Jun 1837, *G.H.W. Schimper Iter Abyss. sect. 1 no. 166* (Lectotype, designated here: P! [P00346261, digital image: JPS, P]; isolectotypes: BM! [BM001010004], BR [BR0000008351036], BR! [BR0000008351039, digital image: BR, JPS], BR! [BR0000008886781, digital image: BR, JPS], G! [G00301597], G! [G00301598], G-DC!, HBG! [HBG506429, digital image: JPS], K! [K000542642, digital image: JPS, K], K! [K000542643, digital image: JPS, K], K! [K000542644, digital image: JPS, K], L! [L0057303, digital image: JPS, L], LG! [LG0000090028250; digital image: JPS], M! [M0105779, digital image: JPS], M! [M0105780, digital image: JPS], P! [P00346261, digital image: JPS, P], P! [P00346262, digital image: JPS, P], S! [S10-19076, digital image: S], TUB [TUB004719, digital image: JPS], TUB [TUB004720, digital image: JPS], W! [W 0011091, digital image: WU], W! [digital image: WU]).
Coccinia adoensis (Hochst. ex A.Rich.) Cogn. var. adoensis Type: Ethiopia. [Tigray]: near Adwa, in thicket, *R. Quartin-Dillon s.n.* (Syntype: P!).
Coccinia adoensis (Hochst. ex A.Rich.) Cogn. var. adoensis Type: Ethiopia. [Amhara?]: Ouodgerate Province, *A. Petit no.*? (Syntype: P?).
Bryonia convolvuloides A.Rich., Tent. Fl. Abyss. 1: 289. 1847.
Coccinia adoensis (Hochst. ex A.Rich.) Cogn. var. adoensis Type: Eritrea/Ethiopia. [Gash-barka/Tigray]: Chiré [borderland between both regions, between Tekezé river and Mareb/Gash river], male, fl, *R. Quartin-Dillon & A. Petit s.n.* (Lectotype, designated here: P! [P05621227, digital image: P]; isolectotype: P! [P05621226, digital image: P]).
Coccinia adoensis (Hochst. ex A.Rich.) Cogn. var. adoensis Type: Ethiopia. No location, male, 1844, *R. Quartin-Dillon & A. Petit s.n.* (Syntype: P! [P05621255, digital image: P]).
Bryonia jatrophiifolia A.Rich. [sphalm. *Bryonia jatrotrophæfolia* A.Rich.], Tent. Fl. Abyss. 1: 289. 1847. *Coccinia jatrophiifolia* (A.Rich.) Cogn. [sphalm: *Coccinia jatrophæfolia* (A.Rich.) Cogn.] in A.DC. & C.DC., Monogr. Phan. 3: 535. 1881.
Coccinia adoensis (Hochst. ex A.Rich.) Cogn. var. adoensis Type: Ethiopia. [Tigray]: in valley near Adwa, male, fl, Aug 1839, *R. Quartin-Dillon & A. Petit s.n.* (Lectotype, designated here: P! [P00346263, digital image: JPS, P; K neg. 2990], syntype: P! [P05621222, digital image: P]).
Coccinia adoensis (Hochst. ex A.Rich.) Cogn. var. adoensis Type: Ethiopia. [Tigray]: near Tchélatchekanné [Djeladjeranné, in Tekezé river valley (Gillett 1972), on label as “Tchessu Hetchequenné”], male, fl, *R. Quartin-Dillon s.n.* (Syntype: P! [P00346260, digital image: JPS, P; K neg. 4467], P! [P05621223, digital image: P]).
Cephalandra pubescens Sond. in Harv. & Sond., Fl. Cap. 2: 493. 1862. *Coccinia pubescens* (Sond.) Eyles, Trans. Roy. Soc. South Africa 5(4): 498. 1916. *Coccinia pubescens* (Sond.) Cogn. ex Harms in Fries, Notizbl. Bot. Gart. Berlin-Dahlem 8: 491. 1923. nom. superfl.
Coccinia adoensis (Hochst. ex A.Rich.) Cogn. var. adoensis Type: South Africa. [North West]: Magaliesberg, male and female, fl, Dec, *J. Burke 408* (Lectotype, designated here: K! [K000313229, digital image: JPS, K]; isolectotype: BM! [BM000815207], K! [K000313227, digital image: JPS, K], NBG, Z!).
Coccinia adoensis (Hochst. ex A.Rich.) Cogn. var. adoensis Type: South Africa. North West/Gauteng: at Magalies river, male and female, fl, *C.L.P. Zeyher 588* (Syntype: K! [K000313228, digital image: JPS, K], S! [S08-12187, digital image: S]).
Coccinia hartmanniana Schweinf., Reliq. Kotschy.: 42, t. 27, t. 28. 1868.
Coccinia adoensis (Hochst. ex A.Rich.) Cogn. var. adoensis Type: drawing in protologue, t. 27 (Lectotype, designated here).
Coccinia adoensis (Hochst. ex A.Rich.) Cogn. var. adoensis Type: Sudan. Sinnar Province: no detailed location given, 1860, *R. Hartmann s.n.* (Syntype: ?, if B, then destroyed).
Coccinia adoensis (Hochst. ex A.Rich.) Cogn. var. adoensis Type: South Sudan. At White Nile in the region of the Tschier people [a nilotic tribe also called: Chir, Kir, Mandari, Mondari, Mundari, Shir], [possibly the area between Tombe and Mongalla in Central Equatoria state], 1861, *W. von Harnier s.n.* (Syntype: B destroyed, ?).
Coccinia adoensis (Hochst. ex A.Rich.) Cogn. var. adoensis Type: South Sudan. [al-Qadarif]: Gallabat at Gendua [river], Jun 1861, *H. Steudner 843* (Syntype: ?, if B, then destroyed).
Coccinia adoensis (Hochst. ex A.Rich.) Cogn. var. adoensis Type: Ethiopia. [Amhara]: near Matamma at border to Sudan, after beginning of Jun 1865, *G.A. Schweinfurth Flora of Gallabat 62* (Syntype: ?, if B, then destroyed).
Coccinia rigida Cogn., Bot. Jahrb. Syst. 21: 210. 1895.
Coccinia adoensis (Hochst. ex A.Rich.) Cogn. var. adoensis Type: Tanzania. [Tabora]: Ugunda, near Gonda [Igonda], on ground in wet corn fields, *R. Böhm 176* (Holotype: B destroyed, lectotype, designated here: BR! [BR0000008886804, digital image: BR, JPS]).
Coccinia djurensis Schweinf. et Gilg, Bot. Jahrb. Syst. 34: 357. 1904.
Coccinia adoensis (Hochst. ex A.Rich.) Cogn. var. adoensis Type: South Sudan. [West Bahr al-Ghazal or Warab]: Seriba Ghattas, male, fl, 24 May 1869 or Jun 1869, *G.A. Schweinfurth 1878* (Lectotype, designated here: P!; isolectotypes: B [destroyed], E! [E00303230], G!, K! [K000542640, digital image: JPS, K], PRE [PRE0592944-0, digital image: JPS], S! [S08-12060, digital image: S], Z! [Z-000073403, digital image: Z]).
Coccinia adoensis (Hochst. ex A.Rich.) Cogn. var. adoensis Type: South Sudan. [West Bahr al-Ghazal or Warab]: Seriba Ghattas, male, fl, 24 May 1869, *G.A. Schweinfurth 1867* (Syntype: if B, then destroyed).
Coccinia adoensis (Hochst. ex A.Rich.) Cogn. var. adoensis Type: South Sudan. [West Bahr al-Ghazal]: Djur realm, Seriba Agad Wau [Waw], fr, May, *G.A. Schweinfurth 1688* (Syntype: B [destroyed], K! [K000542641, digital image: JPS, K], L!).
Coccinia princeae Gilg, Bot. Jahrb. Syst. 34: 358. 1904.
Coccinia adoensis (Hochst. ex A.Rich.) Cogn. var. adoensis Type: Tanzania. Iringa: Uhehe highland, no detailed location given, fr, *M. von Prince s.n.* (Holotype: B, destroyed).
Coccinia adoensis (Hochst. ex A.Rich.) Cogn. var. adoensis Type: Tanzania. Morogoro: Uluguru Mts, NW slopes, male, fl, 19 Jan 1933, *H.J.E. Schlieben 3271* (Neotype, designated here: B!; isoneotypes: G!, HBG!, M! [M-0210964], P! [P05621242, digital image: P], S! [S08-12183]).
Coccinia parvifolia Cogn. in Schinz, Vierteljahresschr. Naturforsch. Ges. Zürich 52: 433. 1907.
Coccinia adoensis (Hochst. ex A.Rich.) Cogn. var. adoensis Type: South Africa. Limpopo: [Mopani District], [Leydsdorp area], Mt Marovounge [Mt Marovougne], male, fl, May 1904, *H.A. Junod 2491* (Holotype: Z! [Z-000004444, digital image: Z], isotype: BR! [BR0000008886811, digital image: BR, JPS]).
Coccinia homblei Cogn., Bull. Jard. Bot. État Bruxelles 5: 114. 1916.
Coccinia adoensis (Hochst. ex A.Rich.) Cogn. var. adoensis Type: D. R. Congo. Katanga: Lualaba region, Kapanda confluence plains, male, fl, Dec 1912, *H. Homblé 992* (Lectotype, designated here: BR! [BR0000008887146, digital image: BR, JPS]; isolectotype: BR! [BR0000008887474, digital image: BR, JPS]).
Coccinia adoensis (Hochst. ex A.Rich.) Cogn. var. adoensis Type: D. R. Congo. Katanga: Kapiri valley, fr, Feb 1919, *H. Homblé 1198* (Syntype: BR! [BR0000008886927, digital image: BR], BR! [BR0000008886859, digital image: BR, JPS]).
Coccinia adoensis (Hochst. ex A.Rich.) Cogn. var. adoensis Type: D. R. Congo. Katanga: Kapiri valley, male, fl, Feb 1919, *H. Homblé 1199* (Syntype: BR! [BR0000008887177, digital image: BR, JPS], BR! [BR0000008887504, digital image: BR, JPS], BR! [BR0000008886842, digital image: BR, JPS, K neg. 4875]).
Coccinia subspicata Cogn., Bull. Jard. Bot. État Bruxelles 5: 115. 1916.
Coccinia adoensis (Hochst. ex A.Rich.) Cogn. var. adoensis Type: D. R. Congo. Katanga: Lualaba region, Kapanda confluence plains, male, fl, Dec 1912, *H. Homblé 992a* (Holotype: BR! [BR0000008887528, digital image: BR, JPS, K neg. 4876]).
Coccinia roseiflora Suess., Proc. Trans. Rhodes. Sci. Ass. 43: 134. 1951.
Coccinia adoensis (Hochst. ex A.Rich.) Cogn. var. adoensis Type: Zimbabwe. [Mashonaland East]: Marandellas [Marondera], Cave Tatooma, 31 Nov 1941, *G. Dehn 188A* (Holotype: M! [M0105778, digital image: JPS]).

#### Description.

Perennial climber or creeper. Stems up to 6 m, glabrous to densely covered with trichomes. Indumentum whitish to beige. Trichomes < 0.5 mm, sometimes curved (Fig. 21). Petioles 0.6–3.5 cm, glabrous, pubescent to tomentose with indumentum like on stem. Leaves 4.2–13.5 × 4.7–16 cm, cordate, shallowly to deeply 3- or 5-lobate, rarely elliptical with hastate base. If profoundly to deeply lobate, then lobes often (slightly) pointing towards apex (as in *Coccinia keayana*; Fig. 32). Lobes triangulate, lanceolate, lineal, elliptical to obovate. Leaf margin entire to serrate (especially on lateral sides) to lobulate. Tips of lobes retuse, obtuse to acute, often with apical tooth. Upper leaf surface clear to white pustulate, rarely with whitish trichomes. Lower leaf surface soft pubescent with short, whitish trichomes, rarely glabrous, often with black to dark brown glands between nerves towards the base, rarely also along main nerves. Probracts missing or obovoid up to 2.5 mm long. Tendrils simple, rarely bifid. Male flowers in few to many-flowered racemes, often accompanied by one solitary flower. Common peduncle 1–11 cm, glabrous or with short trichomes. Pedicel of racemous flower 0.4–1.3 cm, indumentum like peduncle, pedicel of solitary flower 1.5–4 cm glabrous or with short, white trichomes. Bracts missing (caducous?) or up to 2 mm. Perianth tube glabrous or with short (< 0.5 mm), white trichomes. Calyx lobes 1.2–2.5(–3) mm, linear to broadly triangular, adpressed to spreading, apex obtuse to acute. Corolla 0.9–1.6 cm, yellow, salmon-pink, orange, maroon, veins sometimes purplish-brown, lobes 2–5 mm. Filament column whitish to orange, anthers whitish?, yellowish to orange, pollen sacs yellow to orange. Female flowers solitary. Pedicel 0.8–3.5 cm, often glabrous or with short, white trichomes. Hypanthium glabrous or with short (< 0.5 mm), white trichomes. Calyx lobes and corolla like in male flowers. Ovary glabrous. Style and stigmas not seen. Fruits 3–7 × 1–1.5 cm, ovoid, oblong to shortly cylindrical, while ripening (usually?) with dark green longitudinal mottling), ripe orange-red to red, often with sterile tip (“beaked”). Seeds 4–6 × 3–4 × 1–1.7 mm (L/W/H), symmetrically obovate, face lenticular (Fig. 14a, 21).

#### Phenology.

Flowering time: January–May, August–December.

#### Distribution.

**Figure 22.**
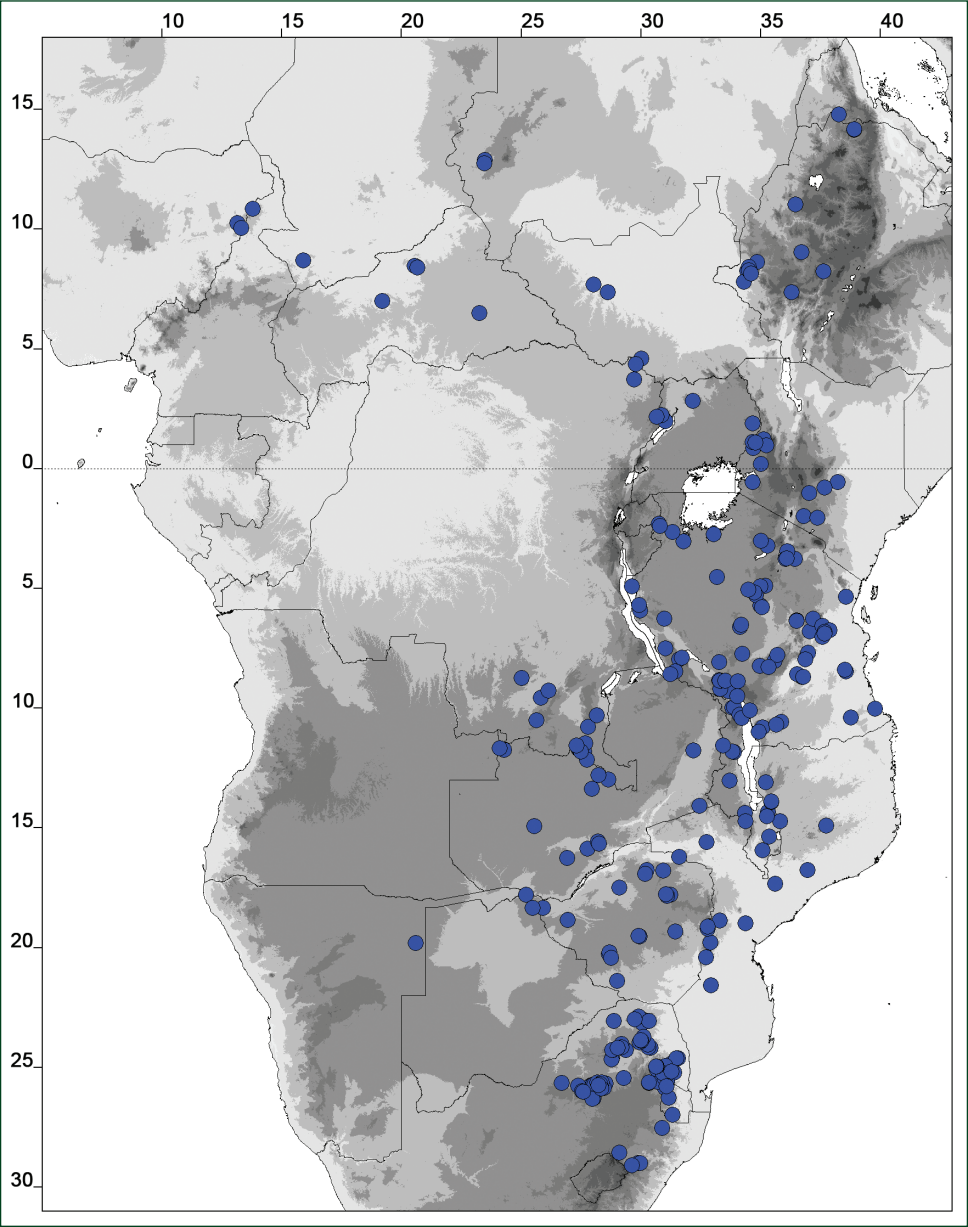
Distribution map of Coccinia adoensis var. adoensis (based on 311 collections).

Fig. 22. Angola? (likely in the South and East since the species occurs close-by), Botswana (North-West District), Burundi?, Cameroon (Extreme North), N Central African Republic, S Chad, Democratic Republic of Congo (Katanga, along the Western Rift), Eritrea (Gash-Barka, likely wider distributed as relicts in the highlands), Ethiopia (except the dry southeast), Kenya (in the west and central highlands), Malawi, Mozambique, N Namibia, Nigeria (only known from Adamawa State, but likely more widely distributed), Rwanda (Eastern Province, maybe wider distributed), South Africa (Gauteng, KwaZulu-Natal, Limpopo, Mpumalanga, E North West, E Free State), South Sudan, Sudan (West Darfur, maybe also in woodland relict sites in other provinces), Swaziland, Tanzania, Uganda (Northern Region), Zambia, Zimbabwe. Elevation 130–3450 m. On sandy and silty soils, clay loam, loam, laterite, syenite soils, dolerite soil, dolomite soil, limestone. *Hyparrhenia cymbaria* savanna; *Crossopteryx* tree savanna; *Acacia*-*Combretum*-*Stereospermum*-*Cussonia* woodland; *Pseuodoprosopis fischeri* woodland, *Anogeissus leiocarpus* woodland, *Brachystegia* woodland, sourveld grassland, *Melhania rehmannii*-*Enneapogon scoparius* mixed bushveld (Siebert et al. 2010).

#### Use.

Roots are boiled and drunk for fever (*J.C. Lovett & C.J. Kayombo 3434*). The potato-like tubers are eaten (*F.W. Andrews 1310*), also raw (*T. Scudder 56*). The greens are used as spinach, among others by the Venda (*N.J. van Warmelo s.n. Mar 1960*, *J. Gerstner 5838*) and also eaten by the Luo (Johns and Kokwaro 1991). Ripe fruits are edible (*J.C. Lovett et al. 3842*, *J. Pawek 11008*, *T. Scudder 56*). According to de Boer et al. (2005), the Pare people in Tanzania use an infusion of leaves and stems for abortions, uterus cleansing, and against chickenpox. *Coccinia adoensis* is quite variable and so there are likely many varieties, of which some might not be edible. Rehm et al. (1957) reports the cytotoxic cucurbitacin B and traces of cucurbitacin D in immature fruits, but edible ripe fruits are known from South Africa. Gradé et al. (2009) report that the usability or toxicity of the tuberous roots is disputed, suggesting chemical variability.

#### Vernacular names.

Bokora tribe [Karamojong?]: edaldalakisin (Gradé et al. 2009); Kiluo: mutkuru (Johns and Kokwaro 1991), nyatunduguwoge (*J.G.B. Newbould 5745*); Kipare: mlushi (de Boer et al. 2005); Kisafwa: tandandala (*J.C. Lovett & C.J. Kayombo 3434*), ndandala (*J.C. Lovett & C.J. Kayombo 3773*); Kisagara [Kisagala]: lutetere (*F. Haerdi 563/0*); Nhungoé [Cinyungwe]: mugwingwi (*L. Macuácua 1441*); Sotho [most likely Northern Sotho]: sephu (*J. Gerstner 5838*); Tigrinya: entatakh (fide G.H.W. Schimper (Schweinfurth 1893)), entota (fide A. Richard (Schweinfurth 1893)); Tshivenda: tshiphu (*N.J. van Warmelo s.n. Mar 1960*); Zande: bawiriokoro (*F.W. Andrews 1601*).

#### Remarks.

*Coccinia adoensis* is widespread and morphologically variable. Some populations or local forms appear to be distinct, but there are intermediate individuals or similar-looking collections from different parts of the overall distribution range. In East Africa (C Tanzania, Malawi), one can find forms linking to *Coccinia aurantiaca*, which is treated here as variety of *Coccinia adoensis*, and to *Coccinia senensis*. The latter forms have a similar plastid haplotype with *Coccinia senensis*, but lack a specific deletion in the *trn*S^GCU^–*trn*G^UCC^ intergenic spacer. These forms from central and southern Tanzania are discussed here under the name Coccinia adoensis var. jeffreyana, while another form from Kenya remains in var. adoensis. The non-monophyly in the plastid tree (Holstein and Renner 2011b) makes *Coccinia adoensis* even more peculiar. The scenario given in the chapter Evolution and phylogeny might explain this pattern, but without phylogeographic analysis and crossing experiments, this will remain speculative.

Some specimens from the Kingupira area (Lindi, Tanzania; *K. Vollesen 3182*, *3212*, *3384*, *4320*) have an unusual morphology by having veins that run along the leaf margin, which is unique in *Coccinia*. Except for this character, they match Coccinia adoensis var. adoensis well (sympetalous, obovate probracts). They strongly resemble *Eureiandra* species in vegetative characters, but not in generative traits.

#### Taxonomic remarks.

The lectotypification of *Momordica adoensis* by Meeuse (1962) is not effected, as he did not specify which specimen was supposed to be the lectotype. However, the present author follows his suggestion and chose among the two *Schimper 166* specimens from P.

The types of *Bryonia jatrophaefolia* are not too obvious as such. The protologue states “Tchélatchekanné”, but Paris Herbarium holds two Quartin-Dillon and Petit specimens (P00346260 and a non-barcoded one) with a location “Tchessu Heckequenné”. Although the spelling has some similarities, they are quite different. On the other hand, the Ge’ez letter sat [S] and läwe [L] look similar and might have been mistranscribed if the name was written down in Ge’ez script. Additionally, the two specimens bear the species name in Richard’s handwriting (C. Bräuchler, pers. comm.). Gillett (1972) notes that Achille Richard, the author of the botanical treatment of Quartin-Dillon's and Petit's journey, wrote consistently “Tchelatchekanné”, although Schimper calls it “Djeladjeranné”. However, there is a locality with that name [Tchelatchekenneh at c. 13°43'N, 38°22'E] in Johnston’s atlas (Johnston 1861), to which Gillett (1972) refers.

The *G.A. Schweinfurth 1668* specimen in L is not obviously a type specimen as it lacks the original label. However, the Herb. D’Alleizette label mentions “*Coccinia djurensis*”, and the location is the same as the K duplicate with the original label. Additionally, the specimen is a fruiting female, like the specimen in K. Hence, d’Alleizette must have obtained a duplicate of the type.

The placement of *Coccinia hartmanniana* as a synonym of *Coccinia adoensis* is done with a high level of confidence although no type specimens were found. The protologue contains drawings showing lenticular seeds and short calyx lobes, which match well other collections of the *Coccinia adoensis* complex. According to Ascherson (in Schweinfurth 1867), von Harnier’s collections consisted of two duplicates, one of them in B. Other duplicates of von Harnier are in BM and K but not this collection apparently. Since no type specimen was seen, despite extensive search, a drawing from the protologue was chosen as lectotype.

For *Coccinia princeae*, a neotype was selected because the holotype was destroyed. The leaves of the chosen specimen, *H.J.E. Schlieben 3271*, match the description well, and the specimens have been identified as *Coccinia princeae* when the original material was still existing. The specimens differ in the generative characters (fruiting in the holotype, male flowers in the neotype), but Gilg referred strongly to the distinctive leaves, so the neotype appears to be a good match.

The holotype of *Coccinia roseiflora* is the drawing in M, which has the number 188a. The protologue states: “descriptio sec. tabulam cl. Dehniae” (described following/based on the illustration of Ms. Dehn). There are (at least) two specimens with the number 188 (K, SRGH), which partially might have served as basis for the drawing, but they are not types. The drawing contains all necessary characters to synonymize it with certainty with *Coccinia adoensis*: seed shape, fruit maculation and calyx lobe morphology.

#### Specimens examined.

(Selection, in total: 483) Botswana. North-West District: Ngamiland, Motantanyane, *H.H. Curson 784* (M). Central African Republic. Nana-Grébizi: Gribingui (Ft-Crampel) [=Kaga-Bandoro], *A.J.B. Chevalier 6325* (P [P05621208]). D. R. Congo. Orientale: Faradje (Kibali-Ituri), *J. Lebrun 3406* (BR, WAG [WAG0225534]). Eritrea. Gash-Barka: Seraé [Seraè, a former province], in Tucul region, *A. de Benedictis 519* (FT). Ethiopia. Tigray: Edaga Sciaba, *E. Chiovenda 581* (FT). Malawi. Northern Region: Karonga district, 17 mls [27.2 km] N of Chilumba, *J. Pawek 11008* (DSM, MO, PRE, WAG [WAG0234129]). Mozambique. Zambézia: Massingire [Morrumbala district], M’bôbo [river at Morrumbala], on road to Mopeia, *A.R. Torre 5337* (M). Nigeria. Adamawa: 10 miles [16 km] from Mubi to Toyola, *P. Wit et al. 1797* (BR, MO (2), P [P05621253], WAG [WAG0041392], WAG [WAG0041393]). Rwanda. Eastern Province: Kibungo prefecture, Rusumo [Ruzumo Falls], savanna park, on opposite slope of A.I.D.R. [Association internationale de développement rural] camp, *J. Lambinon 74/1568* (M, MO, WAG [WAG0225516]). South Africa. Gauteng: [City of Johannesburg], N of Eikenhof, Johannesburg, Walkerville rd., *L.E. Davidson 3781* (B, M). Sudan. West Darfur: Zalingei, *G.E. Wickens 1800* (K). Tanzania. Lindi: Selous Game Reserve, Kingupira, 8°28'S, 38°33'E, [38°34'E], *K. Vollesen MRC 3384* (DSM, EA, WAG [WAG0234135]). Ruvuma: Songea, *E. Milne-Redhead & P. Taylor 7876* (EA); ibid., *E. Milne-Redhead & P. Taylor 7950* (B, EA, K, P [P05621243], S [S08-11819]). Zambia. Lusaka: c. 10 km S of Chilanga and 23 km S of Lusaka, c. 1 km N of Kafue Road near Chilanga Cement housing; Shimabala Cave, 15°39'01"S, 28°14'15"E, *D.K. Harder & M.G. Bingham 2584* (K, MO). Zimbabwe. Matabeleland North: Matetsi Safari Area Headquarters House no. 2, *P. Gonde 256* (E, G, MO, P [P05621269]).

### 2b. Coccinia adoensis var. aurantiaca

Taxon classificationPlantaeCucurbitalesCucurbitaceae

(C.Jeffrey) Holstein stat. nov.

urn:lsid:ipni.org:names:77148917-1

Coccinia aurantiaca C.Jeffrey, Kew Bull. 17: 169. 1963.
Coccinia adoensis var. aurantiaca Type: Tanzania. Dodoma: Kondoa District, Great North road, 15 miles S of Kondoa, 1310 m, fl, fr, 19 Jan 1962, *R. Polhill & S. Paulo 1221* (Holotype: K! [K000313236, digital image: JPS, K], isotypes: B! [B 10 0154922, digital image: B, JPS], B! [B 10 0154923, digital image: B, JPS], BR! [BR0000008887252, digital image: BR, JPS], EA (2)!, PRE! [PRE0592951-1, digital image: JPS], PRE! [PRE0592951-2, digital image: JPS]).
Coccinia adoensis var. aurantiaca Type: Tanzania. Dodoma: 1 ml. [1.6 km] S of Dodoma, Imagi hill, *R. Polhill & S. Paulo 1274* (Paratypes: B! [B 10 0154921, digital image: B, JPS], BR!, EA!, K (3)!, P! p.p.,
Coccinia adoensis var. aurantiaca PRE!, S! [S08-11841, digital image: S]).
Coccinia adoensis var. aurantiaca Type: Tanzania. Mwanza: Mwanza, Ilemera, Butimba, *R.E.S. Tanner 1902* (Paratypes: BR!, EA!, K (2)!).
Coccinia adoensis var. aurantiaca Type: Tanzania. Mwanza: Mbarika [chiefdom], Buzomo, *R.E.S. Tanner 1068* (Paratypes: BR!, COI (2)!, EA!, K!, NY!).
Coccinia adoensis var. aurantiaca Type: Tanzania. Mwanza: Mwanza, *R.E.S. Tanner 646* (Paratype: K!).
Coccinia adoensis var. aurantiaca Type: Tanzania. Shinyanga: near Shinyanga, *R.D. Bax 57* (Paratypes: K (2)!).
Coccinia adoensis var. aurantiaca Type: Tanzania. Shinyanga: hills near Shinyanga, *B.D. Burtt 2517* (Paratype: K!).
Coccinia adoensis var. aurantiaca Type: Tanzania. Shinyanga: Shinyanga, *H. Koritschoner 1823* (Paratypes: EA (2)!, K!).

#### Description.

Perennial climber. Stems up to 10 m, almost tomentose with short (< 0.5 mm), stiff, whitish trichomes. Petiole 1.5–3.5 cm, indumentum similar to stems. Leaves 5.2–12.5 × 6.4–14.5 cm, cordate, shallowly to profoundly 3- or 5-lobate. Lobes triangulate, ovate, elliptical to obovate. Margin serrate to lobulate. Apex obtuse, rarely acute, with final tooth. Upper leaf surface white-pustulate sometimes more or less pubescent with short, whitish trichomes. Lower leaf surface usually densely covered with curved or short, straight trichomes on nerves. Probracts up to 1.5 mm, often caducous. Tendrils simple. Male flowers solitary or in racemes. Common peduncle 0.4–3 cm, pedicels in racemes up to 0.5 cm, pedicel of solitary flowers 1–2 cm, indumentum as on stem. Bracts up to 1.5 mm, persisting. Perianth tube densely covered with short (< 0.5 mm) trichomes. Calyx lobes 1–3 mm, narrow triangular to dentate, spreading. Corolla 1.6–2.4 cm long, pale yellow-brown to orange, rarely? yellow, with green to orange venation, lobes 0.6–1 cm. Filament column, anther head, and pollen sacs more or less pale orange, rarely yellowish? (Fig. 10b). Female flowers solitary, pedicel 1.2–4 cm, indumentum as on stems. Hypanthium densely covered with short (< 0.5 mm) trichomes, calyx lobes, and corolla as in male flowers. Style shape not seen, green. Stigma shape not seen, yellow to orange. Ovary with short trichomes. Unripe fruits pale green with irregular lighter spots and dark green longitudinal lines. Fruits 5–9 × 1.5–3.5 cm, long ovoid, apex sometimes beaked, when ripe orange-red. Seeds 6–6.5 × 3.5–4 × 1.5–1.7 mm [L/W/H], slightly asymmetrically obovate, face flatly lenticular.

#### Phenology.

Flowering time: January, March, July, October, December.

#### Distribution.

**Figure 23.**
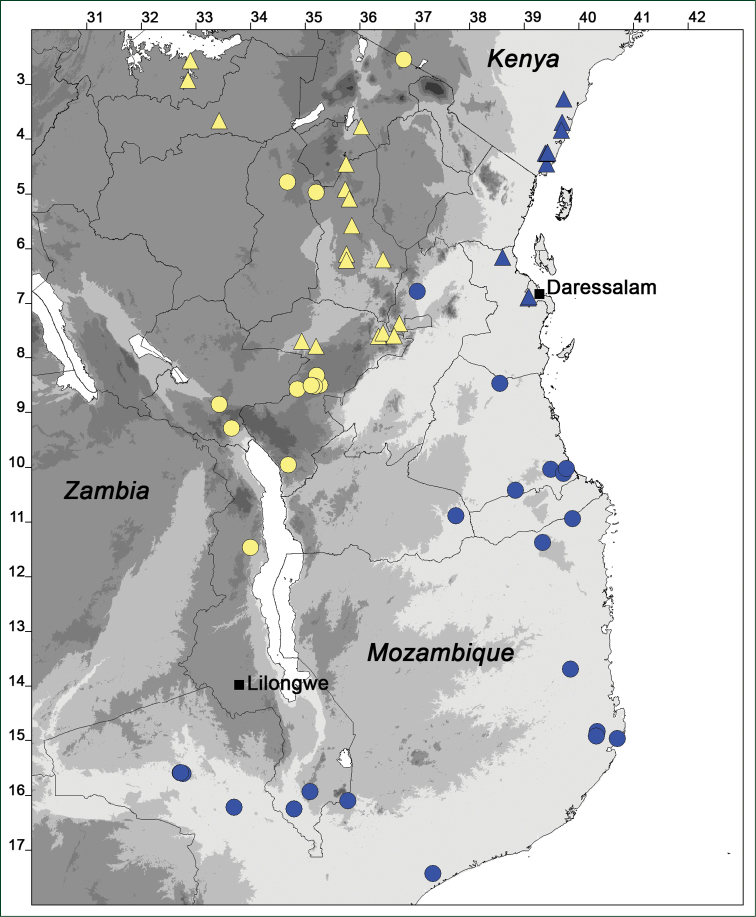
Distribution map of Coccinia adoensis var. aurantiaca (pale yellow triangles; based on 18 collections), Coccinia adoensis var. jeffreyana (pale yellow dots; based on 18 collections), *Coccinia pwaniensis* (blue triangles; based on 11 collections, including a natural hybrid), and *Coccinia senensis* (blue dots; based on 30 collections). For Tanzania the borders of the regions are given.

Fig. 23. Tanzania (Dodoma, Iringa, Manyara, Morogoro, Mwanza, Shinyanga). Elevation 600–1200 m. Red sandy soil, red clay, granite. White clay. Gray sand. Brown sandy loam. Dry Miombo woodland, *Acacia tanganyikensis*-Acacia tortilis subsp. spirocarpa-*Adansonia digitata*-*Maerua crassifolia*-*Balanites aegyptiaca* woodland, long grass savannas, dry (*Commiphora*-*Acacia*) bushland, thickets (e.g., *Combretum* thickets), among rocks on hills.

#### Use.

Leaves are boiled and eaten (*J.L. Newman 62*). Fruits edible when ripe and dry (*E.S. Macha 600*).

#### Vernacular names.

Sandawe language: koba (*J.L. Newman 62*).

#### Remarks.

The status of this taxon as species is unclear, therefore it is treated as a variety of the polymorphic *Coccinia adoensis*. Coccinia adoensis var. aurantiaca specimens as listed here are usually more densely covered with trichomes than Coccinia adoensis var. adoensis. Jeffrey segregated this species from the polymorphic *Coccinia adoensis* because of the non-beaked fruits and flat seeds with a hyaline girdle. The beak is a sterile part of the ovary with variable length, but it does not occur in all populations. Two of the paratypes (*R. Polhill & S. Paulo 1274* (BR, P)), which match other Coccinia adoensis var. aurantiaca collections vegetatively, have a slightly beaked fruit, although most other collections do not. The seeds are also hardly distinct from *Coccinia adoensis*, perhaps somewhat larger. Seeds in *Coccinia* are enclosed in a hyaline aril. Jeffrey only observed the dry collapsed aril, which is not part of the seed, as a “hyaline girdle”. The orange color of the petals, even with purple venation also occurs in individuals of Coccinia adoensis var. adoensis that have a less dense indumentum. The corolla is thus not a good distinguishing character either. However, this variety seems to occur in a drier part of the range of the overall *Coccinia adoensis* distribution (Holstein and Renner 2011b).

#### Taxonomic remarks.

The flowers in the *R. Polhill & S. Paulo 1274* specimen in P do not belong to *Coccinia*. The calyx appears to be *Momordica foetida* Schum. & Thonn. The HEID specimen (HEID779579) of that collection is also mistaken, eventually a mix-up while mounting the specimen. It has a completely different indumentum and a narrow, almost cylindrical perianth tube.

#### Specimens examined.

(Selection, in total: 29) Tanzania. Dodoma: Dodoma–Kondoa road, c. 20 km S of Kondoa, 05°16'31.5"S, 35°53'01.1"E, *N. Holstein et al. 85* (DSM, M), and *86* (M). Iringa: Iringa Rural District, along road Iringa–Morogoro road and Lukosi River, at bottom of Kitonga Gorge, c. 6 km W of Mahenge village at milepost 253 km from Morogoro, 7°38'S, 36°14'E, [7°34'S, 36°19'E], *C.M. Taylor et al. 8485* (K, MO); [Ruaha National Park], Msembi [near airfield], *P.J. Greenway & K. Kanuri 14811* (EA (2), K, M). Manyara: Tarangire National Park, road Tarangire camp–Babati, 1 ml. from camp, *H.M. Richards 24817* (EA, K). Morogoro: Kilosa district, Elphon’s Pass, 7°22'S, 36°42'E, *J.C. Lovett & T.C.E. Congdon 2931* (K, MO).

### 2c. Coccinia adoensis var. jeffreyana

Taxon classificationPlantaeCucurbitalesCucurbitaceae

Holstein var. nov.

urn:lsid:ipni.org:names:77148914-1

Coccinia adoensis var. jeffreyana Type: Tanzania. Iringa, Mufindi District, Ngwazi, 8°30'S, 35°15'E, 1830 m, female, fl, fr, 25 Feb 1987, *J.C. Lovett 1597* (Holotype: MO!, isotype: EA!).

#### Diagnosis.

This variety has affinities with *Coccinia adoensis* and *Coccinia senensis*. The abaxial side of the petiole and the lower leaf surface bears simple trichomes with long cells, which appear crumpled or articulate when dry. Most of the trichomes, especially on the nodes, exceed 0.8 mm (–1.2 mm), whereas trichomes of Coccinia adoensis var. adoensis and var. aurantiaca are shorter < 0.5(–0.8) mm. The calyx lobe length often exceeds 2 mm (in contrast to other *Coccinia adoensis* varieties), but the lobes are not subulate or narrowly acute as in *Coccinia senensis* but rather linear or if narrowly triangulate, then not with a pointed tip.

#### Description.

Perennial creeper or climber. Stems up to 3 m, more or less densely covered with long (at least on the nodes > 0.8 mm, Figs 3a, 5c) trichomes that appear articulate when dry. Petiole 0.25–3.5 cm, subsessile to distinctly petiolate, with long patent trichomes. Leaves 3.2–10.5 × 2.6–12 cm, shallowly to profoundly 3- or 5-lobate, lobes triangular, ovate to elliptical, margin dentate, slightly serrate, apex acute to obtuse with apical tip. Upper leaf surface glabrous or with few trichomes, hyaline to white pustulate. Lower leaf surface more or less densely covered with articulate trichomes, rarely almost glabrous with white pustules on veins. Probracts up to 3 mm. Tendrils simple. Male flowers in racemes, often accompanied by a single flower or one solitary flower. Common peduncle 5–5.5 cm, with short articulate trichomes. Pedicel of racemous flowers 5–9 mm, with short articulate trichomes. Bracts up to 1 mm, caducous. Pedicel of solitary flower 2.2–7.8 cm, with short articulate trichomes. Hypanthium with short trichomes. Calyx lobes 1–3.5 mm, narrowly triangular but not subulate, erect. Corolla 1.1–1.65 cm, yellow, orange, to dark crimson with darker veins outside, lobes 4–7 mm. Color of filament column pink, anther head orange-yellow to orange, color of pollen sacs not seen. Female flowers solitary. Pedicel 0.6–1.7 cm long, puberulous. Ovary with short to long, articulate trichomes. Fruit size c. 2–6 × c. 1 cm long, elliptical, often with sterile apical tip (“beaked”), glabrous, green with white spots when unripe, red when ripe. Seeds 4–5.5 × 3–3.5 × 1.5 mm (L/W/H), symmetrically obovate, face lenticular (Fig. 14a).

#### Phenology.

Flowering time: January–March, November, December.

#### Distribution.

Fig. 23. Malawi (Northern Region, Southern Region), Tanzania (Dodoma?, Iringa, Mbeya, Morogoro?, Singida), Kenya (southern Rift Valley Province). 1300–2600 m. Soil preferences unknown. With *Dodonea viscosa*; under pines; in *Eucalyptus* plantation, highland grassland, in open woodland with *Combretum* sp., *Grewia* sp., *Strophanthus emenii*, *Acacia tortilis*, *Tapiphyllum obtusifolium*, *Burttia* sp., *Cassia* sp.

#### Etymology.

The epithet was chosen to honor Charles Jeffrey, who worked extensively on the Cucurbitaceae and the flora of East Africa.

#### Use.

Unripe and ripe fruits are reported to be edible (*C.J. Kayombo 296*, *P. Kuchar 22631*), roots taken to make stomach medicine (*P. Kuchar 22631*).

#### Vernacular names.

Kihehe: mtumbulansoka (*W. Carmichael 171*); Kinyaturu: mukunguhi (*P. Kuchar 22631*).

#### Remarks.

Morphologically, this variety closely matches *Coccinia senensis* (with rather short petiolate to subsessile leaves, and a *Coccinia senensis*-like indumentum), but it has the calyx lobes rather of Coccinia adoensis var. adoensis, with the lobe length being intermediate between *Coccinia senensis* and Coccinia adoensis var. adoensis. The sequenced specimens do not cluster with most other *Coccinia adoensis* haplotypes from East Africa or southern Africa, and lack the typical deletion of *Coccinia senensis* in the *trn*S^GCU^–*trn*G^UCC^ intergenic spacer (Holstein and Renner 2011b). A Coccinia adoensis var. adoensis-like collection (*S.A. Robertson 1925*) also clusters with this variety, but it lacks the long trichomes. Long trichomes also appear in populations of *Coccinia grandiflora* or *Coccinia mackenii* in higher altitudes or in areas with higher precipitation. The collections of this variety are distributed above 1300 m and thus receive higher amounts of rainfall, so the long trichomes could be an adaptation. On the other hand, very similar trichomes regularly occur in *Coccinia senensis*, sometimes short though, but that species does not occur in such high altitudes. As the collections of this variety differ from the “typical” *Coccinia adoensis*, but still belong to *Coccinia adoensis*, they are treated as a new variety.

The collection *R.E. Gereau & C.J. Kayombo 3582* (K, MO; *Coccinia adoensis* 4 in Fig. 17) is morphologically inseparable from this variety, and the plastid haplotype clusters within East African *Coccinia adoensis*. This collection has a normal-sized corolla, and therefore seems to be fertile, which supports the hypothesis that the var. jeffreyana is not reproductively isolated from var. adoensis. This is also why the present author refrains from designating it as a paratype, namely in order to avoid confusion about the genetic definition of this variety.

Phylogenetically, it is uncertain whether this variety retains an ancestral morphology of the common ancestor of Coccinia adoensis var. adoensis and *Coccinia senensis* or whether the longer trichomes are homoplastic due to an adaptive nature or this is a case of incomplete lineage sorting. Given the strong impact of aridification caused by the ice ages, the ancestor of *Coccinia adoensis* and *Coccinia senensis* presumably survived during an arid era in more humid coastal “forests” and woodlands of East Africa, where it evolved to *Coccinia senensis* and *Coccinia pwaniensis*. Other morphs evolved in woodlands rather in the inland, and are now pooled as *Coccinia adoensis*. Interestingly, the distribution of Coccinia adoensis var. jeffreyana, *Coccinia senensis*, and the allied *Coccinia pwaniensis* (shares the subulate calyx lobes with *Coccinia senensis*) is very similar to that of the Apocynaceae species *Carvalhoa campanulata* K.Schum. (Leeuwenberg 1985), which suggests shared ecological preferences.

The collections from Singida occur in drier habitats than those from C and S Tanzania. Collections with an indumentum like Coccinia adoensis var. jeffreyana also occur in NE D. R. Congo (*A. Taton 128*, *G. Troupin 570*), but it is uncertain whether these are also genetically linked to Coccinia adoensis var. jeffreyana, so they are listed here under Coccinia adoensis var. adoensis.

#### Specimens examined.

(selection, in total: 26) Kenya. Rift Valley Province: Namanga, cultivated in Munich Botanical Garden, *N. Holstein 125* (M) and *130* (M). Malawi. Northern Region: Mzimba Dist., 3 mi. W of Mzuzu at Katoto, *J. Pawek 10404* (MO, WAG [WAG0234128]). Tanzania. Iringa: Ludewa district. Livingstone Mountains, E slope of Msalaba Mountain, above stand of *Acacia abyssinica* on foot trail from mission at Luana, 09°59'S 34°36'E, *R.E. Gereau & C.J. Kayombo 3535* (DSM, EA, MO, NHT, PRE); Great North Road, Sao Hill, 61ml. S of Iringa, *R. Polhill & S. Paulo 1722* (B, EA, P [P05621244], PRE). Mbeya: Nyassa Hochland, Station Kyimbila, *A.F. Stolz 504* (JE, M, U, W). Singida: 8½ km along road from Singida to Sepuka, 04°46'35"S 034°40'00"E, *P. Kuchar 23919* (MO, S [S08-12129]).

### 3. Coccinia barteri

Taxon classificationPlantaeCucurbitalesCucurbitaceae

(Hook.f.) Keay, Kew Bull. 8: 82. 1953.

Staphylosyce barteri Hook.f. in Oliv., Fl. trop. Afr. 2: 554. 1871. *Physedra barteri* (Hook.f.) Cogn. in A.DC. & C.DC., Monogr. Phan. 3: 525. 1881.
Coccinia barteri Type: Nigeria. Nupe [Niger State]: exact locality not specified, male, fl, *C. Barter 1525* (Lectotype, designated by Jeffrey (1967: 60): K!, digital image! [K]).
Coccinia barteri Type: Equatorial Guinea. [Fernando Po] Bioko Island, female, *C. Barter no.* ? (Syntype: K?), see Taxonomic remarks.
Coccinia macrocarpa Cogn., Bull. Jard. Bot. État Brux. 5: 113. 1915–1919. Pro parte [except *E. Luja 125*].
Coccinia barteri Type: D. R. Congo. Sankuru river [tributary of Kasai river], no detailed location given, on farmland and in bushland, male, fl, Jul 1904, *E. Luja 205* (Lectotype, designated by Jeffrey (1967: 61): BR! [BR0000008888570, digital image: BR, JPS, photo: K!).
Coccinia barteri Type: Sankuru river [tributary of Kasai river], no detailed location given, female, fr, Nov 1903, *E. Luja 125* (Syntype: BR! [BR0000008888228, digital image: BR, JPS]).
Coccinia subhastata Keraudren, Fl. Cam. 6: 131. 1967.
Coccinia barteri Type: Cameroon. South Region: Bitye, male, fl, 1917, *G.L. Bates 1469* (Holotype: BM!).

#### Description.

Perennial climber. Stems up to 10 m, glabrous or puberulous. Petioles 1–3.5(–8.5) cm, glabrous to puberulous, adaxial side rarely with trichomes. Leaves 3.5–20 × 4–23 cm, cordate, subhastate, shallowly to deeply 3- or 5-lobate. Lobes triangulate, ovate to oblong. Margin entire with few to many teeth to serrate. Apex obtuse to acute, with apical tooth. Upper leaf surface glabrous with clear or white pustules, lower leaf surface glabrous to puberulous on main nerves, esp. towards base, with or without small dark glands. Probracts ovate to elliptical, up to 5 mm long or missing. Tendrils simple or bifid. Male flowers in few- to many-flowered racemes. Common peduncle up to 3–8 mm long, glabrous to puberulous. Pedicel < 8 mm, indumentum like peduncle. Flowers without or with up to 1.5 mm long bracts. Perianth tube glabrous to puberulous. Calyx lobes 1–2.5 mm, subulate, lineal, rarely somewhat lanceolate, reflexed, spreading or erect and adpressed to corolla, sometimes seemingly fleshy. Corolla 1.1–2.4 cm, salmon, yellow to orange-yellow, lobes up to 3–10 mm. Filament column, anther head, and pollen sac color not seen. Female flowers in racemes, sometimes accompanied with a solitary flower or one solitary flower only. Peduncles and petioles in racemes like in males. Solitary female flowers with up to 1.5 cm long glabrous to puberulous pedicel. Ovary glabrous. Hypanthium glabrous to puberulous, calyx lobes and corolla as in males. Style not seen. Stigma shape not seen, more or less dark yellow. Fruit 1.5–2.5 × 1.5 cm, shortly elliptical to subglobose, unripe green with pale spots, ripe red. Seeds 5.5 × 2.5–3 × 1–1.5 mm (L/W/H), more or less symmetrically obovate, face flat to flatly lenticular.

#### Phenology.

Flowering time: January–June, August–November.

#### Distribution.

**Figure 24.**
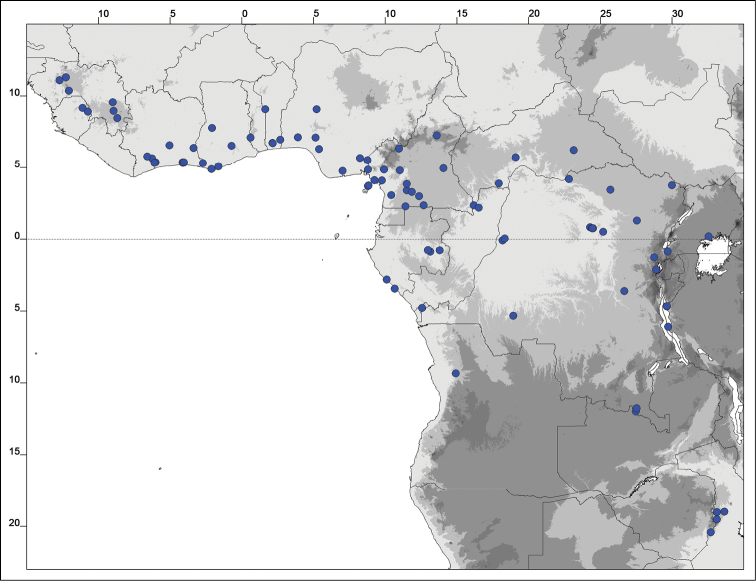
Distribution map of *Coccinia barteri* (based on 99 collections).

Fig. 24. Humid tropical West Africa, Angola (Cabinda, Cuanza Norte), Burundi?, C and S Cameroon, C and S Central African Republic, D. R. Congo, R. Congo, Equatorial Guinea, Gabon, Mozambique (Manica), S South Sudan?, Uganda (Western, Central), Rwanda?, W Tanzania, Zambia (Northern Province), Zimbabwe (Manicaland). Elevation from sea level to 1650 m. Soil preference not well known, on loam soil, on granite (*J.B. Gillett 15298*). (*Newtonia*) rainforest; forest margins; near open water with *Pandanus candelabrum*, *Oxystigma mannii*, and *Raphia vinifera*; near river with *Saba comorensis*; on border of gallery forest and *Terminalia glaucescens* woodland; in riverine bushes, on river islands with *Alchornea cordifolia*; in fallows.

#### Use.

The Turumbu people mash young leaves, mixed with white argil, and put the paste onto the heads of ill children (*W. Kesler 1034*).

#### Vernacular names.

Lissongo [Mbati]: makpo (*C. Tisserant (Équipe) 2250*); Twi: isamaŋ kyẽkyẽa (*F.R. Irvine 2604*); Turumbu: eliki e litoko (*J. Louis 2253*), ndombo di ilo (*W. Kesler 1034*)

#### Remarks.

*Coccinia barteri* is treated here in a wide sense as it contains several forms (see also Holstein and Renner 2011b). This is because data on these forms are scarce and do not unambiguously allow to refer to these as species. Therefore, the present author refrains from creating an intraspecific classification as a phylogeographic treatment and crossing experiments appear to be necessary to clarify this problematic taxon.

There are collections in Gabon that are of intermediate morphology between *Coccinia barteri* and *Coccinia racemiflora* (*M.A. van Bergen 490* (WAG) = *Coccinia barteri* 6 in Fig. 17). Holstein and Renner (2011b) suggested that hybridization occurs between these species. Whether the hybrids are fertile or sterile is not known.

#### Taxonomic remarks.

*Coccinia barteri* (Hook.f.) Keay is type species of the genus *Staphylosyce* Hook.f.

Joseph Dalton Hooker mentions collections from Fernando Po [Bioko Island] and Nupe in the protologue of *Coccinia barteri*. He only gives the name of Barter, whose Nupe specimen is in K, but there are no *Coccinia* specimens by Barter from Fernando Po. However, there are two specimens collected by G. Mann (*Mann N199*! and *N1166*!) in Hooker’s herbarium (now in K). These were collected on that island, and they contain drawings that were most likely the basis for Hooker’s description of *Staphylosyce barteri*. Possibly, Hooker mistakenly left out Mann’s name in the protologue, whose collections contain many type specimens (Hooker 1871).

Keay published in error *Coccinea barteri* [*sic*] in his new combination, but accepted the species as belonging to *Coccinia* in Hutchinson and Dalziel’s Flora of Tropical Africa (1954).

The syntypes of *Coccinia macrocarpa* certainly belong to different taxa. The present author concurs with Kéraudren, who placed the male specimen *É. Luja 205* into the polymorphic *Coccinia barteri* (1967). However, the female plant *É. Luja 125* is clearly not part of *Coccinia*. *Coccinia* seeds are up to 7 mm long, at the base attenuate to truncate and with a rounded apex. In contrast, the seeds of *É. Luja 125* are subquadratic as Jeffrey already pointed out on the type specimen. A placement in *Momordica* by Jeffrey (on the sheet) seems to be correct, whether this is *Momordica multiflora* Hook.f. (1871) as identified by Jeffrey or *Momordica parvifolia* Cogn. (1916) as identified by Kéraudren is beyond the present author's knowledge.

*Coccinia subhastata* was described under the assumption that *Coccinia barteri* has long calyx lobes, as it can be seen in Flore du Cameroun (Kéraudren 1967). Monique Kéraudren in her research on western Central African Cucurbitaceae (Kéraudren-Aymonin 1975a; Kéraudren 1967) treated *Coccinia*/*Physedra barteri* and *Physedra heterophylla* as synonymous. However, she confused the long subulate calyx lobes of *Physedra heterophylla* as a character for *Coccinia barteri*, describing a specimen with short calyx lobes and flowers in long racemes as a new species, *Coccinia subhastata* Keraudren. She also described several differences of *Coccinia subhastata* to *Coccinia barteri*, which are not supported when carefully examined. *Coccinia subhastata* should only have simple tendrils, but the holotype of *Coccinia subhastata* also has a bifid tendril. Furthermore, the *Coccinia barteri* lectotype *C. Barter 1525* has a subhastate leaf and simple tendrils. The description of *Coccinia subhastata* thus is thus wrong and the species is a synonym of *Coccinia barteri*, as it has been pointed out by Holstein and Renner (2010). In addition to the confusion of *Coccinia heterophylla* and *Coccinia barteri*, Kéraudren separated the western Central African specimens with few-flowered racemes as *Coccinia keayana* R.Fern. (Kéraudren 1967). *Coccinia keayana*, however, does in fact not occur in this region but only in West Africa, and her *Coccinia keayana* specimens from Cameroon belong to the polymorphic *Coccinia barteri*.

#### Specimens examined.

(Selection, in total: 153) Angola. Cuanza Norte: Cazengo municipality, near Agricultural Station Cazengo, *J. Gossweiler 5492* (COI, LISU), and *5507* (LISU). Benin. Atlantique: Allada commune, Dahounkpa (Niaouli), 6°44'N, 2°07'E, *A. Akoègninou & F. Bada 2992* (WAG [WAG0314946]). Cameroon. South Region: Bipinde [Bipindi], *G.A. Zenker 1657* (E, G (4), HBG, P [P05621189], P [P05621192], W, Z). Central African Republic. Lobaye: Boukoko, *C. Tisserant (Équipe) 2176* (BM, P [P05620598], P [P05621175]). D. R. Congo. Katanga: [Haut Katanga district], 40 km on road from Lubumbashi to Sakanja, *A. Schmitz 4465* (EA (2), WAG [WAG0225507], WAG [WAG0225508]). Equatorial Guinea. Bioko Norte: Malabo–Punta Hermosa km 9, 32NMK8114, *F.J. Fernández Casas 12077* (BM, MA n.v., MO, WAG [WAG0069018]). Gabon. Haut-Ogooué: 21 km on road from Okonja to Akiéni, 0°45.84'S, 13°47.01'E, *J.J. Wieringa et al. 6387* (M, WAG [WAG0250956], WAG [WAG0250957], WAG [WAG0250958]). Ghana. Volta: Agumatsa Wildlife Sanctuary, at town Wli-Agorviefe, W of Park Guard HQ, 7°06'46"N, 0°35'25"E, *H.H. Schmidt et al. 2192* (K, MO). Guinea. Nzérékoré: [Beyla préfecture], Bola [Boola], Famondou [Famodougou], 8.48068°N 8.70129°W, *E. Achigan-Dako 07 NIA 899* (GAT (3)). Ivory Coast. Lagunes: Abidjan, Banco Forest Reserve, in marshy valley, near the entrance, *J. de Koning 6144B* (WAG [WAG0099439]). Nigeria. Rivers: Old GRA [Government reservation area], Port Harcourt, *B.E. Okoli 150* (IFE (cited and picture of plant in Okoli 1984)). R. Congo. Sangha: 36.89 km E-SE Bomassa, 2°11.85'N, 16°31.10'E, *S.T. Ndolo Ebika 401* (E [E00486113]). Sierra Leone. Kono: Tingi Mts, top of E ridge, *J.K. Morton & D. Gledhill SL1886* (WAG [WAG0225480], WAG [WAG0225481]). Tanzania. Kigoma: Kigoma Rural District, Gombe Stream National Park, Mitumba Valley, research staff houses, 4°39'11"S, 29°38'09"E, *G. Gobbo et al. 471* (MO). Zimbabwe. Manicaland: Chirinda Forest, *B. Goldsmith 39/62* (COI, K, NY).

### 4. Coccinia grandiflora

Taxon classificationPlantaeCucurbitalesCucurbitaceae

Cogn., Bot. Jahrb. Syst. 21: 211. 1895.

Coccinia grandiflora Cogn. ex Engl., Abh. K. Preuss. Akad. Wiss.: 34. 1894. nom. nud.
Coccinia grandiflora Type: Tanzania. [Tanga]: Mlalo, dry hill range. *C.H.E.W. Holst 506a* (Holotype: B, destroyed).
Coccinia grandiflora Type: Tanzania. Tanga: Usambara, near Amani, male, fl, [*H.J.P.?] Winkler 3611* (Neotype, designated here: BR!).
Coccinia engleri Gilg, Bot. Jahrb. Syst. 34: 354. 1904.
Coccinia grandiflora Type: Tanzania. [Tanga]: West Usambara, Sakare [Sakarre], at waterfall in primeval forest, 1100 m, fl, fr, Sep, *A. Engler, Reise nach Ostafrika 948* (Holotype: B, destroyed).
Coccinia grandiflora Type: drawing in protologue (Lectotype, designated here).

#### Description.

Perennial climber. Stems up to 20 m, glabrous or (when from higher altitudes) sparsely covered with long, whitish trichomes. Petioles 2.5–13 cm, indumentum as on stem. Leaves 12–20 × 11–20 cm, profoundly 5-lobate. Lobes triangulate, ovate to oblong. Leaf margin smooth to slightly serrate, dentate. Apex obtuse to acute with final tooth. Upper leaf surface glabrous with small hyaline pustules. Lower leaf surface glabrous, rarely with few trichomes on the main nerves esp. at base, with blackish glands scattered esp. along main nerves. Probracts up to 5 mm long (Fig. 8a). Tendrils bifid. Male flowers solitary or in (usually few-(–10-)flowered) racemes. Common peduncle 4–12 cm, glabrous. Pedicels of flowers in racemes 0.2–1.3 cm, glabrous. Bracts up to 3 mm or missing. Pedicel of solitary flowers 4–15 cm, glabrous. Perianth tube glabrous. Calyx lobes (2–)4–13 mm, lineal, narrowly lanceolate to triangulate, tip subulate to subacute. Corolla 4–6.5 cm long, apricot, salmon, yellowish-buff to yellow, lobes 2–4.7 cm. Filament column and anther head not seen. Pollen sac yellow-orange. Female flowers solitary, rarely in racemes, glabrous. Common peduncle < 1 cm, pedicel in racemes, pedicel of solitary flowers 3–4 cm. Ovary long cylindrical, glabrous or with hyaline–whitish pustules. Hypanthium glabrous, calyx lobes and corolla like in male flowers. Style columnar, yellowish to buff. Stigmas 2-lobed, yellow. Fruits < 30 × 2–4 cm, long cylindrical, glabrous, when unripe green, ripe (orange-)red. Seeds 4.5 × 2.5 × 1–1.2 mm (L/W/H), symmetrically obovate, face flatly lenticular.

#### Phenology.

Flowering time: January–December.

#### Distribution.

**Figure 25.**
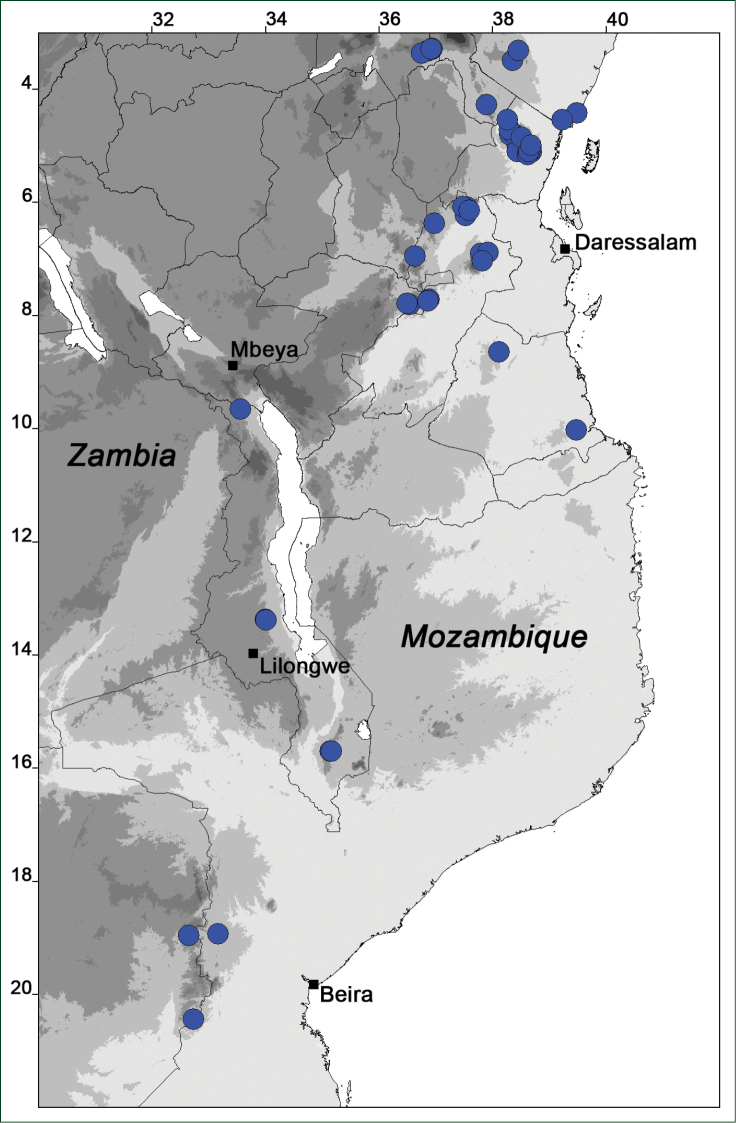
Distribution map of *Coccinia grandiflora* (based on 62 collections). For Tanzania the borders of the regions are given.

Fig. 25. Southeastern Kenya (Coast Province: Taita Hills, coastal forests), Tanzania (SE Dodoma; Iringa: Uzungwa Mts; Kilimanjaro; Lindi; Morogoro: Nguru Mts, Ukaguru Mts, Uzungwa Mts; Mtwara; Tanga: Usambara Mts), Malawi (Northern Region: Misuku Hills; Central Region: Nchisi Mts; Southern Region: Lisao Hill), Mozambique (Manica: East African Highlands with foothills), Zimbabwe (Manicaland: East African Highlands with foothills). Elevation 30–1900 m. On rich red-brown clay, over limestone, on diabase outcrops. Coastal and lowland forests and forest margins (*Parinari* sp.-*Newtonia buchanii* forests and others), riverine forests (with *Cola clavata*, *Synsepalum msolo*, *Sorindeia madagascariensis*), rarely in montane forests (*Cassipourea malosana*-*Teclea simplicifolia*-*Teclea nobilis*-*Olea mildbraedii*-*Tabernaemontana* forest), or Miombo from degraded forests.

#### Use.

Fruits are reported to be either poisonous (*A. Peter 56598*) or edible (*W.J. Kindeketa 630*). Leaves cooked in water used against fever (*K. Braun 714*).

#### Vernacular names.

Kihehe: mudesselema (*F. Haerdi 617/0*), Kipare: hotwe (*W.J. Kindeketa 630*), Kishamba: matombo shanga (*G.R. Williams G43*), Kisamba: matombo ya nyoka “snake breasts” (*M.A. Mwangoka & A. Kalage 1578*).

#### Remarks.

The southern distributed individuals in Zimbabwe and C Mozambique often bear short trichomes, and the leaves are rather shallowly lobate, just as in *Coccinia schliebenii*. These populations may represent hybrids or descendants of a non-differentiated common ancestor.

It is difficult to distinguish between *Coccinia grandiflora* and *Coccinia mildbraedii* in the Central Tanzanian highlands (Eastern Arc Mts). Both species also occur in high altitude forests and are clearly delimited by flower size. *Coccinia grandiflora* also has larger probracts than *Coccinia mildbraedii*, but this is rarely well visible. *Coccinia grandiflora* may also be confused vegetatively with *Coccinia barteri* in Mozambique and Zimbabwe.

#### Taxonomic remarks.

The *Coccinia grandiflora* holotype by Holst was destroyed in the fire of the Berlin herbarium in 1944. The Winkler specimen was chosen as neotype because it was already designated as type in December 2008. There is no annotation on the type label, however, and it seems that this neotypification was not published. However, the Winkler specimen label bears Cogniaux’ handwriting. Strangely, the Winkler specimen also states “mars 1892”, with the 92 crossed out. This is the date when Holst collected his specimen; but H. J. P. Winkler collected in Tanzania in 1910.

As the holotype of *Coccinia engleri* also was destroyed, the original material left is a drawing in the publication of the protologue. The drawing is of sufficient quality to synonymize unambiguously *Coccinia engleri* with *Coccinia grandiflora*.

#### Specimens examined.

(Selection, in total: 105) Kenya. Coast Province: Taita-Deveta District, Taita Hills, Mbololo Forest, 3°19'S, 38°27'E, Mwambirwa Forest Station, *R.B. Faden et al. 799* (EA, WAG [WAG0234141]). Malawi. Southern Region: Chiradzulu district, Lisau Hill above Njuli P.O., *R.K. Brummitt & I.H. Patel 18534* (K, WAG [WAG0361192]). Mozambique. Manica: Chimoio, Garuzo mountain ridge, *J.G. Garçia 522A* (LISC, MO). Tanzania. Arusha: above Saje, N side of Ngurdoto Crater rim, *P.J. Greenway & K. Kanuri 13444* (EA, K, M, PRE). Kilimanjaro: Chome [ward], Njokava forest, *R. Abdallah 814* (EA, NHT). Lindi: 40 km W of Lindi, Lake Lutamba, Mirola Valley, *H.J.E. Schlieben 5905* (B, HBG, G, M, S [S08-12068], Z (2)). Morogoro: Kilosa district, Ukaguru Mts, along track between Mandege and Ihanga rock, c. 6°24'S, 36°56'E, *M. Thulin & B.E. Mhoro 2877* (DSM, EA, K, MO). Tanga: western Usambara Mts, Shagai forest, forestry house near Sunga, *R.B. Drummond & J.H. Hemsley 2783* (B, EA, K, S [S08-12075]); [eastern Usambara Mts], Amani, c. 50 m before gate of headquarters, 05°05'58.3"S, 38°39'11.2"E, *N. Holstein et al. 96* (B, DSM, M), *97* (BR, DSM, M), *98* (DSM, M, WAG), *99* (DSM, M), and *100* (M). Zimbabwe. Manicaland: Chipinge district, outskirts of Chirinda [forest] above Msilizwe river, *B. Goldsmith 2/63* (B, BR, S [S08-12072]); [near Mutare], S slope of Murahwa’s Hill, *N.C. Chase 8008* (COI, K, LISC, MO).

### 5. Coccinia grandis

Taxon classificationPlantaeCucurbitalesCucurbitaceae

(L.) Voigt, Hort. suburb. Calcutt.: 59. 1845.

Bryonia grandis L., Mant. Pl.: 126. 1767. *Coccinia indica* Wight & Arn., Prodr. fl. Ind. orient.: 347. 1834. nom. illeg. [nom. superfl. as epithet has not been adopted]. *Coccinia grandis* M.Roem., Syn. Pepon.: 93. 1846. nom. illeg. [nom. superfl.] *Cephalandra indica* Naudin, Ann. Sci. Nat. Bot. ser. 5, 5: 16. 1866. nom. illeg. *Cephalandra grandis* (L.) Kurz, J. As. Soc. Beng. 46(2): 103. 1877. Type: Sri Lanka. *Bryonia foliis subrotundis angulosis, momordicae facies* Burm., Thes. zeylan.: 49, t. 19, fig. 2. 1737 (Type: drawing in l.c.). Type: Sri Lanka. *Vitis alba indica* Rumphius [G. E. Rumpf], Herb. Amboin. 5: 448, t. 166, fig. 1. 1747 (Type: drawing in l.c.).
Coccinia grandis Type: No detailed information, female, fl, *Anon. in Herb. Linn. 1153.2* (Typolectotype, designated by Nazimuddin and Naqvi (1984): LINN! [digital image: LINN]).
Coccinia grandis Type: India. Gottori, male and female, fl, *Anon. in Herb. Linn. 1153.3* (Type?: LINN! [digital image: LINN]).
Coccinia grandis Type: India. [Gujarat]: Suratt [Surat], female, fl, *Anon. in Herb. Linn. 1153.12* (Type?: LINN! [digital image: LINN]).
Coccinia grandis Type: India. No detailed information, female, fl, *Anon. in Herb. Linn. 1153.13* (Typotype: LINN! [digital image: LINN]).
Cucumis sativus var. arakis Forssk., Fl. aegypt.-arab.: 169. 1755.
Coccinia grandis Type: Yemen. [Al-Hudaydah Governorate]: Lohaja [Al-Luhayyah] [region?], Môr [Mawr], male, fl, *P. Forsskål 660* (Holotype: C [C10002122, digital image: JPS, microfiche IDC: 35 II, 7–8]).
Turia moghadd Forssk. ex J.F.Gmel., Syst. nat. 2(1): 403. 1791. *Turia moghadd* Forssk., Fl. aegypt.-arab.: 166. 1755. nom. inval. *Coccinia moghadd* (Forssk. ex J.F.Gmel.) Asch. in Schweinf., Beitr. Fl. Aethiop.: 251. 1867. *Cephalandra moghadd* (Forssk. ex J.F.Gmel.) Broun et Massey, Fl. Sudan: 105. 1929.
Coccinia grandis Type: Yemen. [Al-Hudaydah Governorate]: Lohaja [Al-Luhayyah], female, fl, *P. Forsskål 663* (Lectotype, designated here: C! [microfiche: IDC 110 I, 1–2]).
Coccinia grandis Type: ibid., male, fl, *P. Forsskål 662* (Syntype: C! [microfiche: IDC 109 III, 7–8]).
Coccinia grandis Type: ibid., *P. Forsskål 666* (Syntype: C! [microfiche: IDC 110 I, 3–4]).
Bryonia alceifolia [sphalm. *alceaefolia*] Willd. in Rottler, Neue Schriften d. Ges. Naturf. Freunde Berlin 4: 223. 1803. Coccinia cordifolia (L.) Cogn. var. alceifolia [sphalm. *alceaefolia*] (Willd.) Cogn. in A.DC. & C.DC., Monogr. Phan. 3: 531. 1881.
Coccinia grandis Type: India. [Tamil Nadu]: Tiruchinapally [Tiruchirappalli], male and female, fl, Nov 1793, *Anon.* [*J.G. Klein, B. Heyne or J.P. Rottler] in Herb. J.G. Klein 177* (Lectotype, designated here: B-W! [B-W 18065], isolectotype: K!).
Momordica covel Dennst., Schlüssel Hortus malab.: 23. 1818. *Cucumis pavel* Kostel., Allg. med.-pharm. Fl. 2: 738. 1833. nom. illeg. [nom. superfl.]
Coccinia grandis Type: *Covel* Rheede, Hort. malab. 8: 27, t. 14. 1688 (Holotype: drawing in l.c.).
Momordica bicolor Blume, Bijdr. fl. Ned. Ind.: 928. 1825–26.
Coccinia grandis Type: Indonesia. [Java], Kuripan, in calcareis [on calcareous ground?], *K.L. Blume 1012* (Holotype: L! [L 0587745]).
Momordica bicolor var. a Blume, Bijdr. fl. Ned. Ind.: 928. 1825–26. nom. inval. Indonesia. Maluku Province, Timor, *A. Zippelius s.n.* (L! [L 0587743]).
Momordica bicolor var. b Blume, Bijdr. fl. Ned. Ind.: 928. 1825–26. nom. inval. [Indonesia]. [Java], Parang Mts, calcareous mountains. *K.L. Blume 1016* (L! [L 0587744]).
Bryonia moimoi Ser. in DC. Prodr. 3: 305. 1828. *Moï-moï* Adans., Hist. nat. Sénégal: 159, Paris. 1757. nom. inval. *Coccinia moimoi* (Ser.) M.Roem., Syn. Pepon.: 93. 1846. Type: Sri Lanka. *Bryonia folio anguloso acuto glabro* Burm., Thes. zeylan.: 48, t. 19, fig. 1. 1737 (Holotype: drawing in l.c.).
Momordica monadelpha Roxb., Fl. ind. [2^nd^ ed.], 3: 708. 1832. nom. illeg. [nom. superfl.] pro parte. *Bryonia foliis cordatis oblongis angulatis dentatis glabris*. Fl. Zeyl. 356. 1747. nom. inval. Type: Sri Lanka. *P. Hermann Mus. Zeyl.* 2: 37 (Type: BM [BM000621642], BM [BM000621643]) [these two specimens are *Cucumis maderaspatanus* L.].
Coccinia grandis Type: Sri Lanka. *P. Hermann Mus. Zeyl.* 5: 225 (Lectotype, designated here:: BM [BM000595000]).
Coccinia grandis Type: Sri Lanka. *P. Hermann Mus. Zeyl.* 5: 321 (Type: BM [BM000621089]). Type: Sri Lanka. *Bryonia folio anguloso acuto glabro* Burm., Thes. zeylan.: 48, t. 19, fig. 1. 1737. (Type: drawing in l.c.). Type: Sri Lanka. *Bryonia foliis subrotundis angulosis, momordicae facies* Burm., Thes. zeylan.: 49, t. 19, fig. 2. 1737 (Type: drawing in l.c.). Type: Sri Lanka. *Vitis alba indica* Rumphius [G. E. Rumpf], Herb. Amboin. 5: 448, t. 166, fig. 1. 1747 (Type: drawing in l.c.).
Coccinia loureiriana M.Roem., Syn. Pepon.: 93. 1846. *Bryonia grandis* Lour., Fl. cochinch. 1(2): 595. 1790. nom. illeg. and Fl. cochinch. 2: 731. 1793. nom. illeg. Type: Sri Lanka. *Bryonia foliis subrotundis angulosis, momordicae facies* Burm., Thes. zeylan.: 49, t. 19, fig. 2. 1737. (Type: drawing in l.c.). Type: Sri Lanka. *Vitis alba indica* Rumphius [G. E. Rumpf], Herb. Amboin. 5: 448, t. 166, fig. 1. 1747 (Lectotype, designated here: drawing in l.c.).
Coccinia wightiana M.Roem., Syn. Pepon.: 93. 1846. Coccinia cordifolia (L.) Cogn. var. wightiana (M.Roem.) Cogn. in A.DC. & C.DC., Monogr. Phan. 3: 531. 1881. Coccinia grandis var. wightiana (M.Roem.) Greb. in R. Mansfeld & J. Schultze-Motel, Verz. Landwirtsch. u. Gaertn. Kulturpfl. 2: 929. 1986. *Coccinia indica* Wight et Arn. β, Prodr. fl. Ind. orient.: 347. 1834.
Coccinia grandis Type: India. [Tamil Nadu]: [= Wall. Cat 6711a], [*J.G. Klein, B. Heyne* or *J.P. Rottler*] *in Herb. Madras s.n.* (Syntype: E! [E00174668, digital image: E, JPS]).
Coccinia grandis Type: India. [Tamil Nadu]: [Chennai, Saidapet], Nopalry [= in Wall. Cat. 6711b or e], female, fl, *R. Wight 1124* (Lectotype, designated here: E! [E00174667, digital image: E, JPS]).
Coccinia grandis Type: India. [Tamil Nadu]: Negapatam [Nagapattinam], female, fl, fr, *R. Wight 1124* (Syntype: E! [E00174666, digital image: E, JPS]).
Coccinia grandis Type: unknown. *R.Wight 1124* (Syntypes: E! [E00174664], NY! [00172358, digital image: NY]).
Coccinia grandis var. quinqueangularis Miq., Fl. Ned. Ind. 1(4): 673. 1856.
Coccinia grandis Type: [Indonesia]. [Central Java]: near Soerakarta [Surakarta], *T. Horsfield s.n.* (Holotype: U!).
Coccinia schimperi Naudin, Ann. Sci. Nat. bot., ser. 4, 8: 366. 1857. *Cephalandra schimperi* (Naudin) Naudin, Ann. Sci. Nat. Bot. ser. 5, 5: 16. 1866.
Coccinia grandis Type: Ethiopia. In Semen [Semien Mts], female, fl, 1854, *G.H.W. Schimper Herb. Abyss. 1215* (Lectotype, designated here: P! [the specimen with thick branch and fruit], isolectotype: P!).
Coccinia grandis Type: Ethiopia. Biria Dekeno et Dschadscha, 5000’, female, fl, 1853, *G.H.W. Schimper Herb. Abyss. 1215* (Syntype: P!).
Coccinia grandis Type: Ethiopia. Dschadscha, 5000’–5500’, male and female, fl, fr, 13 and 21 Jul 1853, *G.H.W. Schimper Herb. Abyss. 1215* (Syntype: P!).
Coccinia grandis Type: Ethiopia. Dschadscha, 5000’, male and female, fl, fr, 13 and 21 Jul 1853, *G.H.W. Schimper Herb. Abyss. 1215* (Syntype: P!).
Coccinia grandis Type: Ethiopia. without details, *G.H.W. Schimper Herb. Abyss. 1215* (Syntypes: BR! [BR0000005113958, digital image: BR, JPS], BR! [BR0000005114641, digital image: BR], BR! [BR0000008251053, digital image: BR, JPS], BR [BR0000008351050], G-DC!).
Coccinia grandis Type: Ethiopia. Dschadscha, female, 1853, *G.H.W. Schimper Herb. Abyss. 1215* (Syntype: BR! [BR0000005111923, digital image: BR]).
Coccinia palmatisecta Kotschy, Sitzungsber. K. Akad. Wiss. Math.-Naturwiss. Cl. Abt. 1, 51: 360–361. 1865.
Coccinia grandis Type: [South Sudan], Kyk [Ciec (a Dinka subtribe) realm, S of confluence of Bahr al-Ghazal and White Nile], male, fl, no date given, *M.L. Hansal s.n.* (Lectotype, designated here: W! [K neg. 4837]).
Coccinia grandis Type: [South Sudan]. marshes in Noer [Nuer] realm [S to E of Malakal], no detailed location given, *F. Binder s.n.* (Syntype: W! [K neg. 4838]).
Cephalandra indica var. palmata C.B.Clarke in Hook.f., Fl. Brit. Ind. 2(6): 621. 1879.
Bryonia alceifolia [sphalm. *alceaefolia*] Willd. in Rottler, Neue Schriften d. Ges. Naturf. Freunde Berlin 4: 223. 1803. *Coccinia wightiana* M.Roem., Syn. Pepon.: 93. 1846.
Coccinia grandis Type: India, [Tamil Nadu]: Tiruchinapally [Tiruchirappalli], male and female, fl, Nov 1793, *Anon.* [*J.G. Klein, B. Heyne or J.P. Rottler] in Herb. J.G. Klein 177* (Syntypes: B-W! [B-W 18065], K!)
Coccinia grandis Type: India. [Tamil Nadu]: [= Wall. Cat 6711a], [*J.G. Klein, B. Heyne* or *J.P. Rottler*] *in Herb. Madras s.n.* (Lectotype, designated here: E! [E00174668, digital image: E, JPS]).
Coccinia grandis Type: India. [Tamil Nadu], [Chennai, Saidapet], Nopalry [= in Wall. Cat. 6711b and e], female, fl, *R. Wight 1124* (Syntype: E! [E00174667, digital image: E, JPS]).
Coccinia grandis Type: India. [Tamil Nadu]: Negapatam [Nagapattinam], female, fl, fr, *R. Wight 1124* (Syntype: E! [E00174666, digital image: E, JPS]).
Coccinia grandis Type: unknown. *R.Wight 1124* (Syntypes: E! [E00174664], NY! [00172358, digital image: NY]).
Coccinia cordifolia (L.) Cogn. in A.DC. & C.DC., Monogr. Phan. 3: 529. 1881. pro parte majore, non *Bryonia cordifolia* L.
Coccinia cordifolia (L.) Cogn. var. genuina Cogn. in A.DC. & C.DC., Monogr. Phan. 3: 531. 1881. nom. invalid.

#### Description.

Perennial climber or creeper. Stems up to 5 m, glabrous, when older often white pustulate. Petioles 0.5–5.5 cm, glabrous, rarely some trichomes on adaxial side. Leaves 3–11 × 3–13 cm, cordate to 3-lobate or 5-edged to 5-lobate, sometimes lobulate. Lobes triangulate, ovoid, oblong, to obovoid. Leaf margin dentate, teeth usually with yellowish-reddish to brownish gland (Fig. 7a), becoming black when dried. Margin rarely with short (< 1 mm), whitish trichomes. Apex obtuse to acute with final tooth. Upper leaf surface glabrous, more or less dense hyaline to white pustulate. Lower leaf surface glabrous, with glands that are usually framed with lighter color between major nerves, nerves sometimes with white pustules. Probracts < 1.5 mm or missing. Tendrils simple. Male flowers 1(–3) solitary, rarely in short racemes. Peduncle 0.3–1.5 cm, glabrous, pedicels of flowers in racemes up to 3.2 cm, glabrous, pedicels of solitary flowers up to 4.5 cm, glabrous. Bracts inconspicuous (< 1 mm), often absent. Perianth tube glabrous. Calyx lobes 1.2–3.5 mm long, lineal, spreading to reflexed, often with reddish to brownish gland on lower side at the acute tip (Fig. 7a). Corolla 1.7–4 cm long, yellowish buff (mostly African collections) to snow-white (esp. outside Africa). Corolla lobes 0.7–1.7 cm. Filament column and anther head pale greenish, pollen sacs yellow. Female flowers 1(–2) solitary. Pedicel up to 1 cm, glabrous. Hypanthium glabrous, calyx and corolla like in males. Ovary glabrous. Style columnar, yellowish-green. Stigmas 2-lobed, greenish. Fruit globose to ellipsoid, cultivated also shortly cylindrical, 3–4.5 × 1.5 cm, glabrous, unripe greenish with few pale spots and/or lines, becoming scarlet red when ripe. Seeds 5–7 × 2.5–3.5 × 1.2 mm (L/W/H), asymmetrically obovate, face flat (Fig. 14f).

#### Phenology.

Flowering time: All over the year, but not at the end of the dry season or in cold seasons. Seems to need 1–2 weeks of at least 10 hours daylight with sunny weather for flower induction (pers. observ. from greenhouse cultivation).

#### Distribution.

**Figure 26.**
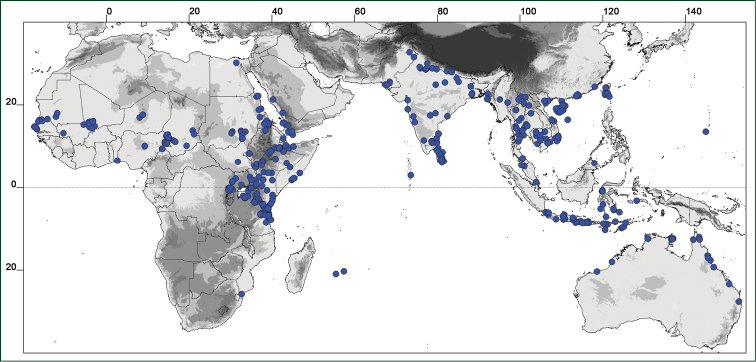
Partial distribution map (Africa to Australia) of *Coccinia grandis* (based on 629 collections).

**Figure 27.**
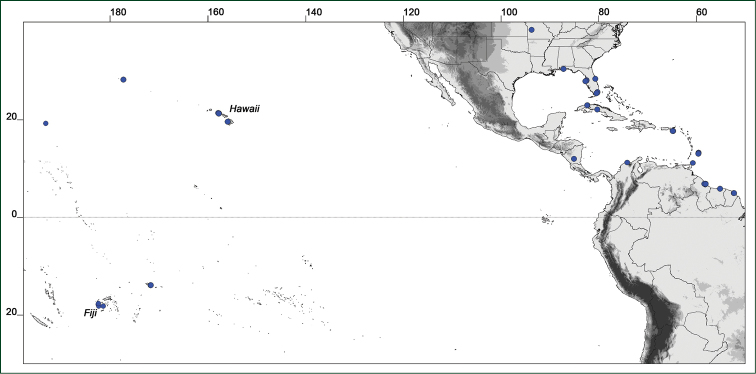
Partial distribution map (Pacific Ocean and Americas) of *Coccinia grandis* (based on 629 collections). State borders are given for the USA.

Figs 26, 27. Benin, N Cameroon, S Chad, D. R. Congo (in Great African Rift valley), Djibouti, Egypt (along Nile, Elba Mts), Eritrea, Ethiopia, Kenya, Mali (except N), S Mauritania, Niger, Senegal, Somalia, South Sudan, S and E Sudan, N Tanzania (Arusha, Dar es Salaam, Manyara, Morogoro, Mwanza, Pwani, Tanga, Zanzibar), Uganda, mountains and oases of the southern and western Arabian Peninsula, tropical and subtropical India, subtropical Nepal, Pakistan, Sri Lanka, South East Asia, S China incl. Hainan. Occurrence likely due to recent introduction in: Australia (Northern Territory, Queensland, Western Australia), R. China, Maldives, Mauritius, Mozambique, USA (Florida, Guam, Hawaii, Wake Island), many tropical Pacific islands, Caribbean area, Central and tropical South America. 0 to 1600 m. Black clay soil, black cotton soil, sand, on limestone. There seems to be a certain salt tolerance (Bharathi 2007). Grasslands, bushlands, (semi-arid) savannas, thickets, along rivers, ruderal sites, seemingly avoiding closed canopies (woodlands and forests). *Coccinia grandis* is, especially in tropical Asia, often reported from sand or from calcareous grounds (karst areas), which are well-drained. Although precipitation in South East Asia is much higher than in Africa, the quick loss of surface water allows *Coccinia grandis* to survive there.

#### Use.

Fruits (raw and cooked) and shoots (cooked) edible. The Luo eat the leaves as spinach (Orech et al. 2005). The sap is used against diabetes (Ramachandran and Subramaniam 1983) due to its hypoglycemic activity (Chopra and Bose 1925; Shibib et al. 1993). It is used in traditional Indian medicine in India for various diseases, and seems to have a general antibacterial effect (see also Use, economic potential, and phytochemistry).

#### Vernacular names.

Agau or Chomir [most likely: Khamir/Xamtanga language of Agaw language family]: amballa gosa (*G.H.W. Schimper 365*, Schweinfurth 1893); Arabic: mogad (Getahun 1974b); Bagirmi: na odio (*A.J.B. Chevalier 9527*); Bengali: tala-kucha (Nadkarni and Nadkarni 1976), tela kucha [tēlākucā] (*J. Sinclair 4414*); Canarese [Kannada]: tonde-konde, tonde-kayee [toṇḍe kāyi] (Nadkarni and Nadkarni 1976); Chinese: hong gua [Hóng guā] (Wu et al. 2011); Dassanetch [Daasanach]: dali (*C.J. Carr & C. Metolo 867*); Galinya [Oromo]: raho (*T. Ebba 624*; Getahun 1974b); Gujarati: gholi (Nadkarni and Nadkarni 1976); Hindi: kan-duriki-bel (Nadkarni and Nadkarni 1976), kundru [kundrū] (Bharathi 2007); Karen [in Mae Hong Son Province, Thailand]: khae-do (de Wilde and Duyfjes 2008); Khmer: (sloek’) bah (Kéraudren-Aymonin 1975b); Kizigua: lukewja (*R.E.S. Tanner 2691*), pondwa (*R.E.S. Tanner 2030*); Lao: (khùa ‘phăk) tăm ling, tăm nhing (Kéraudren-Aymonin 1975b), phak tam nin (*J.-M. Dubost 200*); Luo: nyathud gush (*S.H. Padwa 71*); Mahrati [Marathi]: ran-tondula, tondli (Nadkarni and Nadkarni 1976); Malayalam: kova (Nadkarni and Nadkarni 1976), kovakka (Bharathi 2007); Masai: olaposhi (*C.H. Pecler 3*); Nepali: gol kankri (Aryal 2007); Ngadha (Baba-Ngadha): `uta kala (*J.A.J. Verheijen 5415*); Ngadha (Mataloko-Ngadha): uta kobho (*J.A.J. Verheijen 5415*); Persian [Farsi]: kabare-hindi (Nadkarni and Nadkarni 1976); Punjabi: kanduri (Nadkarni and Nadkarni 1976); Sanskrit: vimboshta, vimbaja, bimba, tundika [tuṇḍikā] (Nadkarni and Nadkarni 1976); Serer: ayandyaloup (*J.-F. Ezanno Cucurbitaceae 9*); Sinhala: kowakka (*D. Philcox et al. 10454*, *D. Philcox et al. 10458*); Somali: masskar (*T. Ebba 804*); Songhai: lombaria (*A.J.B. Chevalier 2996*); Sunda: aroi papassang (Miquel 1855); Tamasheq [Touareg]: seffel (*H. Lhote 2*). Tamil: kovai [kowai] (Nadkarni and Nadkarni 1976), kovakkai (Bharathi 2007); Telugu: dondakaya [doṇḍa kāya] (Bharathi 2007), dondatiga, kakidonda (Nadkarni and Nadkarni 1976); Thai: in Central Region: phak tamlueng, in Northern Region: phak khaep (de Wilde and Duyfjes 2008); Tigrinya: asumbeh (*G.A. Schweinfurth & D. Riva 1007*); Vietnamese: northern: hoa bát, southern: (dây) bai bác, bình bát, ma'nh bát, lá bát (Kéraudren-Aymonin 1975b); Wolof: barbouf (*J. Trochain 663*).

#### Remarks.

With the exception of South and South East Asia, *Coccinia grandis* is easily recognizable, especially by the lack of an obvious indumentum and the pale framed (in living state) glands in the axils of the nerves at the base of the lower leaf lamina. In NE Africa, collections with finely dissected leaves can be similar to *Coccinia ogadensis*. When compared to collections from South Africa, Coccinia sessilifolia var. variifolia and some forms of *Coccinia mackenii* are also similar, but Coccinia sessilifolia var. variifolia is glaucous, *Coccinia mackenii* plants have bifid tendrils, and both South African species lack colored leaf teeth and have erect to spreading calyx lobes instead of spreading to reflexed calyx lobes. In South and South East Asia, some vegetatively similar *Gymnopetalum* species (e.g., *Gymnopetalum chinense*) can be mistaken for *Coccinia grandis*, as in both taxa the leaf shape is 5-edged to cordate, and glands on the lower leaf lamina can be found. However, *Gymnopetalum* species are rather densely beset with trichomes, have ribbed fruits and are monoecious, whereas *Coccinia grandis* is glabrous, has smooth fruits, and is dioecious, at least in wild populations.

Asian and (at least most of the) African *Coccinia grandis* differ genetically (e.g., in a short sequence in the 5’-beginning of the *LEAFY*-like 2^nd^ intron) and in petal color (white in Asian, buff in African individuals). Hence, the distribution in Asia (at least India) is not due to human impact. Whether *Coccinia grandis* is introduced or native to Malesia, northern Australia and southern China and Taiwan is not known. Crossing experiments by Naudin (1862) indicate full compatibility between the African and Asian morphs though.

#### Taxonomic remarks.

Up to the 21^st^ century (e.g., Bulbul et al. 2011; Hussain et al. 2011), there has been quite a lot of confusion about the valid name of the species that is now called *Coccinia grandis*. Wight and Arnott (1834) established the genus *Coccinia*, with the name based on the scarlet-red fruits of a species that Wight had collected several times during his 13-year stay in South India (Stafleu and Cowan 1988). The name of the only species they described in their new genus, *Coccinia indica* Wight & Arn., is illegitimate since they included *Bryonia grandis* L., of which they ought to have adopted the epithet (Art. 52.1 ICN). The illegitimacy of *Coccinia indica*, however, does not affect the legitimacy of the genus name *Coccinia* (Art. 42.2), although there was also some confusion about which genus name to use. In 1845, Voigt published the correct combination *Coccinia grandis* (L.) Voigt with a description exactly matching *Bryonia grandis*.

Although *Coccinia indica* is not valid, and the problem seemed to have been solved, a third species name was brought into discussion by Cogniaux. He thought that *Bryonia cordifolia* L. and *Bryonia grandis* L. referred to the same species as Linnaeus cited Rumphius’ *Vitis alba indica* (*Coccinia grandis*) under *Bryonia cordifolia* (Linnaeus 1763). Hence, Cogniaux created a more broadly circumscribed *Coccinia cordifolia* (L.) Cogn. (1878). However, Linnaeus described *Bryonia cordifolia* beforehand (1753) citing the description from his *Flora Zeylanica* number 354 (1747), and only in the 2^nd^ and 3^rd^ edition of his *Species Plantarum* did Linnaeus synonymize Rumphius’ *Vitis alba indica* (1747). Cogniaux therefore erred, when he stated that Linnaeus had based his *Bryonia cordifolia* on Rumphius’ figure, and the epithet *cordifolia* is hence misapplied in *Coccinia*.

There are four specimens in Linnaeus’ herbarium, which belong to *Coccinia grandis* but it is unclear which are type material. The number *1153.2* is designated (by Linnaeus?) as *Bryonia grandis* on the sheet and is therefore the best choice for lectotypification as is has been done by Nazimuddin and Naqvi (1984). The specimen *1153.13* bears the note “Bryonia foliis subrotundis angulosis, momordicae facie” on the specimen flip side referring to one of the citations to drawings in the protologue. Hence, this specimen is also original material. The other two specimens (*1153.3* and *1153.12*) do not bear indications that Linnaeus referred to them as *Bryonia grandis*, but there is no contraindication either, so they might be original material, too.

The drawing to *Bryonia foliis subrotundis, angulosis, momordicae facie* Burm. (Thes. zeylan.: 49, t. 19, fig. 1. 1737), which is original material of *Bryonia grandis* L. appears to be a product of artistic freedom. The calyx lobes of the three uppermost and the lowermost flowers are reflexed and match *Coccinia*/*Bryonia grandis* well, whereas the calyx of the other two flowers appears to consist of almost free elliptic petals, quite like in *Momordica foetida* (except for the soft spines that are missing in the drawing). The addition “momordica facies” seems to relate to this. Strangely, the drawing of *Bryonia folio anguloso acuto glabro* Burm. (Thes. zeylan.: 48, t. 19, fig. 1. 1737) matches the current definition of *Bryonia grandis* also well, but has not been cited by Linnaeus. Eventually, the synonyms that Burman used, which also include *Bryonia cordifolia* L. (*Cucumis maderaspatanus* L.), made it hard to interpret the species and also lead to the confusion of Cogniaux.

The *Forsskål 660* specimen (C10002122) has a hand-written field label by Forsskål (according to notes in JStor Plant Science) on the flip side, stating “Cucumis incerta. Arakis, Mour.” (incerta meaning “uncertain”) and two hand-written identifications “Cucumis inedulis Fl. Yemen CXXII nr. 580” and “c. s. Arakis cent VI nr. 61 p. 169”. The location in the text is the same as on the field label: Môr/Mour [Mawr, a small town about 30 km E of al-Luhayyah]. The former identification is a nom. nud. with the number 580 on page CXXII of Forsskål’s Flora Ægyptiaco-Arabica (1775). The second identification links to a *Cucumis sativus* variety that is validly described in that book on page 169. The description matches well *Coccinia grandis* except for the tuberculate ovary. Additionally the collection is supposed to be from Loheja [al-Luhayyah], but might only indicate the region, in which Mawr is localized. However, the Arabic name of *Cucumis inedulis* and Cucumis sativus var. arakis are both: Arakis [3raqīs], so they can be cross-referenced. The description of the variety also mentions that the plant is not edible, just as the supposed species epithet.

The genus name *Turia* has been created by Forsskål in his Flora Ægyptiaco-Arabica (1775). There is a debate, however, whether it is validly published (Friis 1984). The descriptions of this part of Forsskål’s book (page number in Latin) must be used in consideration with the corresponding parts in the lists of local floras (Roman page numbers), which is on page CXXI in the case of *Turia*. The first species there is *Turia sativa* (no. 550), which is called “turia” in Arabic and is cultivated according to the epithet. This matches exactly the first description in the descriptive part of the book (p. 165). Forsskål lists five *Turia* species in the floristic part and describes five species in the descriptive part. Therefore, the genus *Turia* lacks a description (Jeffrey 1962), because Forsskål does not mention any character to be typical for the genus. The name *Turia* was legitimately described by Gmelin in an extension of Linnaeus’ Systema naturae (1791), and so was the name *Turia moghadd*.

The typification of *Turia moghadd* is not straight-forward, because Forsskål added little, if any marks on the sheets (Friis 1983). The flip side of the *Forsskål 663* specimen has three notes: “Cucumis glandulosus”, a second one with a different handwriting: “Bryonia Turia 35 Forsk” and a third one with another handwriting “Turia gijef Forsk Cent. 6 no. 38” with all words except for “Turia” crossed out. It can be hypothesized that these are different trials to identify the specimen, but not by Forsskål himself. Aside from these, the specimen lacks any written marks, but it matches exactly the description of *Turia moghadd*, as do the specimens *Forsskål 662* and *666*, which are syntypes. The *Forsskål 663* specimen is chosen to be the lectotype, because the original description mentions the occurrence of fruits and this specimen is the only female of the three.

Willdenow describes *Bryonia alceifolia* in a travel report by J. P. Rottler from 1799, but he only mentions that he separates Rottler’s *Bryonia epigaea* from another new species, viz. *Bryonia alceifolia*, so the type was not necessarily collected in 1799. Willdenow knew *Bryonia alceifolia* from Klein’s specimens in his herbarium. Rottler was missionary in Tranquebar, the same place in which J. G. Klein was surgeon (Jensen 2005). Both are known to have collected together (with B. Heyne). The lectotype collection is in the describer’s herbarium under the Klein number 177, but it is not certain, whether Klein collected the specimen himself, of if it was by Rottler or even Heyne. A duplicate with the same label data as in B-W is deposited in K. For Willdenow described the species, the specimen in his herbarium was chosen to be lectotype.

The name *Momordica monadelpha* Roxb. is superfluous, because Roxburgh synonymized *Bryonia moimoi* Ser. in total by citing the only element of that name and *Bryonia grandis*. The other elements of *Momordica monadelpha* are also interesting, though. Roxburgh cited “*Bryonia foliis subrotundis*” with the citation of Burman’s Thes. zeylan.: t. 19, fig. 1. 1737 and fig. 2, *Vitis alba indica*, all *Coccinia grandis*, and Herman’s Musæum Zeylanicum 356. The latter one consists of two specimens (2: 37), which are *Cucumis maderaspatanus* L., however, there are also two drawings (5: 225 and 5: 321) with the number 356. Both drawings represent *Coccinia grandis* because of the fruit size, fruit shape, and the flower morphology (calyx lobe length and position, corolla size), rather than *Cucumis maderaspatanus* L., *Diplocyclos palmatus* L. or *Cayaponia laciniosa* (L.) C.Jeffrey. That the drawing 5: 225 (BM000595000) shows a plant with male and female flowers on one individual might be explained best by artistic freedom.

The name Coccinia indica var. palmata C.B.Clarke is valid and legitimate but not obvious to typifiy. Despite *Coccinia indica* being illegitimate as a nomen superfluum, the variety is legitimate and validly described. Clarke cites *Bryonia alceifolia* Willd. and the *Coccinia indica* protologue with page 348 although *Coccinia indica* was described on page 347 (Wight and Arnott 1834). Page 348, however, only comprises the β variety, which itself was the basis for *Coccinia wightiana* M. Roem. This unnamed variety consists of several elements, of which the literature citations of *Bryonia palmata* are mentioned with a question mark and are thus not eligible for typification (Art. 52.2 N1). The element “Bryonia palmata Linn.? herb. Madr.!” relates to a collection in Herbarium Madras that was identified a *Bryonia palmata*, but Wight and Arnott appear to have been in doubt whether the name was used sensu Linnaeus, hence the question mark after the name. This specimen (or a duplicate) is found in E and represents a deeply lobed *Coccinia grandis* just as the protologue of the β variety says. Clarke obviously takes the epithet of the variety from this misidentification and not from *Bryonia palmata* L., which is why the name was typified with this collection. That Clarke meant a deeply lobed *Coccinia grandis* is evident since he also cites *Bryonia alceifolia*, which is also deeply lobed. It is thus not a new combination and status change but a new variety.

The name Coccinia cordifolia var. genuina Cogn. cannot be regarded as intended to represent a new variety, because Cogniaux divides all specimens he had seen into three varieties. Hence, the epithet “*genuina*” and his “*genuina*” variety is just synonymous with the autonym.

#### Specimens examined.

(Selection, in total: 1066). Australia. Queensland: Weipa, Awonga Court, 12°37'28"S, 141°52'39"E, *B.M. Waterhouse 7428* (BRI, CANB [CANB 682857]). Bangladesh. Sylhet: Sillet, *F. de Silva in Wall. list 6700g* (M [“439”], M [“440”]). Barbados. St. Michael: Bridgetown, old railway yard, Sep 1940, *E.G.B. Gooding s.n.* (BAR00002591). Brazil. Minas Gerais: No detailed location, < 1840, *P.C.D. Clausen s.n.* (G-DC). Cameroon. North Region: Pitowa [Pitoa], c. 17 km NE of Garoua, *W.J.J.O. de Wilde et al. 4932* (MO, WAG [WAG0225471]). Chad. Chari-Baguirmi: Baguirmi et région du Lac Fittri, Tjecna, *A.J.B. Chevalier 9527* (G, P [P05620497]). P. R. China. Hainan: Ch’ang-kiang District [Changjiang country], [Bawangling Nature Reserve], Ka Chik Shan [Qicha hill], *S.K. Lau 3008* (GH n.v., P [P06394498], S). Hong Kong: near Ouang-nei-Tchong [Wang nai chung] – Happy Valley, *E.M. Bodinier 1367* (E). Yunnan: Nan-chiao [Meng che], *C.W. Wang 76988* (A n.v., IBSC [0207527], KUN n.v., PE [01178321], PE [01178322]). R. China. Pingtung County: Hsinpi [Sinpi], *Y.-P. Cheng 4603* (BR [BR0000005164189], TAIF). Cuba. Havana: Santiago de las Vegas, *A. O’Donovan 5106* (G, S [S08-12050]). D. R. Congo. Orientale: Mahagi territory, Mahagi-Port, *J. Lebrun 3800* (BR (2), EA, WAG [WAG0225470]). East Timor. Dili: just W of Dili, Tasutolu area, 8°33'57"S, 125°30'05"E, *L.D. Cowie 10658* (L). Egypt. Red Sea Governorate: [Hala’ib triangle], Gebel Shendib, *G.W. Murray 3857* (K [K000037307]). Eritrea. Gash-Barka: Mai Mentai [at Sciagolgol River, SE of Agordat], *N. Beccari 118* (FT). Northern Red Sea Region: [NE of Ghimda/Gimda], Pianura Sabarguma, *A. Pappi 3970* (G, MO, P [P05620558], S [S08-12133], W). Ethiopia. Oromia: 85 km NE of Nazareth, along road to Awash, c. 5 km W of Metahara, 8°55'N, 39°55'E, *J.J.F.E. de Wilde 6870* (BR, MO, WAG [WAG0225448], WAG [WAG0225449], WAG [WAG0225450]). Tigray: near Djeladjeranné, 23 Apr 1841, *G.H.W. Schimper 1570* (P [P05620544], specimens from BM, G, MO, S [S08-12147], and W [W 0011063] might also be from this location, for details see under *Cucurbita schimperiana*). Guyana. Demerara-Mahaica: Georgetown, *W. Hahn 4810* (MO, US). India. West Bengal: Sibpur [Shibpur in Howrah city] near Calcutta [Kolkata], *J.G. Hallier s.n.* (M [“999”]). Indonesia. East Java: Pasoeroean [Pasuruan], 17 Jul 1928, *J.J. Ochse s.n.* (B, BR [BR0000009938076], L [L 0587578], P [P06394462], U). Mali. Kayes: Bafoulabé Cercle, Bamafélé arondissement, 3.2 km ESE of Manantali, 13°10.847'N, 10°26.260'W, *C.S. Duvall 130* (MO (2)). Mozambique. Maputo: Vila Luísa [Marracuene], along Incomáti river, *A. Balsinhas 1273* (COI). Nepal. Madhya Pashchim: [Rapti], Tulsipur, *O. Polunin et al. 5906* (E). Nicaragua. Chontales: St Tomas [Santo Tomàs], 1841, *E. von Friedrichsthal s.n.* (W). Niger. Agadez: [Aïr Mountains], Mt Bagwezan [Mt Bagzan, Idoukal-n-Taghès], 31 May 1920, *A. Buchanan s.n.* (MO [acc. no. 1667128]). Pakistan. Sindh: 7 mls [c. 11.2 km] from Hyderabad to Tando M.[Mohammad] Khan, *S. ul-Abedin 3839* (B, KUH n.v., MO). Senegal. Dakar: Jof [Yoff], *J.-G. Adam 1806* (MO (2), [non P [P00694028/P05590411], which is a *Citrullus* sp.]). Singapore. South East District: Potong Pasir off Serangoon Road, *J. Sinclair 5434* (E, L [L 0587600], P [P0639450]). Somalia. Togdheer: [c. 54 km SSW of Berbera], foot of Sheikh Pass, *P.R.O. Bally 11827* (G (3), K, PRE). Sri Lanka. North Central Province: Anuradhapura, *F.R. Fosberg & N. Balakrishnan 53431* (MO, PDA n.v.); ibid., *W. Forstner s.n. W13705* (W). Sudan. North Kurdufan: Chursi [Khursi], *K.G.T. Kotschy Iter nub. 308* (BM, BR (2), E, G (2), G-DC, GOET, K, L, M [“400”], M [“401”], M [“782”], MO, P [P05620478], P [P05620484], P [P05620578], PRC, S [S08-12152], TUB [TUB004721], TUB [TUB004722], W [W 0011066], W (2), WAG [WAG0234161]). Tanzania. Tanga: Tanga town, near public beach, 200 m E of 05°03'19.2"S, 39°07'31.3"E, *N. Holstein et al. 94* (B, DSM, M, WAG), and *95* (DSM, M). Zanzibar Urban/West: Chukwani, *H.G. Faulkner 2782* (BR, COI, K n.v., P [P05620529]). Thailand. Bangkok: Bangkok, *R. Zimmermann 66* (B (2), BR [BR0000009938878], G (2), H, L [L 0587657], M, MO, P [P06394565], PR [PR 801377], PRC, S, U, W). USA. Florida: along Indian River [lagoon], Coco [Cocoa], 9 May 1918, *J.K. Small s.n.* (FLAS [FLAS 29152], G, MO [acc. no. 1161454], US). Hawaii: O’ahu Island, Dole St, behind U Hawaii Manoa dorms, *B. Kennedy 47* (MO, US [00674433]). Midway Atoll: Sand Island, 4208 Commodore Ave., *F. Starr & K. Martz 080601-01* (BISH, PTBG [PTBG1000013099]). Vietnam. Da Nang: Tourane [Da Nang] and vicinity, no detailed location, *J. Clemens & M.S. Clemens 3257* (A n.v., G, MO, P [P06394668], U, W, Z). Yemen. Al-Hudaydah: Mor [Mawr], 15°40'N, 28–30 Mar 1825, *F.W. Hemprich s.n.* (P [P06394550], S [S08-12138]).

### 6. Coccinia heterophylla

Taxon classificationPlantaeCucurbitalesCucurbitaceae

(Hook.f.) Holstein, Kew Bull. 65(3): 435–441. 2010 [published in 2011].

Physedra heterophylla Hook.f. in D.Oliv., F. T. A. 2: 553. 1871.
Coccinia heterophylla Type: Angola. [Cuanza Norte]: Golungo Alto, along the banks of the stream Casaballa, at the base of the mountains in Sobato de Bumba, male, fl, Oct 1855, *F.M.J. Welwitsch 791* (Lectotype: BM! [BM000948006, digital image: BM, JPS], selected in Holstein and Renner (2010: 440); isolectotype p.p.: LISU! [LISU214547, digital image: JPS]).
Coccinia heterophylla Type: Angola. No detailed location, female, fr, Jan 1856, *F.M.J. Welwitsch 791* (Syntype: BM! [BM000948008, digital image: BM, JPS]). Type: Angola. In rugged places at Delamboa river, with *Coffea melanocarpa*, no clear date given, *F.M.J. Welwitsch 791* (Syntype: BM! [BM000948007, digital image: BM, JPS]).
Coccinia heterophylla Type: Angola. No detailed location and date, *F.M.J. Welwitsch 791* (Syntype: COI! [COI00005515, digital image: JPS]).
Coccinia heterophylla Type: Angola. No detailed location, male, fl, no date, *F.M.J. Welwitsch 791* (Syntype: K! [K000313234, digital image: JPS]).
Coccinia heterophylla Type: Angola. No detailed location, female, fl, no date, *F.M.J. Welwitsch 791* (Syntype: LISU! [LISU214548, digital image: JPS]).
Coccinia heterophylla Type: Angola. No detailed location, female, fr, Jan 1856, *F.M.J. Welwitsch 791* (Syntype: LISU! [LISU214549, digital image: JPS]).
Coccinia heterophylla Type: Angola. No detailed location, male, fl, no date, *F.M.J. Welwitsch 791* (Syntype: LISU! [LISU214550, digital image: JPS]). Type: Angola. In rugged places at Delamboa river, with *Coffea melanocarpa*, Sep 1855, *F.M.J. Welwitsch 791* (Syntype: LISU! [LISU214551, digital image: JPS]).
Coccinia heterophylla Type: Angola. At Delamboa river, no date, *F.M.J. Welwitsch 791* (Syntype: LISU! [LISU214552, digital image: JPS]).
Coccinia heterophylla Type: Angola. No detailed location and date, *F.M.J. Welwitsch 791* (Syntype: LISU! [LISU214553, digital image: JPS]).
Coccinia heterophylla Type: Angola. No detailed location and date, *F.M.J. Welwitsch 791* (Syntype: P! [digital image: JPS]).
Coccinia heterophylla Type: Angola. No detailed location and date, *F.M.J. Welwitsch 791* (Syntype: G-DC!).
Coccinia heterophylla *Physedra heterophylla var. hookeri* Hiern in Cat. Welw. Afr. Pl. 1(2): 400. 1898.
Coccinia heterophylla Type: Angola. [Cuanza Norte]: Golungo Alto, near Ponte de Felix Simões, female, fl, Dec 1855, *F.M.J. Welwitsch 792* (Holotype: BM! [BM000948009, digital image: BM]).

#### Description.

**Figure 28.**
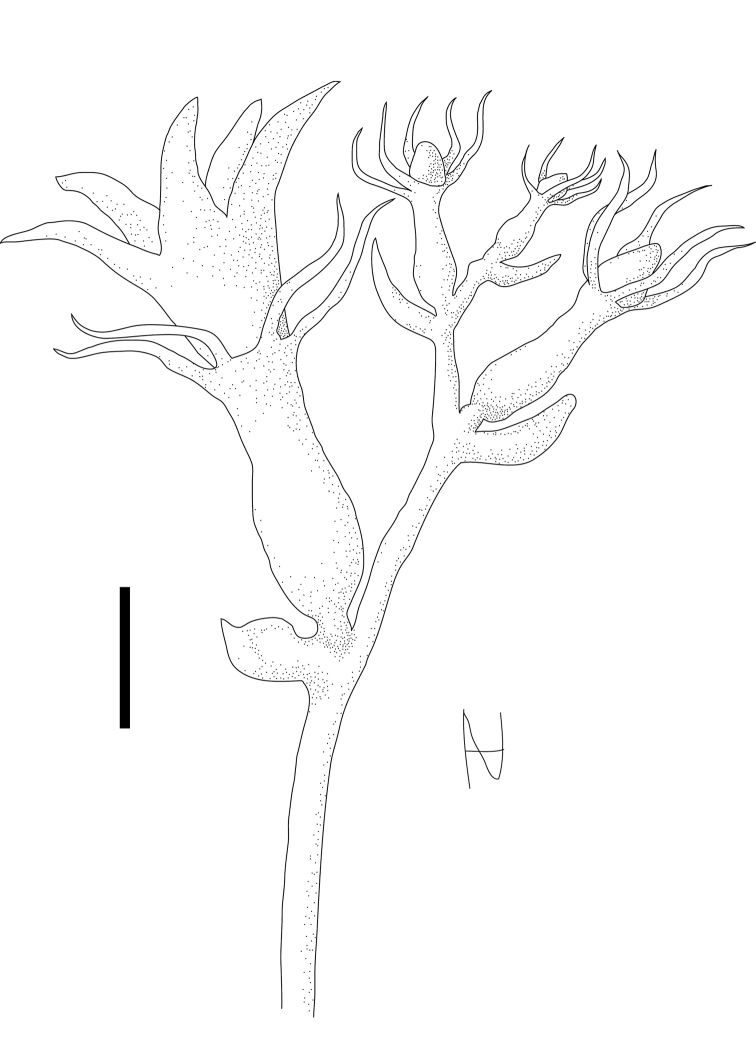
Reconstruction of the habit of a female raceme of *Coccinia heterophylla* based on *T.-J. Klaine 414* (P). Black bar equals 0.5 cm.

Perennial climber. Stem up to 5–6 m, glabrous, sometimes whitish-speckled. Petiole 1–2 cm long, puberulous on abaxial side with few tiny trichomes, rarely with up to 0.8 mm long, yellowish trichomes, sometimes white-speckled, on adaxial side with small, yellowish-dirty trichomes. Leaves 7.5–12.5 × 10–14 cm, cordate, deltoid-subhastate to 5-lobate, auriculate, or 7-lobate. Lobes triangulate to ovoid. Leaf margin dentate, often serrate. Apex acute with final tooth to acuminate. Upper leaf surface glabrous, small hyaline pustulate. Lower leaf surface glabrous often with blackish glands, nerves glabrous, except for basis with up to 0.8 mm long, yellowish trichomes and sometimes white-speckled. Probracts up to 5 mm. Tendrils bifid. Male flowers in short racemes. Common peduncle 2–13 mm long, not exceeding the pedicel bearing part in length, puberulous with tiny yellowish-dirty trichomes (magnifying glass!). Pedicels < 4 mm, glabrous to puberulous. Bracts up to 3.5 mm. Perianth tube glabrous. Calyx lobes 5–7 mm, subulate, erect. Corolla up to 1.6 cm, dirty yellowish, dirty orange to reddish-orange. Corolla lobes 4–7 mm. Color of filament column, anther head, or pollen sacs not seen. Female flowers solitary or in short or long (up to 15 cm) racemes (Fig. 28). Raceme and bracts as in males, except raceme can be elongated (see remarks). Hypanthium glabrous, calyx lobes and corolla like male flowers. Ovary glabrous. Style columnar to 3-parted to the middle, color not seen. Stigma bulging, pale yellow. Fruits up to 4–6 × 2–2.5 cm, ovoid-elliptical to shortly cylindrical, glabrous. Fruits ripen from green? via orange-colored with longitudinal green bands, ripe fruits unknown. Seed size not known, rather symmetrically obovate, face flat.

#### Phenology.

Flowering time: February–April, June, September–December.

#### Distribution.

**Figure 29.**
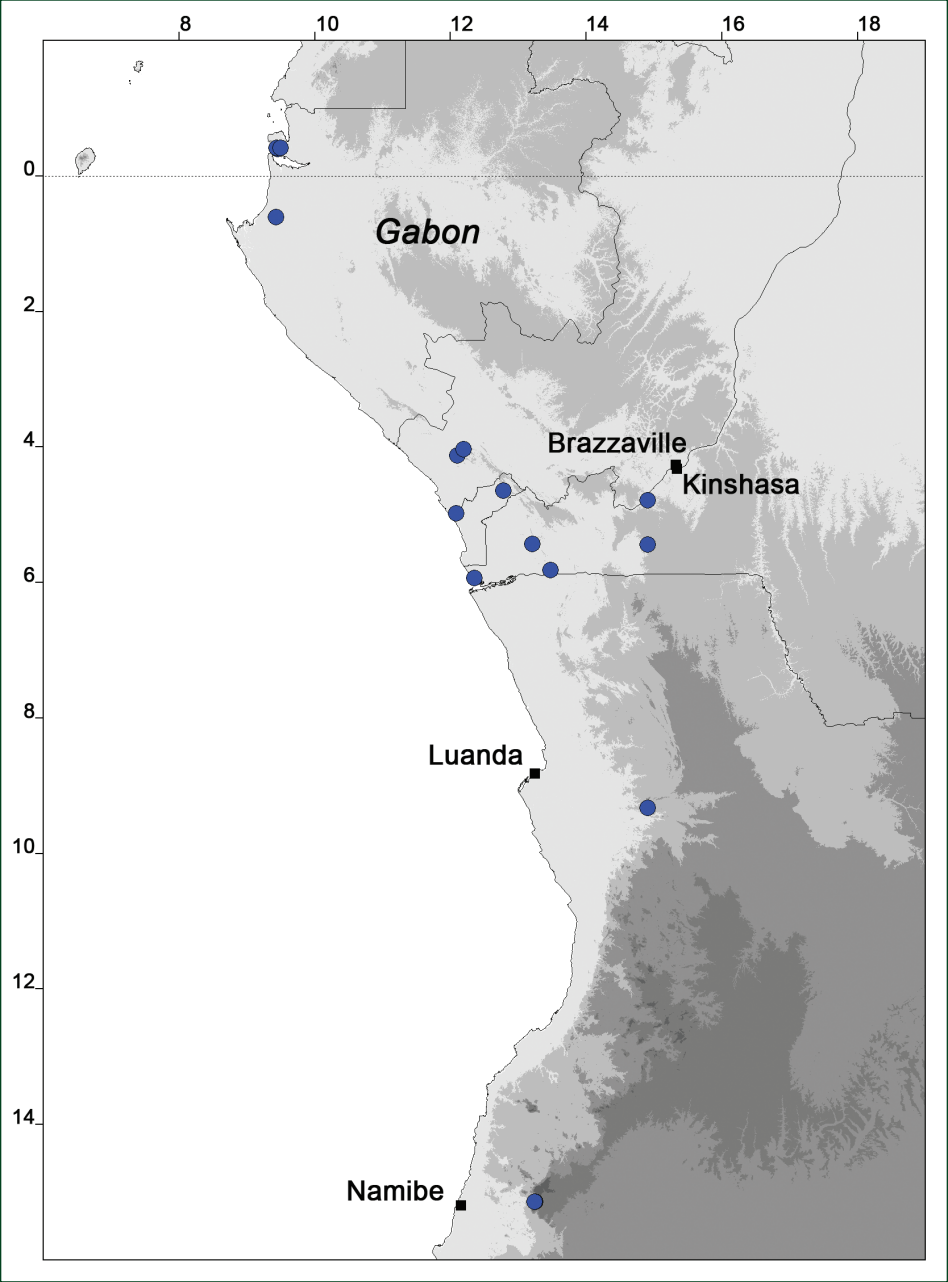
Distribution map of *Coccinia heterophylla* (based on 19 collections).

Fig. 29. Western Gabon (Estuaire), southern R. Congo (Kouilou), western D. R. Congo (Bas-Congo), western Angola (Cuanza Norte, Namibe). Elevation 10 to 900 m. Soil preference unknown. Transition between tropical lowland rainforest and woodlands, and its relict sites along the Angola highland escarpment, in secondary regrowths, on shrubs, along rivers.

#### Remarks.

The long, subulate calyx lobes are the only good character for distinguishing this species, which otherwise can be easily confused with *Coccinia barteri*. Female collections from Libreville (Gabon) and R. Congo have elongated racemes while the racemes are more condensed in the south. Whether this character shows affinity (introgression?) to *Coccinia racemiflora*, which also has elongated racemes, defines an own species, or is just a coincidental observation of intraspecific variation is not known.

#### Taxonomic remark.

This species is the type species of *Physedra* Hook.f. The genus was described by J. D. Hooker (Bentham and Hooker 1867) with three species belonging to it. However, in Oliver’s Flora of Tropical Africa (Hooker 1871), Hooker only describes two species, *Physedra heterophylla* and *Physedra longipes*. When Jeffrey (1962) transferred *Physedra longipes* into a new genus, *Ruthalicia*, he indirectly lectotypified *Physedra*.

Monique Kéraudren regarded *Physedra heterophylla* as synonymous to *Coccinia barteri* (Kéraudren-Aymonin 1975a; Kéraudren 1967) from which it, in fact, differs by the long calyx lobes. This, however, led to the creation of *Coccinia subhastata* with short calyx lobes, which is a synonym to *Coccinia barteri* (Holstein and Renner 2010).

#### Specimens examined.

(Selection; in total: 37) Angola. Cabinda: Maiombe [Forest], Belize, *J. Gossweiler 7653* (COI, K, LISU). Cuanza Norte: Cazengo municipality, near Agricultural Station Cazengo, *J. Gossweiler 5655* (COI, LISU [LISC 031407], LISC [LISC 031408], LISC [LISC 031409], LISC [LISC031410], LISC [LISC 031411], LISU]), and *5707* (COI, LISC [LISC 031412], LISU). Huíla: Châo da Chela, between [Lago] Tchivinguiro and Bruco, on middle slope of Serra da Chela escarpment, *E.J. Mendes 925* (BM, COI, LISC); ibid. *L.A. Grandvaux Barbosa 9448* (COI). D. R. Congo. Bas-Congo: Lukula territoire, Temvo, *F.M.C. Vermoesen 1824* (BR, EA, WAG [WAG0225504]). R. Congo. Kouilou: on left bank of Kouilou river, 4 km upstream of Kakamoeka, Sounda on path to level meter, *C. Farron 4980* (P [P05621154], P [P05621155], P [P05621156]). Gabon. Estuaire: Libreville, *T.-J. Klaine 414* (P [P05620605], P [P05620610], P [P05621146], P [P05621147], P [P05621149], P [P05621150], P [P05621152], P [P05621153]); Libreville, I.R.A.F. building, 0°25'N, 9°26'E, *J.M. Reitsma & B. Reitsma 2120* (MO, NY, WAG [WAG0225493]).

### 7. Coccinia hirtella

Taxon classificationPlantaeCucurbitalesCucurbitaceae

Cogn. in Schinz, Bull. Herb. Boiss. 4: 821. 1896.

Coccinia hirtella Type: [South Africa]. KwaZulu-Natal: Howick, 3400’, male, fl, 18 Feb 1895, *R. Schlechter 6775* (Lectotype, designated by Meeuse (1962: 101): Z! [Z-000004443, digital image: Z]; isolectotypes: BR! p.p. [BR0000008887184, digital image: BR, see taxonomic remarks], GRA [GRA0002851-0, digital image: JPS], K! [K000542639, digital image: K]).

#### Description.

Perennial creeper or climber. Stems up to 3 m, densely covered with long (> 0.5 mm), upright, whitish trichomes. Petioles 1.5–4 cm long, indumentum like on stem. Leaves 3–10 × 2.5–11 cm, 5-lobate. Lobes obovate or elliptical in outline, rarely ovate. Margin lobulate or coarsely serrate, (at maturity pale brownish) dentate (Fig. 13a). If margin serrate then lobe tips acute, else often rounded. Upper and lower leaf surface, esp. on the nerves, densely covered with long, upright, whitish trichomes. Probracts up to 3 mm. Tendrils simple or bifid. Male flowers solitary, rarely in few-flowered raceme. Common peduncle 1.8–4.5 cm, with white trichomes, pedicel of racemous flowers 0.2–3 cm, with white trichomes. Bracts up to 2 mm, caducous. Pedicel of solitary flowers 3–8.5 cm, with white trichomes. Perianth tube with white trichomes. Calyx lobes narrow triangulate to lineal, 3.5–6.5 mm long, spreading. Corolla 2–3.7 cm long, buff, lobes 1–2 cm. Filament column white, anther head pale yellowish, pollen sacs yellow to orange. Female flowers one solitary. Pedicel 0.5–5.5 cm, with long, white trichomes. Hypanthium with white trichomes, calyx lobes and corolla like in male flowers. Style columnar, whitish. Stigma bulging, yellow. Ovary with some long trichomes. Fruit oblong ovoid, 5–6 cm × 2.5–3 cm, sparsely covered with long trichomes to glabrous, ripening from green via green with longitudinal whitish mottling, via yellow, orange to red when ripe (Fig. 13a). Seeds 5.5–6.5 × 3–3.5 mm (L/W), seed height not seen, rather symmetrically obovate, face flatly lenticular.

#### Phenology.

Flowering time: January–April, September, November, December.

#### Distribution.

**Figure 30.**
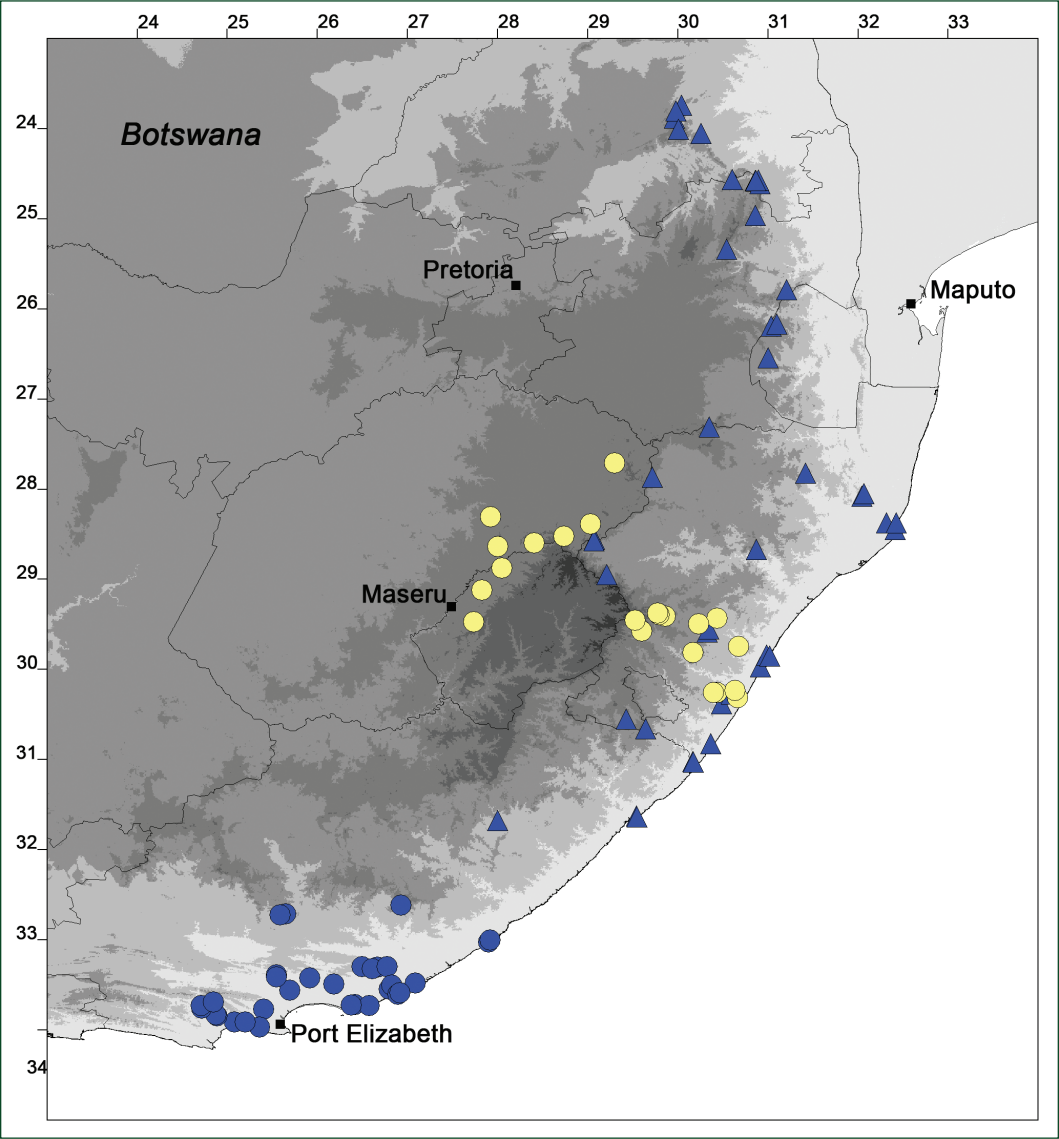
Distribution map of *Coccinia hirtella* (pale yellow dots; based on 22 collections), *Coccinia mackenii* (blue triangles; based on 50 collections), and *Coccinia quinqueloba* (blue dots; based on 38 collections). For South Africa the borders of the provinces are given.

Fig. 30. Lesotho, South Africa (S KwaZulu-Natal, SE Free State). Afromontane scrubland and sourveld grassland. Elevation 110–1900 m. On sand, sandstone, loam, well-drained soils, full sun, grazing maybe tolerated.

#### Use.

It is said that if a Masuto dreams unpleasantly about an ancestor (“balimo”), then relief is given after a bath with sun-dried *Coccinia hirtella* roots and ironstone in a hole on the threshold of the “lelopa” (the circular fence around the hut) (Phillips 1933).

#### Vernacular names.

Sotho: leraka-la-balimo (Jacot Guillarmod 1971; Phillips 1933), Sotho: monyaku (Jacot Guillarmod 1971).

#### Taxonomic remarks.

The BR type specimen (BR0000008887184) contains two labels and is mixed. The female parts on the sheets have most likely been detached from the lectotype in Z, because it is a female branch with shallowly lobate leaves, just as part of the lectotype. The male parts on the BR specimen, however, are mixed. The leaves with the obtuse lobules are also likely type material, whereas the leaf with the acute lobules is very similar to the leaves of the *R. Schlechter 6708* collection, which is not a type.

#### Specimens examined.

(Selection; in total: 36). Lesotho. Leribe: Léribé [Hlotse], *H. Dieterlen & A. Dieterlen 145* (BR [BR0000008887467], P [P05620661], P [P05620662], Z). Maseru: Roma, Map[h]otong, *M. Schmitz 8039* (PRE). South Africa. Free State: Rooiberge area, Ross Kloof, *M. Jacobs 8565* (L, LISU, PRE). KwaZulu-Natal: 17 km from Nottingham Road on road to Loteni, *E. Retief 1638* (MO, PRE); Albert Falls, *A.K. Meebold 13160* (M); Giants Castle Game Reserve (Dinosaur Footprint area), *W.R. Trauseld 930* (PRE).

### 8. Coccinia intermedia

Taxon classificationPlantaeCucurbitalesCucurbitaceae

Holstein, PhytoKeys 7: 28. 2011.

Coccinia intermedia Type: Benin. Atakora: Natitingou, Kouaténa (Perma), 10°12.00'N; 1°30.18'E, river bed, female, fl, fr, 3 Oct 2000, *A. Akoègninou et al. 3625* (Holotype: WAG! [WAG0278370]; isotype: WAG [WAG0278369]).
Coccinia intermedia Type: Ghana. Greater Accra: Shai Hills Game Reserve, monoecious, fl, fr, 25 May 1976, *J.B. Hall & J.M. Lock GC 46016* (Paratypes: K! (4), MO!).
Coccinia intermedia Type: Ivory Coast. Zanzan: Bouna, male, fl, 10 Aug 1967, *C. Geerling & J. Bokdam 662* (Paratypes: MO!, WAG! [WAG0225492]).
Coccinia intermedia Type: Togo. Maritime: between Lomé and Aného, female, fr, 25 Jun 1994, *L. Aké Assi 18983* [typographical error in orig. publication stated 18982] (Paratype: MO!).

#### Description.

Perennial climber. Stem length unknown, but likely several meters, glabrous, at maturity with clear to white pustules. Petioles 2.8–10.8 cm, glabrous, when older with clear to white pustules. Leaves 6–15 × 7–18 cm, shallowly to profoundly 5-lobate, more or less auriculate. Margin conspicuously dentate, blackening when dried. Apex acute. Upper leaf surface glabrous with clear to whitish pustules. Lower leaf surface glabrous, often with small dark glands near the leaf base. Probracts up to 2.5 mm long. Tendrils simple or bifid. Male flowers in few-flowered racemes, likely sometimes accompanied by a single flower. Common peduncle up to 1 cm, pedicels in racemose flowers 2–4 mm, each glabrous. Bracts up to 1.5 mm. Perianth tube glabrous, calyx lobes c. 1.5 mm, lineal to narrowly triangulate, erect with slightly recurved tips. Corolla 1.6 cm long, pale reddish-yellow to yellow, lobes 0.7 cm. Filament column and anther head not seen, pollen sacs yellowish. Female flowers 1–3 clustered (strongly reduced raceme). Pedicels 0.6–1.2 cm, glabrous. Hypanthium tube glabrous, calyx lobes and corolla like in male flowers. Ovary glabrous. Style and stigmas not seen. Fruit 4.5 × 2.5 cm, elliptical to oblong. Unripe fruit green with pale green longitudinal mottling, ripe orange?, more likely becoming red via orange ripening stage. Size of mature seeds unknown (≥ 5.5 × 3.5 × 1.3 mm (L/W/H)), symmetrically (to slightly asymmetrically) obovate, face flat.

#### Phenology.

Flowering time: May, August, October.

#### Distribution.

**Figure 31.**
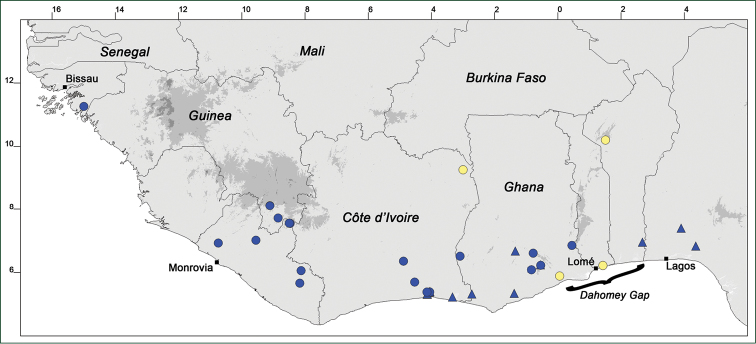
Distribution map of *Coccinia intermedia* (pale yellow dots; based on 4 collections), *Coccinia keayana* (blue dots; based on 23 collections), and *Coccinia longicarpa* (blue triangles; based on 22 collections).

Fig. 31. NE Ivory Coast, SE Ghana (likely also in the north), S Togo (likely also in the north), NW Benin. Elevation sea level to 415 m. Wooded grasslands (semi-humid savanna), woodlands, dry forests, in riverbeds.

#### Remarks.

This species is rather cryptic and imperfectly known. The leaves seem to develop conspicuous margin teeth during maturity, like e.g., *Coccinia grandis*, but the darkish sublaminal glands differ from that species. The erect calyx lobes with slightly recurved tips appear to be the most indicative character for *Coccinia intermedia*. The clustered female flowers and the fruits link to *Coccinia barteri*, from which it, among other characters, differs in ecology. Two *J.B. Hall & J.M. Lock GC 46016* specimens from K have male and female flowers/fruits on one twig and are thus monoecious. As all other *Coccinia intermedia* collections are dioecious, this could be a case of “leaky dioecy” (Baker and Cox 1984), which also has been observed in other Cucurbitaceae (Schaefer and Renner 2010).

### 9. Coccinia keayana

Taxon classificationPlantaeCucurbitalesCucurbitaceae

R.Fern., Bol. Soc. Brot. ser. 2, 33: 191. 1959.

Coccinia keayana Type: Guinea-Bissau. Tombali: region Cacine, rainforest, fl, Aug 1933, *J.V.G. do Espírito Santo 603* (Holotype: COI).
Coccinia keayana Type: ibid., *J.V.G. do Espírito Santo 631* (Paratypes: LISC [LISC 011640, digital image: IICT, JPS], LISC! [LISC 011641, digital image: IICT, JPS], LISC [LISC 011642, digital image: IICT, JPS], LISJC).
Coccinia keayana Type: Guinea-Bissau. Tombali: between Cacine and Guileje, plantation, 1 Aug 1945, *J.V.G. do Espírito Santo 2151* (Paratypes: COI, LISC [LISC 002508, digital image: IICT, JPS], LISC! [LISC 002509, digital image: IICT, JPS]).
Coccinia keayana Type: Liberia. [Margibi County]: Firestone plantation, at Du River, 29 Jul 1926, *D.H. Linder 121* (Paratype: K!).
Coccinia keayana Type: Sierra Leone. Jigaya, c. 350 m, 28 Sep 1914, *W. Thomas 2844* (Paratype: K!).
Coccinia keayana Type: Sierra Leone. [Northern Province]: Bumban National Park, 30 Aug 1928, *F.C. Deighton 1221* (Paratype: K!).
Coccinia keayana Type: Sierra Leone. [Southern Province]: Moyamba District, Moyamba, 25 Aug 1931, *F.C. Deighton 2217* (Paratype: K!).
Coccinia sp. A Keay, Fl. W. Trop. Afr. 1: 216. 1954.

#### Description.

**Figure 32.**
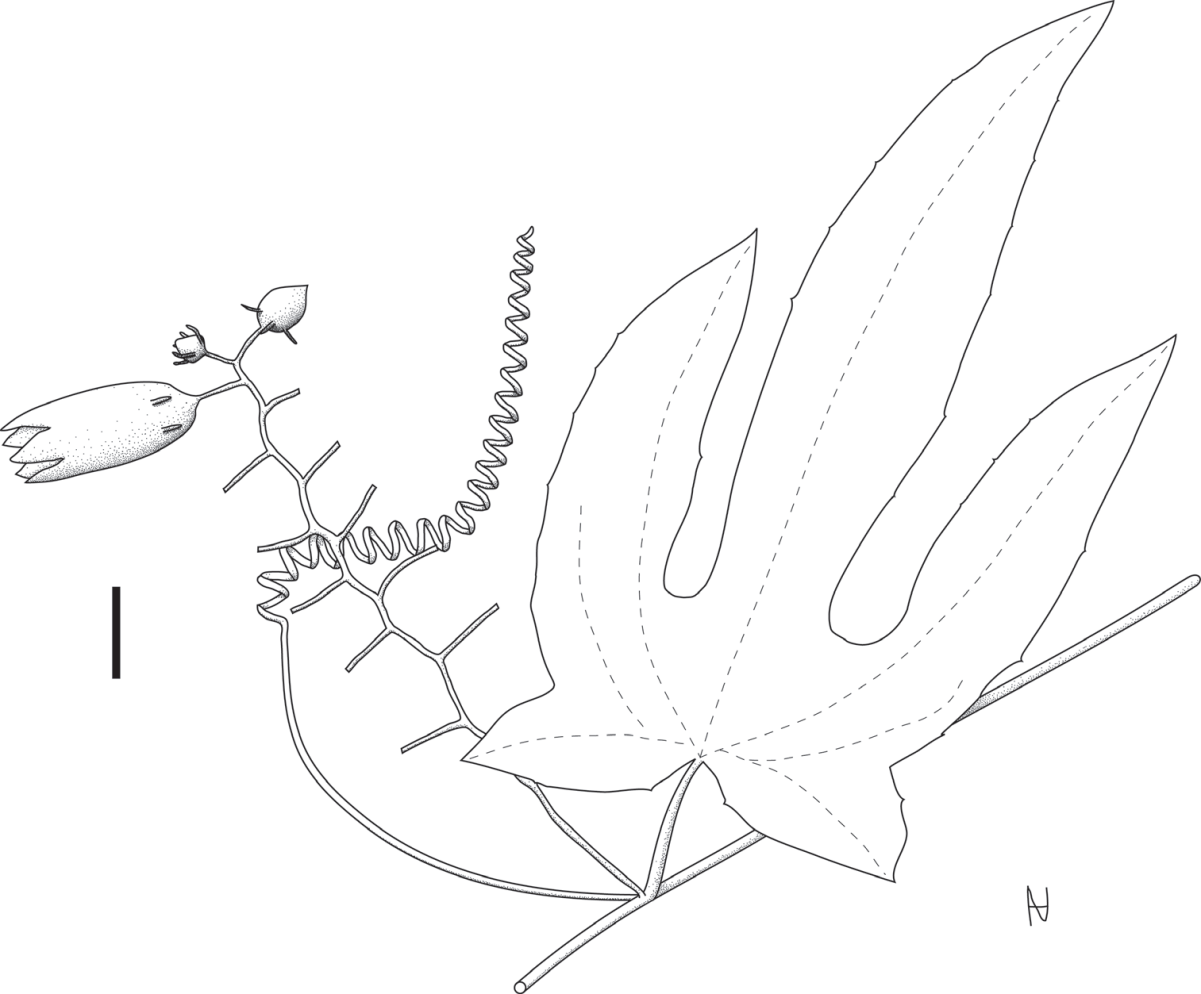
Reconstruction of the habit of *Coccinia keayana* based on *C.C.H. Jongkind et al. 6542* (WAG). Black bar equals 1 cm.

Perennial? climber. Stem up to 5 m, glabrous. Petiole 1.5–5 cm, with short, few-cellular trichomes on adaxial side, glabrous on abaxial side. Leaves 5–11 × 3.5–11 cm, (shallowly to) profoundly 3-(or 5-)lobate, auriculate, rarely long cordate. Margin rather remotely dentate to slightly serrate. Lobe apex acute or subacute with final tooth. Upper leaf surface tiny hyaline pustulate. Lower leaf surface with blackish glands, dried often with bluish-green tinge, glabrous or rarely with soft multicellular trichomes on nerves. Probracts up to 3 mm. Tendrils simple. Male flowers ebracteate, in lax racemes with up to 20 flowers, sometimes accompanied by a solitary flower (Fig. 32). Common peduncle up to 1.7 cm, shorter than racemous part, glabrous. Pedicels of racemous flowers up to 1 cm, pedicels of solitary flowers up to 1.5 cm long, each glabrous. Perianth tube glabrous. Calyx lobes linear, 2.5–3 mm, in buds spreading, later reflexed. Corolla 1.7–2 cm long, white, yellow, dirty orange, salmon to dull pinkish. Corolla lobes 3–5 mm long. Filament column and anther head not seen, pollen sacs pink–orange. Female flowers solitary or in few-flowered lax racemes. Common peduncle 1.2–2.1 cm, glabrous. Pedicel flowers in racemes up to 1 cm, glabrous, pedicels of solitary flowers up to 2.7 cm, glabrous. Hypanthium glabrous, calyx lobes and corolla as in male flowers. Ovary glabrous. Style and stigma not seen. Fruits 2–3 × 2 cm, subglobose to globose, unripe glaucous green, ripening via yellow to pinkish [rather glaucous?] red. Seeds 4.5 × 2.5 × ? mm (L/W/H), rather symmetrically obovate, face flatly lenticular.

#### Phenology.

Flowering time: March to November.

#### Distribution.

Fig. 31. Tropical West Africa: Guinea-Bissau (Tombali), Sierra Leone, Liberia, Guinea (Nzérékoré), S Ivory Coast, S Ghana, S Togo? Elevation sea level to 1250 m. On sandy soil, lateritic soils. Tropical rainforest, in high trees, high bushes, on roadsides.

#### Vernacular names.

Cf. Koranko: nala (*W. Thomas 2844*), Limba: ngolibwe (*W. Thomas 2844*), Mende: ndogbo-gojai (*F.C. Deighton 2217*), Temne: efosa (*W. Thomas 2844*).

#### Remarks.

The long racemes with ebracteate flowers and the linear, reflexed calyx lobes are good characters for this species. It is barely distinguishable from *Coccinia barteri* without flowers. *Coccinia keayana* collections often have a bluish green tinge and the lobes conspicuously point forwards (see Fig. 32), which might only rarely occur in *Coccinia barteri*. The corolla is rather tubular, sometimes somewhat inflated.

#### Taxonomic remarks.

The name *Coccinia keayana* R.Fern. is misapplied for the Flora of Cameroon or western Central Africa in general (for details, see taxonomical remarks of *Coccinia barteri*).

#### Specimens examined.

(Selection; in total: 33) Ghana. Eastern Region: Asiakwa district, Atewa Range Forest Reserve, Accra-Kumasi highway 5–6 km along forest road that intersects the highway at Sagyimase village, 6°13'48"N, 0°32'42"W, *M. Merello et al. 1179* (MO). Volta: Amedzofe, *J.B. Hall GC40053* (P [P05620653]). Western Region: Bia National Park, *J.B. Hall & J.M. Lock GC46493* (WAG [WAG0046501]). Guinea. Nzérékoré: Nzérékoré Préfecture, [WSW of] Nzérékoré, 7°43'35.54"N, 8°51'21.28"W, *E. Achigan-Dako 07 NIA 917* (GAT). Guinea-Bissau. Tombali: Bedanda sector, Cantanhez, *J. Alves Pereira 3172* (H, LISC). Ivory Coast. Lacs: Oroumba Boca [Orombo Bocca Mt.], *H.C.D. de Wit 5772* (WAG [WAG0234139], WAG [WAG0234140]). Lagunes: Abidjan, Banco Forest Reserve, *W.J.J.O. de Wilde 893* (BR, EA, WAG [WAG0044624], WAG [WAG0044625], Z). Liberia. Bong: 3 miles NE of Suacoco, *Z.D. Traub 256* (BR, G, MO). Nimba: Yéképa, Mt Nimba, *J.-G. Adam 21232* (MO, P [P00694038/P05590407], P [P05620652], PRE).

### 10. Coccinia longicarpa

Taxon classificationPlantaeCucurbitalesCucurbitaceae

Jongkind, Blumea 49: 83. 2004.

Coccinia longicarpa Type: Ivory Coast. Lagunes: Forêt du Banco, S of Arboretum, near river, male and female, fr, 20 Jul 1973, *J. de Koning 1965* (Holotype: WAG! [WAG0099441, digital image: JPS], isotype: K! [K000035540, digital image: K]).
Coccinia longicarpa Type: Ivory Coast. Lagunes: Forêt du Banco, Route Martineau, secondary forest, 10 Oct 1974, *J. de Koning 4077* (Paratype: WAG! [WAG0099430]).
Coccinia longicarpa Type: Ivory Coast. Lagunes: Forêt du Banco, near swampy secondary forest, 8 Aug 1975, *J. de Koning 5904* (Paratypes: K [K000035541], MO!, WAG! [WAG0099438, digital image: JPS]).
Coccinia longicarpa Type: Ivory Coast. Lagunes: Forêt du Banco, N of center, near Banco river, in forest clearing on clear spot, 16 Jun 1975, *W.J. van der Burg 551* (Paratype: WAG! [WAG0062627]).
Coccinia longicarpa Type: Ivory Coast. Lagunes: near Abidjan, 6 Sep 1967, *C. Geerling & J. Bokdam 829* (Paratypes: WAG! [WAG0011282], WAG! [WAG0011283]).
Coccinia longicarpa Type: Ivory Coast. Lagunes: [W of Abidjan], Adiopodomé [Adiopo-Doumé], margin of bush pathway, 3 Aug 1956, *J.J.F.E. de Wilde 183* (Paratypes: WAG! [WAG0044616], WAG! [WAG0044617], WAG! [WAG0044618], WAG! [WAG0044619]).
Coccinia longicarpa Type: Ghana. Eastern Region: near Kibi, Atewa Range Forest Reserve, Jun 1976, *J.M. Lock GC 43991* (Paratype: K).
Coccinia longicarpa Type: Liberia. Gbapolu/Lofa: Gbanga, Sep 1926, *D.H. Linder 576* (Paratype: K).
Coccinia longicarpa Type: Ghana. Nsuta, no detailed location, 1500 ft, May 1929, *C. Vigne 1735* (Paratypes: K, P!).
Coccinia sp. A C.Jeffrey, Key to the Cucurbitaceae of West Tropical Africa. J. W. African Sci. Assoc. 9: 87 p.p., 1964.
Coccinia sp. B Keay, Fl. W. Trop. Afr. 1(1): 216 p.p., 1954. Nigeria. [Ogun]: Ijebu-Ode District, toward the head of extraction Rd. from Grace Camp [in Omo Forest Reserve] westwards toward the R. Omo [Shasha], about 1.5 mls from the Grace Camp, 2 May 1946, *S. Tamajong FHI 16938* (K!); *D.H. Linder 576* (K); *C. Vigne 1735* (K, P!).
Coccinia sp. D Keay, Fl. W. Trop. Afr. 1(1): 216 p.p., 1954. Nigeria. [Oyo]: Lagos [Colony], Ibadan forest, 1 Dec 1900, *C. Punch 46* (K!).

#### Description.

Perennial climber. Stems up to 5 m, glabrous. Petiole 0.9–4.5 cm, glabrous. Leaves 7–12 cm × 2.5–11 cm, 5-angularly subcordate, subhastate, rarely 3-lobate, auriculate. Lobes elliptical. Margin suspiciously dentate, whitish in living state, blackening when dried. Apex acute with final tooth. Upper leaf surface pale to white pustulate. Lower leaf surface glabrous, nerves often white-speckled. Probracts < 1 mm, often missing. Tendrils simple, rarely bifid. Male flowers ebracteate, in glabrous racemes, occasionally accompanied a solitary flower. Common peduncle 0.7–1.5 cm, pedicel of flowers in raceme 2.5–8 mm, pedicel of solitary flower 5–10 mm. Perianth tube glabrous, calyx lobes 2–3 mm long, (0.75–)1.2–1.8 mm broad at base, erect. Corolla 0.8–1.5 cm long, yellow to (pale) orange, sometimes with greenish nerves. Corolla lobes up to 2 mm long. Filament column and anther head not seen, pollen sacs yellow to orange. Female flower solitary. Petiole 1.5–2.2 cm, glabrous. Hypanthium glabrous, calyx lobes and corolla not seen, but likely as in male plants. Ovary glabrous. Style and stigmas not seen. Fruit up to 20 cm long, c. 1 cm in diam., long cylindrical, unripe waxy green, ripe unknown. Seed size and shape unknown, face flat.

#### Phenology.

Flowering time: May–December.

#### Distribution.

Fig. 31. Tropical West Africa: SW Ivory Coast, S Ghana, SE Benin, SW Nigeria. Elevation sea level to 460 m. Soil preference not known. In primary or secondary forests, in disturbed places (roadsides, near rivers).

#### Remarks.

The broad calyx lobes are, apart from the long cylindrical fruit, the best character for identifying this species. An urceolate corolla (Jongkind 2004) also occurs in *Coccinia barteri*.

A single collection (*H.J. Beentje 602* from M) mentions a lilac corolla color, which would be unique in *Coccinia*. Although this might be possible, since there are also pinkish flowers reported in *Coccinia adoensis*, the fact that the WAG duplicate with the same collection number is a *Ruthalicia* makes it more likely that the observation is due to a mixed collection, eventually from a Convolvulaceae.

#### Specimens examined.

(Selection; in total: 24) Benin. Plateau: Pobe, 6°57'52.56"N, 2°40'19.70"E, *E. Achigan-Dako 07 NIA 731* (GAT). Ghana. Ashanti: Bobiri Forest Reserve, 6°41'N, 1°21'W, *C.C.H. Jongkind 3970* (WAG [WAG0023747], WAG [WAG0023748]). Central Region: Twifo/Hemang/Lower Denkyira District, Kakum, 5°20'54.31"N, 1°23'1.39"W, *E. Achigan-Dako 07 NIA 734* (GAT). Eastern Region: Atewa Range Forest Reserve, 06°15'N, 00°33'W, *C.C.H. Jongkind et al. 1538* (MO, WAG [WAG0020265]). Western Region: Jomoro District, Fawoman, 5°19'32.63"N, 2°43'28.13"W, *E. Achigan-Dako 06 NIA 050* (GAT); ibid., *E. Achigan-Dako 06 NIA 051* (GAT). Ivory Coast. Lagunes: Abidjan. Banco Forest Reserve, *J.J. Wieringa 5386* (WAG [WAG015997]). Sud-Comoé: Aboisso, 5 km NNW of Nganda-Nganda [Forest Reserve], near lagoon Aghien [lagune Aby?, because lagune Aguien is NE of Abidjan], 5°14'N, 3°20'W, *H.J. Beentje 602* (M, but not WAG). Nigeria. Ogun: Ijebu-Ode District, Omo Forest Reserve, Compartment 8, *J.R. Charter FHI 38635* (K).

### 11. Coccinia mackenii

Taxon classificationPlantaeCucurbitalesCucurbitaceae

Naudin ex C.Huber [sphalm. Mac-Kennii, after John M’Ken, ICN 60C.5], Cat. Print.: 5. 1865.

Cephalandra mackenii (Naudin ex C.Huber) Naudin [sphalm. mac kennii], Ann. Sci. Nat. Bot. 5: 17, ser. 5. 1866.
Coccinia mackenii Type: Cultivated. Cultivated in Paris Botanical Garden from seeds from Huber’s Garden in Olbia [Hyères, France] who obtained the seeds from M’Ken from near Port Natal [Durban, KwaZulu-Natal, South Africa], female, fl, 1864, *C.V. Naudin s.n.* (Syntypes: P!, G-DC! [G00211343, digital image: G]), P [P06745731, digital image: P], P [P06745732, digital image: P].
Coccinia mackenii Type: Cultivated. Cultivated in Paris Botanical Garden from seeds from Huber’s Garden in Olbia [Hyères, France] who obtained the seeds from M’Ken from near Port Natal [Durban, KwaZulu-Natal, South Africa], 1863, *C.V. Naudin s.n.* (Syntypes: P! [P06745737, digital image: P], P! [P06745739, digital image: P], P [P06745740, digital image: P]).
Coccinia mackenii Type: Cultivated. Cultivated in Huber’s Garden in Olbia [Hyères, France] who obtained the seeds from M’Ken from near Port Natal [Durban, KwaZulu-Natal, South Africa], male and female, fl, 1864, *C.V. Naudin s.n.* (Lectotype, designated here: P! [P06745735, digital image: P; K neg. 2993]; isolectotypes: G-DC! [3 sheets, all G00211344, digital images: G], K! [K000542637, digital image: K], K! [K000542638, digital image: K], K (2)!, P! [P06745730, digital image: P], P! [P06745733, digital image: P]).
Coccinia palmata (Sond.) Cogn. in A.DC. & C.DC., Monogr. Phan. 3: 540. 1881. Nom. illeg. *Momordica palmata* E.Mey. ex Drège, Zwei pflanzengeogr. Dokum.[addition to Flora 26(2)]: 156, 159, 202. 1843. Nom. nud. *Cephalandra palmata* Sond. in Harv. & Sond., Fl. Cap. 2: 493. 1862.
Coccinia mackenii Type: [South Africa]. [KwaZulu-Natal]: near Port Natal [Durban; cited in l.c. p. 159], male and female, fl, fr, 7 Apr 1832, *J.F. Drège s.n.* (Lectotype, designated by Meeuse (1962: 96): S! [S08-12155, digital image: S], isolectotype: P! [P00748835, digital image: P]).
Coccinia mackenii Type: [South Africa]. Without location and date, male, fl, *J.F. Drège s.n.* (Syntypes: G! [G00226835, digital image: G], HBG! [HBG506427], K! [K000313198, digital image: K], K! [K000313199, digital image: K], L!, P! [P00748833, digital image: P], P!, PRC!, W! [W 0026939, digital image: WU]).
Coccinia mackenii Type: [South Africa]. [KwaZulu-Natal]: Omsamculo [Umzimkulu], between shrubs and thickets, near river mouth, female, fr, 5 Mar 1832, *J.F. Drège 4637* (Syntype: P! [P00748834, digital image: P])
Coccinia dinteri André, Rev. hort. [Paris] 72: 276. 1900.
Coccinia mackenii Type: Unnumbered plate in l.c.

#### Description.

Perennial climber or creeper. Stems up to 9.5 m, glabrous. Petioles 0.7–11 cm long, glabrous or with thin trichomes. Leaves 3–13.5 × 3–15.5 cm, shallowly to profoundly 5-lobate, in the latter case often weakly lobulate. Lobes triangulate, lanceolate, ovate to obovate. Margin smooth, dentate, sometimes serrate to lobulate, esp. towards the apex. Apex acute with final tooth. Upper leaf surface glabrous with clear to white pustules, rarely with few trichomes. Lower leaf surface glabrous or with thin, stiff or articulate trichomes, towards base usually with glands. Probracts up to 4 mm, oblong-lanceolate. Tendrils bifid, rarely simple. Male flowers solitary or ebracteate in few-flowered racemes. Common peduncle 5–6.5 cm, pedicel of flower in raceme up to 2.5 cm, pedicel of solitary flowers 6–9 cm, all glabrous, rarely with long trichomes. Perianth tube glabrous. Calyx lobes 1.5–6.5 mm, lineal, subulate to narrowly triangulate, when young erect, later spreading to reflexed. Corolla 1.3–2.7 cm long, cream to pale buff, corolla lobes subulate to triangulate, 0.7–1.1 cm. Filament column, anther head, and pollen sacs not seen. Female flowers one solitary. Pedicel 0.7–5 cm long, glabrous. Hypanthium glabrous, calyx and corolla like in male flowers. Ovary glabrous. Style columnar, color not seen. Stigma bulging, color not seen. Fruits elliptical to oblong, c. 10 × 2–2.5 cm. Unripe green with white mottling, ripe red-orange to red, sometimes? with white mottling. Seeds 6–7 × 4–4.5 × 1.5 mm (L/W/H), slightly asymmetrically obovate, face flatly lenticular.

#### Phenology.

Flowering time: January–April, July, November, December.

#### Distribution.

Fig. 30. South Africa (E Eastern Cape, KwaZulu-Natal, Mpumalanga, Limpopo), Swaziland. Elevation sea level to 1750 m. Clay, Berea red sand, sandstone, quartzite, poorly drained soils. Afromontane forests, coastal forests, littoral forests, forest margins, sometimes grassland. In frost-free areas (Meeuse 1962).

#### Use.

Leaves and fruits are eaten by Tsonga people (Shackleton et al. 1998).

#### Vernacular names.

Xitsonga: Gomo, XipapaXipapana (Shackleton et al. 1998).

#### Remarks.

Some collections with deeply lobate leaves and short petioles resemble the closely related *Coccinia quinqueloba*, and some *Coccinia quinqueloba* individuals have long petioles (*C.V. Naudin s.n. 1863*, *C.V. Naudin s.n. 1863–1865*, *E. Retief 1215*). However, Naudin (1866) reports considerable problems with seed production in interspecific crosses. It would be desirable to validate this observation.

#### Taxonomic remarks.

The initial designation of the *Coccinia mackenii* lectotype (Holstein and Renner 2010: 440) is not valid, because it erroneously designated a female specimen from Paris Botanical Garden. However, Naudin stated that all plants from Paris Botanical Garden were male (P06745733), so the former designation was ambiguous. The new lectotype was chosen from Olbia [Hyères] material. In contrast to Naudin’s statement, that Olbia material was female, there is a male K specimen (K000542638), from Huber’s Garden in Olbia. Eventually, this specimen is from Paris Botanical Garden but incorrectly labeled, because the lack of the opposite sex affected Naudin’s crossing experiments.

Due to an overlooked published combination *Coccinia mackenii* bore the illegitimate name *Coccinia palmata* for more than 120 years. When Wight and Arnott published *Coccinia indica* they also included a specimen tentatively identified as *Bryonia palmata* L. Although without relevance for the genus *Coccinia* itself, it lead to further complications. One year after Voigt’s publication of the correct combination *Coccinia grandis*, Roemer (1846) also recognized the apparently missing combination and that Linnaeus’ *Bryonia palmata* and *Bryonia grandis* indeed referred to different species. Roemer treated them, amongst other species, as *Coccinia grandis* (L.) M.Roem. (nom. illeg.) and *Coccinia palmata* (L.) M.Roem. In addition to the name *Coccinia palmata* (L.) M.Roem. another species from South Africa was described with the name *Cephalandra palmata* E.Mey. ex Sond. (Harvey and Sonder 1862). Cogniaux (1881) accepted this species in *Coccinia*, overlooking *Coccinia palmata* (L.) M.Roem. He thus created an illegitimate *Coccinia palmata* (E.Mey. ex Sond.) Cogn., which has since been used for this species. Holstein and Renner (2010) called attention to this erroneous usage by resurrecting the correct name, *Coccinia mackenii* Naudin ex C.Huber.

The drawing of *Coccinia dinteri* in the protologue shows a bifid tendril. Since all other characters match *Coccinia mackenii* and the resemblance was already discussed in the protologue, it is feasible to synonymize it with that species. M. Proschowsky grew this plant in the Fabron quarter of Nice, France, but the origin of the seeds was not indicated. The label named it “*Coccinia dinteri*” after Moritz Kurt Dinter (in the protologue erroneously spelled as “Hurt Dinter”), who was curator in La Mortola (Giardini Botanici Hanbury, Liguria, Italy) where many South African plants were cultivated. Hence, it is reasonable to assume this origin as done by André there, which again would match *Coccinia mackenii*. There is a specimen in K herbarium containing only seeds and a label indicating that they were sent from Hanbury, La Mortola in 1897. A note mentions that the seeds were sown in Kew Gardens. The identification is given as *Cephalandra mackenii* with a question mark and a later note with the *Coccinia dinteri* citation. It is plausible to assume that these seeds come from the same plant stock that was used to grow and to describe *Coccinia dinteri*. Although the seeds fit the description of *Coccinia mackenii* seeds, it is not possible to use them to identify the species unambiguously.

#### Specimens examined.

(Selection, in total: 71) South Africa. Eastern Cape: Port St. Johns, Jan 1933, *A.O.D. Mogg s.n.* (L, PRE [PRE 42990], Z). Kwazulu-Natal: Durban district, Isipingo North, *C.J. Ward 3747* (COI, PRE); Umzinto district, Vernon Crookes Nature Reserve, far end of Golden Valley, *K. Balkwill et al. 10930* (E [E00264193], MO); Pietermaritzburg, Ferncliffe Forest, *J. Bodenstein 92* (PRE); Nkandlha [Nkandla], Qudeni Forest, 5 mls [8 km] S of Qudeni P.O., *L.E.W. Codd 6991* (PRE). Limpopo: near Lydenburg, near Echo Cave, *R.G. Strey 3762* (M, PRE, WAG [WAG0234163]); Woodbush [Forest Reserve], 6 Aug 1925, *A.O.D. Mogg s.n.* (COI, L, PRE [PRE 43066], Z). Mpumalanga: Letaba district, E side of shoulder extending northwards from ridge above Weltevreden, *J.C. Scheepers 1110* (M, PRE). Swaziland. Hhohho: about 20 km N of Mbabane, Ngwenya Hills, Castle peak, north slopes, *B. Maguire 7553* (B [B 10 0019799], E). Manzini: Usutu Forests, *R.H. Compton 32287* (PRE).

### 12. Coccinia megarrhiza

Taxon classificationPlantaeCucurbitalesCucurbitaceae

C.Jeffrey, Kew Bull. 15(3): 347. 1962.

Coccinia megarrhiza Type: Kenya. Northern Province [Eastern Province]: Moyale, 3800 ft, male, fl, 28 Apr 1952, *J.B. Gillett 12967* (Holotype: K! [K000313235, digital image: JPS, K], isotypes: B! [B 10 0154926, digital image: B, JPS], S! [S08-12479]).
Coccinia megarrhiza Type: Kenya. Northern Province [North Eastern Province]: [western Mandera District], Dandu, 2600 ft, 10 Apr 1952, *J.B. Gillett 12759* (Paratype: K! [K000354130]).
Coccinia megarrhiza Type: Kenya. Northern Province [North Eastern Province]: [western Mandera District], Dandu, 2700 ft, 9 May 1952, *J.B. Gillett 13122* (Paratypes: EA!, K! [K000354129]).
Coccinia megarrhiza Type: Kenya. Northern Province [North Eastern Province]: [western Mandera District], Dandu, 3000 ft, 14 May 1952, *J.B. Gillett 13191* (Paratypes: EA!, K!).
Coccinia megarrhiza Type: Kenya. Northern Province [Eastern Province]: Moyale, 3200 ft, 3 Oct 1952, *J.B. Gillett 13986* (Paratype: K! [K000354128]).
Coccinia megarrhiza Type: Kenya. Northern Province [Eastern Province]: Moyale, 3600 ft, 14 Oct 1952, *J.B. Gillett 14036* (Paratypes: B! [B 10 0154927, digital image: B, JPS], BR!, EA!, K! [K000354131], S! [S08-12482]).
Coccinia megarrhiza Type: Kenya. ibid., *J.B. Gillett 14037* (Paratypes: BR! [BR0000008914033], K! [K000354133], K! [K000354134], S! [S08-12481]).
Coccinia megarrhiza Type: Kenya. ibid., *J.B. Gillett 14038* (Paratypes: K! [K000354125], K! [K000354132]).
Coccinia megarrhiza Type: Kenya. ibid., *J.B. Gillett 14039* (Paratypes: K! [K000354126], S! [S08-12480]).

#### Description.

Perennial climber or creeper. Stem up to 6 m, with long, whitish to beigeish patent trichomes, which appear articulate when dried. Petioles 1.5–5.6 cm, indumentum as on stem (Fig. 9). Leaves 3–11 × 4.2–17 cm, reniform to 3- or 5-lobate. Margin dentate (teeth at maturity brownish, when dried blackening), serrate to lobulate. Upper leaf surface glabrous with pale pustules or with short, whitish to beigeish trichomes, lower leaf surface with indumentum as on stem, rarely glabrous. Probracts up to 3 mm long. Tendrils simple. Male flowers clustered. Pedicel < 1.5 cm, indumentum as on stem. Perianth tube with long, beigeish, upright trichomes that appear articulate when dried. Calyx lobes 2.5–4 mm, subulate to lineal, spreading. Corolla 1.2–1.3 cm, yellow to pale orange, lobes 4–6 mm. Filament column greenish, anther head pale greenish, pollen sacs orange-yellow. Female flowers 1(–2) solitary. Hypanthium with long, beigeish, upright trichomes that appear articulate when dried, calyx lobes and corolla like in male flowers. Ovary green with whitish spots. Style columnar, green. Stigma bulging, yellow (Fig. 11c). Fruit ovoid–ellipsoid, up to 6.5 cm long, unripe green with longitudinal white mottling. During ripening mottling partly developing a dark green corona (Fig. 13c). Ripe red (Fig. 12b). Seeds 7 × 4 × 1.5 mm (L/W/H), asymmetrically obovate, face flat.

#### Phenology.

Flowering time: March–May, August–October.

#### Distribution.

Fig. 20. Ethiopia (Oromia, Somali Region), Kenya (Eastern Province, North Eastern Province). Elevation 800–1600[–2000] m. On granite outcrops and red lateritic soils, *Acacia*-*Commiphora* bushland, *Dracaena* vegetation, *Balanites* vegetation, *Euphorbia candelabrum* woodlands, dry *Juniperus* forest, grazing is tolerated.

#### Remarks.

*Coccinia megarrhiza* and *Coccinia abyssinica* form a species complex. Distinction between these two species can be difficult, especially in young plants, when the color of the marginal teeth of the leaf is not well developed. While the peduncle length differs, the earlier appearing solitary flowers can have the same length in both species. The broad leaves with an emarginate, obtuse to cuspidate tip (*Coccinia megarrhiza*) versus rather long leaves with an acute tip (*Coccinia abyssinica*) seems to be the best character. At maturity, the teeth coloration in *Coccinia megarrhiza* is also much more conspicuous than in *Coccinia abyssinica*. A phylogeographic analysis and crossing experiments would shed light on the question, whether these are ecologically differentiated forms or true species. Plants from the mountains near Yebelo with very large leaves are almost glabrous and occur, untypically, in dry *Juniper* “forests”. However, they have the typical cuspidate to obtuse central lobes and bear the colored leaf margin teeth. As larger leaves are also observed in high altitude individuals of *Coccinia microphylla*, these forms might be regarded as mast specimens.

#### Specimens examined.

(Selection; in total: 29) Ethiopia. Oromia: 38 km S of Neghelli [Negele Boran] on Wachelli road, *J.W. Ash 814* (EA (2), K); Arero (Meta-Gafersa), *G. Cufodontis 273* (FT, W); Bombal ca. 40 km on the way to Jijiga from Harar, *T. Ebba 622* (K, WAG [WAG0285707]); c. 20 km NW of Moyale on the road to Mega, just after the turn off to Tuqa (and Sololo in Kenya) (3°39'N, 38°56'E), *I. Friis et al. 8736* (K); c. 36 km from Harar to Jijiga and then c. 20 km to S, *J.J.F.E. de Wilde 4793* (B, K, MO, WAG [WAG0285708], WAG [WAG0285709]). Somali Region: 95 km from Negele of Filtu road, 5°00'N, 40°12'E, *M.G. Gilbert & B.M.G. Jones 110* (K).

### 13. Coccinia microphylla

Taxon classificationPlantaeCucurbitalesCucurbitaceae

Gilg, Bot. Jahrb. Syst. 34: 357. 1904.

Coccinia microphylla Type: Tanzania. [Kilimanjaro]: at base of Pare Mountains, between Kiswani [Kisiwani] and Maji ya Juu [Madji-ja-juu], mix of thornbush and wooded grassland [“gemischte Dornbusch- und Obstgartensteppe”], 700 m, fl, Oct, *A. Engler, Reise nach Ostafrika 1587* (Syntype: B destroyed).
Coccinia microphylla Type: Tanzania. [Kilimanjaro]: between Kihuiro [Kihurio] and Gonja, thornbush steppe, fl, Oct, *A. Engler, Reise nach Ostafrika 948* (Syntype: B destroyed).
Coccinia microphylla Type: Kenya. Coast Province: near Mariakani, NW of Mombasa, male, fl, 15 Oct 1955, *E. Milne-Redhead & P. Taylor 7104* (Neotype, designated here: LISU!, isoneotypes: B!, EA!)
Coccinia buikoensis Zimm., Die Cucurbitaceen 2: 177, 24, 51, 84, 96, 114, fig. 17 I–III, fig. 63 II, fig. 74 VII–XII, fig. 81 XVI, XVII. 1922.
Coccinia microphylla Type: Tanzania. [Tanga]: Lushoto District, [S of Pare Mts], [between Hedaru and Mkomazi], near Buiko, steppe, male and female, fl, fr, Dec 1919, *P.W.A. Zimmermann G6595* (Holotype: B destroyed, lectotype designated by Jeffrey (1967: 68): EA [EA000002132, digital image: JPS]).
Coccinia sp. C in C.Jeffrey, F. T. E. A.: 69. 1967. Kenya, Northern Province: Furroli, lava plateau, semi-desert, *Acacia*-*Commiphora* shrub, on sand, female, fl, fr, 12 Sep 1952, *J.B. Gillett 13820* (B!, EA!, K!, P!, S! [S08-12180]) and *J.B. Gillett 13826* (K!).

#### Description.

Perennial creeper or climber. Stems up to 4 m, glabrous or more or less densely covered with short, white trichomes, when older often densely white pustulate. Petiole 0.45–4 cm, with erect, often thick, when longer sometimes bent trichomes that are sometimes soft spiny (< 1 mm) or only wart-like. Leaves 0.7–7.5 × 1.1–12 cm wide, usually rather small, shallowly to deeply 3- or 5-lobate, sometimes lobulate, rarely reniform. Lobes narrow to broadly triangulate to lanceolate. Upper leaf surface more or less densely white pustulate, pustules sometimes with a short, thick trichome (Fig. 2a). Lower leaf surface glabrous or more or less dense with often stiff, pale yellowish trichomes, sometimes with darker glands between nerves, nerves usually with thick, erect trichomes, sometimes reduced to wart-like appearance. Leaf margin rather remotely denticulate, usually with minute, bent trichomes. Apex acute to obtuse, with final tooth. Probract usually absent, if present then < 1.5 mm. Tendrils simple. Male flowers 1–2 solitary, if fasciculate or in few-flowered racemes, then accompanied by 1 solitary flower. Common peduncle < 5 mm, glabrous or with minute to long, articulate trichomes. Pedicel of solitary flowers 2–7(–25 mm), pedicel of flowers in inflorescences < 5 mm, glabrous or especially at apex with long, yellowish, articulate trichomes. Perianth tube usually with long, yellowish, articulate trichomes, rarely almost glabrous. Calyx lobes 1.5–4 mm, narrowly lanceolate to lineal, when young erect, later spreading to reflexed. Corolla 0.7–1.2 cm long, greenish white to yellowish-orange with darker green veins, lobes 4–7 mm. Filament column and anther head pale greenish yellow. Pollen sacs yellow. Female flowers 1(–2) solitary (Fig. 2a). Pedicels up to 0.7 cm, glabrous or with white trichomes. Ovary glabrous, with some articulate trichomes to densely wooly with long (when dry articulate) trichomes. Style columnar, pale green. Stigmas bulging, greenish yellow. Ripe fruit globose to shortly obovate, 1.8–2.5 × 1.4–2.5 cm, glabrous or with few articulate trichomes, unripe green sometimes with longitudinal, whitish mottling (Fig. 2a), which obtains a dark green corona during ripening, when ripe bright orange-red to red. Seeds 4.5–6 × 2–3 × 1–1.5 mm (L/W/H), asymmetrically obovate to somewhat falcate, face flattened.

#### Phenology.

Flowering time: January, April, May, July, October–December.

#### Distribution.

**Figure 33.**
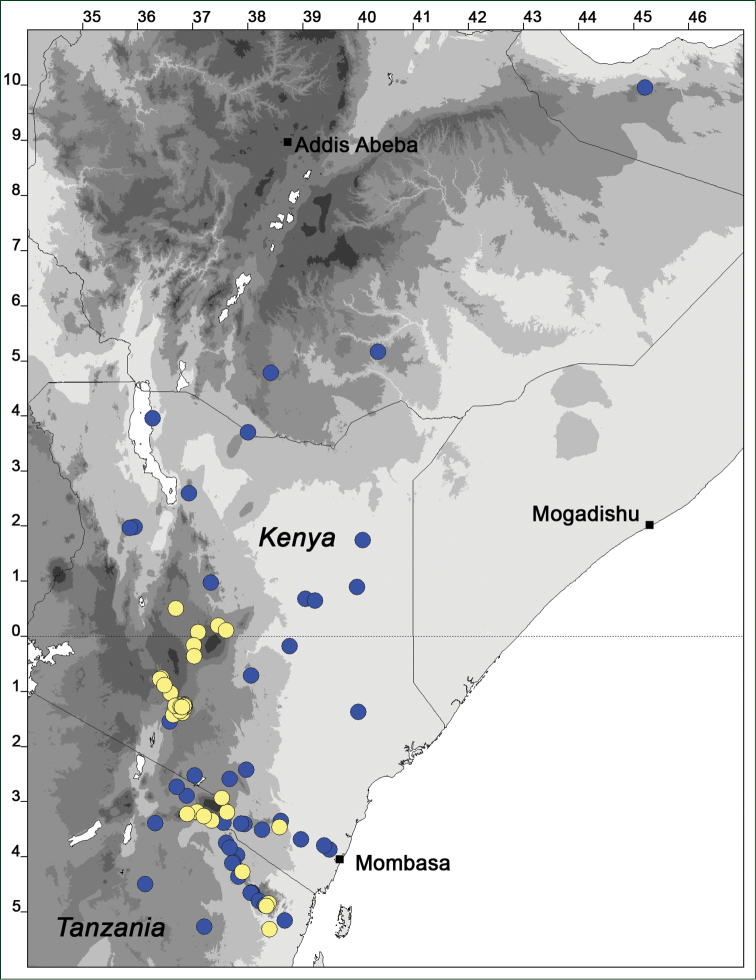
Distribution map of *Coccinia microphylla* (blue dots; based on 49 collections) and *Coccinia trilobata* (pale yellow dots; based on 51 collections).

Fig. 33. NE Tanzania (Arusha, Kilimanjaro, Manyara, Tanga), Kenya (Coast, Eastern, North Eastern, Rift Valley), S Ethiopia (S and C Oromia), Somalia. 70–1300(–1600) m. Savanna, open *Acacia*-*Commiphora* bushland, degraded *Combretum* bushland, open grassland, cultivated land, roadsides. Red sand, dark brownish-black soil. Limestone.

#### Vernacular names.

[Akiek; Ogiek]: notoku (*A.S. Vincent 29*, *A.S. Vincent 221*), Maa [Maasai language]: ndegegeya (*A.S. Vincent 29*), sikuni (*Kiamba et al. KEFRI 112*).

#### Remarks.

Some collections have a mixed (not intermediate) phenotype with *Coccinia trilobata*: the calyx lobes are unusually long (up to 7 mm), which speaks for *Coccinia trilobata*, but the indumentum matches *Coccinia microphylla*. However, these do not occur in a single location, but are found in the Ndoto Mts (*O. Kerfoot 2644*), in Kiboko (*P. Kirika et al. 002/020/2011*), and around Voi (*M. Hucks 579*, *B. Verdcourt 3888*). Whether these are hybrids (F2 or later) or just a variety is not known. These collections also resemble *Coccinia megarrhiza*, which occurs in northern Kenya and Ethiopia, however, the indumentum does not match either.

Despite the epithet, the leaves can become quite large, especially at higher altitudes. Then, collections may resemble *Coccinia trilobata*, which has a denser indumentum. In dry low altitude areas, leaves and flowers emerge quickly, e.g., soon after a rain shower. The leaves are thus not well developed and small. Collections from more arid locations tend to be smaller in many characters, but whether these represent a new species is doubtful. There are only few collections of the proposed species (*Coccinia* sp. *Burger 2947A*, *Coccinia* sp. *Gilbert & Jones 129* (Jeffrey 1995)), but the natural scope of *Coccinia microphylla* is hardly assessed. This will not be resolved without a phylogeographic analysis and more intensive collecting from Ethiopia.

#### Specimens examined.

(Selection, in total: 72) Ethiopia. Oromia: 105 km on road from Negelli [Negele] to Filtu, *J.J.F.E. de Wilde & M.G. Gilbert 346* (K, WAG [WAG0285710], WAG [WAG0285711], UPS). Kenya. Coast Province: near Mariakani, NW of Mombasa, *E. Milne-Redhead & P. Taylor 7105* (B, EA, LISU). Eastern Province: E side of Lake Rudolf, between Koobi Fora and Shin (hill), 3°57’–58'N, 36°12'–20'E, *R.B. Faden & A. Evans 71/301* (EA, K). North Eastern Province: Wajir District, Catholic Girl’s Town 2 km E of Wajir, *J.B. Gillett 21273* (EA, K, WAG [WAG0234120]). Rift Valley Province: Turkana District, *I. Ohta 24* (EA). Somalia. Togdheer: Malol [Mt Malool] near Sheikh, *J.R. Ironside 5/73/31* (K). Tanzania. Arusha: Monduli district, Longido division, SE of Longido, c. 100–300 m from Arusha Municipality, 2°43'14"S, 36°42'02"E, *C.J. Kayombo & K. Kitaba 4242* (MO). Dodoma: Tarangire National Park, Kalima Hill, *S. Chuwa et al. 5329* (NHT). Kilimanjaro: Same district, Mkomazi Game reserve, Ibaya Hill, 3°58'S, 37°48'E, *R. Abdallah & K. Vollesen 95/198* (BR, K, P [P05620649]). Tanga: Korogwe District, 2 km W of Mkomazi, under power line, 4°38'53.7"S, 38°03'26.7"E, *N. Holstein et al. 90* (B, DSM, M).

### 14. Coccinia mildbraedii

Taxon classificationPlantaeCucurbitalesCucurbitaceae

Gilg, Wissenschaft. Ergebn. Deutsch. Zentral-Afrika-Exped. 1907–1908 Herzog Adolf Friedrich zu Mecklenburg, Bot. 2(4): 343. 1914.

Coccinia mildbraedii Type: Rwanda. [Western Province]: Kissenye [Gisenyi]. Bugoy [Bugoyi] forest, mixed bamboo forest, c. 2500 m, fl, fr, 30 Oct 1907, *J. Mildbraed 1425* (Holotype: B, destroyed).
Coccinia mildbraedii Type: Burundi. Muramvya: [Mt] Teza, 3°13'S, 29°33'E, female, fl, fr, *M. Reekmans 7399* (Neotype, designated here: K!; isoneotypes: BR!, EA!, MO!, WAG! [WAG0225430], WAG! [WAG0225433]).
Coccinia ulugurensis Harms in Mildbraed, Notizbl. Bot. Gart. Berlin-Dahlem 11: 1091. 1934.
Coccinia mildbraedii Type: Tanzania. [Morogoro]: Uluguru Mts, northwestern side, c. 1350 m, over shrubs at forest margin, male, fl, 14 Mar 1933, *H.J.E. Schlieben 3643* (Holotype: B! [B 10 0154929, digital image: B, JPS], isotypes: B! [B 10 0154930, digital image: B, JPS], BM! [BM000815208], BR! [BR0000008886163, K neg. 5264, digital image: JPS], BR! [BR0000008887498, digital image: BR, JPS], G! [G00301594], HBG! [HBG506425, digital image: JPS], LISC! [LISC 002496, digital image: IICT, JPS], M! [M0105771, digital image: JPS], MA! [MA386121, digital image: JPS], MO!, P! [P00346275, digital image: JPS, P], S [S-G-1519, digital image: JPS], Z! [Z-000004448, digital image: Z], photo of isotype from BR! [EA, K]).

#### Description.

Perennial climber. Stems up to 20 m, when young sometimes villose with whitish, articulate trichomes, later often subglabrous to glabrous. Petioles 4–8 cm long, glabrous or with pale, articulate trichomes. Leaves 9.5–16.5 × 10–16.5 cm, shallowly to profoundly 3- or 5-lobate. Lobes triangulate, ovate to elliptical. Leaf margin entire and denticulate to serrate. Upper leaf surface glabrous or with hyaline to white pustules. Lower leaf surface glabrous or sometimes villose with whitish, articulate trichomes, sometimes with white pustules on the main veins. Probracts up to 3.5 mm. Tendrils simple or bifid. Male flowers in racemes, rarely accompanied by one solitary flower, or one single flower only. Common peduncle 3–4.5 cm, pedicels up to 7 mm, bracts up to 1mm, caducous. Pedicels of solitary flowers up to 2.5 cm, each glabrous. Perianth tube glabrous, calyx lobes up to 2.5 mm, triangulate to lineal, in buds adpressed to corolla, later spreading. Corolla 1.2–2.9 cm long, orange buff, lobes 0.3–1 cm. Filament column and anther head not seen. Pollen sacs cream yellow. Female flowers solitary, pedicel 3–8 cm, glabrous. Hypanthium glabrous, calyx lobes and corolla like in male flowers. Ovary glabrous, ribbed. Style not seen, stigma bulging, yellow. Fruit up to 20 cm long and 5 cm in diameter, unripe green with white mottling and longitudinal green lines, ripening via yellow, orange into deep red. Seeds 6–7 × 5 × 1.5 mm (L/W/H), symmetrically obovate, face flatly lenticular.

#### Phenology.

Flowering time: January–April, June, August, September, November, December, likely throughout the year.

#### Distribution.

**Figure 34.**
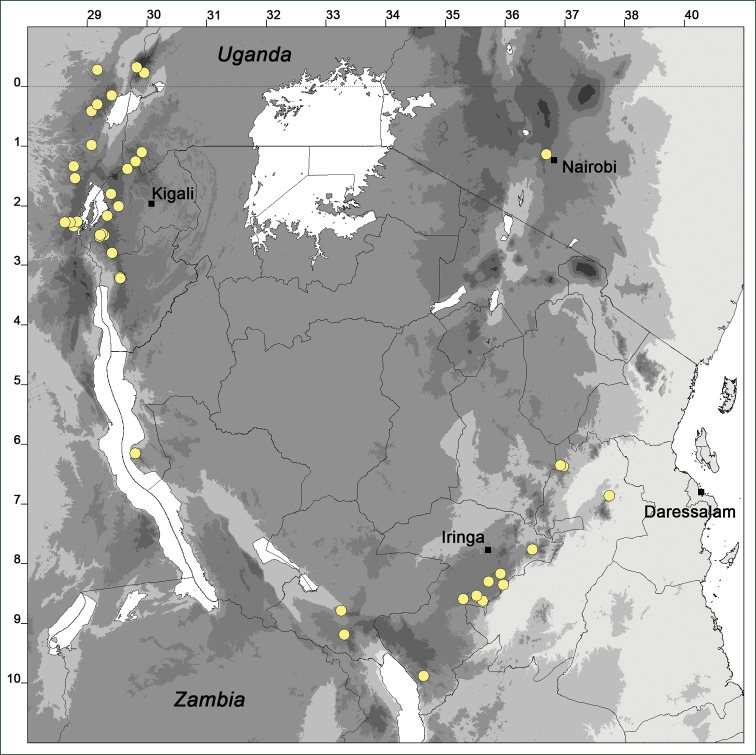
Distribution map of *Coccinia mildbraedii* (based on 51 collections). The individual in Kenya is introduced. For Tanzania the borders of the regions are given.

Fig. 34. NW Burundi, D. R. Congo (North Kivu, South Kivu), Rwanda (Western Province), Tanzania (Iringa: Kipengere Range, Uzungwa Mts; Kigoma: Mahali Mts; Mbeya: Kipengere Range; Morogoro: Uluguru Mts; Ukaguru Mts, Uzungwa Mts), Uganda (Western Region). Elevation 1200–2600 m. Afromontane cloud forests and mist forests, upland rainforests, bamboo forest, in *Macaranga kilimandsharica* shrubs, rarely in *Pennisetum* savannas. On basalt and graphitic schist soils, lateritic clay. Introduced in Kenya.

#### Vernacular names.

Kihunde: mutanga (*Deru 485*), Kindanda: mwore (*Deru 485*), Kinande: mombowa (*P. Gille 218*), Kinyarwanda: umuvunguvungu (*G. Bouxin 820*), umufungofungo (*G. Troupin 11163*), umwonkalere (*Deru 485*), Kisafwa: itangalulu (*C.J. Kayombo 1003*).

#### Remarks.

*Coccinia ulugurensis* cannot be definitely distinguished from *Coccinia mildbraedii*. The leaves are 3-lobate with rather triangulate lobes towards central Tanzania (*Coccinia ulugurensis*), whereas in the western areas the leaves may be deeper lobate with lanceolate lobes (*Coccinia mildbraedii*). Collections of the *Coccinia ulugurensis* form also occur in the Ukaguru Mts (*M. Thulin & B. Mhoro 2933*) from which a close-by located population has been recollected for sequencing (*N. Holstein et al. 76*) because collections from the Uluguru Mts were not available. However, forms similar to *Coccinia ulugurensis* also occur in the Western Rift area. Vice-versa, 5-lobate leaves also occur in central Tanzania. Jeffrey (1967) used to distinguish the species also by the occurrence of “crisped hairs”, but these do not seem to be specific. Such trichomes also occur in other species, such as *Coccinia adoensis*, *Coccinia grandiflora* and *Coccinia mackenii*, and the fine pubescence as described for *Coccinia ulugurensis* regularly occurs in young shoots and often disappears later on. Collections that represent the two species cluster together (Fig. 17) and either both species share major parts of their distribution ranges and only differ morphologically in nuances of lobation depth, or they belong to a single species, of which here the latter case is assumed here.

#### Specimens examined.

(Selection, in total: 76) D.R. Congo. North Kivu: Lubero territory, Bingi, *A. Léonard 5415* (BR, EA, WAG [WAG0225422], WAG [WAG0225423], WAG [WAG0225424]). South Kivu: Kabare territory, Marais Musisi, 28°42'E, 2°16'S, *P. Bamps 2844* (BR, EA, WAG [WAG0225420]). Kenya. Central Province: Limuru, tea estate, introduced from Tanzania, *J.B. Gillett 20185* (EA, MO). Rwanda. Western Province: Shangugu territoire, Mont Bigugu, *A.R. Christiaensen 1616* (EA, WAG [WAG0225419]). Southern Province: Rutovu, km 64 on Astrida [Butare]–Shangugu [Cyangugu] route, *M. Reynders 394* (BR). Tanzania. Iringa: Dabaga Highlands, Ihangana Forest Reserve, near Kibangu, 18 mls [29 km] S of Dabaga, *R. Polhill & S. Paulo 1476* (B, BR, EA, K, P [P05620648], PRE). Kigoma: Mpanda district, Mahali Mts, Sisaga, c. 6°S 30°E, *T.G. Jefford & J.G.B. Newbould 1924* (COI, EA). Mbeya: Mbeya rural district, Umalila Forest Reserve, c. 7 km W of Ruanda II on road to Izumbwe (2 km SSE of main peak of Mbogo Mt.), 9°11'S, 33°18'E, *R.E. Gereau et al. 5060* (K, MO). Morogoro: Kilosa district, Ukaguru Mts, between Mandege and Masenge, 6°22'S, 36°58'E, *M. Thulin & B.E. Mhoro 2792* (DSM, EA, K, MO). Uganda. Western Region: Kigezi district, Virunga chain, northern foot of Mzhavura Mt., Nkanda, *H.U. Stauffer 931* (BR, M, Z).

### 15. Coccinia ogadensis

Taxon classificationPlantaeCucurbitalesCucurbitaceae

Thulin, Kew Bull. 64: 485. 2009.

Coccinia ogadensis Type: Ethiopia. Somali Region: Harerge, 5 km S of Qarsonney, female, fr, 15 May 2006, *M. Thulin et al. 11183* (Holotype: ETH; isotypes: K! [K000543219, digital image: K], UPS!).
Coccinia ogadensis Type: [Ethiopia]. [Somali Region]: Somaliland, Harradigi [Harradigit], Mar 1885, *F.L. James & J.G. Thrupp s.n.* (Paratype: K!).
Coccinia ogadensis Type: [Ethiopia]. [Somali Region]: Somaliland, Harradiqi [Harradigit] or Boobi, Mar or Apr 1885, *F.L. James & J.G. Thrupp s.n.* (Paratype: K!).
Coccinia ogadensis Type: [Ethiopia]. [Somali Region]: Agar Ven [Agar Uen], 6°30'N, 45°20'E, 2500 ft [c. 760 m], red sandy soil, bushland, 25 Oct 1953, *P. Ellis 163* (Paratypes: FT!, K (2)!).
Coccinia ogadensis Type: [Ethiopia]. [Somali Region]: W of Shillavo (Scillave) [Shilabo], 6°25'N, 44°42'E, 1300 ft [c. 400 m], sandy soil, bushland, Nov 1955, *P. Ellis 383* (Paratype: K!).
Coccinia ogadensis Type: [Ethiopia]. [Somali Region]: E of Gorrahei [Korahe], 700 m, 1 Nov 1967, *P.R.O. Bally 12989* (Paratypes: G!, K!).
Coccinia ogadensis Type: [Ethiopia]. [Somali Region]: Scillave [Shilabo]–Wardere road, 6°13'N, 44°45'E, 1130 ft [c. 344 m], red sandy soil, open bushland, male, fl, 2 Apr 1956, *J. Simmons S63* (Paratypes: EA!, K!).
Coccinia ogadensis Type: [Ethiopia]. [Somali Region]: 11 km NE Scillave [Shilabo], 6°10'N, 44°52'E, 1300 ft [c. 400 m], red sandy soil, open bushland, 13 Apr 1956, *J. Simmons S179* (Paratypes: EA!, K!).
Coccinia ogadensis Type: Somalia. [Mudug]: 47 miles [75 km] from Galkayo [Gaalkacyo] on Garoe [Garoowe] road, c. 1000 ft [c. 300 m], red sandy loam and limestone ridges, 15 Oct 1959, *C.F. Hemming 1713* (Paratypes EA!, K!).

#### Description.

Perennial? climber or trailer. Stems up to 2 m or longer. Stems glabrous, except for nodes with short trichomes, sometimes white pustulate. Petioles 4–15 mm long, glabrous or nearly so. Leaves deeply (3- or) 5-pedately lobate. Central lobe 2–8.5 cm long, 1–8 mm wide, lateral lobes shorter. Lobes entire or dentate to lobulate, linear to oblanceolate. Leaf margins often revolute, apex obtuse with final (brownish? colored) tooth to acute. Upper leaf surface glabrous, pale to white pustulate; pustules up to 5 mm in diam. Lower leaf surface glabrous, at base with pale aureolate glands between nerves. Probracts < 1 mm with short, whitish trichomes. Tendrils simple. Male flowers solitary, clustered, or in few-flowered racemes. Common peduncle up to 2 cm, glabrous. Pedicels up to 4–20 mm long, subglabrous to glabrous. Perianth tube glabrous, calyx lobes 1–6 mm long, in buds erect, later reflexed, glabrous or nearly so, lineal to narrowly triangulate. Corolla 1.7–2.5 cm, white with green veins or yellow, lobes 0.7–1.3 cm. Petals inside with multicellular trichomes, outside with short, oligocellular trichomes. Color of filament column, anther head, and pollen sacs not seen. Female flowers not seen, but very likely solitary, pedicels, hypanthium/perianth tube, calyx lobes and petals not largely differing from male flowers. Fruits spindle-shaped to shortly cylindrical, 4.5–5.5 cm long, c. 1.5 cm in diameter, sometimes with short apical tip (“beaked”). Unripe green with elongate with spots, turning red with whitish, elongate spots. Seeds 4–5.5 × 2–2.5 × 1–1.5 mm (L/W/H), slightly asymmetrically obovate, face flatly lenticular.

#### Phenology.

Flowering time: Imperfectly known, flowering in April and in October and November during rainy seasons.

#### Distribution.

**Figure 35.**
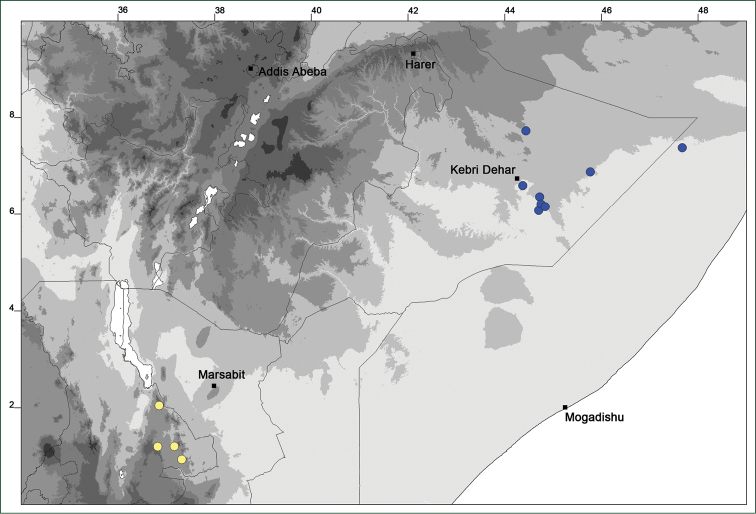
Distribution map of *Coccinia ogadensis* (blue dots; based on 8 collections) and *Coccinia samburuensis* (pale yellow dots; based on 4 collections). For Ethiopia the borders of the regions and for Kenya the borders of the provinces are displayed.

Fig. 35. Eastern Ethiopia (C and E Somali Region), Central Somalia (Mugud). Elevation 300 to 800 m. Red sand and sandy loam, limestone soils. Open *Acacia*-*Commiphora* bushland, *Cordeauxia edulis* bushland, semi-desert.

#### Use.

Fruits are reported to be edible, juicy, and thirst-quenching (*P.R.O. Bally 12989*).

#### Vernacular names.

Somali: dudu (*P.R.O. Bally 12989*), ilgeel (Thulin 2009), Somali?: lehailu (*J. Simmons S179*), Somali?: salo dudub (*J. Simmons S63*).

#### Remarks.

This species is similar to collections of *Coccinia grandis* with deeply lobate leaves (described as *Coccinia palmatisecta*). However, the lobules in *Coccinia grandis* are much more distinct when the lobulation is that deep. Apart from this, fruit and seed shape of *Coccinia ogadensis* resemble that of *Coccinia adoensis*.

Ellis notes on the collections no. *163* and *383* a smell of rotten meat. However, it is unclear, whether this is coming from the flowers or from crushed leaves. Several cucurbit species have a putrid smell when crushed, such as *Kedrostis foetidissima* or *Momordica foetida*, but this has never been reported for a *Coccinia* species.

#### Specimens examined.

(in total: 10). Ethiopia. Somali Region: Ogaden, *J. Simmons 64* (EA).

### 16. Coccinia pwaniensis

Taxon classificationPlantaeCucurbitalesCucurbitaceae

Holstein, Kew Bull. 65(3): 435. 2010 [publ. 1 Jan 2011].

Coccinia pwaniensis Type: Kenya. [Coast Province]: Kwale District, Buda Mafisini forest, 8 miles [12.9 km] WSW of Gazi, 80 m, male, fl, 22 Aug 1953, *R.B. Drummond & J.H. Hemsley 3953* (Holotype: K! [3 sheets, K000309479, the other two sheets without barcode], isotype: EA!). Type: Kenya. Coast Province: Kilifi District, Mangea Hill, 39°42'E, 03°16'S, 450 m, dry bushland with *Cynometra* sp., *Brachylaena* sp., *Manilkara* sp., *Brachystegia*sp., *Julbernardia* sp., *Diospyros* sp., *Xylopia* sp., *Inhambanella* sp., 28 Dec 1988, *W.R.Q. Luke 1601* (Paratype: EA!).
Coccinia pwaniensis Type: Kenya. [Coast Province]: Kwale District, Cha Simba forest, 300 m, fr, 1 Feb 1953, *R.B. Drummond & J.H. Hemsley 1078* (Paratype: K!).
Coccinia pwaniensis Type: Kenya. [Coast Province]: Kwale District, Shimba Hills, Giriama Point area, 1250 ft [c. 381 m], forest edge, 17 Mar 1968, *F.C. Magogo & P. Glover 315* (Paratypes: EA!, K!).
Coccinia pwaniensis Type: Kenya. [Coast Province]: Kwale District, Shimba Hills, Pengo Hill area, 1500 ft [c. 457 m], forest, 27 Mar 1968, *F.C. Magogo & P. Glover 493* (Paratypes: EA!, K!).
Coccinia pwaniensis Type: Kenya. Shimba Hills, SE-part of Longomagandi Forest, 350 m, lowland rainforest, 13 Nov 1988, *R. Schmidt 1203* (Paratype: EA!).
Coccinia pwaniensis Type: Kenya. Kwale District, no detailed location given, 15 Jun 1957, *Saunders 11241* (Paratype: EA!).
Coccinia pwaniensis Type: Tanzania. Pwani: Bagamoyo District, Zaraninge Forest in Kiono Plateau, 38°36'E, 6°09'S, 1000 ft [c. 305 m], dry evergreen coastal forest, on sand, 14 Mar 1990, *Frontier-Tanzania Coastal Forest Research Programme 1041* (Paratype: K!).
Coccinia pwaniensis Type: Tanzania. [Pwani]: Kirasawe District: Pugu Hills Forest Reserve on Dar es Salaam–Kisarawe road. Roadside in forest, 100–270 m, 12 May 1970, *K.H. Macauley CVL 102* (Paratypes: DSM!, EA!).
Coccinia pwaniensis Type: Tanzania. [Pwani]: Pugu Hills, 19 Mar 1939, *J.H. Vaughan 2774* (Paratype: EA!).
Coccinia pwaniensis Type: Tanzania. [Pwani]: Pugu Hills Forest Reserve, road W from road-tunnel, 100 m, in bushes by car-track through forest, 23 Jul 1972, *R.C. Wingfield 2056* (Paratypes: DSM!, EA!).
Coccinia sp. B in C.Jeffrey, F. T. E. A.: 69. 1967. *R.B. Drummond & J.H. Hemsley 1078* (K!); *R.B. Drummond & J.H. Hemsley 3953* (K!, EA!); *Saunders 11241* (EA!); *J.H. Vaughan 2774* (EA!).

#### Description.

Perennial climber or creeper. Stems up to 3 m long, glabrous. Petiole 0.6–4.1 cm, adaxial side glabrous or with short, stiff trichomes, abaxial side with stiff, patent trichomes that can be quite reduced, then appearing wart-like or subglabrous. Leaves 2–10.4 × 2.7–11.4 cm, shallowly to profoundly 3-(or 5-)lobate, lobes broadly triangulate to elliptic, margin minutely dentate, tips acute. Upper leaf surface minutely hyaline pustulate, nerves sometimes with tiny trichomes, lower leaf surface glabrous, rarely with blackish glands at base, nerves towards the base with stiff, patent trichomes that can be quite reduced, then appearing wart-like or subglabrous. Probracts 2–3 mm long. Tendrils simple. Male flowers in racemes, sometimes accompanied by 1–2 solitary flowers. Peduncle 3.2–7.7 cm, glabrous, pedicels of flowers in racemes 0.2–1 cm, bracts 1–1.5 mm, pedicels of solitary flowers up to 3.8 cm. Perianth tube glabrous, calyx lobes 2.5–3.5 mm long, subulate and spreading, corolla pale yellow to pale orange-yellow, 1.7–2.6 cm, lobes 1–2 cm. Color of filament stalk, anther head, and pollen sacs not seen. Female flower not seen, perianth likely like in male flowers. Style and stigmas not seen. Fruit solitary, petioles at maturity 20–33 mm long, fruit shape oblong-fusiform, 6.2–8 × 1.8–2.3 cm, rarely (?) with an up to 5.5 cm long sterile apical tip, immature green with pale longitudinal mottling, at maturity becoming orange-red to scarlet-red with pale mottling. Seeds 6.5–7 × 4–4.5 × c. 1.5 mm (L/W/H), more or less symmetrically obovate, face lenticular.

#### Phenology.

Flowering time: January–March, June–August.

#### Distribution.

Fig. 23. Kenya (Coast Province), Tanzania (Pwani, but likely also in Dar es Salaam region and Tanga). Elevation 80–460 m. On sandy soil. Open and disturbed places of East African coastal forests and woodlands (*Brachystegia* sp., *Julbernardia* sp., *Diospyros* sp.).

#### Vernacular names.

Kidigo: mnokonyoka (*F.C. Magogo & P. Glover 493*), mtambaa (*F.C. Magogo & P. Glover 315*), Kijibana: muri ya nyoka (*L.J. Lap 258*).

#### Remarks.

Morphologically this species (the only one missing DNA sequences) is close to *Coccinia senensis*. The indumentum is reduced in prominence and in extent to the petiole and leaves in *Coccinia pwaniensis*, and the leaves are rather 3-lobate and long petiolate, in contrast to often 5-lobate and shortly petiolate leaves in *Coccinia senensis*. The racemes in *Coccinia pwaniensis* have considerably more flowers than in *Coccinia senensis*. However, both species share the subulate calyx lobes, and fruit and seed shape suggest that both species are closely related with *Coccinia adoensis*. As *Coccinia pwaniensis* and *Coccinia senensis* do not co-occur, they might be sister species from allopatric speciation, with *Coccinia pwaniensis* occurring in a refugial distribution in the northern coastal forests of East Africa.

#### Specimens examined.

(in total: 13) Kenya. Coast Province: Kilifi district, Kaya Jibana, entering southern forest patch of Kaya Jibana following the path from shop/hoteli at Mwarakaya–Ribe road, 3°50'0"S, 39°40'30"E, *L.J. Lap 258* (WAG [WAG0195516], WAG [WAG0195517]); Kwale district, Shimba Hills, Longomagandi Forest, *R. Schmidt 527* (EA, UBT).

### 17. Coccinia quinqueloba

Taxon classificationPlantaeCucurbitalesCucurbitaceae

(Thunb.) Cogn. in A.DC. & C.DC., Monogr. Phan. 3: 533. 1881.

Bryonia quinqueloba Thunb., Prodr. Pl. Cap. 1: 13. 1794. *Cephalandra quinqueloba* (Thunb.) Schrad. ex Eckl. & Zeyh., Enum. pl. afric. austral. 2: 280. 1836. *Momordica quinqueloba* (Thunb.) E.Mey. ex Drège, Zwei pflanzengeogr. Dokum.: 126, 132, 133, 137, 202. 1843.
Coccinia quinqueloba Type: [South Africa]. [Eastern Cape]: sylva Krakakamma, male, fl, Dec, *C.P. Thunberg 22836* (Lectotype, designated by Meeuse (1962: 99) and here: UPS-THUNB! [K neg. 2978]).
Coccinia quinqueloba Type: ibid., male, fl, Dec, *C.P. Thunberg 22837* (Syntype: UPS-THUNB! [K neg. 2977]).
Coccinia quinqueloba Type: Cap. b. spei [Cape of Good Hope colony], *C.P. Thunberg s.n.* (Syntype: S! [S08-12379]).

#### Description.

Perennial creeper or climber. Stems up to 9 m, glabrous (rarely with remote trichomes). Leaves usually subsessile, petiole 3–8(–17) mm, glabrous (rarely with remote trichomes). Leaves, 3–9.5 × 4–10 cm, 3- or 5-lobate, auriculate. Lobes oblong, elliptical to obovate. Leaf margin remotely dentate, apices towards lobe often serrate. Lobe apices obtuse with a point. Upper leaf surface pale pustulate, lower leaf surface glabrous, rarely a few blackish glands near base, nerves rarely white-speckled. Probracts < 1 mm or missing. Tendrils simple. Male flowers solitary or in racemes. Common peduncle 0.5–2 cm, petiole in racemous flowers up to 1.8 cm, bracts > 1 mm or missing, solitary flowers with petiole 1.8–4 cm, all glabrous. Perianth tube glabrous, calyx lobes 1.5–3 mm, narrow triangulate, erect to spreading. Corolla 1.2–2.2 cm long, pale yellow, corolla lobes 0.8–1.2 cm. Color of filament stalk, anther head, and pollen sacs not seen. Female flowers one solitary. Petiole 1–2.5 cm, glabrous. Hypanthium glabrous, calyx lobes and corolla like in male flowers. Style and stigmas not seen. Fruits 3.5–9 × 3–4 cm, elliptical to oblong, sometimes short elongated tip, unripe green with longitudinal, white mottling, ripe (orange-)red. Seeds 6–7.5 × 3–3.5 × 1–1.2 mm (L/W/H), slightly asymmetrically obovate, face (flatly) lenticular.

#### Phenology.

Flowering time: January, February, April, July, September, November, December.

#### Distribution.

Fig. 30. Southern and western Eastern Cape, South Africa. Elevation sea level to 1000 m. Sandy soils, also on dolomite soil. Coastal bushland, forest, dry bush, on bushes along rivers, along roadsides.

#### Remarks.

See also under *Coccinia mackenii*.

#### Taxonomic remarks.

*Cephalandra quinqueloba* is the type species of the genus *Cephalandra*. Meeuse (1962) designated the lectotype of *Bryonia quinqueloba* to UPS but did not choose a specimen, which is done here.

#### Specimens examined.

(Selection, in total: 77) South Africa. Eastern Cape: East London, Dec 1916, *H.G. Breyer s.n. TRV23225* (PRE); Amatle Mts, Hogsback Pass, 32°36'50"S, 26°55'25"E, *P.B. Phillipson 1079* (MO, PRE); Glen Avon, Feb 1923, *Mrs. J.E. Brown s.n.* (PRE [“PRE43005”], Z); Grahamstown, Old Quarry, *R.D.A. Bayliss 8470* (G (2), M, MO, Z); 28 mls [45 km] from Grahamstown on Port Elizabeth road, *R. Story 2346* (B [B 10 0019800], L, M (2), MO, PRE, S [S08-12378]); near Port Alfred, *J.L. Sidey 1095* (PRE, S [S08-12464]).

### 18. Coccinia racemiflora

Taxon classificationPlantaeCucurbitalesCucurbitaceae

Keraudren, Adansonia, ser. 2, 8(1): 41. 1968.

Coccinia racemiflora Type: Gabon. [Moyen-Ogooué]: Abanga, C. E. F. A. [Compagnie d’Exploitations Forestière Africaine] lot, male, fl, Jun 1963, *N. Hallé 2425* (Holotype: P! [P00346267, digital image: JPS], P! [P00748828, digital image: JPS, P]).
Coccinia racemiflora Type: Gabon. Ibid., female, fl, *N. Hallé 2305* (Paratype: P! [P00748829, digital image: JPS, P], P! [P00748830, digital image: JPS]).

#### Description.

Perennial climber or prostate creeper. Stems up to 5 m, glabrous. Petioles 0.5–2.5 cm, on adaxial side often with line of thin smutty-beige trichomes or glabrous, abaxial side glabrous. Leaves 6.5–11 × 5–9.5 cm, hastate to 3-lobate with central lobe dominating, auriculate (auricles may reach the stem). Lobes triangulate. Leaf margin entire to somewhat angulate, remotely dentate. Teeth darkening when dried. Upper leaf surface with waxy cover glabrous with few-celled clear pustules. Lower leaf surface glabrous with dispersed blackish glands. Probracts up to 2 mm. Tendrils bifid. Male flowers ebracteate in lax, glabrous racemes. Common peduncle up to 2.5 cm, pedicels 0.3–1 cm. Perianth tube glabrous, calyx lobes 0.5 mm, shortly lineal, spreading. Corolla c. 1.2 cm, yellowish to orange, lobes 2–4 mm. Color of filament column, anther head, and pollen sacs not seen. Female flowers ebracteate in lax, glabrous racemes, like in males. Hypanthium glabrous, calyx lobes, and corolla like in males. Ovary glabrous. Style and stigmas not seen. Unripe fruits glabrous, glaucous, globose. Ripe fruits unknown, size c. 1.5 cm in diam.? Seeds 5 × 3 × 1.5 mm (L/W/H), rather symmetrically obovate, flatly lenticular.

#### Phenology.

Flowering time: January–March, imperfectly known.

#### Distribution.

**Figure 36.**
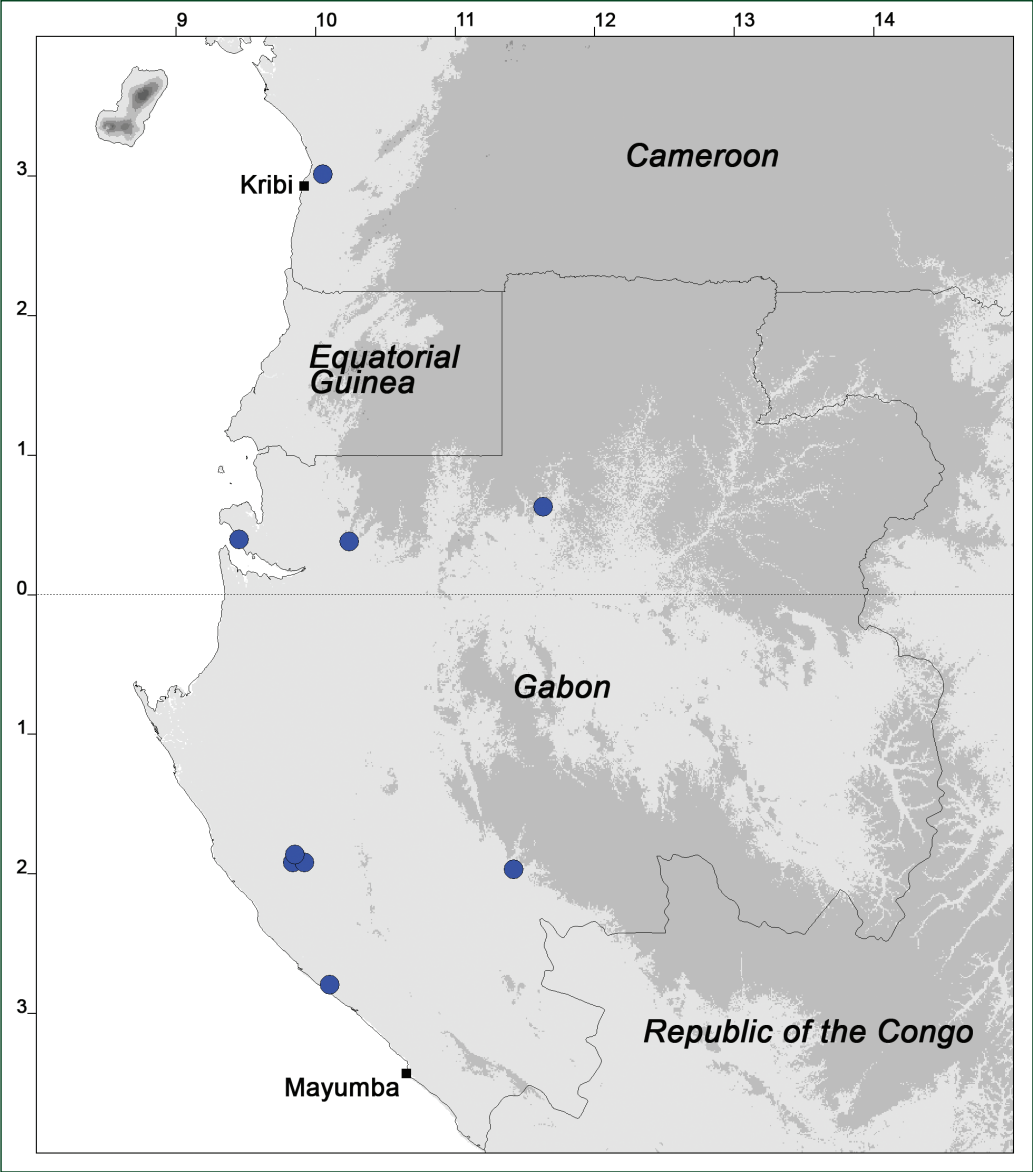
Distribution map of *Coccinia racemiflora* (blue dots; based on 8 collections, including 1 supposed hybrid).

Fig. 36. Gabon, S Cameroon. Tropical lowland rainforest.

#### Taxonomic remarks.

One *Hallé 2425* specimen bears a type label but it is not clear whether it was attached by Kéraudren herself. Since both specimens appear to be part of the same individual, both are best to be treated to be holotypes mounted on two sheets.

#### Remarks.

A collection from the Gamba area in S Gabon (*M.A. van Bergen 490* (WAG [WAG0151338])) is morphologically close to *Coccinia racemiflora* but shares the calyx lobes with *Coccinia barteri* and thus may represent a hybrid. Using plastid markers, this collection (*Coccinia barteri* 6) clusters within *Coccinia barteri*, while in the nuclear *LEAFY*-like tree, it clusters with one representative of *Coccinia racemiflora*, but not with both (Figs 17, 18; Holstein and Renner 2011b). Another collection (*F.J. Breteler et al. 8835* (MO, WAG) from Saint Germain area, C Gabon, has calyx lobes typical for *Coccinia racemiflora*, but a more condensed raceme as in the *M.A. van Bergen 490* collection. The hypanthia of the female flowers are urceolate, whereas in male flowers they are cup-shaped. Urceolate hypanthia are also found in some *Coccinia barteri* forms, and a phylogenetic significance, e.g., by introgressive hybridization, cannot be ruled out.

#### Specimens examined.

(Selection; in total: 10) Cameroon. South Region: 3 km N of km 20 Kribi-Lolodorf, high forest exploitation, 3°01'N, 10°03'E, *J.J. Bos 6590* (WAG [WAG0225514], WAG [WAG0225515]). Gabon. Estuaire: 12 km SW of Kinguélé Falls, *N. Hallé & J.F. Villiers 5357* (K, P [P05620813]). Ngounié: 35 km on road from Lebamba to Yéno, 1°58'S, 11°25'E, *J.J.F.E. de Wilde & M. Sosef 10456* (WAG [WAG0044628]). Ogooué-Maritime: Rabi North, 1°51.6'S, 9°51'E, *I. van Nek 536* (WAG [WAG0044627]).

### 19. Coccinia rehmannii

Taxon classificationPlantaeCucurbitalesCucurbitaceae

Cogn. in Schinz, Bull. Herb. Boiss. 3: 418. 1895.

Coccinia rehmannii Type: South Africa. Transvaal: boshveld ad Klippan [according to Meuse (1962) in Limpopo: Greater Sekhukhune District Municipality, Doornpoort; 24°37'S, 29°26'E], 1875–1880. *A. Rehmann 5156* (Syntype: Z! [Z-000004445, digital image: Z]; syntype: BR! [BR0000005111602, digital image: BR, JPS]).
Coccinia rehmannii Type: South Africa. Ibid., *A. Rehmann 5157* (Syntype: Z! [Z-000060807, digital image: Z]).
Coccinia rehmannii Type: South Africa. Ibid., *A. Rehmann 5168* p.p. (Lectotype, designated by Meeuse (1962: 102): Z! [Z-000060808, digital image: Z]; isolectotype: BR! [BR0000005111930, digital image: BR, JPS], K! [K000313196, digital image: JPS, K]).
Coccinia rehmannii Type: South Africa. At Eland river, *A. Rehmann 4944* [*sic*, must be *A. Rehmann 4954*, see Taxonomic remarks] (Syntype: Z! [Z-000060806, digital image: Z]).
Coccinia rehmannii Cogn. var. littoralis A.Meeuse, Bothalia 8: 104. 1962. pro parte ex *R. de Carvalho s.n.* (Paratypes: COI (2)!).
Coccinia rehmannii Type: South Africa. [Eastern Cape]: [Amatole District Municipality], Komgha, Kei Mouth, *H.G. Flanagan 457* (Holotype: PRE [PRE0190559-0, digital image: JPS], isotypes: BOL?, NBG?).
Coccinia rehmannii Type: South Africa. [Eastern Cape]: Cape Morgan, *H.G. Flanagan 457* (Paratype: GRA [GRA0002852-0, digital image: JPS], BOL?, NBG?).
Coccinia rehmannii Type: South Africa. [Eastern Cape]: East London, Nahoon, *M.W. Nanni 151* (Paratype: PRE!).
Coccinia rehmannii Type: South Africa. [Eastern Cape]: Coffee Bay, *W. Tyson 24* (Paratypes: B!, COI!, GRA, MO!, NY!, PRE!, S! [S08-12380]).
Coccinia rehmannii Type: South Africa. [KwaZulu-Natal]: 10 mls NW of Mtubatuba, *L.E.W. Codd 9620* (Paratypes: COI!, M! [M-0198513], PRE).
Coccinia rehmannii Type: South Africa. [Kwa-Zulu Natal]: Umhlanga Rocks, *A. Dohse & B. de Winter 223* (Paratypes: NH, PRE!).
Coccinia rehmannii Type: South Africa. [Kwa-Zulu Natal]: Manaba Store, *J. Gerstner 3407* (Paratype: NH).
Coccinia rehmannii Type: South Africa. [Kwa-Zulu Natal]: Dhlebe, *J. Gerstner 4261* (Paratypes: NH, PRE).
Coccinia rehmannii Type: South Africa. [Kwa-Zulu Natal]: near Durban, *T.J. Jenkins TRV7092* (Paratype: PRE).
Coccinia rehmannii Type: South Africa. [Kwa-Zulu Natal]: Mtunzini, *S.M. Johnson 612* (Paratype: NBG).
Coccinia rehmannii Type: South Africa. [Kwa-Zulu Natal]: Stella Bush, *W.E. Marriott herb. no. 24341* (Paratype: ?).
Coccinia rehmannii Type: South Africa. [Kwa-Zulu Natal]: ibid., *W.E. Marriott herb. no. 27143* (Paratype: NH).
Coccinia rehmannii Type: South Africa. [Kwa-Zulu Natal]: Shelly Beach, *A.O.D. Mogg 11941* (Paratype: ?).
Coccinia rehmannii Type: South Africa. [Kwa-Zulu Natal]: ibid., *A.O.D. Mogg 12070* (Paratypes: M! [M-0198511], M! [M-0198512], PRE!).
Coccinia rehmannii Type: South Africa. [Kwa-Zulu Natal]: without detailed location, *T.B. Oatley C 15* (Paratype: PRE).
Coccinia rehmannii Type: South Africa. [Kwa-Zulu Natal]: Berea, *Small herb. no. 34714* (Paratype: NH).
Coccinia rehmannii Type: South Africa. [Kwa-Zulu Natal]: Ubombo coastal veld, *P.A. Tosh 28* (Paratype: NU).
Coccinia rehmannii Type: South Africa. [Kwa-Zulu Natal]: Ndumu Game Reserve, *Ward 3169* (Paratype: ?).
Coccinia rehmannii Type: South Africa. [Kwa-Zulu Natal]: ibid., *Ward 3170* (Paratype: ?).
Coccinia rehmannii Type: South Africa. [Kwa-Zulu Natal]: Umvoti, Thorns near Greytown, *J.M. Wood 5318* (Paratype: NH).
Coccinia rehmannii Type: South Africa. [Kwa-Zulu Natal]: Durban, Berea, *J.M. Wood 6350* (Paratypes: BOL, L!, NBG, NH, PRE).
Coccinia rehmannii Type: South Africa. [Kwa-Zulu Natal]: Doonside, *J. Wylie herb. no. 23299* (Paratype: NH).
Coccinia rehmannii Type: Mozambique. Maputo: Lourenço Marques [Maputo], *J.M. Borle 253* (Paratypes: M! [M-0198510], MO!, P! [P05620807, digital image: P]).
Coccinia rehmannii Type: Mozambique. Maputo: ibid., *J.M. Borle 427* (Paratype: ?).
Coccinia rehmannii Type: Mozambique. Maputo: ibid., *J.M. Borle 442* (Paratype: PRE!).
Coccinia rehmannii Type: Mozambique. Maputo: Inhaca Island, *H.G. Breyer TRV20506* (Paratype: PRE).
Coccinia rehmannii Type: Mozambique. Maputo: Inhachingo, *A.W. Exell et al. 630* (Paratype: SRGH).
Coccinia rehmannii Type: Mozambique. Maputo: Massinga, *A.W. Exell et al. 645* (Paratype: SRGH).
Coccinia rehmannii Type: Mozambique. Maputo: Lourenço Marques [Maputo], *A.J.W. Hornby 4599* (Paratype: PRE!).
Coccinia rehmannii Type: Mozambique. Maputo: Delagoa Bay [Maputo Bay], *H.A. Junod 20* (Paratypes: BR!, G (2)!, Z! [Z-000073406, digital image: Z]).
Coccinia rehmannii Type: Mozambique. Maputo: Inhaca Island, 6 Jul 1958, *A.O.D. Mogg s.n.* (Paratype: PRE!).
Coccinia rehmannii Type: Mozambique. Maputo: ibid., 14 Dec 1955, *A.R.A. Noel s.n.* (Paratype: PRE!).
Coccinia rehmannii Type: Mozambique. Maputo: Lourenço Marques [Maputo], *R. Schlechter 11555* (Paratypes: BOL, COI!, G (3)!, GRA, HBG! [HBG518897], PR! [PR 801378], WAG! [WAG0234182], Z! [Z-000073405, digital image: Z]).
Coccinia rehmannii Type: Mozambique. Maputo: Katembe [Catembe], *R. Schlechter 11614* (Paratypes: G (2)!, GRA, PRE!, Z! [Z-000073407, digital image: Z]).
Coccinia ovifera Dinter & Gilg in Dinter, Veg. Veldkost Südw.-Afrik.: 16. 1912.
Coccinia rehmannii Type: [Namibia]. Karas: Sandverhaar, *M.K. Dinter 1214* (Syntype: ?).
Coccinia rehmannii Type: [Namibia]. Otjozondjupa: Otjiwarongo, female, fl, fr, Jan 1912, *M.K. Dinter s.n.* (Syntype: SAM [SAM0072115-0, digital image: JPS]).
Coccinia rehmannii Type: [Namibia]. Waldau, female, fr, 3 Feb 1917, *M.K. Dinter 432* (Lectotype, designated here: SAM [SAM0066515-0, digital image: JPS]).
Coccinia rehmannii Further possible syntypes (cited in Dinter 1919/20) if collected before end of 1912: Oshikoto: Gaub, *M.K. Dinter 2412* (?); Tsumeb, *M.K. Dinter s.n.* (?). Unknown: Hereroland, Palmenwald, *M.K. Dinter s.n.* (?); Hereroland, Wilhelmsberg, *M.K. Dinter s.n.* (?).

#### Description.

Perennial climber or creeper. Stems up to 4 m, glabrous or with broad-based trichomes, when old often densely white pustulate (esp. in drier areas). Petiole 0.2–4.2 cm, glabrous or with erect, broad-based or often up to 1.5 mm long, articulate trichomes or only wart-like, when old sometimes dense white pustulate (esp. in drier areas). Leaves 0.9–9.7 × 1.4–16.6 cm, shallowly to deeply 3- or 5-lobate, auriculate, sometimes lobulate, rarely cordate. Lobes and lobules usually extending, rarely pointing towards tip, narrowly to broadly triangulate to lanceolate. Leaf margin rather remotely denticulate. Apex acute to obtuse, apiculate. Upper leaf surface more or less densely white pustulate, pustules sometimes with a thick, small trichome, on nerves often with thick, small trichomes. Lower leaf surface glabrous, sometimes with small, blackish glands between nerves, nerves usually with erect trichomes, sometimes wart-like. Probract usually absent, if present then up to 3.5 mm. Tendrils simple. Male flowers 1–3 solitary, if fasciculate or in few-flowered racemes then accompanied by 1–2 flowers. Common peduncle 0.7–4.5(–8.5) cm, glabrous or with long, articulate trichomes. Pedicel of flowers in inflorescences 0.6–2.8 cm, bracts up to 2.5 mm or missing. Pedicel of solitary flowers (0.2–)0.5–5(–9) cm, glabrous or especially at apex with long, articulate trichomes. Perianth tube usually with long (> 0.5 mm) trichomes, rarely almost glabrous. Calyx lobes 0.2–7 mm, narrowly lanceolate or lineal, when young erect, later also spreading to reflexed. Corolla 0.8–2.5 cm long, buff to more or less pale yellow, sometimes with green venation. Lobes 0.3–1.1 cm. Filament column pale buff, anthers buff, pollen sacs yellow (Fig. 10a). Female flower solitary. Pedicel 0.4–1.5 cm, glabrous or with long, articulate trichomes. Hypanthium usually with long (> 0.5 mm) trichomes, rarely almost glabrous, calyx lobes and corolla like in male flowers. Ovary rarely glabrous, often more or less densely covered with articulate trichomes. Style columnar, green. Stigmas 2-lobed, yellow (Fig. 11b). Immature fruit, rarely also at maturity, with whitish, longitudinal mottling that develops a dark green corona during ripening. Ripe fruit globose to elliptical 1–6.2 × 1–2.8 cm, glabrous or with few articulate trichomes, bright orange to red. Seeds 4.5–7 × 2–3.5 × 1–1.2 mm (L/W/H), asymmetrically obovate to somewhat falcate, face flat.

#### Phenology.

Flowering time: January–April, June, October–December.

#### Distribution.

**Figure 37.**
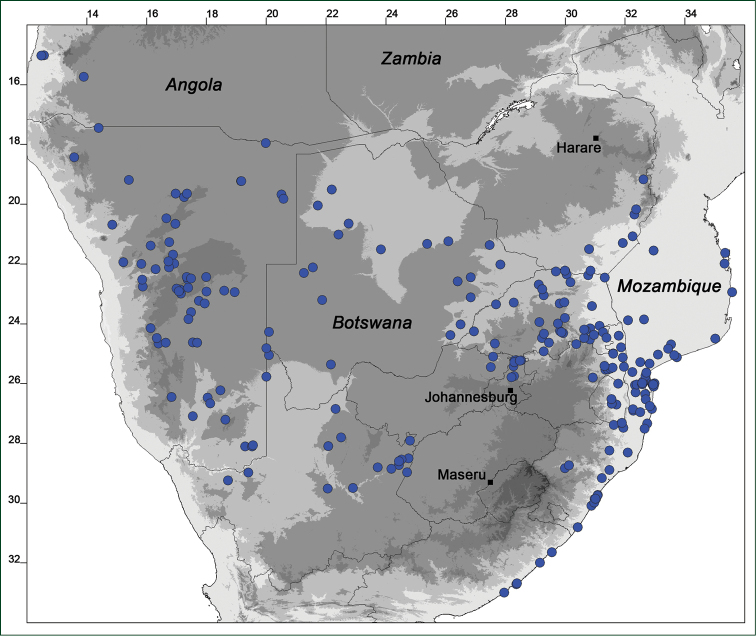
Distribution map of *Coccinia rehmannii* (blue dots; based on 251 collections). For South Africa the borders of the provinces are given.

Fig. 37. South Africa (except Western Cape and SW Eastern Cape), Namibia (except hyperarid regions), Swaziland, southern Mozambique (Gaza, Inhambane, Maputo), Botswana, Zimbabwe (Manicaland, Masvingo, Matabeleland South), southern Angola (Namibe, Huila, Cunene, Cuandocubango). Elevation sea level to 1850 m. Limestone, dolomitic, quartzitic, granitic, and ultrabasic soils. Possibly some tolerance to Ni and Cu. On loam, clay, white and red sand, sandstone, and gravel, but prefers sandy (well drained) soils (Meeuse 1962). Full sun to shade. Coastal dunes, riverbanks, *Acacia sclerocarya*–*Acacia caffra* woodland, *Combretum apiculatum* bushland, *Grewia flava* bushland, mopane, Kalahari thornveld, grassland, semi-desert, dunes. Grazing is tolerated. Light frost seems to be tolerated (Meeuse 1962).

#### Use.

Tuber edible after baking (Dinter 1912, *M.E. Keith 50*, *B. de Winter & O.A. Leistner 5598*). Leaves used as spinach by Shangane people in Gaza province of Mozambique (*K.L. Tinley 3000*). Fruits edible (*R.H.W. Seydel 938*, *R. Story 5029*, *B. de Winter & O.A. Leistner 5598*).

#### Vernacular names.

Muchope [Xichope?]: fuculumué (*L.A. Grandvaux Barbosa & F. de Lemos 8502*), Otjiherero: otjimaga (*M.K. Dinter s.n. Jan 1912*), Ronga dialect [of Xitsonga language]: inyamgwazi (*A.O.D. Mogg 31308*), shiracarana (*L. Macuácua 73* and *75*), Tsonga [Xitsonga]: inyamwazi (*A.O.D. Mogg 31538*), nyampape (*C. Liengme 491*), Zulu [isiZulu]: uselwa-iwenyoka (Sewram et al. 2006).

#### Remarks.

The (sub-)glabrous “*littoralis*” form can be easily confused with the polymorphic *Coccinia adoensis* (Hochst. ex A.Rich.) Cogn., which differs in shorter calyx lobes and lenticular seeds, and with *Coccinia senensis*, which also has lenticular seeds and usually long-peduncled male racemes.

Meeuse’s variety *littoralis* is hard to define as the paratypes are variable, and characters for delimitation are unclear. For example, although the variety should lack white speckles on the stems, there are some individuals with white speckles along with long pedicels or conspicuous racemes in KwaZulu-Natal and southern Mozambique as in the variety *littoralis*. The holotype of var. littoralis is, in the present author’s opinion, rather intermediate between the holotype of var. rehmannii and the subglabrous forms, e.g., from Inhaca Island. However, the tendency that Meeuse describes is apparent. Other characters in the collections of his variety, viz. relatively long calyx lobes and petals, also occur in the high mountains of Namibia but also in the whole periphery of the *Coccinia rehmannii* distribution range. Strangely, Meeuse does not mention the most striking difference between *Coccinia rehmannii* collections from the inland/dry areas and coastal/peripheral collections being the globose fruit in inland/dry area individuals or long elliptical fruit in coastal/peripheral individuals (shown and mentioned in this treatment as "Coccinia rehmannii aff. var. littoralis"; Fig. 10a) respectively. Collections with this fruit morph also occur in Angola and Zimbabwe but do not follow Meeuse’s other characters of the var. littoralis. On the other hand, collections with long calyx lobes and long petiolate flowers can have globose fruits. Like the area of the southeastern coast of South Africa, areas in the north of southern Africa receive more and longer rainfall per year than the inland, so there is a possible correlation between precipitation and fruit morphology. Elliptical fruits also occur in the closely related *Coccinia trilobata* from mountainous and thus more humid habitats but not in *Coccinia microphylla* from the dry bushlands of NE Africa whose fruit is globose. However, the characterization by Meeuse that *Coccinia rehmannii* is an aggregate species seems to be true. It might be interesting to link morphological characters with haplotypes and to test the fitness of these morphs in the different habitats. In any case, the morphological differentiation seems to be stable in cultivated individuals, and artificial crosses between different forms (inland vs. subglabrous from the Southeast) result in the onset of a normal fruit (resulting seeds were not used for cultivation).

The placement of Coccinia rehmannii var. littoralis forms with other *Coccinia rehmannii* forms in plastid and nuclear phylogenies (Figs 17, 18) also rejects the hypothesis of a hybrid origin (e.g., with *Coccinia adoensis* or *Coccinia senensis*), but rather suggests parallel evolution due to ecological factors.

#### Taxonomic remarks.

The protologue contains a literal mistake for the syntype from Eland River and must be corrected from 4944 to 4954. On the one hand, *A. Rehmann 4944* (GRA, K) is a Malpighiaceae. On the other hand, there is *A. Rehmann 4954* (a *Coccinia rehmannii*) in Z from Eland river and with a remark by Cogniaux ‘sp. nov.’ Hence, *A. Rehmann 4954* is the syntype of *Coccinia rehmannii*, not *A. Rehmann 4944*.

The GRA specimen of *H.G. Flanagan 457*, which is supposed to be the isotype (cited by Meeuse) of Coccinia rehmannii var. littoralis, is in fact merely a paratype. Meeuse stated clearly the location as “Komgha: Kei Mouth” and chose the PRE specimen from there as the holotype, but the GRA specimen is from the nearby located Cape Morgan. Apparently, Flanagan used the same collection number for different gatherings. The GRA specimen thus cannot be regarded as a duplicate despite the same number. As the GRA specimen does not have a label by Meeuse, he just cited the specimen without seeing it.

The similarity of Coccinia rehmannii var. littoralis to *Coccinia senensis* led to a misplaced paratype. One of the two specimens by R. de Carvalho is a syntype of Coccinia jatrophiifolia var. australis Cogn. and the two R. de Carvalho specimens from COI are paratypes of *Coccinia subglabra*, which are both synonyms of *Coccinia senensis*.

*Coccinia ovifera* is a validly published name, although the description is a little cryptic, hence the species name is not a *nomen nudum*. Dinter writes that he has found, viz. collected, the species around Grootfontein, in Hereroland (not in the narrow sense of the 1968 homeland) and in Sandverhaar (Namaland). Therefore, the requirements for validity are met (37.3 Note 2). The latter site is cited by him explicitly in a later publication (Dinter 1919/20). The collections designated as syntypes above match the description as given in the protologue. As the present author did not see the specimens designated as “possible syntypes”, they are tentative and might have been destroyed in Berlin.

#### Specimens examined.

(Selection, in total: 321) Angola. Huíla: Cambos, near Chiange, *A. Menezes 3629* (LISC [LISC 031347], P). Namibe: andados ca. 55 km de Moçamedes [Namibe] para Dois Irmãos [Caraculo], *E.J. Mendes 3969* (COI, LISC). Botswana. Ghanzi: 200 mls [320 km] NW of Molepolole, *R. Story 5029* (COI (2), EA (2), PRE (2), Z). Kgalagadi: c. 50 mls [80 km] NNW of Tsabong, *O.A. Leistner 3120* (LISU, M, PRE). Ngamiland: 80 km W of Tsau on Cae Cae road, *D.G. Long & D.A.H. Rae 416* (E). Mozambique. Gaza: Vila de João Belo [Xai-Xai], entre Chicumbane e a Barra do Limpopo: próximo da povoação commercial da Barra, *F. de Lemos & A. Balsinhas 131* (BM, COI) and *133* (BM, COI (2)). Inhambane: Pomene, in hotel area, *P.C.M. Jansen et al. 7533* (G n.v. [G00305107], MO, WAG [WAG0234198], WAG [WAG0234199]). Maputo: between Costa do sol and Marracuene, Mutanhane, *A. Balsinhas 230* (BM, COI, PRE). Namibia. Erongo: Farm Anschluss, 150 km E of Swakopmund on Khomas road to Windhoek, *B. de Winter & D. Hardy 8001* (M, PRE, WAG [WAG0234173]). Hardap: c. 20 mls [32 km] from Kalkrand on road to Rehoboth, *B. de Winter 3538* (COI (2), L, M, PRE). Karas: Dassiefontein Farm, 2–3 km E of highway, in foothills of Groot Karasberge, c. 64 km NNE of Grünau, *G. Davidse & A. Loxton 6240* (M, MO, S). Omaheke: [Farm] Breitenberg, border of Kalahari, *R.H.W. Seydel 2513* (COI, M, WAG [WAG0234172]). Otjozondjupa: 160 mls [257.5 km] E of Grootfontein, Gautscha Pan, *R. Story 6219* (PRE); ibid., *R. Story 6238* (M (2), PRE (2), S [S08-12417]). South Africa. Eastern Cape: Port St. Johns distr., First Beach, *M.J. Wells 3434* (MO). Gauteng: Pretoria, Brummeria: BRI, *A. Balsinhas 3474* (MO, PRE, WAG [WAG0234189]). KwaZulu-Natal: Lower Tugela Valley, below Maqumbi, *D. Edwards 3053* (B, M, HEID, PRE); 5 mls [8 km] on Nkonkoni–Pongola road, *M.J. Wells 2162* (B, EA, M, Z [Z-000060820]). Limpopo: Immerpan, near post office on roadside, *A.D.J. Meeuse 9452* (B, COI, L, PRE, Z [Z-000060813]). Mpumalanga: Impala, siding, *E. Retief 1260* (PRE); ibid., *E. Retief 1261* (MO, PRE); Steelport, Burgersfort, 2 km E of town, 24°40'S, 30°22'E, *H.J. Venter & A. Venter 10260* (S [S08-12382], WAG [WAG0234178]). Northern Cape: 25 ml. [40.2 km] W of Kimberley, *H.J.E. Schlieben & H.R. Tölken 11017* (G (2), HEID, M, PRE, S). Northwest: Rooisloot, 6 Apr 1935, *A.O.D. Mogg s.n.* (B, COI, L, PRE); Farm Welgevonden, 6 Apr 1935, *A.O.D. Mogg s.n.* (B, L, PRE [PRE43077], Z [Z-000060812]). Swaziland. Lubombo: Thsaneni [Tjaneni], *I.F. La Croix 4909* (MO, WAG [WAG0234170]). Zimbabwe. Manicaland: Chipinga district, Sabi Valley Experimental Station, *C. Soane 162* (COI (3)). Matabeleland South: Beit Bridge [Beitbridge], *A.W. Exell et al. 425* (LISC); ibid., *L.C. Leach 10700* (COI, MO).

### 20. Coccinia samburuensis

Taxon classificationPlantaeCucurbitalesCucurbitaceae

Holstein, Kew Bull. 65(3): 438. 2010 [published on 1 Jan 2011].

Coccinia samburuensis Type: Kenya. [Rift Valley Province]: Samburu East District, on Wamba-Isiolo road, 0.7 km S of turnoff to Maralal, c. 1300 m, female, fl, fr, 4 Jul 1974, *R.B. Faden & A.J. Faden 74/948* (Holotype: MO!, isotype: WAG! [WAG0234153]).
Coccinia samburuensis Type: Kenya. Rift Valley Province: Samburu District, Mt Nyiru, southern slopes, near a river, 2°03'N, 36°51'E, 1600 m, 1 Apr 1995, *B. Bytebier et al. 355* (Paratypes: EA (2)!). Type: Kenya. Operoi, 1°12'N, 36°49'E, 1350 m, rocky outcrop in *Acacia* woodland, 23 Dec 2004, *W.R.Q. Luke & P.A. Luke 10787* (Paratypes: EA!, K!).
Coccinia samburuensis Type: Kenya. Near Maralal, Lowaweregoi [Lowua Werekoi Mt] 4000 ft [c. 1220 m], rocks in bushland, 5 Dec 1958, *J.G.B. Newbould 3233* (Paratype: K!).

#### Description.

Perennial climber. Stems up to 5 m, glabrous, except for minute few-cellular trichomes visible under 5–10× magnification. Petioles glabrous, at base white speckles may occur. Leaves 6–14 cm × 10–17 cm wide, (5- or)7-lobate. Leaf lobes elliptical, margin serrate (to lobulate), teeth (lobule tips) with yellowish glands. Lobe apex subacute, apiculate. Upper leaf surface glabrous, more or less clear to white pustulate. Lower leaf surface glabrous, nerves white-speckled. Probracts up to 4 mm. Tendrils simple. Male flowers 1–2 solitary. Pedicel up to 5 cm long, glabrous. Perianth tube glabrous, calyx lobes 6.5 mm long, linear, erect. Corolla 3.7–4 cm long, brownish yellow, lobes 2.2–2.5 cm. Female flowers solitary. Pedicel 4–5 mm, glabrous. Hypanthium tube glabrous, calyx lobes and corolla like in males. Ovary narrowly cylindrical, glabrous. Fruits c. 14 × 1.5–2 cm, long cylindrical, unripe green with lighter spots, color of ripe fruit unknown but likely red. Seeds 6.5–7 × 3.5–4.5 × (≥ 1) mm (L/W/H), symmetrically obovate, face flatly lenticular.

#### Phenology.

Flowering time: Imperfectly known. Flowering in April, July, and December, but likely to flower as long water is available (rainy seasons).

#### Distribution.

Fig. 35. Only known from Samburu area in Kenya (hence the epithet). Only known from seepage line in rocky (granite) outcrops in *Acacia*-*Commiphora* deciduous bushland.

### 21. Coccinia schliebenii

Taxon classificationPlantaeCucurbitalesCucurbitaceae

Harms, Notizbl. Bot. Gart. Berlin-Dahlem 11: 685. 1932.

Coccinia schliebenii Type: Tanzania. Morogoro: Ulanga district, Mahenge ward, Mtimaliassi near Mahenge station. 900–1000 m, male, fl, 14 Jan 1932, *H.J.E. Schlieben 1620* (Holotype: B! [B 10 0154928, digital image: B, JPS], isotypes: BM! [BM001010003], BR! [BR0000008886828, digital image: BR, JPS], BR! [BR0000008887580, digital image: BR, JPS], G! [G00301595], M! [M0105774, digital image: JPS], MA [MA386129, digital image: JPS], P! [P00346274, digital image: JPS, P], S [S-G-1518, digital image: S], Z! [Z-000004447]).
Coccinia calophylla Harms in Mildbraed, Notizbl. Bot. Gart. Berlin-Dahlem 12: 522. 1935.
Coccinia schliebenii Type: Tanzania. Lindi: Muera plateau, Bakari, fl, 26 Oct 1934, *H.J.E. Schlieben 5551* (Holotype: B! [B 10 0154924, digital image: B, JPS], isotypes: BM! [BM001010001], BM! [BM001010002], BR! [BR0000008887153, digital image: BR, JPS], BR! [BR0000008887511, digital image: BR, JPS], G! [G00301767], G! [G00301768], HBG! [HBG506428, digital image: JPS], M! [M0105775, digital image: JPS], MA [MA386127, digital image: JPS], P! [P00346272, digital image: JPS, P], P! [P00346273, digital image: JPS, P], S [S08-11865, digital image: JPS, S], Z! [Z-000073408, digital image: Z], Z! [Z-000073409, digital image: Z]).

#### Description.

Perennial climber. Stems up to 12 m, densely covered with short, stiff, smutty-brownish trichomes. Petioles 1.5–11 cm, indumentum as on stem. Leaves 5–18 × 4.5–18 cm, slightly to deeply palmately 5-lobate. Lobes broadly triangulate to long elliptical, margin dentate, tips acute or obtuse. Upper leaf surface usually densely covered with short, thin trichomes. Lower leaf surface densely (esp. on nerves) covered with short, stiff, dirty-brownish-beige trichomes. Probracts up to 4.5 mm. Tendrils simple or bifid. Common peduncle 1.1–6.5 cm, with indumentum like on stem to puberulous, pedicels of flowers in racemes with up to 4 mm, indumentum as on peduncle, bracts 3–4 mm. Pedicels of solitary flowers 1.2–5 cm, indumentum as on peduncle. Perianth tube with indumentum like on stem to puberulous. Calyx lobes lineal to narrowly lanceolate 10–15 mm. Corolla 4–6.2 cm long, yellow, apricot, pale orange, sometimes marked with purple, lobes 2–3.2 cm. Filament color not seen, anther head not seen, pollen sacs dark yellow to orange. Female flowers solitary, pedicels 2.5–4.5 cm long, densely covered with short trichomes. Hypanthium with indumentum like on stem to puberulous, calyx lobes, and corolla like in male flowers. Ovary with smutty-brownish trichomes. Style 3–6 mm, color not seen. Stigmas 2-lobed, orange-yellow. Fruit 7–9 × c. 2.5 cm long, oblong to shortly cylindrical, ripening from green with 10 more deeply colored ribs via yellow to red. Seeds 5.5–6 × 2.5–3 × 1 mm (L/W/H), symmetrically obovate, face lenticular.

#### Phenology.

Flowering time: January–March, May–July, December.

#### Distribution.

**Figure 38.**
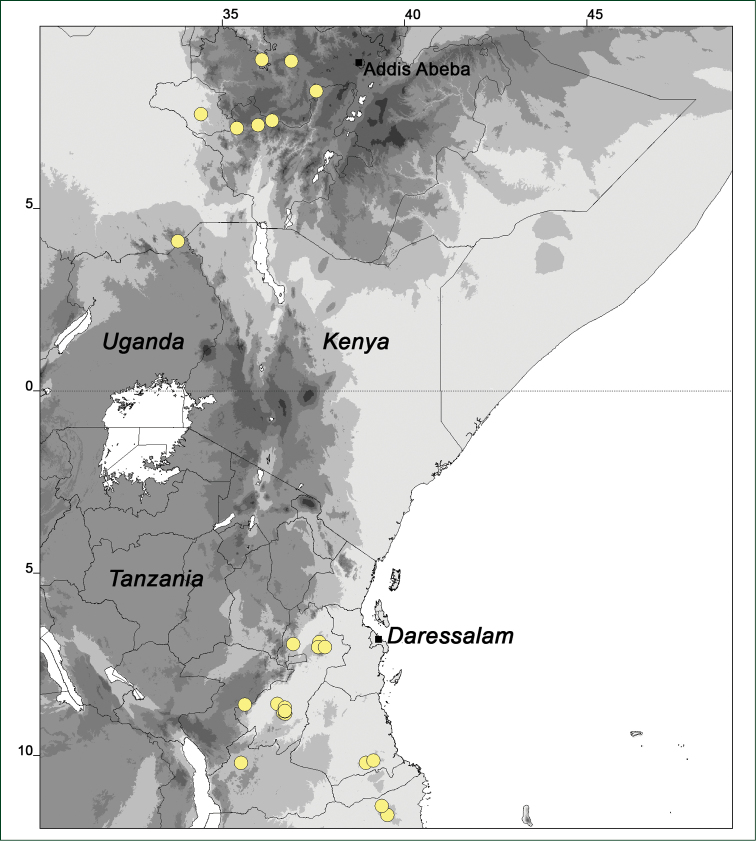
Distribution map of *Coccinia schliebenii* (pale yellow dots; based on 27 collections). For Ethiopia and Tanzania the borders of the regions are given.

Fig. 38. Ethiopia (Benishangul-Gomaz?, Gambela, Oromia, SNNPR), Mozambique (Cabo Delgado), South Sudan (Eastern Equatoria), Tanzania (Iringa, Lindi, Morogoro, Mtwara?, Ruvuma). Elevation 300–1900 m. Black cotton soil, reddish soil, volcanic underground. *Markhamia*-*Dombeya* woodland, *Chlorophora*-*Albizia* woodland, *Dalbergia*-*Pterocarpus*-*Combretum*-*Acacia* woodland, woodland with *Acanthus sennii*, *Baphia abyssinica* forests, lower afromontane forests, termite hills, gallery forests.

#### Use.

Fruits edible (*W.J. Kindeketa et al. 2793*).

#### Vernacular names.

Didinga: moroich (*J.G. Myers 10918*), Kipogoro: mdalla (*W.J. Kindeketa 2747*), Mokonde: ncauedi (*M.F. Correia 92*).

#### Specimens examined.

(Selection, in total: 31) Ethiopia. Gambela: 20 km E of Punido, along the new road to Gog, 7°34'N, 34°24'E, *I. Friis et al. 7317* (C, K). Oromia: c. 25 km E of Lekemti [Nekemte], *W.J.J.O. de Wilde & B.E.E. de Wilde-Duyfjes 7184* (K, MO, WAG [WAG0225409], WAG [WAG0225410], WAG [WAG0225411]). SNNPR: Ghibie [Gibe] or upper Omo gorge, 182 km SW of Addis Abeba on road to Jimma, north bank, *J.W. Ash 898* (EA (2), K). Mozambique. Cabo Delgado: Macondes, 2 km from Mueda to Negomano, near Santo António mission, *M.F. Correia 92* (LISC). South Sudan. Eastern Equatoria: Didinga Mts, Mt Lotuke, Char, *J.G. Myers 10918* (K). Tanzania. Iringa: Mufindi district, Lulanda village, N and NW of Ihili forest patch, 8°35'59"S, 35°37'12"E, *M.A. Mwangoka & C.J. Kayombo 63* (MA n.v., MO, P [P05620800]). Lindi: Rondo Plateau, *E. Milne-Redhead & P. Taylor 7630* (EA, K). Morogoro: Ulanga district, Kitonga subvillage, 8°46'46"S, 36°42'34"E, *G.S. Laizer et al. 1449* (BM, MO). Ruvuma: [near Gumbiro], by R. Mtandazi [river], *E. Milne-Redhead & P. Taylor 8538* (B [B 10 0312902], EA, K, LISC, P [P05620801]) and *8539* (EA, P [P05620802]).

### 22. Coccinia senensis

Taxon classificationPlantaeCucurbitalesCucurbitaceae

(Klotzsch) Cogn. in A.DC. & C.DC., Monogr. Phan. 3: 535. 1881.

Cephalandra senensis Klotzsch in W.C.H. Peters, Naturw. Reise Mossambique: 151. 1862.
Coccinia senensis Type: Mozambique. [Zambézia Province]: Rios de Sena [province], without detailed locality, in grassland, *W.C.H. Peters s.n.* (Holotype: B, destroyed).
Coccinia senensis Type: Tanzania, Lindi Region: 40 km W of Lindi, Lake Lutamba, hill, woodland, climbing over bushes, c. 240 m, male, fl, 6 Sep 1934, *H.J.E. Schlieben 5259* (Neotype, designated in Holstein and Renner (2010: 441): M! [M-0165202]; isoneotypes: B! [B 10 0379053, digital image: B], BM!, G! [G00301596], HBG! [HBG518898], MO!, P!, S! [S08-12156, digital image: S], Z!).
Coccinia jatrophiifolia (A.Rich.) Cogn. var. australis Cogn. [sphalm.: Coccinia jatrophæfolia var. australis Cogn.] Bol. Soc. Brot. ser. 1, 7: 228. 1889.
Coccinia senensis Type: Mozambique. [Nampula]: Mossuril et Cabaceira (Zambézia), male, fl, 1884, *R. de Carvalho 15* (Lectotype, designated here: BR!).
Coccinia senensis Type: Mozambique. Ibid., male, fl, 1884–1885, *R. de Carvalho s.n.* (isolectotype: COI!).
Coccinia fernandesiana C.Jeffrey, Kew Bull. 30(3): 478. 1975.
Coccinia senensis Type: Mozambique. Niassa: Erati, between Namapa and Ocúa, near river Lúrio bridge, female, fl, fr, 9 Mar 1960, *F. de Lemos & L. Macuácua 29* (Holotype: COI, isotypes: BM! [BM001010006], BM! [BM001010007], K!, LISC! [LISC 002485, digital image: IICT, JPS], LMA, PRE! [PRE0592949-0, digital image: JPS], PRE! [PRE0592950-0, digital image: JPS], SRGH! [SRGH0106711-1, digital image: JPS], SRGH! [SRGH0106711-2, digital image: JPS], SRGH! [SRGH0106711-3, digital image: JPS]).
Coccinia senensis Type: Tanzania. Mtwara: Masai Distr. [*sic*, must be Masasi Distr.], W of R. Bangala, 390 m, in woodland on gravelly soil, 17 Dec 1955, *E. Milne-Redhead & P. Taylor 7703* (Paratypes: EA!, K (2)!, LISC!, P!).
Coccinia senensis Type: Tanzania. Lindi: Mlinguru, 275 m, shrub woodland, 18 Dec 1934, *H.J.E. Schlieben 5745* (Paratypes: B! [B 10 0379052, digital image: B], B!, BR!, EA!, HBG! [HBG518899], K (2)!, P!, LISC!, MO!, PRE!, SRGH).
Coccinia senensis Type: L. cl., *F. de Lemos & L. Macuácua 30* (Paratypes: BM! [BM001010005], COI! [2 sheets], K!, LISC! [LISC 002482, digital image: IICT, JPS], LMA, P!, SRGH).
Coccinia senensis Type: Mozambique. Niassa: Erati, between Namapa and Nacarea, *F.A. Mendonça 1128* (Paratypes: LISC! [LISC 002483, digital image: IICT, JPS]).
Coccinia senensis Type: Ibid., *F.A. Mendonça 1129* (Paratypes: LISC! [LISC 002484, digital image: IICT, JPS]).
Coccinia senensis Type: Mozambique. Zambézia: Milange, 95 km towards Quelimane, *A.R. Torre & M.F. Correia 14060* (Paratypes: K! [K000313233, digital image: JPS, K], LISC! [LISC 002481, digital image: IICT, JPS]). Type: Tanzania. Lindi: Nachingwea, *Pterocarpus*-*Combretum* woodland, *B. Anderson 815* (Paratypes: EA!, K!, NHT!).
Coccinia senensis Type: Tanzania. Lindi: Mbemkuru [also called Mbwenburu, Mto Bwamkuro], in deciduous thicket by roadside, 135 m, *E. Milne-Redhead & P. Taylor 7473* (Paratypes: BR!, EA!, K (2)!, P!), *E. Milne-Redhead & P. Taylor 7473A* (Paratypes: K (2)!), *E. Milne-Redhead & P. Taylor 7473B* (Paratype: K), *E. Milne-Redhead & P. Taylor 7473C* (Paratype: K), *E. Milne-Redhead & P. Taylor 7473D* (Paratype: K).
Coccinia subglabra C.Jeffrey, Kew Bull. 30(3): 479. 1975.
Coccinia senensis Type: Mozambique. Nampula: Nacala, 11 km from Itoculo towards Nacala, 130 m, male, fl, 4 Dec 1963, *A.R. Torre & J. Paiva 9417* (Holotype: LISC [LISC 002486, digital image: IICT, JPS]; isotypes: COI, K! [K000313458, digital image: JPS, K], LMA).
Coccinia senensis Type: Mozambique. Ibid., *A.R. Torre & J. Paiva 9417A* (Paratypes: COI, K! [K000313457, digital image: JPS, K], LISC, LMA).
Coccinia senensis Type: Mozambique. Niassa: Ruvuma River, *J. Kirk s.n.* (Paratype: K!).
Coccinia senensis Type: Mozambique. Nampula: Mossuril e Cabaceira, *R. de Carvalho s.n.* (Paratypes: COI (2)!).
Coccinia senensis Type: Mozambique. Zambézia: 23 km on road [from vila] Maganja da Costa [= Olinga] towards Namacurra, *A.R. Torre & M.F. Correia 14176* (Paratypes: EA!, LISC [LISC 002487, digital image: IICT, JPS], MO!).

#### Description.

**Figure 39.**
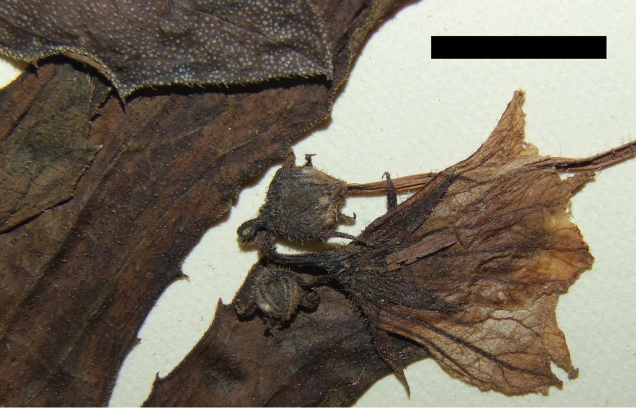
Inflorescence of a male *Coccinia senensis*, note the long triangulate (may be narrower in other collections, then subulate) calyx lobes in contrast to the calyx lobes of Coccinia adoensis var. adoensis in Fig. 21; picture taken from the neotype (*H.J.E. Schlieben 5259* (M)). Black bar equals 1 cm.

Perennial climber or creeper. Stems up to 3 m, glabrous or with erect, stiff, articulate, pale trichomes, glabrescent, when older sometimes with white pustules. Leaves subsessile or distinctly but not long petiolate. Petiole 0.4–4 cm long, abaxial side more or less densely covered with erect, stiff, articulate, pale trichomes, sometimes glabrous. Leaves 4–14 × 5–16 cm, cordate or shallowly to deeply 3- or 5-lobate, sometimes auriculate (Fig. 6). Lobes triangulate, lanceolate to lineal, sometimes with broader end. Finely to coarsely serrate-dentate, especially towards the lobe tips, sometimes lobulate. Lobe apices acute to subacute, with an apical tooth. Upper leaf surface with erect, stiff, articulate, pale trichomes or with whitish pustules. Lower leaf surface glabrous or covered with erect, stiff, articulate, pale trichomes, nerves with same indumentum or with white pustules. Between the main nerves at lamina base often with blackish glands. Probracts up to 4 mm. Tendrils simple, rarely bifid. Male flowers (Fig. 39) in few-flowered racemes, sometimes accompanied with 1–2 solitary flowers. Common peduncle 0.3–6.2 cm long, often more or less densely covered with erect, stiff, articulate, pale trichomes. Petiole of flowers in racemes 0.2–1.2 cm. Bracts up to 2.5 mm, often missing. Petioles of solitary flowers 3.2–6.2 cm long. Petioles in each case with indumentum of peduncle. Perianth tube glabrous or with erect, stiff, articulate, pale trichomes. Calyx lobes 2.5–6.5 mm long, subulate to narrowly triangulate-lanceolate. Corolla 1.1–3 cm, yellow, orange to salmon, lobes 0.9–1.1 cm. Color of filament column, anther head, and pollen sacs not seen. Pedicel of female flower 1.2–2.3 cm. Hypanthium, calyx lobes, and corolla like in male flowers. Ovary glabrous or more or less covered with erect, articulate trichomes. Style and stigma not seen. Fruit 3.5–4.4 × 1–1.5 cm, long ovoid to shortly cylindrical, often with apical sterile tip. Unripe pale green with dark green longitudinal lines, ripe red. Seeds 5.5–7 × 3–5 × 1.2–1.3 mm (L/W/H), symmetrically obovate, face lenticular.

#### Phenology.

Flowering time: January–April, September, December.

#### Distribution.

Fig. 23. Central Tanzania (Iringa, Lindi, Morogoro, Ruvuma), Malawi (Southern Region), Mozambique (Cabo Delgado, Nampula, Tete, Zambezia). Elevation 0–700 m. Sandy soil. *Coccinia senensis* seems to be a typical element of the Zambezian center of endemism (White 1983b). Deciduous woodlands, termitaria, riverine thickets, coastal forests.

#### Vernacular names.

Kihehe: mtumbulansoka (*W. Carmichael 171*), Macua [Makhuwa]: muuco-uco (*F. de Lemos & L. Macuácua 29*)

#### Remarks.

The species is recognizable by the combination of few-flowered racemes, long subulate calyx lobes, and the often subsessile leaves. The trichome type (often appearing articulate when dried) is the same as in *Coccinia rehmannii*, where (sub-)glabrous collections also occur (see also the Taxonomic remarks). Except for the degree of trichome density, a subglabrous collection (*E.M.C. Groenendijk et al. 1031*) from 11 km from the collecting site of the *Coccinia subglabra* holotype was neither morphologically nor genetically (Holstein and Renner 2011b) distinguishable from *Coccinia fernandesiana*, and *Coccinia senensis* (sensu Flora Zambesiaca (Jeffrey 1978) and sensu Holstein and Renner (2010)). Fruit shape and length as well as the length of the female pedicel are variable, so *Coccinia subglabra* is synonymized. Without calyx lobes, *Coccinia senensis* is only hardly, if at all, distinguishable from glabrous *Coccinia adoensis* collections or those with long articulate trichomes, which are described as Coccinia adoensis var. jeffreyana in this treatment. The fruit and seed shape also match the variable *Coccinia adoensis*. Usually, Coccinia adoensis var. adoensis has short trichomes and calyx lobes are ≤ 2 mm, but where both species meet (Malawi, NW Mozambique, S Tanzania), exceptions can be found (listed and further discussed as Coccinia adoensis var. jeffreyana). Whether *Coccinia adoensis* and *Coccinia senensis* are truly separate species and the role of these intermediates needs to be tested by artificial hybridization, field observations, and/or a phylogeographic analysis.

#### Taxonomic remarks.

Although the holotype of *Coccinia senensis* burned during the destruction of the Berlin herbarium in 1943, and the name appears to have been lost, the protologue mentions several characters that allow *Coccinia senensis* to be synonymized with Jeffrey’s *Coccinia fernandesiana*. The *Coccinia senensis* protologue points out “articulate” trichomes and an overall appearance like *Coccinia quinqueloba*, which matches perfectly with many collections of *Coccinia fernandesiana*. Interestingly, many of these collections have been identified as “*Coccinia quinqueloba*” or “*Coccinia palmata*” by various collectors and scientists. The similarity, including the calyx lobes, is easily visible in many collections, but both species are restricted to southern Africa.

Cogniaux described var. australis of *Coccinia jatrophiifolia* (synonym to *Coccinia adoensis*) recognizing the similarity to the polymorphic *Coccinia adoensis*. However, he differentiated between the *R. de Carvalho* specimens with long lineal lobes (BR, COI) and specimens with lanceolate lobes (BR, COI), which he determined as *Coccinia senensis*. When Jeffrey described *Coccinia subglabra*, he cited the two COI specimens (as deduced from his ID labels), but he did not refer to Cogniaux’ variety, which must have been overlooked. The one COI specimen is therefore paratype of *Coccinia subglabra* and syntype of Coccinia jatrophiifolia var. australis. The two COI specimens are also misplaced paratypes of Meeuse’s Coccinia rehmannii var. littoralis. The similarity of the COI specimens of Meeuse’s variety to *Coccinia senensis* is striking, but the long peduncles and the conspicuous black sublaminal glands refer rather to *Coccinia senensis* than to *Coccinia rehmannii*.

#### Specimens examined.

(Selection, in total: 49) Malawi. Northern Region: Rumphi district, Nyika Plateau, 20 mls N of M1, *J. Pawek 13339B* (MO). Southern Region: Bvumbwe, *I.F. La Croix 2653* (MO); Lengwe National Park, near Mukanyu ravine, *A. Hall Marker 1051* (K). Mozambique. Cabo Delgado: Mueda Plateau, 11°22'S, 39°20'E, *W.R.Q. Luke et al. 10084* (EA, K). Nampula: Monapo district, Monapo, forest reserve of Mr. Wolf, *E.M.C. Groenendijk et al. 1031* (WAG [WAG0104327]). Tete: Cabora bassa [Cahora bassa], police post no. 3, 5 km from barrage, *A.R. Torre et al. 18788* (MO). Tanzania. Lindi: Selous Game Reserve, Kingupira, 8°28'S, 38°33'E, *K. Vollesen 1908* (EA); ibid., *K. Vollesen MRC 4316* (DSM, EA, K, WAG [WAG0234144]); ibid., *R.C. Wingfield et al. 3466* (DSM). Morogoro: Kilosa district, Ilonga Research Institute, 9.5 km NNE of Kilosa on road to Dumila, 6°46'31.3"S 37°2'23"E, *N. Holstein et al. 66* (DSM, M).

### 23a. Coccinia sessilifolia (Sond.) Cogn. var. sessilifolia

Taxon classificationPlantaeCucurbitalesCucurbitaceae

Cephalandra sessilifolia Sond. in Harv. & Sond., Fl. Cap. 2: 493. 1862. *Coccinia sessilifolia* (Sond.) Cogn. in A.DC. & C.DC., Monogr. Phan. 3: 534. 1881.
Coccinia sessilifolia (Sond.) Cogn. var. sessilifolia Type: South Africa. Vaal river, *J. Burke 289* (Syntypes: K! [K000313207, digital image: K], PRE, SAM).
Coccinia sessilifolia (Sond.) Cogn. var. sessilifolia Type: South Africa. Slengerfontein in Nieuwe Hantom [area where the provinces Western Cape, Eastern Cape and Free State meet], on rocks, 4500–5000 ft, female, fr, 1839, *J.F. Drège 3375* (Lectotype, designated here: P! [P00346268, digital image: P]).
Coccinia sessilifolia (Sond.) Cogn. var. sessilifolia Type: South Africa. Nieuwe Hantom, on rocks, 4500–5000 ft, 1839, *J.F. Drège s.n.* (Syntypes: BR! [BR0000005111596, digital image: BR], G! [G00301769], K! [K000313206, digital image: K], L!, P! [P00346270, digital image: P], S! [S08-12468, digital image: JPS, S], W! [W 0026938: digital image: WU]).
Coccinia sessilifolia (Sond.) Cogn. var. sessilifolia Type: South Africa. Transvaal, *C.L.P. Zeyher 580* (Syntypes: BM! p.p., E! [E00303259], K! p.p. [K000313205, digital image: K], P! p.p. [P00346271, digital image: P]).
Coccinia sessilifolia var. major Cogn. in Schinz, Verh. Bot. Ver. Provinz Brandenburg 30: 152. 1889.
Coccinia sessilifolia (Sond.) Cogn. var. sessilifolia Type: Namibia. Hereroland, male, fl, 1885, *A. Lüderitz 133* (Lectotype, designated here: Z!).
Coccinia sessilifolia (Sond.) Cogn. var. sessilifolia Type: Namibia. Walvis bay to Odyitambi, Dec 1885–Feb 1886, *A. Lüderitz 1a* (Syntype: Z!).
Coccinia schinzii Cogn., Bull. Herb. Boiss. 3: 419. 1895.
Coccinia sessilifolia (Sond.) Cogn. var. sessilifolia Type: South Africa. Transvaal: Klippan [according to Meuse (1962) in Limpopo: Greater Sikhukhune District Municipality, Doornpoort; 24°37'S, 29°26'E], bushveld, 1875–1880, *A. Rehmann 5162* (Lectotype, designated by Meeuse (1962: 98): Z! [Z-000004446, digital image: Z], isolectotype: BR! [BR0000005112265, digital image: BR, JPS]).

#### Description.

Perennial climber or creeper. Stems up to 5 m long, with slight waxy cover, glaucous (Figs 2b, 7b), glabrous (first shoots may have short, white trichomes). Leaves sessile to amplexicaul (first leaves after appearance of stem can be distinctly petiolate, rarely also when mature (up to 1.5 cm; Figs 2b, 4b, 7b)), glaucous, 1.5–12.5 × 2.2–13.5 cm, (cordate to) deeply palmately 5-lobate. Lobes linear, lanceolate to elliptic. Leaf margin remotely denticulate, with or without lobules. Lobe apex obtuse to acute, apiculate. Upper leaf surface glabrous, clear to white pustulate. Lower leaf surface glabrous, sometimes with dark glands near base of lamina. Probracts up to 1.7 mm or missing. Tendrils simple, very rarely bifid. Male flowers solitary or clustered in few-(rarely many-)flowered racemes (Figs 2b, 7b). Pedicels of solitary flowers 1–4 cm, glabrous. Peduncle 1–6 cm long, glabrous. Pedicels of flowers in racemes 0.3–2.5 cm, glabrous. Bracts glabrous, up to 1.8 mm, or missing. Perianth tube glabrous, calyx lobes 1.5–3.5 mm long, lanceolate to (narrow) triangulate, erect to reflexed. Corolla 1.5–3 cm long, whitish cream to pale yellow, rarely dull orange-brown with conspicuous green venation, lobes 0.9–2 cm. Filament column not seen, anther head not seen, pollen sacs yellow. Female flowers one solitary. Pedicel 1–3 cm, glabrous. Hypanthium glabrous, calyx and corolla like in males. Ovary glabrous. Style columnar, greenish yellow. Stigmas bulging, greenish yellow. Fruit 8–12 × 3–4 cm, ellipsoid to oblong, when immature green with white longitudinal spots to stripes with waxy bloom, ripe red (Fig. 13b). Seeds 6–8 × 3–3.5 × 1–1.5 mm (L/W/H), symmetrically to slightly asymmetrically obovate, face flat (Fig. 14d, e).

#### Phenology.

Flowering time: January–May, October–December.

#### Distribution.

**Figure 40.**
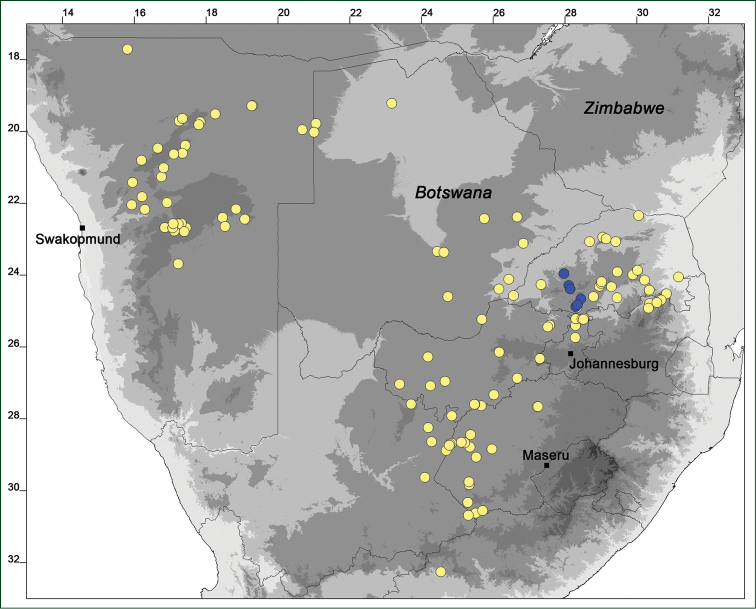
Distribution map of Coccinia sessilifolia var. sessilifolia (pale yellow dots; based on 133 collections) and Coccinia sessilifolia var. variifolia (blue dots; based on 7 collections). For South Africa the borders of the provinces are given.

Fig. 40. South Africa, Botswana, Namibia, except high mountains, hyper-arid regions, and Cape floristic region. Elevation 300–1500 m. Stony soil, sand, sandy loam, clay loam. Granite or calcareous substrate. Semi-desert, grassland (e.g., *Rhynchelytrum* sp.), bushland, open woodland. Full sun to shade. Moderate disturbance tolerated.

#### Use.

Unripe fruits are baked in ashes and eaten (Dinter 1912), ripe fruits edible (*N. Holstein 119*, *R.H.W. Seydel 3829*, *R.H.W. Seydel 4100*).

#### Vernacular names.

Afrikaans: bobbejaan komkommer (*C.A. Smith 3981*), Otjiherero: ekungu (singular), omakungu (plural) (Dinter 1912; 1919/20), Tswana: mogábalá (*J. Snyman & C. Noailles 229*), !Kun [Kung]: kitwa (*R. Story 5167*).

#### Taxonomic remarks.

The *C.L.P. Zeyher 580* specimens (syntype) in BM, K, and P are mixed with a *Trochomeria* sp.

#### Specimens examined.

(Selection, in total: 184) Botswana. Central District: Mahalapye, 2 mls [3.8 km] SW of Kalamare, *H.J. van Rensburg B4019* (PRE). Kgatleng: 15 km SE of Artesia (Mosomane), *D.T. Cole 1542* (PRE). North-West District: Aha Hills, *H. Wild & R.B. Drummond 6953* (COI). South-East District: Lobatsi [Lobatse], *F.A. Rogers 6281* (G, Z). Namibia. Erongo: Karibib, Okongawa, *R.H.W. Seydel 3026* (B (2), COI, FR (2), G (3), H, HEID, M, WAG [WAG0234195], WAG [WAG0234197]). Khomas: [Farm] Aris, mountain in the west, *R.H.W. Seydel 4100* (B (2), M, MO) and *4100a* (B (2)). Oshana: Amboland, Uukuanjama [Oukwanyama], Omupanda, *A. Wulfhorst 18* (Z). Otjozondjupa: 32 mls [51.2 km] N of Nurugas on road to Karakuwisa, *B. de Winter 3710* (M, PRE). South Africa. Eastern Cape: in valley near Graaff-Reinet, *H. Bolus 364* (S [S08-12381]). Free State: Kroonstad townland, NE of confluence of Blomspruit and Vals river, *J.C. Scheepers 1720* (EA, LISU, PRE, S [S08-12461). Gauteng: Pretoria, Brummeria, Botanical Garden, *A. Balsinhas 3406* (MO, PRE, WAG [WAG0234191]); ibid., *A. Balsinhas 3476* (MO, PRE, WAG [WAG0234190]). Limpopo: Penge mine, *E. Retief 1354* (MO, PRE, WAG [WAG0234188]); c. 30 mls [48 km] W of Louis Trichardt, western part of Zoutpansberg, near Mara, Buysdorp, *H.J.E. Schlieben 7453* (B, G, HBG, M). Mpumalanga: Blyderivierspoort Nature Reserve, Sybrand van Niekerk resort at camp area, *E. Retief 1340* (PRE, MO, WAG [WAG0234187]), ibid., *E. Retief 1341* (PRE). Northern Cape: Colesburg, Achtertang, 16 Apr 1934, *J.O. Swinford s.n.* (PRE [PRE42988]). North West: near Klerksdorp, *H.J.E. Schlieben 10695* (HEID, PRE, S [S08-12463]); 60 mls [96 km] NW of Vryburg, Farm Palmyra, *R.J. Rodin 3605* (MO, P [P05620787], PRE).

### 23b. Coccinia sessilifolia var. variifolia

Taxon classificationPlantaeCucurbitalesCucurbitaceae

(A.Meeuse) Holstein stat. nov.

urn:lsid:ipni.org:names:77148916-1

Coccinia variifolia A.Meeuse, Bothalia 8: 100. 1962.
Coccinia sessilifolia var. variifolia Type: South Africa. [Limpopo]: Waterberg, Vaalwater, about 2.25 km from Vaalwater on road to Hermanusdoorns, male, fl, 6 Jan 1959, *A.D.J. Meeuse & R.G. Strey 10413* (Holotype: PRE [2 sheets: PRE0188239-1 and PRE0188239-2, digital image: JPS], isotypes: BOL, L, SRGH).
Coccinia sessilifolia var. variifolia Type: South Africa. Ibid., *A.D.J. Meeuse & R.G. Strey 10413bis* (Paratype: PRE!).
Coccinia sessilifolia var. variifolia Type: South Africa. Limpopo: Palala river, *M.G. Breyer[-Brandwijk] TRV25226* (Paratype: ?).
Coccinia sessilifolia var. variifolia Type: South Africa. Limpopo: Rietspruit near Nylstroom [Modimolle], *G.P.F. van Dam TRV23372* (Paratype: PRE!).
Coccinia sessilifolia var. variifolia Type: South Africa. Limpopo: Nabomspruit, Mosdene, *E.E. Galpin s.n.* (Paratype: ?).
Coccinia sessilifolia var. variifolia Type: South Africa. Limpopo: 11 km from Warmbaths [Bela Bela] on Nylstroom road, *R. Story 1525* (Paratype: ?).
Coccinia sessilifolia var. variifolia Type: South Africa. Limpopo: Warmbaths, c. 3600 ft [1100 m], grassland/bush veld, *H. Bolus 11893* (Paratype: BR!).
Coccinia sessilifolia var. variifolia Type: South Africa. Ibid.?, *R. Leendertz TRV7579* (Paratype: ?).
Coccinia sessilifolia var. variifolia Type: South Africa. [Limpopo]: Waterberg, 5.5 mls [8.85 km] NNE of Warmbaths, c. 1220 m, sour bushveld, *J.P.H. Acocks 13903* (Paratype: S! [S08-12475]).

#### Description.

Perennial climber. Stems up to 1.2 m, likely also longer, glabrous. Petiole 0.7–1.6 cm, glabrous. Leaves 5.2–6 × 6–7.5 cm, deeply to shallowly 5-lobate, lobes outwards lobulate. Leaf margin remotely dentate, apex obtuse with final tooth. Upper leaf surface glabrous, with clear to whitish pustules. Lower leaf surface glabrous, with glands at base between nerves. Probracts up to 2 mm. Tendrils simple. Male flowers in racemes, accompanied by a solitary flower. Common peduncle 1–1.4 cm, pedicel in racemes 3–6 mm, each glabrous. Bracts up to 1.5 mm, narrowly ovate. Pedicel of solitary flowers 0.9–2 cm, glabrous. Perianth tube glabrous. Calyx lobes 2.5–4 mm, subulate to narrowly triangulate, erect. Corolla c. 2 cm, pale buff, lobes not measured. Filament column, anther head, and pollen sacs not seen. Female flowers solitary or clustered in reduced 2-flowered racemes. Common peduncle 1 mm, pedicel in racemes 0.9–1 mm, pedicel of solitary flower not seen, each glabrous. Hypanthium most likely glabrous and perianth as in male flowers. Ovary glabrous. Style and stigma not seen. Fruit and seeds not seen.

#### Phenology.

Flowering time: January–March, November, December. Likely as in Coccinia sessilifolia var. sessilifolia.

#### Distribution.

Fig. 40. Only known from Limpopo Province in South Africa. Elevation 800–1200 m. On sandstone, well-drained stony sand. Low closed woodland.

#### Remarks.

The new status of *Coccinia variifolia* was chosen due to the minor differences to *Coccinia sessilifolia* s.str. Subsessile leaves spontaneously occur in *Coccinia sessilifolia* (*H. Bolus 364*, *F.A. Rogers 19262*) and young individuals usually (always?) have petiolate leaves (*N. Holstein 131*, Fig. 4b). However, these collections of mature plants with distinctly petiolate leaves have only been observed in Limpopo Province in South Africa. Sessile *Coccinia sessilifolia* leaves can be quite variable, profoundly to deeply lobate, sometimes also lobulate. Compared to the rather uniform *Coccinia quinqueloba* (Meeuse 1962), the leaves thus appear to be extraordinarily variable. Meeuse’s *Coccinia variifolia* shares the sublaminal glands (cp. Fig. 7b) and the calyx lobes of *Coccinia sessilifolia*, and it is geographically nested within this species (hence no climatic differentiation). Acocks (*J.P.H. Acocks 13903*) also reports a “stark glaucous” appearance, just as in *Coccinia sessilifolia*. As petiolate leaves also occur in young *Coccinia sessilifolia* plants, and subsessile leaves also occur in mature plants, it is more likely that the distinctly petiolate *Coccinia sessilifolia* individuals represent a local fixation of this character. As Coccinia sessilifolia var. sessilifolia is derived from petiolate plants, this variety might even represent a remnant population of these.

#### Specimens examined.

(in total: 8) South Africa. Limpopo: Waterberg Distr., *F.A. Rogers 24932* (Z [Z-000073427]), *N. Rooyen 1667* (PRE), *R.H. Westfall 2136* (PRE).

### 24. Coccinia subsessiliflora

Taxon classificationPlantaeCucurbitalesCucurbitaceae

Cogn., Bull. Jard. Bot. État Brux. 4(1): 225. 1914.

Coccinia subsessiliflora Type: D. R. Congo. [Équateur Province]: around Likimi, male, fl, 15 Oct 1910, *L.C.E. Malchair 433* (Lectotype, designated here: BR! [BR0000008887481, digital image: BR, JPS], syntype: BR! [BR0000008886835, digital image: BR, JPS]).
Coccinia sp. D in C.Jeffrey, F. T. E. A.: 70. 1967. Uganda. [Western Region]: Kigezi District [(Kanungu District/Kisoro District)], Kayonza Forest Reserve [Bwindi Forest Reserve / Impenetrable Central Forest Reserve], *S. Paulo 644* (EA!, K!, MO!); [Central Region]: Mengo district, Mabira forest, *M.V. Loveridge 87* (?); [Central Region]: Mabira Forest, near Kiwala, *R.A. Dummer 3195* (?).

#### Description.

Perennial creeper or climber. Stems up to 4 m, glabrous. Petiole 2–12 cm, glabrous, sometimes with white pustules. Leaves 5.5–15 × 6.5–17.5 cm, almost to the base palmately 5-lobate. Lobes lanceolate, sometimes lobulate; tip acute, acuminate. Margin serrate-lobulate, denticulate. Upper leaf surface glabrous with clear to white pustules, rarely with few fine (up to 1.5 mm long) trichomes. Lower leaf surface glabrous, rarely with dispersed small blackish glands, rarely with tiny trichomes; sometimes nerves with white pustules. Probracts up to 1.5 mm or missing. Tendrils simple. Male flowers in glabrous, dense, compact racemes. Peduncles up to 6 mm. Pedicels up to 4 mm. Bracts 2–2.5 mm. Perianth tube glabrous. Calyx lobes 1–2 mm, subulate, triangulate to lineal, erect to reflexed. Corolla 1.2–1.3 cm, orange, pale yellow-orange, yellow, lobes c. 3 mm. Filament column, anther head, and pollen sacs not seen. Female flowers solitary or in few flowered racemes. Common peduncle up 1 cm, glabrous. Pedicel of flowers in racemes up to 4 mm. Bracts up to 2 mm or missing. Pedicel of solitary flowers up to 1.1 cm, glabrous. Hypanthium glabrous, calyx lobes and corolla like in males. Ovary glabrous. Style columnar, pale yellow. Stigma 2-lobed, yellow. Fruit 2–2.4(–7) × 1.7 cm, globose to long ovoid, unripe green with glaucous waxy cover, ripe color not known, most likely red. Seeds ≥ 4.5 × 2–2.5 × 1–1.5 mm (L/W/H), asymmetrically obovate, face flat.

#### Phenology.

Flowering time: January, April, July, August, October, December.

#### Distribution.

**Figure 41.**
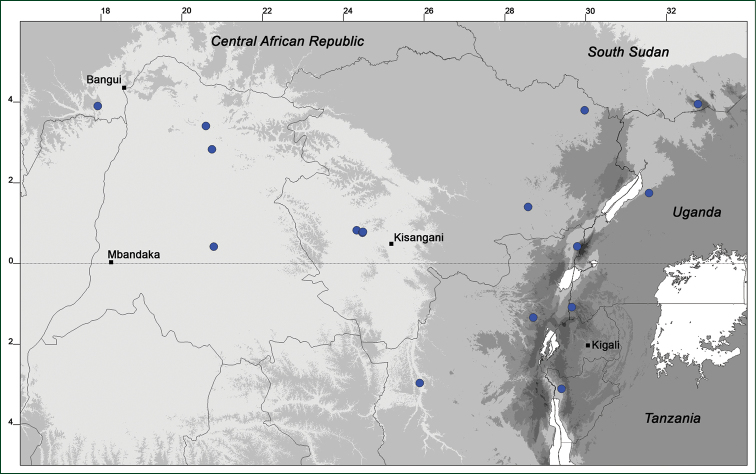
Distribution map of *Coccinia subsessiliflora* (blue dots; based on 18 collections). For D.R. Congo the borders of the provinces (until 1988) are given.

Fig. 41. Congo basin (Central African Republic, D. R. Congo). Forested mountains of NW Burundi, D. R. Congo (North Kivu, South Kivu), W Rwanda, Uganda (Western Province), South Sudan (Eastern Equatoria/Sharq al-’Istiwa’iyah: Lotti Forest). Elevation 300–1950 m. Soil preferences unknown. Tropical rainforests. *Macrolobium* [= *Gilbertiodendron*?] forest, swamp forest, disturbed ground in open forest.

#### Vernacular names.

Kihunde: mutangatanga (*R. Gutzwiller 965*), Lissongo [Mapti]: kanganga (*C.Tisserant (Équipe) 1103*), Turumbu: ndombo (*J. Louis 2709*).

#### Taxonomic remarks.

There are two *Malchair 433* specimens. As they do not contain any indication of having been separated from a single specimen, they are treated as syntypes. The two specimens do not differ in quality of the material, so the specimen with the original label was chosen to be the lectotype.

#### Remarks.

Collections from the eastern parts of the distribution (esp. E of the Western Rift) have longer fruits but it appears to be a variable character.

Rarely (*J. Louis 5672*, *J. Louis 13030*), the lower leaf lamina and the adaxial petiole side have short trichomes and the upper lamina has some long trichomes. These features are unusual, but the other characters match the species.

Although *Coccinia subsessiliflora* is nested within *Coccinia barteri* in the molecular tree from plastid markers (Fig. 17), it can be regarded as a proper morphospecies. The deeply lobate leaves are a distinct character, and the species is distributed only in the Congo Basin and the eastern rainforests. The present author supposes that *Coccinia subsessiliflora* might have evolved peripatrically in an arid period of the Pliocene/early Pleistocene period, eventually near the Kivu Mts, and *Coccinia barteri* populated these areas later on.

#### Specimens examined.

(Selection; in total: 21) Burundi. Bubanza: Bubanza, *J. Lewalle 6504* (BR, EA); ibid., *M. Reekmans 1477* (BR (2)). Central African Republic. Lobaye: Boukoko, *C. Tisserant (Équipe) 1103* (BM, G, P [P05620797], P [P05620798]); ibid., *2276* (BM, G, P [P05620796], P [P05620799], P [P05621163]). D.R. Congo. Équateur: [Nord-Ubangi district] Businga territoire, between Karawa and Businga, *J. Lebrun 1928* (BR, P [P05620794], WAG [WAG0225403]). Maniema: between Kindu and Katakokombe, *J. Lebrun 6011* (P [P05620791], WAG [WAG0225402]). North Kivu: Beni territory, Kiandolili river, Gongobotsi Camp of Albert National Park guards, *H. Fredericq in Herb. G.F. de Witte 8288* (BR, M, PRE, WAG [WAG0225407]). Orientale: Haut-Uélé district, Faradje territoire, Kurukwata (Aba), *P. Gerard 3564* (BR, EA, WAG [WAG0225405]); Ituri district, Mambasa territoire, Réserve de Faune à Okapi, Epulu, 1°25'N, 28°35'E, *C.E.N. Ewango 2290* (M, MO); Yangambi, *J. Louis 13030* (BR [BR0000008912916], BR [BR0000008913272], P [P05620795], WAG [WAG0225404]). South Sudan. Eastern Equatoria: Torit district, Lotti Forest, *J.K. Jackson 3026* (K). Uganda. Western Region: [Masindi district], Bunyoro, Bujenje county, Budongo Forest, *A.B. Katende K2801* (MO); ibid. *T.J. Synott 1322* (EA).

### 25. Coccinia trilobata

Taxon classificationPlantaeCucurbitalesCucurbitaceae

(Cogn.) C.Jeffrey, Kew Bull. 15: 349. 1962.

Peponia parviflora var. trilobata Cogn., Bot. Jahrb. Syst. 21: 210. 1895. *Peponia trilobata* (Cogn.) Engl., Pflanzenw. Ost-Afr. C: 399. 1895, nom. illeg. [*Peponia* is a diatom genus]. *Peponium trilobatum* (Cogn.) Engl., Engl. & Prantl, Pflanzenfam., Nachtr.: 318. 1897.
Coccinia trilobata Type: Tanzania. Kilimanjaro: Mkuu [c. 3°10'S, 37°36'E], 1500 m, in hedges, fl, fr, Mar 1894, *G. Volkens 1956* (Holotype: B, destroyed; lectotype acc. to sheet, but not published, so designated here: BR! [BR0000008887160, digital image: BR, JPS]; isolectotype: BR!).
Coccinia kilimandjarica Cogn. ex Harms in Fries, Notizbl. Bot. Gart. Berlin-Dahlem 8: 489. 1923.
Coccinia trilobata Type: Tanzania. Kilimanjaro: Kibohöhe [farm at c. 3°15'50"S, 37°12'0"E], 1100–1200 m, fl, *R. Endlich 52a* (Holotype; B, destroyed; lectotype, designated here: M! [M0105772, “1122”, digital image: JPS], isolectotype: H!).
Coccinia kilimandjarica var. subintegrifolia Cogn. ex Harms in Fries, Notizbl. Bot. Gart. Berlin-Dahlem 8: 490. 1923.
Coccinia trilobata Type: Tanzania. Kilimanjaro: Kibohöhe, 1100–1200 m, fl, *R. Endlich 52* (Holotype: B, destroyed; lectotype, designated here: M! [M0105773, “1121”, digital image: JPS], isolectotype: H!).

#### Description.

Perennial climber. Stems up to 3 m, with soft, whitish trichomes, at least along nerves. Petiole 1.5–16.5 cm, with whitish trichomes (Fig. 8b). Leaves 2.6–14.5 × 3.2–18 cm, cordate, 5-angulate to 5-lobate, sometimes lobulate. Margin denticulate. Apex at least of central lobe acute, often acuminate. Upper leaf surface with hyaline to white pustules and usually with white trichomes. Lower leaf surface more or less covered with soft trichomes, denser on nerves. Probracts up to 1.5 mm or missing. Tendrils simple. Male flowers solitary or in short few-flowered racemes. Common peduncle up to 1.7 cm long, pedicel in raceme up to 3.2 cm, each glabrous or with short trichomes. Bracts up to 1 mm. Pedicel in solitary flowers 0.7–4.7 cm, indumentum as in racemes. Perianth tube with articulate trichomes. Calyx lobes 2–5 mm long, lineal, erect to reflexed. Corolla 0.7–2.2 cm, orange-yellow to reddish-orange, deeper colored on the inner side of the lobes, outside with green venation, lobes 0.7–1.3 cm. Filament column pale yellowish green, anther head a bit darker than filament column. Pollen sacs yellow. Female flowers 1(–2) solitary, pedicel 0.5–4 cm, glabrous or sparsely covered with short trichomes. Hypanthium with articulate trichomes, calyx lobes and corolla like in males. Ovary with trichomes, becoming glabrous towards fruit ripening. Style and stigmas not seen. Fruits ovoid to oblong, 4–4.7(–9) × 2 cm, unripe green with white longitudinal stripes that develop a dark green corona during ripening, ripe fruits orange-red. Seeds 6.5–7 × 2.5–3.5 × 1.5 mm (L/W/H), more or less asymmetrically obovate, face flat (Fig. 14c).

#### Phenology.

Flowering time: January, May–July, October–December.

#### Distribution.

Fig. 33. Tanzania (Arusha, Kilimanjaro, Tanga), Kenya (Central, Coast, Eastern, Nairobi, southern Rift Valley Province). Elevation 1100–2100 m. Red soil, black soil. Open forest, savannas, evergreen bushland, shrubland, grassland.

#### Use.

Leaves eaten as vegetable (*Coilly? 24*, *F. Msajiri 19*).

#### Vernacular names.

Dholuo: angwe (*G.R. Williams 307*), Kinandi: notondwe (*G.R. Williams 307*), Meru: katakeru (*Coilly? 24*), Kikuyu: kigerema (*P. Njogu EA13835*), Kipare: itotwe (*W.J. Kindeketa 648*).

#### Remarks.

The fruits are reported to be poisonous (*G.R. Williams 307*).

There are some collections that have a mixed (not intermediate) phenotype with *Coccinia microphylla*: the calyx lobes are unusually long (up to 7 mm), which is a strong argument for *Coccinia trilobata*, but the indumentum matches *Coccinia microphylla*. These morphs do not occur in single location but are found in the Ndoto Mts (*O. Kerfoot 2644*), in Koboko (*P. Kirika et al. 002/020/2011*), and around Voi (*M. Hucks 579*, *B. Verdcourt 3888*, *R. Polhill & S. Paulo 962*). Whether these are hybrids (F2 or later) or just a variation is not known. These collections look also quite like *Coccinia megarrhiza*, which occurs in northern Kenya and Ethiopia, however, the indumentum does not match either. A clarification where these collections belong to would require sequence data and a better understanding of the plastid and nuclear haplotypes in the three species, which is not available so far.

The collections in the Usambara Mts are often quite glabrous or the trichomes are minute and thus easy to mix up with *Coccinia microphylla*.

#### Specimens examined.

(Selection, in total: 63) Kenya. Central Province: South Nyeri district, S of road (D450), c. 4 km E of Nairobi–Nanyuki road, 3 km N of Kiganjo, *S.S. Hooper & C.C. Townsend 1697* (K [K000353353]). Eastern Province: Nkunga Crater Lake, *P.A. Luke et al. 7256* (EA). Nairobi: Nairobi river Valley, Chiromo, 1°16'30"S, 36°48'E, *R.B. Faden & A.J. Faden 74/822* (BR, DSM, EA, MO, WAG [WAG0234201]). Rift Valley Province: Naivasha District [Nakuru district], Ol Longonot Estate, *O. Kerfoot 3543* (EA, S [S08-12472]). Tanzania. Arusha: Small Momela Lake, *H.M. Richards 20036* (EA (2), K [K000353413]). Kilimanjaro: valley slopes near Alt Moschi [Old Moshi], *A. Peter 56453* (B). Tanga: western Usambara Mts, Mombo–Soni road, *R.B. Drummond & J.H. Hemsley 3007* (B, EA, K, LISC, S [S08-12484]).

### Insufficiently known taxa

#### Coccinia sp. A

Taxon classificationPlantaeCucurbitalesCucurbitaceae

C.Jeffrey, Fl. Zambes.: 450. 1978.

Coccinia sp. A Zambia. Northern Province: Chilongowelo, Tasker’s Deviation waterfall, 4900 ft, female, fl, 27 Feb 1952, *H.M. Richards 883* (K!).

##### Distribution.

Only known from single collection.

##### Remarks.

Jeffrey (1978) suggested that this collection is allied with *Coccinia barteri*. The inflorescence matches that of *Coccinia racemiflora*, which differs in an urceolate perianth tube/hypanthium, more coriaceous leaves, and a glabrous surface. Simple tendrils also occur in *Coccinia barteri*, so this is not a good distinctive character. As the plastid haplotypes of *Coccinia racemiflora* are nested in *Coccinia barteri*, and the distribution of *Coccinia* sp. A is within the *Coccinia barteri* range, Jeffrey’s hypothesis is sound. However, the corolla lobes are quite long, and it seems that the corolla is open campanulate. Therefore, there is some similarity with *Coccinia mildbraedii* and even with *Coccinia grandiflora*.

#### Coccinia sp. B

Taxon classificationPlantaeCucurbitalesCucurbitaceae

C.Jeffrey, Fl. Zambes.: 450. 1978.

Coccinia sp. B Zambia. Southern Province: Mazabuka, on Nanga Estate near Kafue pilot polder [c. 15°45'S, 27°54'E], female, fl, 7 Mar 1963, *H.J. van Rensburg 1620* (K!).

##### Distribution.

Only known from single collection.

##### Remarks.

Like *Coccinia* sp. A, Jeffrey (1978) suggested a closer relationship to *Coccinia barteri*. The tubular corolla and the coriaceous leaves support that. The collection was found in riverine bush in *Acacia* woodland and *Hyparrhenia*/*Setaria* grassland in a flood plain area, which is unusual for *Coccinia barteri* as it rather occurs in (rain) forests. White (1983a) calls the phytochorion of that region “edaphic grassland with semi-aquatic vegetation”. On the one hand, the local soil conditions are not known, and water might be available throughout the year, on the other hand this individual might also represent a local adaptation towards increased drought tolerance.

### Dubious names

***Coccinia aostae* Buscal. & Muschl., Bot. Jahrb. Syst. 59: 499. 1913.**

Type: [Eastern Africa]. At Mbusi river [authors state that this river flows into the Indian Ocean in Mozambique, the collector went upstream towards Zambezi river and Victoria falls; most likely the Buzi River is meant], tree steppe, fl, 14 Dec 1909, *H. von Aosta* [H.L.F.H. d’Orléans] *105* (Syntype: B destroyed; duplicate ?).

This species is supposed to be from Mozambique. However, the describing author, Muschler, provoked a scandal with this work as Georg Schweinfurth (1915) and his former supervisor Adolf Engler (Engler et al. 1915; Ryding 2001) accused him of fraud. Gilg, who contributed corrections in the Cucurbitaceae, suggested that *Coccinia aostae* had been described using the *G.A. Schweinfurth 578* specimen from Eritrea, which bore the ms. name *Coccinia lalambae* Schweinf. A drawing of this species exists in BR! (K neg. 4887), which likely represents a *Coccinia adoensis*. However, the name for *G.A. Schweinfurth 578* remains unpublished and the *von Aosta 105* specimen is destroyed, and the name remains dubious. According to the describing authors, duplicates of the von Aosta specimens have been distributed, and White (1962) found some in Florence (FI or FT). However, a loan from FT did not contain any *Coccinia* collections by von Aosta.

***Coccinia buettneriana* Cogn., Bull. Acad. Roy. Sci. Belgique ser. 3, 14: 351. 1887.**

Type: Gabon. No detailed information given, Sep 1884, *R. Büttner 18* (Holotype: B destroyed).

Cogniaux and Harms synonymize (1924) *Coccinia buettneriana* under *Momordica gabonii* Cogn. (1881) as it was collected in close vicinity of *Büttner 17* (*Momordica gabonii*), which is, according to Cogniaux himself almost not distinguishable from *Coccinia buettneriana*.

***Coccinia calantha* Gilg, Bot. Jahrb. Syst. 34: 358. 1904.**

Type: Tanzania. [Tanga]: Usambara Mts, Duga, near Nikunde village, 100 m, in bush and on fencing, fl, Jul, *C.H.E.W. Holst 3190* (Holotype: B destroyed; duplicates ?).

As the holotype is destroyed and the description does not give enough sufficient characters to relate *Coccinia calantha* to other species, the name remains dubious. Zimmermann (1922b) presents a drawing of an anther, but the thecae are too narrow for a *Coccinia* but would match *Eureiandra* species. On the other hand, *Eureiandra* has free petals, whereas *Coccinia calantha* ought to be sympetalous.

***Coccinia helenae* Buscal. & Muschl., Bot. Jahrb. Syst. 59: 498. 1913.**

Type: [Eastern Africa]. At Mbusi river [authors state that this river flows in the Indian Ocean in Mozambique, the collector went upstream towards Zambezi river and Victoria falls; likely the Buzi river is meant], steppe, fl, fr, 3 Dec 1910, *H. von Aosta* [H.L.F.H. d’Orléans] *87* (Holotype: B, destroyed; duplicates ?).

As for *Coccinia aostae*, *Coccinia helenae* seems to be mistaken. Gilg (Engler et al. 1915) suggested that *Coccinia helenae* had been described using the *G.A. Schweinfurth 932* collection from Blue Nile. A drawing of this species exists in BR (K neg. 4846). The drawing, if it represents a *Coccinia* (the two subsessile female flowers on one node are suspicious), does not match any species of the present author’s knowledge from Blue Nile area entirely. It might be *Coccinia abyssinica* if it was collected in the Ethiopian highlands or *Coccinia adoensis* but the fruit would be unusually ovoid. However, if it is from the area as given by Muschler, it might be *Coccinia rehmannii*. As the *von Aosta 87* specimen is destroyed the name remains dubious. According to the describing authors duplicates of the *von Aosta* specimens have been distributed, and White (1962) found some von Aosta specimens in Florence (FI or FT). However, a loan from FT did not contain any *Coccinia* specimens by von Aosta.

***Coccinia longipetiolata* Chiov., Fl. somala 2: 223. 1932.**

Type: Somalia. [Jubbada Dexhe/Jubbada Hoose border]: between Afmadù [Afmadow] and Saamoggia, 1926, *P. Gorini 149* (Syntype: FT [K neg. 4851, digital image: JPS]).

Type: Somalia. [Jubbada Dexhe/Jubbada Hoose border]: between Afmadù [Afmadow] and Saamoggia, 1926, *P. Gorini 150* (Syntype: FT [K neg. 4850, digital image: JPS]).

**Remarks.** The specimens are quite poor. No leaf is spread out, and generative characters are missing. However, 7-lobate leaves, according to description, do only occur in *Coccinia samburuensis*, which differs in coriaceous leaves and a serrate margin with glandular teeth. Hence, this species name is not synonymous with any *Coccinia* species. The tendrils in *Coccinia longipetiolata* are almost equally bifid, which is not found in *Coccinia*, especially not in species not from rainforests. The drawing accompanying the protologue shows stipules, but this can only be seen in a single node of *P. Gorini 149*, while the other nodes are more typical of Cucurbitaceae. It shows, however, more likely a bud and a probract of similar sizes that give the impression of stipules. In all, the specimens are likely to belong to the Cucurbitaceae.

There are neither characters supporting a relationship with *Coccinia*, nor characters contradicting it, except for the tendrils. Jeffrey (1967) suggests a relationship to his *Coccinia* sp. E sensu F.T.E.A. (*Jarman 66*), but as this specimen could not be examined by the present author, it cannot be discussed. Therefore, that species is treated as dubious. Eventually, sequencing could give disclosure about the relationships.

### Invalid names

***Coccinia abdallai* Zimm., nom. nud.**

The name is mentioned on *P.W.A. Zimmermann G6594* (EA!) and in Die Cucurbitaceen 2: 8 (1922b) but not described. This is a *Coccinia trilobata*.

***Bryonia acerifolia* D.Dietr., Syn. pl. 5: 367. 1852, nom. nud.**

Dietrich mentions this species in his synopsis as a name by Willdenow. However, no such name by Willdenow is known. As Cogniaux (1881) synonymizes *Bryonia acerifolia* with *Bryonia alceaefolia* Willd., and Dietrich uses the exact same words for *Bryonia acerifolia* as for *Bryonia alceaefolia*, this name is likely just mistaken.

***Bryonia barbata* Buch.-Ham. ex Cogn. in A.DC. & C.DC., Monogr. Phan. 3: 530. 1881, nom. nud.**

If the plate 625 in the East India Company’s Museum is additioned by a printed label with description, then this name might be valid, but Cogniaux (1881) mentioned the name as unpublished. He synonymized it with *Coccinia cordifolia* (see also there), so it is not clear whether *Bryonia barbata* is a *Coccinia grandis* or a *Cucumis maderaspatanus*. However, the epithet suggests the existence of rigid trichomes, which does not match with *Coccinia grandis*.

**Coccinia cordifolia var. triangularis A.Chev., nom. nud.**

This name appeared on labels in P specimens of *A.J.B. Chevalier 8886*, *9527*, and *10934*, and it is apparently unpublished.

***Coccinia crassifolia* H.K.Walter, Naturwissenschaft und Landwirtschaft 9: 33. 1926, nom. nud.**

This is a typographical error of *Caccinia crassifolia* Kuntze, a Boraginaceae.

***Coccinia dubia* Palacký, Lotos 10(4): 70. 1860, nom. nud.**

Palacký cites a *Coccinia dubia*, which was supposed to be described by von Bunge in his “Reliquiae lehmanniae” (von Bunge 1854). However, Palacký mistyped the genus, which is in fact called *Caccinia*, a Boraginaceae.

***Coccinia glandis* nom. nud.**

This is a typographical mistake for *Coccinia grandis* that has been published several times (Tewtrakul et al. 2006, Jiwajinda et al. 2002). This epithet should hence not be used in *Coccinia*.

***Physedra gracilis* A.Chev., Explor. Bot. Afrique occ. franç. 1: 292. 1920, nom. nud.**

Ivory Coast: Bassin de la Moyenne-Sassandra, at Guidéko, *A.J.B. Chevalier 16416* (P! [P05591866, digital image: P]). Ivory Coast: Bassin de la Moyenne-Sassandra, at Guidéko, *A.J.B. Chevalier 19013* (P! [P05591864], P! [P05591865]).

This is a nom. nud. (ICN 32.3) because the note that the plants have yellow flowers cannot be regarded as intended to describe a new species. The specimens are *Coccinia keayana*.

**Cephalandra indica var. triangularis A.Chev., nom. nud.**

This name appeared on a label by Chevalier from October 1908 on *A.J.B. Chevalier 9527*, but apparently was not published.

***Cucumis inedulis* Forssk., Fl. aegypt.-arab.: CXXII. 1755, nom. nud.**

For details, see the Taxonomic remarks of *Coccinia grandis*, to which the name would belong.

***Cephalandra ivorensis* A.Chev., Explor. Bot. Afrique occ. franç. 1: 295. 1920, nom. nud.**

Collections connected to this nomen nudum have been synonymized with *Physedra eglandulosa* (Hook.f.) Hutch. & Dalziel (now *Ruthalicia eglandulosa* (Hook.f.) C.Jeffrey).

***Bryonia lagenaria* E.Mey. ex Drège, Zwei pflanzengeogr. Dokum. 54, 169. 1843, nom. nud.**

This name appears on some Drège collections and is only listed in the work of Meyer. However, the collections are type specimens *Coccinia sessilifolia*.

***Coccinia medica* M.T.H.Khan in: Gottschalk-Batschkus and Green, Handbuch der Ethnotherapien: 377, 384, 517, 537. 2002, nom. nud.**

Khan used this name but without taxonomic context. Most likely, he meant *Coccinia indica*, an illeg. name for *Coccinia grandis*.

***Coccinia monteroi* Hort., Catalogue des graines du Jardin botanique de Bordeaux. 1866.**

The protologue was not available to the present author, but Cogniaux (1881) listed this name as nomen tantum. It is, however, not mentioned in the notes sections of the Bordeaux Garden catalogues from 1866 or 1867 where Naudin described some new species, so it might well be just a nomen nudum.

***Coccinia moshiensis* Zimm., nom. nud.**

Mentioned on *P.W.A. Zimmermann G6599* (EA!). This is a *Coccinia trilobata*.

***Coccinia natalensis* Burtt-Davy, A manual of the flowering plants and ferns of the Transvaal with Swaziland, South Africa 1: 237. 1926, nom. nud.**

*Cephalandra natalensis* Oliv., unknown.

*Coccinia natalensis* (Oliv.) Cogn., unknown.

The names of Cogniaux und Oliver are mentioned in Burtt-Davy and Pott-Leendertz in Ann. Transvaal Mus. 3(3): 121. 1912. However, no citation is given. Neither *Cephalandra natalensis* in mentioned in Daniel Oliver’s Flora of Tropical Africa, nor any Cogniaux publication with this name is known. Burtt-Davy writes in 1926 that the name ‘appears to have been an unpublished MS. name’.

***Coccinia lalambensis* Schweinf. ex Penzig, Atti Congr. Bot. Int. Genova (1892): 342. 1893, nom. inval.**

Eritrea. [Anseba Province]: Monte Lalambensis near Keren, c. 2000 m, 20 Mar, *G.A. Schweinfurth 568* (B, destroyed). [Northern Red Sea Province]: Habab, *J.M. Hildebrandt plant. Habab 1802* (LE?).

No description given, therefore this name is not validly published.

***Cucurbita laevigata* Bl.?, nom. nud.**?

This name is written on a specimen in L herbarium (L0587542). The specimen was part of the collection of C. G. C. Reinwardt but lacks collector, collecting site, and date. One ink-written label solely states “1766.E.5138.” and the species name. Another label, written with a pencil, says “Cucurbita laevigata” “mihi” and “Callelet W[…]”. The last word is unreadable to the present author. Another specimen (L0587515) bears a similar label with “1766.E.5138.”, however without a pencil-written label. Since both specimens are *Coccinia grandis*, *Cucurbita laevigata* would be a synonym, if it had been validly published. A Waitz collection (L0587563) bears the names “*Cucurbita laevigata* Bl.” and “Callelet Bl.”, so the former name is maybe a Blume manuscript name and the latter one is indigenous.

***Bryonia quinquefolia* Noronha, Verh. Bat. Genootsch. 5: 155(8). 1790, nom. nud.**

Miquel (1855) synonymizes this nomen nudum under *Coccinia wightiana* M.Roem., which is *Coccinia grandis* (see Taxonomic remarks there). However, he also synonymizes *Bryonopsis pedata* Hassk., which cites *Bryonia quinquefolia*. The description of *Bryonopsis pedata* mentions lacinate, almost pinnatifid leaves and male flowers in oblong clustered racemes. This does not match *Coccinia grandis* at all but eventually *Diplocyclos palmatus*, which Roemer excluded from *Coccinia indica* as *Coccinia palmata* M.Roem. The identity of *Bryonia quinquefolia* might be solved, if one finds a Noroña specimen stating “*Bryonia quinqueloba*” or “Oyot-kekèp”, the latter name being the Javanese term for this species. However, the epithet rather links to the deeply lobate leaves of *Diplocyclos palmatus*.

***Bryonopsis pedata* Hassk., Cat. hort. bot. bogor.: 189. 1844, nom. nud.**

Hasskarl cites Noroña’s *Bryonia quinquefolia* and a vernacular name “aroy kalanyar beurriet”. The given description of lacinate, almost pinnatifid leaves and male flowers in oblong clustered racemes does not match *Coccinia grandis*. According to Filet (1859), the vernacular name is used in Sundanese and refers to *Bryonia* [*sic*] *pedata* Hassk., two *Trichosanthes* species and *Luffa cordifolia* Bl. None of these names have been referred to *Coccinia*, so it seems unlikely that *Bryonopsis pedata* does.

***Coccinia peterii* Zimm., nom. nud.**

This is an unpublished ms. name on *R. Soleman 6046* (EA!). The specimen, however, is a *Coccinia grandis*.

***Bryonia ruderalis* Zipp. ex Span., Linnaea 15: 206. 1841, nom. illeg. & nom. nud.**

In L herbarium, there is a specimen determined as “*Bryonia ruderalis* Zp.” from Timor (L0587573), which is a Zippelius collection of *Coccinia grandis*. However, the name is a later homonym of *Bryonia ruderalis* Salisb. Additionally, it lacks a description in the publication, so it is a nomen nudum, too.

***Cucurbita schimperiana* Hochst., nom. nud.**

The name was used on printed labels of *G.H.W. Schimper 1570* (effective publication), which lack a proper description (hence a nom. nud.). Under this distribution number, specimens from two different shipments are included. One is taken from package “P. 16 K. no. 4”, collected on 23 Apr 1841 in Djeladjeranné (label on P specimen). The data of this label were used for C. F. F. Hochstetter’s printed labels. The TUB-004724 and TUB-004725 specimens bear a Schimper label from package “P. 10 D. no. 23” from “Landschaft Modat” collected in April 1839. An unnumbered W specimen also notes this collecting site, hence the specimen might be from the same shipment. Specimens of both collections are *Coccinia grandis*.

***Coccinia schultzei* Gilg, Namaland & Kalahari: 697. 1907, nom. nud.**

Apparently a collection by L. Schultze (*Schultze 320a*) in B herbarium, but not validly published by Gilg afterwards. However, if so, then the holotype was burned in the Berlin herbarium fire in 1943.

***Coccinia sericea* Zimm., nom. nud.**

Zimmermann marked the specimen *P.W.A. Zimmermann G6600* (EA!) to be a new species, but B. Verdcourt pointed out on the specimen that Zimmermann never published it. In any case, this specimen belongs to *Coccinia grandis*.

***Bryonia sinuosa* Wall., Numer. List 6716. 1832, nom. nud.**

The present author did not see a specimen with this number, so it cannot be decided whether Cogniaux’ (1881) partial synonymization of *Coccinia cordifolia* refers to *Coccinia grandis* or *Cucumis maderaspatanus*. Wallich himself supposed that this collection is a mix of (*Bryonia*) *Coccinia grandis* and *Melothria indica*.

***Cephalandra sylvatica* A.Chev., Explor. Bot. Afrique occ. franç. 1: 295. 1920, nom. nud.**

Collections connected to this nomen nudum (ICN 32.3) have been synonymized with *Physedra eglandulosa* (Hook.f.) Hutch. & Dalziel (now *Ruthalicia eglandulosa* (Hook.f.) C.Jeffrey).

***Cucurbita triangulata* Hochst. ex Cogn. in A.DC. & C.DC. Monogr. Phan. 3: 532. 1881, nom. nud.**

Cogniaux cites a Schimper specimen (*Iter Abyss. Sect. 3 no. 1202*) that was supposed to be labeled by C. F. F. Hochstetter. There are several sheets with this distribution number in Paris, but only one bears this name. The location is given by “In Semen” [Semien Mts]. The other Paris specimens with this number are from Baria Dikeno (collected on 6 Aug 1853). The collection is a *Coccinia grandis*.

***Coccinia wightii* Miq., Fl. Ned. Ind. 1(1): 1112. 1855, nom. nud.**

Name variation of *Coccinia wightiana* M.Roem. in the index of the book.

### Exluded taxa

#### Physedra bequaertii

Taxon classificationPlantaeCucurbitalesCucurbitaceae

De Wild., Pl. Bequart. 1: 569. 1922.

Physedra bequaertii Type: D.R. Congo. Along the Semliki [river], female, fl, 16 Jun 1914, *J. Bequaert 4791* (Syntypes: BR [BR0000008886330, digital image: BR, JPS], BR [BR0000008887122, digital image: BR, JPS], BR [BR0000008887245, digital image: BR, JPS]).
Physedra bequaertii Type: D.R. Congo. Along Ruthuru river, female, fl, 17 Nov 1914, *J. Bequaert 6315* [*sic*, should be *6215*] (Syntypes: BR [BR0000008886477, digital image: BR, JPS], BR [BR0000008886521, digital image: BR, JPS]).

##### Remarks.

As *Physedra* is a synonym of *Coccinia* but the specimens are belonging to the genus *Bambekea*, the name *Physedra bequaertii* has to be excluded. Jeffrey (1962: 364) published the new combination *Bambekea bequaertii* (De Wild.) C.Jeffrey, however, it is not clear whether this is a species separate from *Bambekea racemosa* Cogn.

#### Coccinia cordifolia

Taxon classificationPlantaeCucurbitalesCucurbitaceae

(L.) Cogn. in A.DC. & C.DC., Monogr. Phan. 3: 529. 1881. pro parte.

Bryonia L., Fl. zeyl.: 168. 1747. *Bryonia cordifolia* L., Sp. pl. 2: 1012. 1763.
Coccinia cordifolia Type: Sri Lanka. No detailed location given, *P. Hermann 354* (Typotype: Herm. Flora zeylanica 2:22, BM! [BM000521582, digital image: BM]).

##### Remarks.

Cogniaux cites *Bryonia cordifolia* sensu Linnaeus’ Species plantarum 2^nd^ edition, where Linnaeus synonymizes Rumphius’ *Vitis alba indica*, which is *Coccinia grandis*. If *Bryonia cordifolia* would have been originally described in 1763, Cogniaux’ choice would have been valid, but *Bryonia cordifolia* was described in 1753 (Species plantarum 1^st^ edition), where *Vitis alba indica* is not mentioned, but only a collection from Hermann herbarium, which is *Cucumis maderaspatanus* L.

#### Coccinia decipiens

Taxon classificationPlantaeCucurbitalesCucurbitaceae

(Hook.f. in Oliv.) Cogn. in A.DC. & C.DC., Monogr. Phan. 3: 539. 1881.

Cephalandra decipiens Hook.f. in Oliv., F. T. A. 2: 552. 1871.
Coccinia decipiens Type: Angola. [Cuanza Norte]: Pungo Adongo, grassland, *F.M.J. Welwitsch 816* (Holotype: BM! [BM000799218], isotypes: COI [COI00005507, digital image: JPS], K [K000313451, digital image: JPS, K], LISU [LISU00214555, digital image: JPS]).

##### Remarks.

The sessile beaked fruits match to the genus *Diplocyclos*, which has been correctly observed by Jeffrey (1962) as *Diplocyclos decipiens* (Hook.f.) C.Jeffrey.

#### Coccinia ecirrhosa

Taxon classificationPlantaeCucurbitalesCucurbitaceae

Cogn., Bull. Herb. Boissier 4(12): 822. 1896.

Coccinia ecirrhosa Type: Somalia. Abdallah, 1891, *C. Keller 106* [sphalm. 116 in l.c.] (Type: BR! [BR0000008885999, digital image: BR, JPS], Z! [Z-000004442, digital image: Z, K neg. 4832]).

##### Remarks.

The type does not contain much material, but the lower surface of a leaf shows a pinnatifid venation pattern, which is unknown in *Coccinia*. Jeffrey (1967) transferred it correctly (Kocyan et al. 2007) to another genus, namely *Cephalopentandra*.

#### Coccinia gabonensis

Taxon classificationPlantaeCucurbitalesCucurbitaceae

Keraudren, Adansonia 8: 40. 1968.

Coccinia gabonensis Type: Gabon. Ogooué-Ivindo: Bélinga, 950–1000 m, male and female, fl, Nov 1964, *N. Hallé 3018* (Holotype: P [P00348266, digital image: JPS, P], isotypes: P! [P00348264, digital image: JPS, P], P! [P00348265, digital image: JPS, P], K! [K000313237, digital image: JPS, K]).

##### Remarks.

This species does certainly not belong to *Coccinia*. It is monoecious, has free petals, and rather large bracts, in contrast to the dioecious, sympetalous *Coccinia* species with much smaller bracts. It rather belongs to *Momordica*.

#### Coccinia macrantha

Taxon classificationPlantaeCucurbitalesCucurbitaceae

nom. nud.

Physedra macrantha Gilg, Bot. Jahrb. Syst. 34: 356. 1904. Type: Liberia. Gran Bassa: Fishtown, in bush, sand, fl, 10 m, 27 Aug 1898, *M. Dinklage 2019* [Cucurbitaceae no. 1846] (Holotype: B! [B 10 0154925, digital image: B, JPS]).

##### Remarks.

The combination *Coccinia macrantha* was only used in B herbarium for storage but apparently never published. *Physedra macrantha* Gilg has been synonymized, correctly, with *Physedra eglandulosa* Hutch. & Dalziel (1928), which is now in the genus *Ruthalicia*.

#### Coccinia obbadiensis

Taxon classificationPlantaeCucurbitalesCucurbitaceae

(Chiov.) Cufod., Supplem.: Enum. Pl. Aethiop. Spermatophyta 35(2): 1050. 1965.

Cephalopentandra obbadiensis Chiov., Fl. somala: 187, tab. 20, fig. 1. 1929.
Coccinia obbadiensis Type: Somalia. [Mudug]: Obbia [Hobyo] Sultanate, between Dolobscìo and Magghiòle, 27 Apr, *G. Stefanini & N. Puccioni 458* (Holotype: FT [FT003569, K neg. 4845, digital image: JPS], isotype: K! [K000313183, digital image: JPS, K).

##### Remarks.

The author notes five stamens, which are a good reason for not including this species in *Coccinia*. The leaves of the specimen on the picture look like these of *Coccinia quercifolia*, which is also excluded from *Coccinia* and separated by Jeffrey (1967).

#### Coccinia quercifolia

Taxon classificationPlantaeCucurbitalesCucurbitaceae

Hutch. et E.A.Bruce, Bull. Misc. Inform. Kew 2: 99. 1941.

Coccinia quercifolia Type: Somalia. [Nugaal]: Boundary Pillar 93, 45°9'E, 8°37'N, 990 m, fl, 6 Oct, *J.B. Gillett 4194* (Holotype: K! [K000313174, digital image: JPS, K], K! [K000313174, digital image: JPS, K]).
Coccinia quercifolia Type: Ethiopia. [Somali Region]: Harradigit [c. 7°45'N, 45°30'E], Apr, *F.L. James & J.G. Thrupp no.*? (Paratype: K?).

##### Remarks.

The leaves have a pinnatifid venation, just like Cephalopentandra (Coccinia) ecirrhosa, but are deeply lobed. Since the leaf form may vary in Cucurbitaceae, it is likely that these specimens belong together, so that the odd leaf venation excludes this type from *Coccinia*. Jeffrey already synonymized this name with *Coccinia ecirrhosa*.

#### Coccinia palmata

Taxon classificationPlantaeCucurbitalesCucurbitaceae

(L.) M.Roem., Syn. pepon.: 93. 1846.

Bryonia palmata L., Sp. pl. 2: 1012. 1753. *Bryonia zeylanica, folio quinquepartito* Burm., Thes. zeylan.: 49. 1737. *Bryonia foliis palmatis lævibus quinquepartitis: laciniis lanceolatis repando-serratis* L., Fl. zeyl.: 146. 1747.
Coccinia palmata Type: [Sri Lanka]. No location given. *P. Hermann 25* (Type lost?).
Coccinia palmata Type: [Sri Lanka]. No location given. *P. Hermann 41* (Type lost?).
Coccinia palmata Type: [Sri Lanka]. No location given. *P. Hermann 62* (Type lost?).
Coccinia palmata Type: [Sri Lanka]. No location given. *P. Hermann 353* [Musæum zeylonicum 2:58] (Lectotype, designated by Jeffrey (1962: 352): BM [BM-000621700]).

##### Remarks.

The name *Coccinia palmata* has been applied illegitimately for *Coccinia mackenii* for a long time due to an overlooked combination. When Wight and Arnott published the name *Coccinia indica*, they cited *Bryonia grandis* L. and also tentatively included the citation of *Bryonia palmata* L. More likely, however, they meant a specimen in Herbarium Madras that was identified as *Bryonia palmata* L. One year after Voigt’s correction to *Coccinia grandis* (L.) Voigt, Roemer (1846) also recognized the seemingly missing combination and that Linnaeus’ *Bryonia palmata* and *Bryonia grandis* indeed referred to different species. Roemer treated them, amongst other species, as *Coccinia grandis* (L.) M.Roem. (nom. illeg.) and *Coccinia palmata* (L.) M.Roem. Roemer cited the second edition of Linnaeus’ Species plantarum (1763), which has the identical description for this species as the first edition, so he explicitely meant *Bryonia palmata* to be a part of *Coccinia*. The *Bryonia palmata* typolectotype belongs to what is now widely called *Diplocyclos*, due to the globose striped fruits easily recognizable as being not part of *Coccinia*. Apart from that, another species from South Africa was described with the name *Cephalandra palmata* E.Mey. ex Sond. (Harvey and Sonder 1862). Cogniaux (1881) accepted this species in *Coccinia* but overlooked *Coccinia palmata* (L.) M.Roem. He thus created an illegitimate *Coccinia palmata* (E.Mey. ex Sond.) Cogn., which has since been used for this species. Holstein and Renner (2010) called attention to this erroneous usage by resurrecting the correct name, *Coccinia mackenii* Naudin ex C.Huber, while *Coccinia palmata* is a synonym of *Diplocyclos palmatus* in spite of its wide usage for *Coccinia mackenii* since then.

#### Coccinia petersii

Taxon classificationPlantaeCucurbitalesCucurbitaceae

Gilg, Bot. Jahrb. Syst. 34: 356. 1904.

Coccinia petersii Type: Mozambique. [Zambézia]: Rios de Sena and Boror, without detailed locality, male and female, on dry ground, 1846, *W.H.C. Peters s.n.* (Holotype: B destroyed, isotype: K).

##### Remarks.

Jeffrey (1967) synonymized this species with *Eureiandra fasciculata* (Cogn.) C.Jeffrey.

#### Coccinia polyantha

Taxon classificationPlantaeCucurbitalesCucurbitaceae

Gilg, Bot. Jahrb. Syst. 34: 356. 1904.

Coccinia polyantha Type: Tanzania. Lindi: Kilwa district, near Mariwe, upon low shrub, in light, slightly wet ground pori, fl, Dec, *W. Busse 512* (Syntype: B destroyed, isosyntype: EA [EA000002139, digital image: JPS]).
Coccinia polyantha Type: Tanzania. [South central Tanzania], Kissaki steppe at Rufiji river, 250 m, on sandy laterite soil, fr, Nov, *Götze 80* (Syntype: B destroyed).

##### Remarks.

The seeds are described as globose to subglobose, but *Coccinia* seeds are rather flat. It is therefore unlikely that this species belongs to *Coccinia*. Jeffrey (1967) synonymized this species with *Eureiandra fasciculata* (Cogn.) C.Jeffrey.

#### Cucumis rheedii

Taxon classificationPlantaeCucurbitalesCucurbitaceae

Kostel., Allg. med.-pharm. Fl. 2: 738. 1833.

Schunambuvalli Rheede, Hort. malab. 8: 21, t. 11. 1688.
Cucumis rheedii Type: drawing in l.c.

##### Remarks.

Cogniaux and Harms (1924) synonymized this species with *Coccinia cordifolia* (L.) Cogn., a name that Cogniaux used for *Coccinia grandis* (L.) Voigt. However, the drawing shows 3–5-fid tendrils and small? subglobose fruits on an ebracteate panicle. This does not correspond to *Coccinia*.

#### Coccinia stefaninii

Taxon classificationPlantaeCucurbitalesCucurbitaceae

Chiov., Result. Sc. Miss. Stefan.-Paoli Somal. Ital. 1: 84. 1916.

Coccinia stefaninii Type: Somalia. Somali Republic, Jubaland, Dintorni di El Uré, *G. Paoli 1069* (Syntype: FT! [2 sheets, FT003512, digital image: JPS]).
Coccinia stefaninii Type: Somalia. Fra Jagdoudou e Duddumai, *G. Paoli 1179* (Syntype: FT! [FT003513, digital image: JPS]).

##### Remarks.

This name has been transferred to the genus *Dactyliandra* by Jeffrey (1985).

#### Coccinia stolzii

Taxon classificationPlantaeCucurbitalesCucurbitaceae

Harms, Bot. Jahrb. Syst. 57: 241. 1923.

Coccinia stolzii Type: Nyasaland [Tanzania]. Kyimbila district, Mbaku, 600 m, fl, fr, Jun 1913, *A.F. Stolz 2028* (Holotype: ?, isotype: BM!, G! [G00301602], K [K000313456, digital image: JPS, K], L!, P! [P05590096, digital image: P], PR!, PRE [PRE0592846-0, digital image: JPS], S [S08-12469, digital image: JPS], U! [U0074536], W!, Z! [Z-000004440, digital image: Z]).

##### Remarks.

Monoecious plant with several small subglobose fruits per node apply clearly to *Diplocyclos* and must therefore be synonymized as it has been done by Jeffrey (1967) to *Diplocyclos decipiens*.

#### Coccinia suburceolata

Taxon classificationPlantaeCucurbitalesCucurbitaceae

Cogn., Symb. Antill. (Urban) 1: 450. 1900.

Coccinia suburceolata Type: Haiti. Near Port-au-Prince, in Tête bois de pin, 1800 m, male, fl, Nov, *L. Picarda 1498* (Holotype: BR! [BR0000009939141, digital image: JPS], isotypes: B, L, NY, S?).

##### Remarks.

This plant was found on Haiti and is therefore geographically far away from the natural distribution range of the genus *Coccinia*. According to the protologue, the tendrils are often trifid and the fruit is spherical and apple-sized, which does not fit to the morphospace of any *Coccinia* species. Urban (1921) put the specimen and thus species, amongst others, into a new genus: *Penelopeia*, which has been confirmed by Kocyan et al. (2007).

## Supplementary Material

XML Treatment for Coccinia

XML Treatment for Coccinia abyssinica

XML Treatment for Coccinia adoensis (Hochst. ex A.Rich.) Cogn. var. adoensis

XML Treatment for Coccinia adoensis var. aurantiaca

XML Treatment for Coccinia adoensis var. jeffreyana

XML Treatment for Coccinia barteri

XML Treatment for Coccinia grandiflora

XML Treatment for Coccinia grandis

XML Treatment for Coccinia heterophylla

XML Treatment for Coccinia hirtella

XML Treatment for Coccinia intermedia

XML Treatment for Coccinia keayana

XML Treatment for Coccinia longicarpa

XML Treatment for Coccinia mackenii

XML Treatment for Coccinia megarrhiza

XML Treatment for Coccinia microphylla

XML Treatment for Coccinia mildbraedii

XML Treatment for Coccinia ogadensis

XML Treatment for Coccinia pwaniensis

XML Treatment for Coccinia quinqueloba

XML Treatment for Coccinia racemiflora

XML Treatment for Coccinia rehmannii

XML Treatment for Coccinia samburuensis

XML Treatment for Coccinia schliebenii

XML Treatment for Coccinia senensis

XML Treatment for Coccinia sessilifolia (Sond.) Cogn. var. sessilifolia

XML Treatment for Coccinia sessilifolia var. variifolia

XML Treatment for Coccinia subsessiliflora

XML Treatment for Coccinia trilobata

XML Treatment for Coccinia sp. A

XML Treatment for Coccinia sp. B

XML Treatment for Physedra bequaertii

XML Treatment for Coccinia cordifolia

XML Treatment for Coccinia decipiens

XML Treatment for Coccinia ecirrhosa

XML Treatment for Coccinia gabonensis

XML Treatment for Coccinia macrantha

XML Treatment for Coccinia obbadiensis

XML Treatment for Coccinia quercifolia

XML Treatment for Coccinia palmata

XML Treatment for Coccinia petersii

XML Treatment for Coccinia polyantha

XML Treatment for Cucumis rheedii

XML Treatment for Coccinia stefaninii

XML Treatment for Coccinia stolzii

XML Treatment for Coccinia suburceolata

## Supplementary materials

Supplementary material 1 List of examined specimens and geo-references of the localities Data type: specimens data. Explanation note: Voucher information and (predominantly inferred) coordinates of geo-references (in decimal degrees). Specimens included in this study, with the geographic origin of material (only country and 1st administrative division given). Herbarium acronyms follow Index Herbariorum; barcodes or other unique identifiers in brackets behind the acronym. Indication of digitized images refer to the homepage of the corresponding institution indicated by the herbarium acronym, except for JPS (JSTOR plant science) and CVH (= Chinese Virtual Herbarium). This dataset is made available under the Open Database License (http://opendatacommons.org/licenses/odbl/1.0/). The Open Database License (ODbL) is a license agreement intended to allow users to freely share, modify, and use this Dataset while maintaining this same freedom for others, provided that the original source and author(s) are credited.Norbert Holstein

Supplementary material 2 GenBank accessions Data type: specimens data. Explanation note: Voucher information and GenBank accession numbers. Specimens included in this study, with the geographic origin of material (only country and 1st administrative division given) and GenBank accession numbers for all sequences. Herbarium acronyms (in parentheses behind the voucher name) follow Index. This dataset is made available under the Open Database License (http://opendatacommons.org/licenses/odbl/1.0/). The Open Database License (ODbL) is a license agreement intended to allow users to freely share, modify, and use this Dataset while maintaining this same freedom for others, provided that the original source and author(s) are credited.Norbert Holstein
