# Supplementary material for: Monograph of Coccinia (Cucurbitaceae)
Source: PhytoKeys. 2015 Aug 3;(54):1–166. doi: 10.3897/phytokeys.54.3285 (PMC4547038; doi:10.3897/phytokeys.54.3285)
Supplement: Supplementary material 2 — GenBank accessions [file phytokeys-054-001-s002.doc]

Table S2. Voucher information and GenBank accession numbers. Specimens included in this study, with the geographic origin of material (only country and 1^st^ administrative division given) and GenBank accession numbers for all sequences. Herbarium acronyms (in parentheses behind the voucher name) follow Index Herbariorum.

| Species | No. | Voucher | Location | *mat*K | *ndh*F–  *rpl*32R IS | *rpl*20–  *rps*12 IS | *trn*L intron | *trn*L–  *trn*F IS | *trn*S–  *trn*G IS | *LFY* 2^nd^ intron |
| --- | --- | --- | --- | --- | --- | --- | --- | --- | --- | --- |
| *C. abyssinica* (Lam.) Cogn. | 1 | *E. Westphal & J.M.C. Westphal-Stevels 1552* (WAG) | Ethiopia, Oromia Region | HQ608224 |  | HQ608311 |  |  | HQ608429 |  |
| *C. abyssinica* (Lam.) Cogn. | 2 | *E. Westphal & J.M.C. Westphal-Stevels 1951* (WAG) | Ethiopia, Oromia Region |  |  | HQ608312 | HQ608385 | HQ608368 | HQ608430 |  |
| *C. abyssinica* (Lam.) Cogn. | 3 | *N. Holstein 120* (M) | From seeds from Ethiopia, Oromia |  |  | JQ943386 |  |  | JQ943393 |  |
| *C. adoensis* (Hochst. ex A.Rich.) Cogn. | 1 | *L.E. Davidson 3781* (M) | South Africa, Gauteng | HQ608226 | HQ608274 | HQ608314 | HQ608396 | HQ608396 | HQ608432 |  |
| *C. adoensis* (Hochst. ex A.Rich.) Cogn. | 2 | *R. Story 6283* (M) | Namibia, Otjozondjupa | HQ608227 | HQ608275 | HQ608316 | HQ608397 | HQ608397 | HQ608434 | HQ608160 |
| *C. adoensis* (Hochst. ex A.Rich.) Cogn. | 3 | *J. Pawek 6124* (MO) | Malawi, Northern Region | HQ608225 |  | HQ608315 |  | HQ608369 | HQ608433 |  |
| *C. adoensis* (Hochst. ex A.Rich.) Cogn. | 4 | *R.E. Gereau &*  *C.J. Kayombo 3582* (MO) | Tanzania, Iringa | HQ608231 | HQ608273 | HQ608313 |  |  | HQ608431 |  |
| *C. adoensis* (Hochst. ex A.Rich.) Cogn. | 5 | *E.A. Robinson 2944* (M) | Zambia, Southern Prov. | HQ608228 |  | HQ608318 | HQ608398 | HQ608398 | HQ608436 |  |
| *C. adoensis* (Hochst. ex A.Rich.) Cogn. | 6 | *H. Merxmüller 282* (M) | South Africa, Gauteng | HQ608229 |  | HQ608319 |  | HQ608370 | HQ608437 |  |
| *C. adoensis* (Hochst. ex A.Rich.) Cogn. | 7 | *M. Sanane 375* (M) | Zambia, Northern Prov. | HQ608230 |  | HQ608320 | HQ608399 | HQ608399 | HQ608438 |  |
| *C. adoensis* (Hochst. ex A.Rich.) Cogn. | 8 | *A.R. Torre 5337* (M) | Mozambique, Zambezia |  |  | HQ608321 |  | HQ608371 | HQ608439 |  |
| *C. adoensis* (Hochst. ex A.Rich.) Cogn. | 9 | *D.K. Harder & M.G. Bingham 2584* (MO) | Zambia, Lusaka Prov. | HQ608268 | HQ608299 | HQ608364 |  |  | HQ608492 | HQ608191 |
| *C. adoensis* (Hochst. ex A.Rich.) Cogn. | 10 | *S.A. Robertson 1925* (MO) | Kenya, Eastern Prov. | HQ608232 |  | HQ608322 | HQ608400 | HQ608400 | HQ608440 |  |
| *C. adoensis* (Hochst. ex A.Rich.) Cogn. | 11 | *H.J.E, Schlieben 3271* (M) | Tanzania, Morogoro | HQ625501 |  | HQ608317 |  |  | HQ608435 |  |
| *C. adoensis* var. *aurantiaca*  C.Jeffrey (Holstein) | 1 | *M. Richards 20987* (BR) | Tanzania, Iringa | HQ608235 |  | HQ625507 | HQ608401 | HQ608401 | HQ608443 |  |
| *C.adoensis* var.*aurantiaca*  C.Jeffrey (Holstein) | 2 | *P.J. Greenway & Kanuri 14811* (M) | Tanzania, Iringa |  |  |  | HQ608402 | HQ608402 | HQ608444 | HQ608161 |
| *C.adoensis* var.*aurantiaca*  C.Jeffrey (Holstein) | 3 | *N. Holstein et al. 86* (M) | Tanzania, Dodoma | HQ608236 | HQ608276 | HQ608325 | HQ608403 | HQ608403 | HQ608445 | HQ608162 |
| *C.* adoensis var. *jeffreyana* Holstein | 1 | *J.C. Lovett 1597* (MO) | Tanzania, Iringa | HQ608233 |  | HQ608323 | HQ608386 | HQ608372 | HQ608441 |  |
| *C.* adoensis var. *jeffreyana* Holstein | 2 | *C.F. Paget-Wilkes 72* (MO) | Tanzania, Iringa | HQ608234 |  | HQ608324 |  | HQ608373 | HQ608442 |  |
| *C.* adoensis var. *jeffreyana* Holstein | 3 | *N. Holstein 125* (M) | From seeds from Kenya, Rift Valley Province | JQ943384 |  | JQ943391 |  |  | JQ943398 |  |
| *C. barteri* (Hook.f.) Keay | 1 | *E. Achigan-Dako 07 NIA 899* (GAT) | Guinea, Nzérékoré Region | HQ608237 |  | HQ608330 | HQ608404 | HQ608404 | HQ608450 |  |
| *C. barteri* (Hook.f.) Keay | 2 | *J.J. Wieringa 6387* (WAG) | Gabon, Haut-Ogooué | HQ608239 | HQ608277 | HQ608326 | HQ608405 | HQ608405 | HQ608446 | HQ608163 |
| *C. barteri* (Hook.f.) Keay | 3 | *E. Achigan-Dako 06 NIA 294* (GAT) | Guinea, Mamou Region |  |  | HQ608331 | HQ608389 | HQ608376 | HQ608451 |  |
| *C. barteri* (Hook.f.) Keay | 4 | *E. Achigan-Dako 07 NIA 809* (GAT) | Ghana, Eastern Region | HQ608240 |  | HQ608327 | HQ608387 | HQ608374 | HQ608447 | HQ608164 |
| *C. barteri* (Hook.f.) Keay | 5 | *W.J.J.O. de Wilde et al. 3726* (MO) | Cameroon, Central Region | HQ608241 |  | HQ608328 | HQ608388 | HQ608375 | HQ608448 |  |
| *C. barteri* (Hook.f.) Keay | 6 | *M.A. van Bergen 490* (WAG) | Gabon, Ogooué-Maritime | HQ608242 | HQ608278 | HQ608329 | HQ608406 | HQ608406 | HQ608449 | HQ608165 |
| *C. barteri* (Hook.f.) Keay | 7 | *F.J. Fernández-Casas 12077* (MO) | Equatorial Guinea, Bioco Island | HQ608238 | HQ608279 | HQ608332 | HQ608390 | HQ608377 | HQ608453 |  |
| *C. barteri* (Hook.f.) Keay | 8 | *E. Achigan-Dako 07 NIA 875* (GAT) | Guinea, Nzérékoré Region |  |  |  |  |  | HQ608452 |  |
| *C. grandiflora* Cogn. | 1 | *H. Schäfer 05/302* (M) | Tanzania, Tanga | HQ608243 | HQ608280 | HQ608333 | HQ608407 | HQ608407 | HQ608454 | HQ608166 |
| *C. grandiflora* Cogn. | 2 | *N. Holstein et al. 98* (M) | Tanzania, Tanga | HQ608244 | HQ608281 | HQ608334 | HQ608408 | HQ608408 | HQ608455 | HQ608167 |
| *C. grandis* (L.)  Voigt | 1 | *W.J.J.O. de Wilde & B.E.E. Duyfjes*  *22270* (L) | Thailand, Bangkok | DQ536651 | HQ608282 | DQ536537 | DQ536762 | DQ536762 | HQ608456 | HQ608168 |
| *C. grandis* (L.)  Voigt | 2 | *R. Müller s.n.,* Aug. 1999 (MSB) | Sudan, Sannar Prov. |  |  | HQ608335 | HQ608409 | HQ608409 | HQ608457 | HQ608169 |
| *C. grandis* (L.)  Voigt | 3 | *H. Schäfer 05/258* (M) | Tanzania, Pwani | HQ608245 | HQ608283 | HQ608336 | HQ608410 | HQ608410 | HQ608458 | HQ608170 |
| *C. heterophylla* (Hook.f.) Holstein |  | *C.C.H. Jongkind 5905* (WAG) | Gabon, Estuaire | HQ608246 |  | HQ608337 | HQ608411 | HQ608411 | HQ608459 | HQ608171 |
| *C. hirtella* Cogn. | 1 | *N. Holstein 29* (M) | J.-L. Gatard, France, wild source unknown | HQ608247 | HQ608284 | HQ608339 | HQ608412 | HQ608412 | HQ608461 | HQ608172 |
| *C. hirtella* Cogn. | 2 | *S.S. Renner & A. Kocyan 2447* (M) | J.-L. Gatard, France, wild source unknown | HQ608248 |  | HQ608338 | HQ608413 | HQ608413 | HQ608460 |  |
| *C. intermedia* Holstein | 1 | *C. Geerling & J. Bokdam 662* (MO) | Ivory Coast, Bouna area | HQ608269 | HQ608298 | HQ608363 |  | HQ608383 | HQ608491 |  |
| *C. intermedia* Holstein | 2 | *A. Akoègninou et al. 3625* (WAG0278370) | Benin, Atakora | JQ943382 | JQ943380 | JN653687 |  |  | JN653686 | JN653688 |
| *C. keayana*  R.Fern. | 1 | *F.C. Straub 140* (BR) | Liberia |  |  |  |  |  | HQ608462 |  |
| *C. keayana*  R.Fern. | 2 | *C.C H. Jongkind et al. 6542* (WAG) | Liberia, Grand Gedeh | HQ608249 | HQ608285 | HQ608340 |  | HQ608378 | HQ608463 | HQ608173 |
| *C. longicarpa* Jongkind |  | *C.C.H. Jongkind 3970* (WAG) | Ghana, Ashanti Region | HQ608250 | HQ608286 | HQ608341 | HQ608414 | HQ608414 | HQ608464 | HQ608174 |
| *C. mackenii* Naudin ex  C.Huber |  | *R.G. Strey 3762* (M) | South Africa, Mpumalanga | HQ608251 |  | HQ608343 | HQ608415 | HQ608415 | HQ608465 |  |
| *C. megarrhiza*  C.Jeffrey | 1 | *J.J.F.E. de Wilde 6501* (WAG) | Ethiopia, Oromia Region |  |  | HQ608344 | HQ608417 | HQ608417 | HQ608466 |  |
| *C. megarrhiza*  C.Jeffrey | 2 | *I. Friis et al. 2664* (MO) | Ethiopia, Oromia Region | HQ608252 | HQ608287 | HQ608347 | HQ608416 | HQ608416 | HQ608469 | HQ608176 |
| *C. megarrhiza*  C.Jeffrey | 3 | *P.C.M. Jansen 3471* (WAG) | Ethiopia, Oromia Region |  |  | HQ608345 |  |  | HQ608467 |  |
| *C. megarrhiza*  C.Jeffrey | 4 | *J.J.F.E. de Wilde* *4793* (WAG) | Ethiopia, Oromia Region | HQ608253 |  | HQ608346 |  |  | HQ608468 | HQ608175 |
| *C. megarrhiza*  C.Jeffrey | 5 | *N. Holstein 118* (M) | From seeds from Ethiopia, Oromia Region |  |  | JQ943387 |  |  | JQ943394 |  |
| *C. microphylla* Gilg | 1 | *R.B. Drummond & J.H. Hemsley 4087* (B) | Kenya, Coast Province | HQ608254 |  | HQ608348 |  |  | HQ608470 | HQ608177 |
| *C. microphylla* Gilg | 2 | *J.J.F.E. de Wilde & M.G. Gilbert 346* (UPS) | Ethiopia, Somali Regional State | HQ608255 |  | HQ608349 | HQ608418 | HQ608418 | HQ608471 | HQ608178 |
| *C. microphylla* Gilg | 3 | *N. Holstein 134* (M) | From seeds from Tanzania |  |  | JQ943388 |  |  | JQ943395 |  |
| *C. mildbraedii* Gilg ex Harms | 1 | *M. Reekmans 7399* (BR) | Burundi, Muramvya Prov. | HQ608256 |  | HQ608350 |  |  | HQ608472 |  |
| *C. mildbraedii* Gilg ex Harms | 2 | *N. Holstein et al. 76* (M) | Tanzania, Morogoro | HQ608257 | HQ608288 | HQ608351 | HQ608419 | HQ608419 | HQ608473 | HQ608179 |
| *C. ogadensis* Thulin |  | *M. Thulin et al. 11183* (UPS) | Ethiopia, Somali Regional State | HQ608258 | HQ608289 | HQ608352 |  |  | HQ608474 |  |
| *C. quinqueloba* (Thunb.) Cogn. |  | *R.D.A. Bayliss 8470* (M) | South Africa, Eastern Cape | HQ608259 | HQ608290 | HQ608353 | HQ608420 | HQ608420 | HQ608475 | HQ608180 |
| *C. racemiflora* Kéraudren | 1 | *I. van Nek 536* (WAG) | Gabon, Ogooué-Maritime |  |  | HQ608355 | HQ608421 | HQ608421 | HQ608477 | HQ608182 |
| *C. racemiflora* Kéraudren | 2 | *J.J. Bos 6590* (WAG) | Cameroon, South Prov. | HQ608260 |  | HQ608354 | HQ608391 | HQ608379 | HQ608476 | HQ608181 |
| *C. rehmannii* Cogn. | 1 | *S.S. Renner & A. Kocyan 2749* (M) | southern Africa, no detailed information | DQ536652 | HQ608292 | HQ625508 | DQ536799 | DQ536799 | HQ608479 | HQ608184 |
| *C. rehmannii* Cogn. var. *littoralis*  A. Meeuse | 2 | *L.E. Codd 9620* (M) | South Africa, KwaZulu-Natal | HQ608261 |  | HQ625509 | HQ608422 | HQ608422 | HQ608480 |  |
| *C. rehmannii* Cogn. var. *rehmannii* | 3 | *G. Woortman 217* (M) | Namibia, Otjozondjupa | HQ608262 |  | HQ625510 | HQ608392 | HQ608380 | HQ608481 | HQ608185 |
| *C. rehmannii* Cogn.*“ovifera”* | 4 | *B. de Winter &*  *O.A. Leistner 5598* (M) | Namibia, Kunene | HQ608263 | HQ608291 | HQ608356 | HQ608423 | HQ608423 | HQ608478 | HQ608183 |
| *C. rehmannii* aff. var. *littoralis* A.Meeuse | 5 | *N. Holstein 126* (M) | From seeds USDA 365066, wild coll. from SE South Africa | JQ943383 |  | JQ943390 | HQ625496 | HQ625496 | JQ943396 | JQ943381 |
| *C. samburuensis* Holstein |  | *R.B. & A.J. Faden 74/948* (WAG) | Kenya, Rift Valley Prov. | HQ608264 | HQ608293 | HQ608357 | HQ608393 | HQ608381 | HQ608482 | HQ608186 |
| *C. schliebenii* Harms | 1 | *E. Westphal & J.M.C. Westphal-Stevels 5539* (WAG) | Ethiopia, Oromia Region |  | HQ608294 | HQ608358 |  |  | HQ608483 |  |
| *C. schliebenii* Harms | 2 | *G.S. Laizer et al. 1449* (MO) | Tanzania, Morogoro | HQ608265 |  | HQ608359 |  | HQ608382 | HQ608484 | HQ608187 |
| *C. senensis* (Klotzsch) Cogn. | 1 | *N. Holstein et al. 66* (M) | Tanzania, Morogoro | HQ608266 | HQ608295 | HQ608360 | HQ608424 | HQ608424 | HQ608485 | HQ608188 |
| *C. senensis* (Klotzsch) Cogn. | 2 | *K. Vollesen MRC4316* (WAG) | Tanzania, Lindi | HQ608267 | HQ608296 | HQ608362 | HQ608425 | HQ608425 | HQ608487 | HQ608189 |
| *C. senensis* (Klotzsch) Cogn. | 3 | *A.R. Torre et al. 18788* (MO) | Mozambique, Tete |  |  | HQ608361 |  |  | HQ608486 |  |
| *C. senensis* (Klotzsch) Cogn. | 4 | *E.M.C. Groenendijk et al. 1031* (WAG) | Mozambique, Nampula |  |  | HQ625511 |  |  | HQ608489 |  |
| *C. senensis* (Klotzsch) Cogn. | 5 | *H.J.E. Schlieben 5259* (M) |  |  |  |  |  |  | HQ608488 |  |
| *C. sessilifolia* (Sond.) Cogn. var. *sessilifolia* |  | *S.S. Renner et al. 2763* (M) | Plant grown at Mainz Bot. G. (MJG19-54430); wild source unknown | AY968446 | HQ608297 | DQ648163 | AY968568 | AY968385 | HQ608490 | HQ608190 |
| *C. sessilifolia* var. *variifolia* (A.Meeuse) Holstein |  | *F.A. Rogers 24932* (Z000073427) | South Africa, Limpopo | JQ943385 |  | JQ943392 |  |  | JQ943399 |  |
| *C. subsessiliflora* Cogn. |  | *H. Fredericq in* *Herb. G.F. de Witte 8288* (M) | DR Congo, North Kivu | HQ608270 |  | HQ608365 | HQ608395 | HQ608384 | HQ608493 |  |
| *C. trilobata* (Cogn.)  C.Jeffrey | 1 | *N. Holstein & P. Sebastian 9* (M) | From seeds from J.-L. Gatard, France, coll. in Kenya | HQ608271 | HQ608300 | HQ608366 | HQ608426 | HQ608426 | HQ608494 |  |
| *C. trilobata* (Cogn.)  C.Jeffrey | 2 | *N. Holstein 135* (M) | From seeds from Kenya, Rift Valley Prov. |  |  | JQ943389 |  |  | JQ943397 |  |
| *Diplocyclos palmatus* (L.)  C.Jeffrey |  | *J. Maxwell s.n.* 2 Sep. 2002 | Thailand, Chiang Mai | DQ536671 | HQ608301 | DQ536625 | DQ536769 | DQ536769 | HQ608495 | HQ608192 |
| *Diplocyclos schliebenii* (Harms)  C.Jeffrey |  | *H.J.E. Schlieben 4363* (M) | Tanzania, Kilimanjaro |  |  |  | HQ608427 | HQ608427 | HQ608496 | HQ608193 |
| *Cucumis hirsutus* Sond. |  | *N.B. Zimba et al. 874* (MO) | Zambia | DQ536658 |  | DQ536542 | DQ536804 | DQ536804 | HM597074 |  |
| *Cucumis sativus* L. |  | unknown | unknown | AJ970307 | AJ970307 | AJ970307 | AJ970307 | AJ970307 | AJ970307 |  |
| *Peponium vogelii* (Hook.f.) Engl. |  | *S.S. Renner 2710* (M) | Tanzania, Tanga | HQ608272 | HQ608302 | HQ608367 | HQ608428 | HQ608428 | HQ608497 | HQ608194 |
| *Scopellaria marginata* (Bl.) W.de Wilde and Duyfjes |  | *A. Kocyan AK178* (BKF) | Thailand | DQ536751 |  | DQ536612 | DQ536804 | DQ536804 |  |  |
